# A novel taxonomic marker that discriminates between morphologically complex actinomycetes

**DOI:** 10.1098/rsob.130073

**Published:** 2013-10

**Authors:** Geneviève Girard, Bjørn A. Traag, Vartul Sangal, Nadine Mascini, Paul A. Hoskisson, Michael Goodfellow, Gilles P. van Wezel

**Affiliations:** 1 Molecular Biotechnology, Institute of Biology, Leiden University, PO Box 9505, 2300 RA Leiden, The Netherlands; 2 Department of Molecular and Cellular Biology, Harvard University, 16 Divinity Avenue, Cambridge, MA 02138, USA; 3 Strathclyde Institute of Pharmacy and Biomedical Sciences, University of Strathclyde, Glasgow, UK; 4 School of Biology, Newcastle University, Ridley Building, Newcastle upon Tyne NE1 7RU, UK

**Keywords:** *Streptomyces*, cell division, systematics, genome sequencing

## Abstract

In the era when large whole genome bacterial datasets are generated routinely, rapid and accurate molecular systematics is becoming increasingly important. However, 16S ribosomal RNA sequencing does not always offer sufficient resolution to discriminate between closely related genera. The SsgA-like proteins are developmental regulatory proteins in sporulating actinomycetes, whereby SsgB actively recruits FtsZ during sporulation-specific cell division. Here, we present a novel method to classify actinomycetes, based on the extraordinary way the SsgA and SsgB proteins are conserved. The almost complete conservation of the SsgB amino acid (aa) sequence between members of the same genus and its high divergence between even closely related genera provides high-quality data for the classification of morphologically complex actinomycetes. Our analysis validates *Kitasatospora* as a sister genus to *Streptomyces* in the family Streptomycetaceae and suggests that *Micromonospora*, *Salinispora* and *Verrucosispora* may represent different clades of the same genus. It is also apparent that the aa sequence of SsgA is an accurate determinant for the ability of streptomycetes to produce submerged spores, dividing the phylogenetic tree of streptomycetes into liquid-culture sporulation and no liquid-culture sporulation branches. A new phylogenetic tree of industrially relevant actinomycetes is presented and compared with that based on 16S rRNA sequences.

## 2. Introduction

The next-generation sequencing revolution has seen the number of genome sequences publically released accelerate at an extraordinary rate, with microbial genomes published on a daily basis [1]. At present, even sequencing the metagenomes of complex microbial environments seems almost common place. Still, we are only at the beginning, and new technological advances will further accelerate the accumulation of genome sequence information. The sequences of some 8000 bacterial genomes are publically available, including many organisms classified in the phylum Actinobacteria [2]. Members of this taxon, notably streptomycetes, produce around 70% of known antibiotics, and are therefore an important asset in the fight against emerging antibiotic resistance [3,4]. Following the publication of the genome sequence of the model actinomycete *Streptomyces coelicolor* A3(2) a decade ago [5], the sequences of a large number of *Streptomyces* and other actinobacterial genomes have been made available (http://www.genomesonline.org). These developments underline the need for rapid and, at the same time, accurate classification of these commercially and environmentally significant organisms.

Current approaches to the classification of prokaryotes are based on the integrated use of genotypic and phenotypic data, that is, on polyphasic taxonomy [6–8]. This approach is being driven increasingly by advances in molecular biology, as witnessed by the impact that 16S rRNA gene sequence and DNA : DNA relatedness values are having on the delineation of taxa, especially at the rank of species [9,10]. The widespread use of polyphasic taxonomic procedures has led to spectacular improvements in the classification of taxa belonging to the phylum Actinobacteria [2]. Despite this progress, significant problems remain, and with so many related species in genera such as *Streptomyces*, the resolution offered by 16S rRNA and associated phenotypic markers is not always sufficient for the recognition of new taxa. There is a particular need to establish the taxonomic status of closely related genera within morphologically complex actinomycetes, such as those classified in the families Micromonosporaceae and Streptomycetaceae [11,12]. There is, for instance, a pressing requirement to determine whether the genus *Streptomyces* is paraphyletic or whether the inclusion of *Kitasatospora* and *Streptacidiphilus* species within the evolutionarily radiation of this taxon merely reflects insufficient variation in the constituent 16S rRNA gene sequences [13]. Indeed, the circumscription of genera, as opposed to species, is currently highly subjective within the prokaryotes as a whole [14].

Recent observations suggest that highly conserved sequences of the SsgA-like proteins (SALPs), which play an important role in morphogenesis and control of developmental cell division in actinomycetes with complex life cycles, may provide a reliable means of distinguishing between members of closely related actinobacterial genera [15,16]. A number of streptomycetes, such as *Streptomyces granaticolor*, *Streptomyces griseus*, *Streptomyces roseosporus* and *Streptomyces venezuelae*, sporulate not only on surface-grown but also in liquid-grown cultures [17–19]; comparative analyses of the highly conserved protein sequences may establish whether such organisms are evolutionarily more strongly related to one another than to streptomycetes which sporulate only on surface-grown culture.

Members of the SALP protein family are typically between 130 and 145 amino acids (aa) long, with 30–50% aa identity between them. SALPs occur exclusively in morphologically complex actinomycetes, and there is a suggested linkage between the number of paralogues and the complexity of the developmental process in these organisms [16,20]. Actinomycetes that produce single spores typically contain a single SALP (invariably SsgB), those that produce short spore chains typically have two, and those that undergo more complex development typically have multiple SALPs; *Frankia* species, which produce a large sporangium, have three to five SALPs and *Streptomyces* species, which form long spore chains, generally have six to eight SALPs. The model organism *S. coelicolor* A3(2) contains seven SALPs (SsgA–G), and of these, SsgA, SsgB and SsgG are cell division proteins, with SsgA and SsgB essential for sporulation [21,22]; SsgD is required for cell wall integrity; SsgE and SsgF play a role in spore maturation; and SsgC may act as an antagonist of SsgA [23].

SsgA was identified as a sporulation protein in *S. griseus* [24], and enhanced expression of SsgA affects fragmentation of mycelia in liquid-grown cultures [25,26]. SsgA is required for both solid- and liquid-culture sporulation of streptomycetes and is a key connection between these two types of cell division. SsgA localizes to the sites where cell-wall remodelling takes place and is involved in the activation of spore germination and cell division [27]. SsgB is the archetype of the SALPs as it is found in all actinomycetes that have one or more of these proteins [16]. The crystal structure of SsgB from *Thermobifida fusca* was determined at 2.6 Å resolution [28]. This revealed a bell-shaped trimer with intriguing structural similarity to the mitochondrial guide RNA-binding proteins MRP1 and MRP2 [29] and the ssDNA-binding protein PBF-2 [30]. The SsgB protein is part of the cell division machinery and recruits the cell division scaffold protein FtsZ to initiate sporulation-specific cell division in an SsgA-dependent manner [31]. SsgB shows an extraordinary pattern of conservation. It is extremely well conserved within a single genus, with a maximum of one aa variation between all of the SsgB orthologues identified in streptomycetes, whereas between genera the conservation is often as low as 40–50%. This makes SsgB an ideal tool for molecular systematics, especially at the generic level.

In this paper, we demonstrate the usefulness of SsgA and SsgB phylogeny for the accurate taxonomic classification of morphologically complex actinomycetes, and apply this new tool to add resolution to the taxonomy of several actinomycete species. Our data suggest that *Kitasatospora* is very closely related to, but distinct from, the genus *Streptomyces*, and that *Micromonospora*, *Salinispora* and *Verrucosispora* may be congeneric.

## 3. Results and discussion

### 3.1. Distribution of SsgA-like proteins in actinomycetes

SALPs are found exclusively in sporulating actinomycetes and in other morphologically complex actinomycetes such as *Kineococcus* [16]. In addition, detailed analysis of all sequenced genomes of the non-sporulating actinomycetes *Bifidobacterium, Corynebacterium, Mycobacterium, Nocardia* and *Rhodococcus* failed to identify proteins with relevant sequence homology (i.e. higher than roughly 25% aa identity). Further studies revealed the presence of a single SALP in genera that produce one or two spores per hyphae (such as *Micromonospora, Salinispora* or *Thermobifida*) or complex morphological structures (*Kineococcus*) and multiple SALPs in actinomycetes that produce multiple spores on hypha (exemplified by *Streptomyces* and *Saccharopolyspora*) or multisporous sporangia (*Frankia*) (see electronic supplementary material, table S1). Thus, a rule of thumb has emerged, namely that a single SALP (SsgB) correlates with the presence of single spores along hyphae, two SALPs with two spores and multiple SALPs with multiple spores [16]. However, a few exceptions to this concept have now been found, namely *Catenulispora acidiphila* and *Nocardiopsis alba*, which contain a single SALP but form spore chains, and some species of *Micromonospora*, which only have SsgB but produce sporangia. So far, investigations on the function of the SALPs have focused on *Streptomyces*, and more molecular and cell biological research is required to better understand the precise function of the SALPs in additional genera such as those mentioned earlier. A phylogenetic tree of actinomycetes is presented in the electronic supplementary material, figure S1.

Interestingly, some proteins have been identified that contain a C-terminal SALP domain. In *S. griseus*, in addition to the canonical SsgABDEG, three SALP-domain-containing proteins were identified (Sgr_128 and the identical proteins Sgr_41t and Sgr_7098t) that are around 650 aa long; the first around 520 aa lack a recognizable protein domain. A 487 aa SALP-domain-containing protein (SBD_2172) was identified in *Streptomyces bottropensis* ATCC 25435^T^, which appears to be a distant homologue of the long SALPs from *S. griseus*, showing 35% aa identity in its N-terminal 90 residues to Sgr_128. The sequence of plasmid PSED02 from *Pseudonocardia dioxanivorans* CB1190 [32] revealed a gene encoding an SALP (Psed_7011) that is translationally fused to a so-called *wbl* gene (Pset_7010), for a WhiB-like protein. Homology of this protein is highest to WhiB itself (69% aa identity). This provides evidence for a functional relationship between SALPs and WhiB-like proteins, which are both developmental proteins.

The sporulation activator protein SsgA was previously considered unique to streptomycetes, where it activates the localization of SsgB to initiate sporulation-specific cell division [31]. In fact, five of the SALPs found in *S. coelicolor*, namely SsgA, SsgB, SsgD, SsgE and SsgG, have orthologues in all or almost all streptomycetes [23], although *ssgG* is missing in *Streptomyces avermitilis* MA-4680^T^, *Streptomyces* sp. e14 and *Streptomyces griseoflavus* Tü4000. Only SsgB and the related SsgG are generally found in other actinomycetes [16,28]. Some *Streptomyces* genomes encode a rather large number of SALPs, e.g. eight SALPs are encoded by the genomes of *Streptomyces viridochromogenes* Tue57 and *Streptomyces turgidiscabies* Car8, nine for *Streptomyces sviceus* ATCC 29083, 10 for *Streptomyces davawensis* JCM 4913, and a remarkable 14 for *Streptomyces hygroscopicus* subsp. *jinggangensis* 5008.

The precise translational start sites of *ssgA* and *ssgB* are still subject to debate, which is relevant to this work in terms of the subjects for phylogenetic analysis. Our recent mutational analysis (N.M. & G.P.v.W. 2013, unpublished data) revealed that most likely two of the three possible AUG translational start sites for *ssgA* are used *in vivo*, corresponding to nucleotide (nt) positions 4 319 474 and 4 319 504 on the genome of *S. coelicolor* A3(2) (further referred to as *S. coelicolor*). The translational start site annotated in the genome database (nt position 4 319 501) is almost certainly incorrect, as shown by *in vivo* mutation data and by the fact that in several streptomycetes it is an ATC codon, which cannot function as a translational start codon. In line with many genome-sequence annotations, we use the shorter *ssgA* gene product for our phylogenetic analysis (i.e. corresponding to nt position 4 319 504 in *S. coelicolor*). The *ssgB* gene has two alternative translational start sites, which correspond to nt positions 1 650 311 and 1 650 377 in the *S. coelicolor* genome. Because a transcriptional start site was identified downstream of the upstream-located alternative start codon [33], we will refer to the second (downstream) alternative start codon as the translational start site, and hence use the shorter SsgB protein for our analysis. However, it should be noted that the 22 triplets between the two alternative start codons are completely conserved even at the nt level between all streptomycetes.

### 3.2. SsgB as a novel and reliable phylogenetic marker for sporulating actinomycetes

SsgB is most likely the ancestral SALP, with only SsgB orthologues occurring in all morphologically complex actinomycetes [15,31,34]. SsgB orthologues are extremely well conserved in streptomycetes, and are typically identical except for residue 128 (Gln, Thr or in rare cases Lys; see electronic supplementary material, figure S2). Exceptions are *Streptomyces pristinaespiralis* ATCC 25486*, Streptomyces rimosus* subsp. *rimosus* ATCC 10970^T^ and *Streptomyces acidiscabies* 84–104, which all contain an additional S137N mutation at the C-terminal residue, and *S. venezuelae* ATCC 10712^T^, which has a unique but conservative E136D substitution. The evolutionary pressure for the conservation of the aa sequence is even more apparent by the relatively high nucleotide divergence, with 25–30 silent mutations, which are almost exclusively found in the third (wobble) position of the codons. Analysis of the *d*_N_/*d*_S_ ratio of *ssgB* orthologues across the actinomycetes indicates that weak purifying selection (*d*_N_/*d*_S_ ratio < 1) is acting as a functional constraint across the gene family; however, phylum level analysis of *d*_N_/*d*_S_ is inaccurate owing to the relatively high sequence divergence at the nucleotide level in these genes.

**Figure 1.**
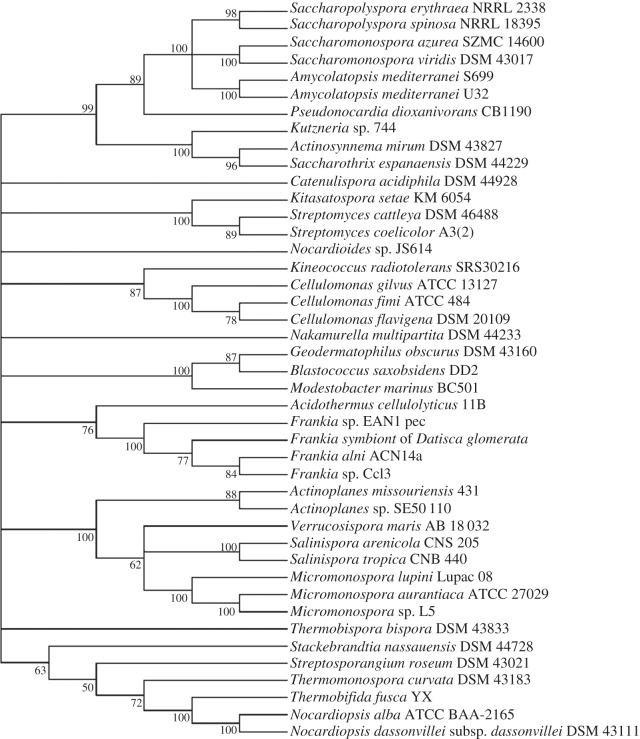
Maximum-likelihood tree based on the alignment of the 16S rRNA genes of morphologically complex actinomycetes. For input sequences and their accession numbers, see the electronic supplementary material, data file S1.

**Figure 2.**
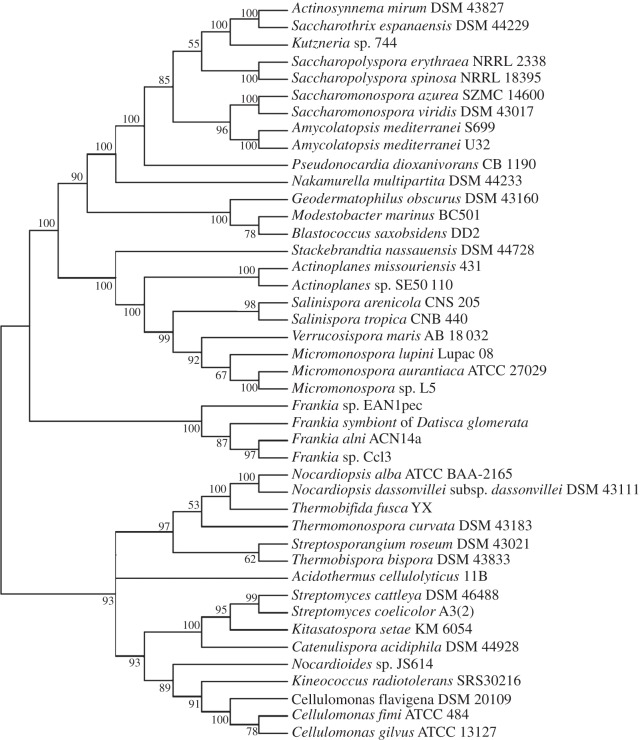
Maximum-likelihood tree based on the alignment of RpoB proteins from a range of morphologically complex actinomycetes. For input sequences and their accession numbers, see the electronic supplementary material, data file S2.

**Figure 3.**
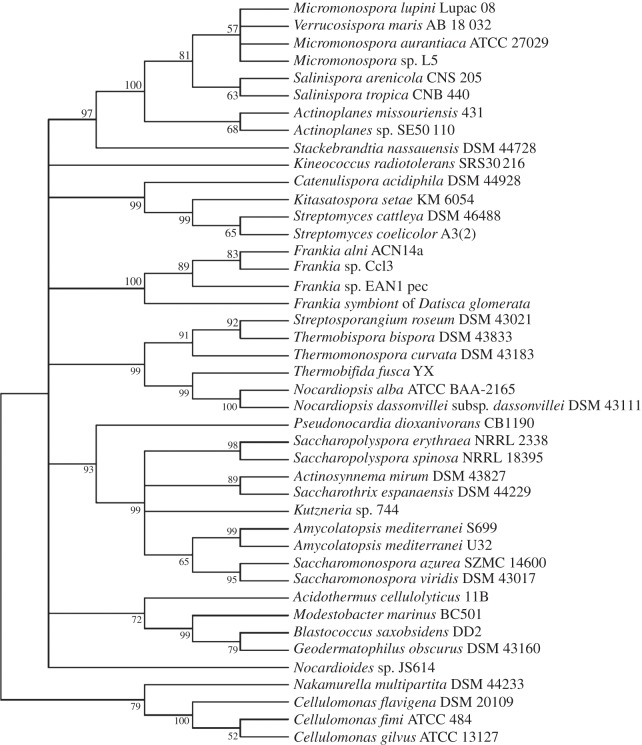
Maximum-likelihood tree based on the alignment of SsgB proteins from a range of morphologically complex actinomycetes. For input sequences and their accession numbers, see the electronic supplementary material, data file S3.

Comparison of maximum-likelihood trees of two standard taxonomic indicators, namely 16S rRNA (figure 1) and RpoB (the β-subunit of RNA polymerase [35]; figure 2), with that of SsgB (figure 3) indicates that the clades consistently group together (terminal branches) within the accepted taxonomic framework [11] as operational taxonomic units (OTUs). The congruence analysis using CONCATERPILLAR [36] revealed a phylogenetic congruence between RpoB and SsgB protein sequences (*p* = 0.2771); however, 16S rRNA nucleotide sequences were topologically incongruent with them (*p* = 0.0015). Interestingly, the groupings of the OTUs are consistent between all the trees, yet the branches indicate the overall phylogenetic history of the genes is likely to be different. The reasons for this can be attributed to gene duplication and gene loss, and to lateral gene transfer, where genes are exchanged between lineages [37,38]. Indeed, expansion of developmental gene families in actinomycetes through duplication has been studied previously [39].

### 3.3. Taxonomic status of the genera *Micromonospora*, *Salinispora* and *Verrucosispora*

It is also interesting that the SsgB proteins from the genera *Actinoplanes*, *Micromonospora*, *Salinispora* and *Verrucosispora* are nearly identical, a result that underlines the close relationship found between these members of the family Micromonosporaceae [40,41]. The next nearest relative is *Stackebrandtia nassauensis* DSM 44728^T^, with only around 65% aa identity to SsgB from Micromonosporaceae. We did not observe notable differences in the phylogenetic analysis of the Micromonosporaceae with or without the sequences from *Stack. nassauensis* (not shown). The low homology of SsgB is very interesting, because the genus *Stackebrandtia* belongs to the order of Glycomycetales, which is loosely associated with the order Micromonosporales based on 16S rRNA gene sequence data [11]. Such a high divergence of SsgB orthologues between relatively closely related genera allows rapid discrimination between morphologically close actinomycetes. This deduction is strongly supported by the analysis of concatenated sequences of 35 broadly distributed proteins as the type strain of *Stack. nassauensis* formed a clade with *Micromonospora aurantiaca* ATCC 27029^T^ and a representative of the genus *Salinispora*; this taxon was supported by a 100% bootstrap value [42].

The SsgB proteins from *M. aurantiaca* ATCC 27029^T^ and *Micromonospora* strain L5 are identical, but two aa changes are found in *Micromonospora* strain ATCC 39149. Interestingly, *Micromonospora* and *Verrucosispora* species have identical SsgB proteins, whereas 25 polymorphic nucleotides exist between the genes (table 1), similar to the differences found between SsgB orthologues from different *Streptomyces* species (maximum one aa change, and 25–30 polymorphic nucleotides). The SsgB sequences suggest that *Micromonospora* and *Verrucosispora* strains may belong to the same genus. *Salinispora tropica* SsgB has only one aa variation compared with the SsgB from *Salinispora arenicola, Micromonospora* and *Verrucosispora* species, but at the nt level the divergence between *S. arenicola* and *S. tropica* is much lower than that between *S. tropica* and the other genera, indicating that *Salinispora* species diverged from *Micromonospora* and *Verrucosispora* and may form a separate clade. The three genera can also be distinguished from one another based on comparisons of fatty acid, menaquinone and sugar profiles [43,44] and *Salinispora* from the other two by its requirement for seawater for growth [40,41]. Finally, members of the genus *Polymorphospora* are strongly related to *Micromonospora* [45], and it would be very interesting to see how closely it relates to the other Micromonosporaceae, and in particular to the genus *Micromonospora*, in terms of the SsgB sequence homologies and their implications for phylogeny.

**Table 1.** Homology between SsgB orthologues from *Micromonospora, Salinispora* and *Verrucosispora* species. Percentage of SsgB aa identity is presented, and in parentheses are the total number of aa and nt changes, respectively.

species^b^	*M.* sp. ATCC 39149	*M.* sp. L5	*M. aurantiaca*	*V. maris*	*S. arenicola*	*S. tropica*	*Actinoplanes* sp. SE50–110
*Micromonospora* sp. ATCC39149	X	98.6 (2/25)	98.6 (2/24)	98.6 (2/24)	97.9 (3/36)	97.9 (3/37)	95.8 (4/71)
*Micromonospora sp.* L5		X	*100* (*0/1*)^a^	*100* (*0/25*)^a^	98.6 (2/39)	99.3 (1/37)	97.2 (4/72)
*Micromonospora aurantiaca* ATCC 27029^T^			X	*100* (*0/25*)^a^	98.6 (2/37)	99.3 (1/36)	97.2 (4/71)
*Verrucosispora maris* AB-18–032^T^				X	98.6 (2/44)	99.3 (1/42)	97.2 (4/75)
*Salinispora arenicola* CNS205^T^					X	*99.3* (*1/14*)^a^	95.8 (4/72)
*Salinispora tropica* CNB440^T^						X	96.5 (4/73)
*Actinoplanes* sp. SE50–110							X

^a^Italicized values suggest that organisms belong to the same genus.

^b^For input sequences and their accession numbers, see the electronic supplementary material, data file S5.

### 3.4. Taxonomic status of the genus *Kitasatospora*

The difficulty of accurately classifying closed related actinomycetes at the generic level is exemplified by the genus *Kitasatospora*, which was first proposed by Omura *et al*. [46], subsequently reduced to a synonym of the genus *Streptomyces* [47] and then re-established as a separate genus [48]. The status of the genus *Kitasatospora* has still to be resolved [12], as exemplified by the fact that while members of the two genera form sister clades when using conserved *rpoB* gene sequences, *Kitasatospora* species were assigned to a large, statistically unsupported clade in the *Streptomyces* 16S rRNA gene tree [13]. Indeed, Labeda and co-workers considered that *Kitasatospora* might only be seen as taxonomically valid if the genus *Streptomyces* proved to be polyphyletic.

To resolve this intriguing taxonomic dilemma, we compared the SsgB orthologue (KSE_14600) identified in the genome of *Kitasatospora setae* KM-6054^T^ [49] with that of streptomycetes. This identified three to four aa changes relative to the *Streptomyces* SsgB orthologues (see electronic supplementary material, figure S1), and some 50 nt changes relative to the *ssgB* DNA consensus sequence (see electronic supplementary material, figure S4). This divergence is certainly significant considering that only a single aa substitution was found in all SsgB orthologues from streptomycetes, but it does not provide conclusive evidence that *Kitasatospora* should retain its status as a separate genus. However, further analysis of the genome of *K. setae* revealed no fewer than 12 genes encoding SALPs; these include orthologues of *ssgA* (KSE_39770) and *ssgG* (KSE_28490), both with strong gene synteny with the respective orthologues of *S. coelicolor*, but *ssgD* and *ssgE*, which are found in all streptomycetes, may be absent. It is particularly interesting that KSE_39770 shares 52–57% end-to-end aa identity with SsgAs from streptomycetes (table 2). This is significantly lower than the sequence homology between *Streptomyces* SsgA orthologues—which typically share 75–90% aa identity, and never lower than 64% (table 2)—but it is high enough to suggest that they may be functional homologues [16]. Gene synteny evidence (see electronic supplementary material, figure S5) shows that it is a true *ssgA* orthologue, as the flanking genes correspond well to those surrounding *ssgA* in *S. coelicolor*, with the upstream gene (KSE_39760) encoding an orthologue of SsgR (58% aa identity), the transcriptional activator of *ssgA* in *S. coelicolor* [50]. Gene rearrangements around *ssgRA* (SCO3925-3926) resulted in movement of SCO3922–3924 from upstream of *ssgR* to downstream of *ssgA*. KSE_39750, which lies immediately downstream of *ssgR* in *K. setae*, is an orthologue of SCO3918. Analysis of other *Kitasatospora* species in our collection revealed that not all *Kitasatospora* species contain an *ssgA* and/or *ssgR* orthologue, suggesting that *ssgA* is perhaps becoming obsolete in this genus (G.G. & G.P.v.W. 2013, unpublished data). Strikingly, like some *Kitasatospora* species, the streptomycete *S. cattleya* lacks an *ssgA* gene. It is yet unclear how these species sporulate without *ssgA*, in other words how these species compensate for its absence, and what the precise implications are from the perspective of taxonomy. These issues are currently under investigation in our laboratory.

**Table 2.** Protein sequence homology (% aa identity/similarity) between SsgA orthologues. Horizontal axis presents accession numbers (in genome database nomenclature), and the vertical axis presents the corresponding species. Note that all strains except *Kitasatospora* are *Streptomyces* species. For input sequences and their accession numbers, see the electronic supplementary material, data file S4.

	SAV_4267	SCLAV_2865	SCO3926	SGR_3655	SSPG_03726	STRS4_05858	SCAB46311	SVEN_3705	SSDG_00559	KSE_3977
*S. avermitilis*	X	64/75	77/86	74/84	77/86	71/82	88/91	76/85	70/83	54/66
*S. clavuligerus*		X	68/78	68/79	68/78	66/76	70/80	70/82	69/79	53/69
*S. coelicolor*			X	77/86	100/100	78/85	87/91	80/88	73/85	57/70
*S. griseus*				X	77/86	69/82	78/87	86/90	80/87	52/66
*S. lividans* TK24					X	78/85	87/91	80/88	73/85	57/70
*S.* sp. S4						X	74/83	78/83	65/80	52/65
*S. scabies*							X	78/85	75/85	53/66
*S. venezuelae*								X	83/88	53/72
*S. pristinaespiralis*									X	56/72
*Kitasatospora setae*										X

The three aa changes in the SsgB orthologues, coupled with the *rpoB* and 16S rRNA data, indicate that the genera *Kitasatospora* and *Streptomyces* are closely related, but distinct genera. The case for considering them as sister taxa is supported by the unique presence of *ssgR* and *ssgA* orthologues—which have not yet been found outside streptomycetes.

### 3.5. Classification of other actinomycetes

High conservation within specific actinomycete genera is also observed for the SsgB orthologues in the plant symbiont *Frankia* (see electronic supplementary material, figure S6). The SsgB orthologues from *Frankia alni* ACN14a, *Frankia* sp*.* EAN1pec, *Frankia* sp*.* EUN1f and *Frankia* sp*.* CN3 show one or two mismatches to the consensus sequence, *Frankia* sp*.* CcI3 and QA3 have four permutations and the symbiont of the Durango root *Datisca glomerata* has seven. Considering the relatively high divergence of the latter, it would be of great interest to determine how closely related this *Datisca* symbiont is to well-studied members of the genus *Frankia*. It is apparent from figure 1 that the SsgB proteins of the representatives of the genera *Acidothermus, Blastococcus, Geodermatophilus* and *Nakamurella* are related both to one another and to the *Frankia* strains, a result in line with 16S rRNA sequence data [11], but not with a phylogenetic tree based on concatenated sequences of conserved proteins [51].

The SsgB orthologue of *Streptomyces* species AA4 is remote to that of streptomycetes (49% aa identity over a stretch of 122 residues), and is identical to that of *Amycolatopsis decaplanina* DSM 44594^T^; this organism has recently been reclassified as an *Amycolatopsis* species based on other criteria [52], further supporting the taxonomic validity of SsgB as a marker.

### 3.6. Correlation between *Streptomyces* liquid-culture morphology and the SsgA protein sequences

**Figure 4.**
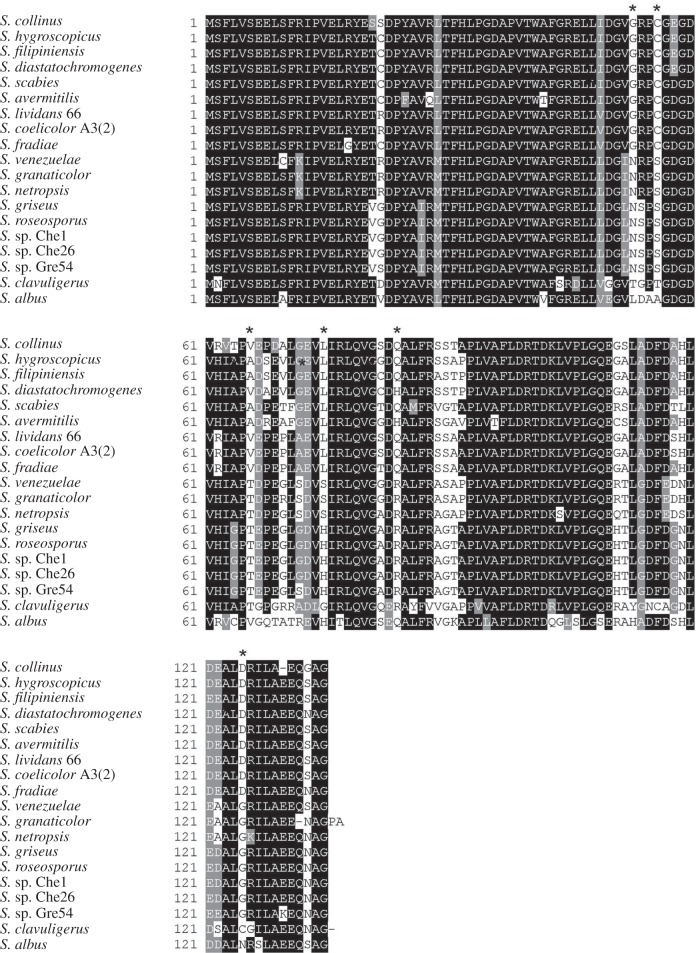
Alignment of SsgA orthologues. Only those SsgA protein sequences have been used as input that are derived from species with known phenotype in submerged cultures. For shading, at least 60% of the aligned proteins should share the same or similar aa residues. Identical residues shaded black, similar residues shaded grey. Residues highlighted with an asterisk above the alignment, are conserved within—but different between—the ‘LSp’ and ‘NLSp’ branches in figure 5 and function as identifiers for the ability of a certain *Streptomyces* species to sporulate in submerged culture. Sequences were labelled by their strain of origin, for sequence labels see §4.2. For input sequences and their accession numbers, see the electronic supplementary material, data file S4.

SsgA proteins from different streptomycetes generally share between 75% and 90% end-to-end sequence identity, with few differences between the N-termini, and regions with higher variability in the core (approx. residues 53–92 of *S. coelicolor* SsgA) and the C-termini (approx. beyond residue 110) of the proteins (figure 4). SsgA from *Streptomyces clavuligerus* ATCC 27064^T^ is the most distinct of all sequenced orthologues, with a sequence identity to other orthologues varying from 63% (compared with *Streptomyces collinus* and *Streptomyces ramocissimus* SsgA) to 73% (compared with *S. venezuelae* SsgA). SsgA proteins from *S. coelicolor* and *Streptomyces lividans* TK24 are identical, whereas their genes contain a single nucleotide difference (His42 encoded by CAT in *S. coelicolor* and by CAC in *S. lividans*). More notably, the predicted SsgA orthologues from *S. griseus* and *S. roseosporus* are also identical, whereas 17 ‘silent’ nucleotide differences occur between their respective DNA sequences, suggesting evolutionary pressure to maintain the aa sequence.

Streptomycetes can be divided morphologically in terms of their liquid-culture morphology into species that produce clumps or mycelial mats, and those that are able to form submerged spores [34]. Several *Streptomyces* species form spores in submerged cultures, including *S. granaticolor, S. griseus*, *S. roseosporus* and *S. venezuelae* [17–19]. The latter category can be subdivided into streptomycetes that only sporulate in minimal medium and typically after nutritional shift-down, with *S. griseus* as a well-known example [19], and those that always produce submerged spores, including in a rich medium, represented by among others *S. venezuelae* [18]. In a recent survey of species in our own strain collection, we discovered many others, including the putatively novel *Streptomyces* spp. Che1, Che26, Gre 19 and Gre54 studied here; these results indicate that submerged sporulation is much more common than previously thought.

**Figure 5.**
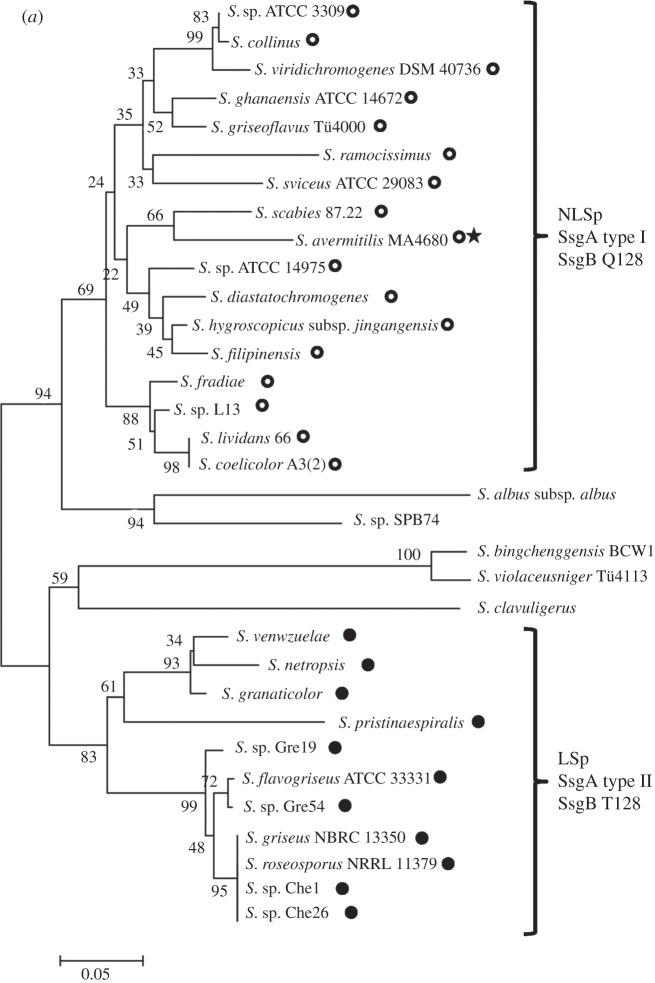

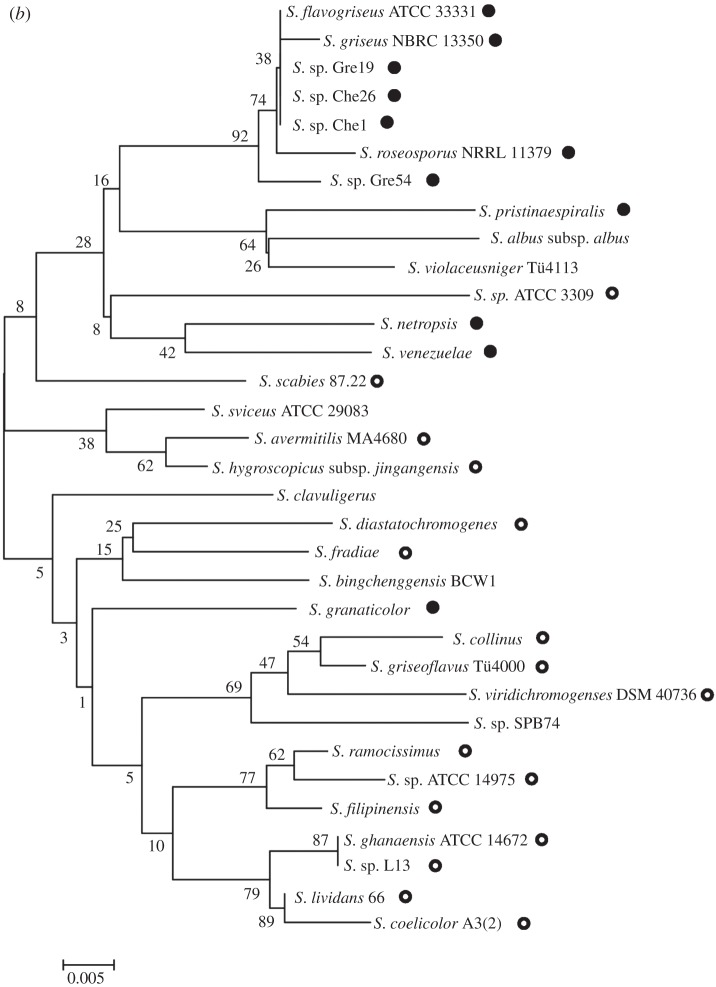
Phylogenetic tree of SsgA protein and 16S rRNA sequences in streptomycetes. Phylogenetic trees are shown for SsgA (*a*) and 16S rRNA (*b*) from 33 *Streptomyces* species (see §3). Two major branches of SsgA proteins are indicated, namely SsgA orthologues (called type I) from strains that produce typical mycelial clumps and do not produce submerged spores (NLSp branch; indicated with open circles), and SsgA orthologues (called type II) from strains that can produce spores in liquid-culture (LSp branch; closed circles). As an exception, NLSp species *S. avermitilis* (indicated with a star in figure 5*a*) carries SsgB variant T128. 16S rRNA-based classification incorrectly positions *S. granaticolor* in the NLSp branch and *Streptomyces* species ATCC 3309 in the LSp branch, while the separation between the two subclasses is also far less obvious as highlighted among others by the unresolved position of *S. scabies* in the 16S rRNA tree. For sequence labels, see §4.2. For input sequences and their accession numbers, see the electronic supplementary material, data file S4.

Interestingly, in the phylogenetic tree, SsgA proteins from strains that produce typical mycelial clumps but fail to produce submerged spores cluster together in a branch, designated NLSp (figure 5*a*). In a second branch, designated LSp, only SsgA proteins are represented that were derived from strains that can sporulate in submerged culture (figure 5*a*). The SsgA lineages are designated type I and type II, correlating with NLSp and LSp phenotypes, respectively. *Streptomyces albus* and *S. clavuligerus* produce large, open mycelial structures but do not form submerged spores. Phylogenetic analysis indicates that these species do not belong to either of the two branches, and several clear differences between their primary sequences and those from the other orthologues are apparent (figure 5*a*).

These results led us to wonder whether strains that sporulate in submerged cultures are evolutionarily more strongly related to one another than to those that only sporulate on surface-grown cultures. To analyse this, we performed a comparison of the 16S rRNA sequences of 33 *Streptomyces* species. In the 16S rRNA phylogenetic tree, similar branches as seen for SsgA proteins are less obvious (figure 5*b*). For example, *S. granaticolor*, which sporulates profusely in submerged culture and should in that sense be close to *Streptomyces netropsis* and *S. venezuelae* [18,53], is classified among the NLSp species based on its 16S rRNA sequence, whereas conversely, ATCC3309 (which fails to sporulate in submerged culture) is classified among the LSp species according to the 16S rRNA sequence. These data reveal that there is complete correspondence between the SsgA protein sequence and the ability of streptomycetes to sporulate in submerged culture, though this is not the case for the 16S rRNA tree. In other words, changes in the SsgA aa sequence provide very good linkage to distinct morphological characteristics of streptomycetes, rather than highlighting only the evolutionary divergence. Closer analysis identified significant differences in the primary sequence of SsgA orthologues from the LSp or the NLSp branches, respectively. Six residues are particularly noteworthy: Gly53, Cys56, Ala/Val66, Leu75, Gln/His84 and Asp125 residues occur exclusively in orthologues from the NLSp branch, whereas orthologues from the LSp branch contain Asn53, Ser56, His/Ser75, Arg84 and Gly125 residues in the corresponding positions (figures 4 and 5*a*). Interestingly, none of these six residues was identified as essential for SsgA function in a previous mutational study, where *ssgA* null mutants were complemented with random mutant *ssgA* variants [54]. This strongly suggests that these amino acids provide additional functionality to SsgA, which correlates with submerged sporulation.

Submerged sporulation has generally been considered as an exception rather than a common trait among streptomycetes. However, our analysis of some 50 taxonomically diverse *Streptomyces* species show that more than half produced submerged spores in minimal medium, and many also in a rich medium. As shown earlier, this ability to form submerged spores can be predicted by reading six letters in the aa code of the SsgA primary sequence. This implies that the biological activity of the type I and type II SsgA proteins may be different. Indeed, we previously showed that overexpression of the type I SsgA from *S. coelicolor* and *S. lividans* does not have a major effect on liquid culture morphology of *S. coelicolor*, whereas the overexpression of a type II SsgA from *S. griseus* results in hyphal fragmentation and even induced the formation of spore chains in submerged cultures of *S. coelicolor* [22,26,55]. Replacement of the chromosomal copy of *ssgA* of *S. coelicolor* by that of *S. griseus* did not confer the ability to produce submerged spores, but resulted in less densely packed clumps in submerged culture (N.M. & G.P.v.W. 2013, unpublished data). Thus, the effect of SsgA on hyphal morphology appears to be dictated by its aa sequence.

Another striking link between SALP protein sequences and liquid culture morphology is seen in the permutations that occur in residue 128 of SsgB. Much to our surprise, we found that all *Streptomyces* species of the LSp type have an SsgB orthologue with a Thr128, whereas those of the NLSp type have an SsgB with Gln128. The only exception is the NLSp *S. avermitilis* MA-4680^T^, which also carries a T128. This coincides with the absence of *ssgG*, a direct functional homologue of *ssgB*, in this species. The exciting implication of a direct relationship between specific aa residues of SsgA and SsgB on the one hand and submerged sporulation on the other hand offers new insights into the function of SsgA and SsgB in the control of *Streptomyces* development and to the development of strains for industrial processes. This phenomenon is currently under investigation.

### 3.7. Concluding remarks

It is becoming increasingly clear that prokaryotic systematists need to re-evaluate their practices in the light of the plethora of information derived from sequencing whole genomes and conserved proteins [10,51,56,57]. This study is a tangible expression of this need as it has been shown that SsgA and SsgB proteins present in morphologically complex actinomycetes are the source of high-quality molecular data that can be used to resolve relationships between diverse genera classified in the class Actinobacteria [2]. Our work highlights the importance of combining molecular systematic and traditional taxonomic approaches, in accordance with the work of others [58,59], to identify chemotaxonomic and morphological markers as an excellent evidence-based way of distinguishing between closely related genera of actinomycetes, as exemplified by the distinction between *Kitasatospora* and *Streptomyces*. Additional comparative studies based on representatives of genera classified in taxa such as the orders Frankiales and Micromonosporales [11,51] can be expected to help resolve longstanding taxonomic enigmas.

It is also apparent from this and earlier studies that SALPs are crucial for developmental cell division in actinomycetes; thus, whereas one SALP (SsgB) suffices to form a single spore, multiple (three or more) SALPs are required to coordinate the production of longer spore chains or sporangia. It would be interesting to determine whether multiple SALPs can trigger the production of multisporous structures in actinomycetes that normally produce single spores or fail to form spores at all. Conversely, combinations of *ssg* mutants in, for example, *S. coelicolor* may result in streptomycetes forming single spores or short spore chains. This interesting concept should be tested and if verified would provide very strong experimental proof for the phylogenetic evidence.

With the rapidly emerging genome sequences, new SALP sequences are highlighted weekly. We expect that analysis of the sequences of the SALPs will facilitate the accurate taxonomic classification of sporulating actinomycetes.

## 4. Material and methods

### 4.1. Strains and medium

*Streptomyces* strains Che1, Che26, Gre19 and Gre54 were isolated from French forest soils in the Loire department (close to the castles of Cheverny and Chambord) and L13 from soil of the Canary Island Lanzarote. For initial isolation of actinomycetes, soil suspensions were spread onto humic acid agar plates [60] supplemented with the antifungal agent nystatin (50 µg ml^−1^) and the antibacterial agent nalidixic acid (10 µg ml^−1^). All of these organisms sporulated abundantly on routine medium such as SFM, R2YE or MM (minimal medium) agar plates supplemented with glycerol (1% w/v) as the sole carbon source [61]. Streptomycetes were grown under routine conditions as described by Kieser *et al*. [61]. To analyse the ability of streptomycetes to sporulate in submerged culture, they were grown in TSBS (tryptic soy broth with sucrose) or modified MM supplemented with mannitol [62], and TSBS-grown cultures were subjected to nutritional shift-down, which induces submerged sporulation [19]. For this, cultures were spun down, washed in MM and transferred to MM with glycerol or mannitol (1% w/v) as the sole carbon source. Submerged spores were harvested by filtration to remove mycelial biomass, checked by their ability to germinate, and plated next to the original strains to confirm their identity. Microscopy was performed as described previously [63]. Cultures were checked at regular intervals by phase contrast microscopy using a Zeiss Standard 25 microscope and colony morphology was studied using a Zeiss Lumar V-12 stereo microscope.

### 4.2. Sequence alignment and phylogenomic analysis

All predicted sequences (aa and nt) were downloaded from the NCBI database (www.ncbi.nlm.nih.gov) on 13 February 2013. Nucleotide and protein sequences in FASTA format and their accession numbers are presented in the electronic supplementary material, data files S1–S5. Homologues were identified by BLASTP against the non-redundant protein sequence database using SsgB from *S. coelicolor* A3(2) (accession number NP_625820) as a query and by searching the Gene database on NCBI for proteins with an SsgA domain (Pfam 04686) in each organism of interest.

Alignment of SsgB, 16S rRNA and RpoB sequences was generated using MUSCLE [64] with default options, followed by manual editing. The neighbour-joining trees [65] were generated with default parameters settings as implemented in MEGA v. 4.0 [66]. The maximum-likelihood trees were made using the best fit models predicted by MEGA: 16S rRNA tree using GTR + G + I, RpoB using rtREV + G + I + F and the SsgB tree following the WAG + G + I model, all with four discrete gamma categories and a complete deletion of missing nucleotides/amino acids. Tree reliability was estimated by bootstrapping with 500 replicates. The groupings that are supported by poor bootstrap values are not reliable. Therefore, we have collapsed the internal branches with a bootstrap value of less than 50% to generate consensus trees using MEGA [66] to emphasize the reliable branching patterns.

Topological congruence between 16S rRNA nucleotide sequences and RpoB and SsgB protein sequences was tested using CONCATERPILLAR [36]. The distances at synonymous (*d*_S_) and non-synonymous sites (*d*_N_) were calculated with Jukes and Cantor correction for the nucleotide sequence alignment of *ssgB* gene using DnaSP [67] to calculate *d*_N_/*d*_S_ ratios. Alignments shown were visualized with BoxShade v. 3.21 (http://www.ch.embnet.org/software/BOX_form.html) or EbioX tools (http://www.ebioinformatics.org/ebiox/).

## Supplementary Material

Supplemental Material

## Data accessibility

All accession numbers of the DNA and protein sequences have been made available in the electronic supplementary material.
