# Supplementary material for: A novel taxonomic marker that discriminates between morphologically complex actinomycetes
Source: Open Biol. 2013 Oct;3(10):130073. doi: 10.1098/rsob.130073 (PMC3814722; doi:10.1098/rsob.130073)
Supplement: Supplemental Material [file rsob130073supp1.pdf]

SUPPLEMENTARY MATERIAL

belonging to the manuscript

A novel taxonomic marker that discriminates between morphologically complex actinomycetes

by

Geneviève Girard[1], Bjørn A. Traag[2], Vartul Sangal[3], Nadine Mascini[1], Paul A. Hoskisson[3], Michael Goodfellow[4] and Gilles P. van Wezel[1, #]

[1]Molecular Biotechnology, Institute of Biology, Leiden University, PO Box 9505, 2300RA, Leiden, The Netherlands; [2] Department of Molecular and Cellular Biology, Harvard University, 16 Divinity Avenue, Cambridge, MA 02138; [3] Strathclyde Institute of Pharmacy and Biomedical Sciences, University of Strathclyde, Glasgow, UK; [4] School of Biology, Ridley Building, Newcastle University, NE1 7RU Newcastle upon Tyne, UK.

CONTENT:

Supplemental Table S1

Supplemental Figures S1-S6

Supplemental Data files 1-5

Table S1. Number of SALPs and developmental characteristics of completely sequenced actinomycetes. For a list of all protein sequences and their accession numbers see Supplemental Data file 5.

Species	NCBI reference sequence	genome size (Mbp)	Nr of SALPs	cell morphology, development
Streptomyces avermitilis	NC_003155	9	6	aerial mycelium, long spore chains
Streptomyces coelicolor	NC_003888	8.7	7	
Streptomyces griseus	NC_010572	8.5	5	
Streptomyces scabiei	NC_013929	10.1	7	
Streptomyces clavuligerus	NZ_CM001015	9.1	6	
Kitasatospora setae	NC_016109	8.8	12	
Amycolatopsis mediterranei U32	NC_014318	10.2	5	short spore chains
Frankia alni	NC_008278	7.5	5	multisporous sporangium
Frankia sp. CcI3	NC_007777	5.4	3	
Frankia sp. EAN1pec	NC_009921	9	3	
Frankia sp. QA3	NZ_CM001489	7.6	4	
Frankia symbiont of Datisca glomerata	NC_015656	5.3	2	sporangium
Actinosynnema mirum	NC_013093	8.3	2	dome like structures with Synnemata
Pseudonocardia dioxanivorans	NC_015312	7.4	2	budding and fragmenting aerial mycelium
Saccharomonospora viridis	NC_013159	4.3	2	single spores in aerial mycelium
Saccharothrix espanaensis	NC_019673	9.4	2	nocardioform mycelia and rod-shaped spores
Saccharopolyspora erythraea	NC_009142	8.2	3	spore chains with spiny spores
Saccharopolyspora spinosa NRRL 18395	NZ_AEYC00000000	8.5	8	
Streptosporangium roseum	NC_013595	10.4	2	sporangia on aerial hyphae
Acidothermus cellulolyticus	NC_008578	2.4	1	long slender filaments, no spores
Actinoplanes missouriensis	NC_017093	8.8	1	sporangium in mycelium
Blastococcus saxobsidens DD2	NC_016943	4.9	1	aggregated cocci or rods forming multiseptate filaments
Catenulispora acidiphila	NC_013131	10.5	1	chains of cylindrical arthrospores
Cellulomonas fimi	NC_015514	4.3	1	irregular rod shape, no spores
Geodermatophilus obscurus	NC_013757	5.3	1	aggregated cocci or rods forming multiseptate filaments
Kineococcus radiotolerans	NC_009664	5.0	1	multicellular structures
Micromonospora	NC_014391	7.0	1	single spores from vegetative

aurantiaca				mycelium
Modestobacter marinus	NC_017955	5.6	1	aggregated cocci or rods forming multiseptate filaments
Nakamurella multipartita	NC_013235	6.1	1	cocci in small irregular clusters; no spores
Nocardioides sp. JS614	NC_08699	5.3	1	rods or cocci, no spores
Nocardiopsis alba	NC_018524	5.9	1	hyphae that fragment into smooth rod-shaped spores
Salinispora arenicola	NC_009953	5.8	1	single spores from vegetative mycelium
Salinispora tropica	NC_009380	5.2	1	
Stackebrandtia nassauensis	NC_013947	6.8	1	fragmenting aerial hyphae
Thermobifida fusca	NC_007333	3.6	1	single spores borne on branched sporophores
Thermobispora bispora	NC_014165	4.2	1	aerial mycelia branch monopodally and bear longitudinal pairs of spores
Thermomonospora curvata	NC_013510	5.7	1	single, warty spores on sporophores (on vegetative and aerial hyphae)
Verrucosispora maris	NC_015434	6.7	1	single spores from mycelium

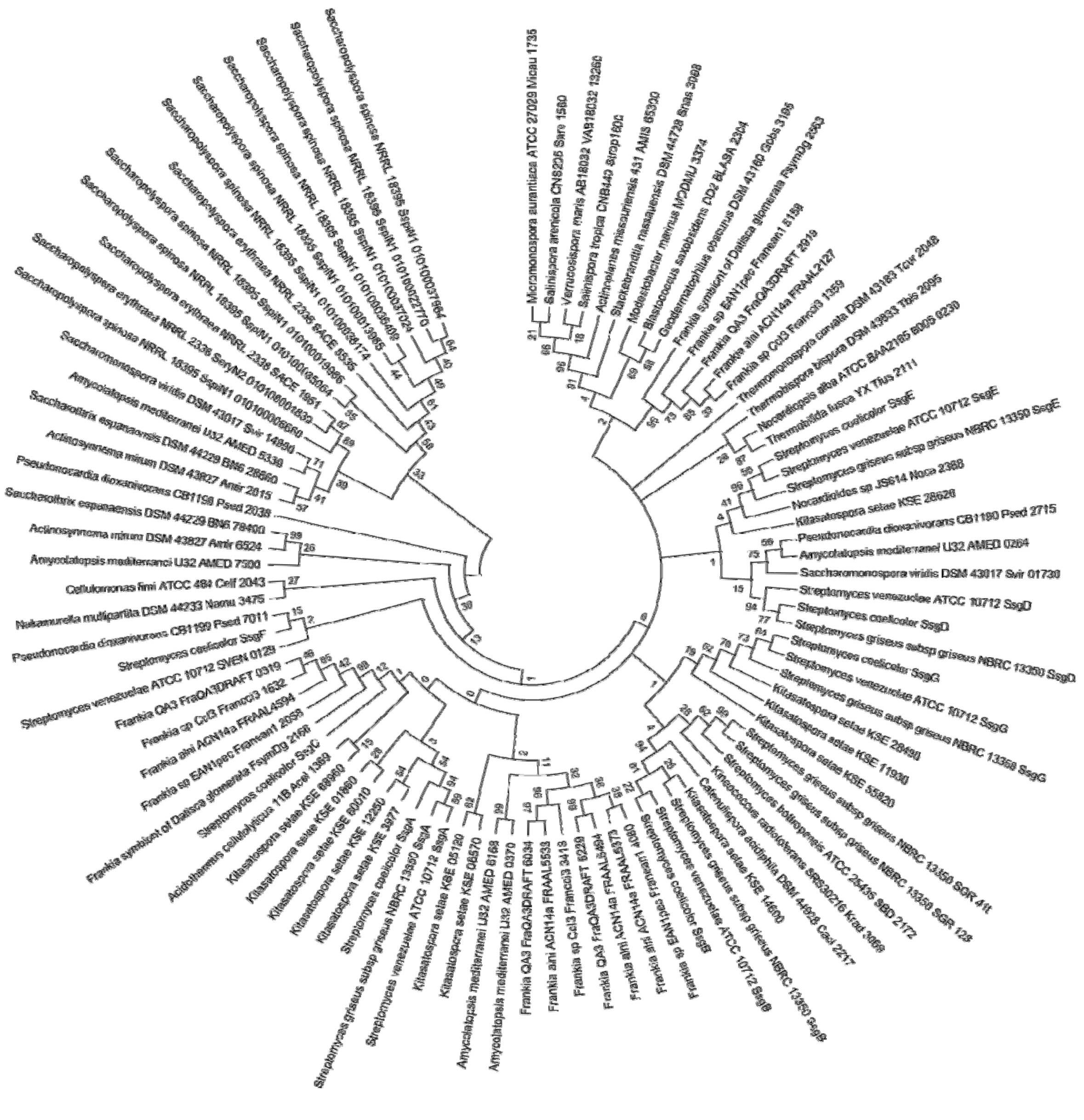

Figure S1. Maximum-likelihood tree based on the alignment of SALPs proteins from a range of morphologically complex actinomycetes. For input sequences and their accession numbers see Supplemental Data file 5.

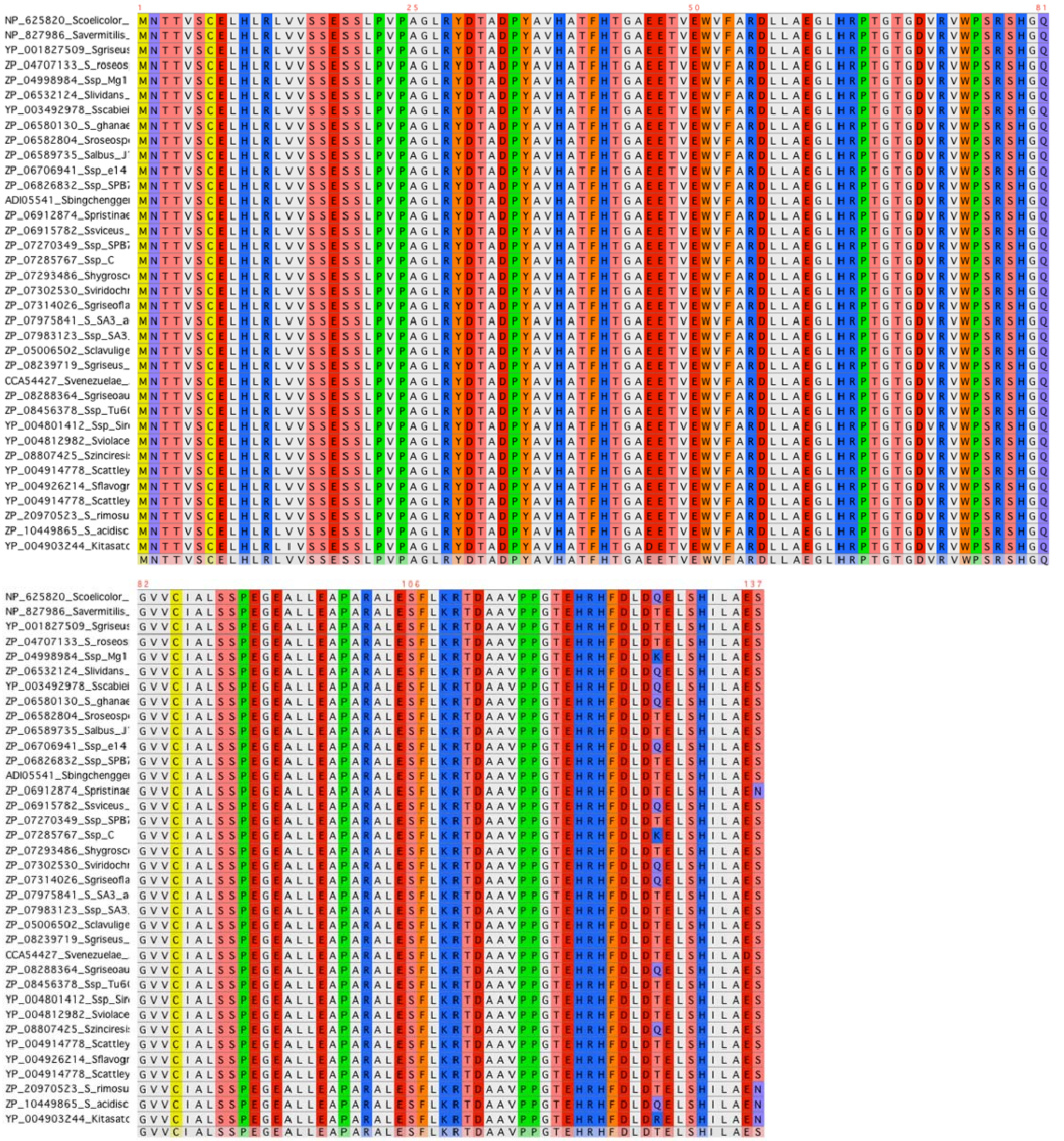

Figure S2. Alignment of SsgB orthologues. All species are *Streptomyces* sp., except *Kitasatospora setae* at the bottom. For input sequences and their accession numbers see Supplemental Data file 5.

```
Salinispora_arenicola             1 MSVIRPTTVEVETSLRLVAPDATALPVRASLRYDPADPYAVHVLFHAESAGGEAVSWSFA
Salinispora_tropica               1 MSVIRPTTVEVETSLRLVAPDATALPVRASLRYDPADPYAVHVLFHAESAGGEAVSWSFA
Micromonospora_ATCC39149          1 MSVIRPTTVEVETSLRLVAPDATALPVRASLRYDPADPYAVHVLFHAESAGGEAVSWSFA
Micromonospora_sp.L5              1 MSVIRPTTVEVETSLRLVAPDATALPVRASLRYDPADPYAVHVLFHAESAGGEAVSWSFA
Verrucosispora_maris              1 MSVIRPTTVEVETSLRLVAPDATALPVRASLRYDPADPYAVHVLFHAESAGGEAVSWSFA
Micromonospora_aurantiaca_ATCC    1 MSVIRPTTVEVETSLRLVAPDATALPVRASLRYDPADPYAVHVLFHAESAGGEAVSWSFA
Actinoplanes_sp.SE50/110          1 MSTIRPTTVEVETSLRLVAPDATALPVRASLRYDPSDPYAVHVLFHAESAGGEAVSWSFA

Salinispora_arenicola            61 RELLVTGLDEPAGIGDVRVWPWATPRGDFVALALSSPDGNALFEVPRSVLVRFLRRTYVV
Salinispora_tropica              61 RELLVTGLDEPAGIGDVRVWPWATPRGDFVALALSSPDGNALFEVPRSVLVRFLRRTYVV
Micromonospora_ATCC39149         61 RELLVTGLDEPAGIGDVRVWPWATPRGDFVALALSSPDGNALFEVPRSVLVRFLRRTYVV
Micromonospora_sp.L5             61 RELLVTGLDEPAGIGDVRVWPWATPRGDFVALALSSPDGNALFEVPRSVLVRFLRRTYVV
Verrucosispora_maris             61 RELLVTGLDEPAGIGDVRVWPWATPRGDFVALALSSPDGNALFEVPRSVLVRFLRRTYVV
Micromonospora_aurantiaca_ATCC   61 RELLVTGLDEPAGIGDVRVWPWATPRGDFVALALSSPDGNALFEVPRSVLVRFLRRTYVV
Actinoplanes_sp.SE50/110         61 RELLVTGLDEPAGIGDVRVWPWATPRGDFVALALSSPDGNALFEVPRSVLVRFLRRTYVV

Salinispora_arenicola           121 VARGREAEHLDVDTAVSRLLAGR
Salinispora_tropica             121 VARGREAEHLDVDTAVNRLLAGR
Micromonospora_ATCC39149        121 VPRGREADHLDVDTAVTRLLAGR
Micromonospora_sp.L5            121 VPRGREAEHLDVDTAVNRLLAGR
Verrucosispora_maris            121 VPRGREAEHLDVDTAVNRLLAGR
Micromonospora_aurantiaca_ATCC  121 VPRGREAEHLDVDTAVNRLLAGR
Actinoplanes_sp.SE50/110        121 VPRGRESEHLDVDAAVNRLLAGR
```

Figure S3. Alignment of SsgB proteins from *Micromonospora*, *Salinispora* and *Verrucosispora*. Species from which SsgB orthologues originate are indicated on the left. For shading, at least 50% of the aligned proteins should share the same or similar aa residues. Identical residues shaded black, similar residues shaded grey. For input sequences and their accession numbers see Supplemental Data file 1.

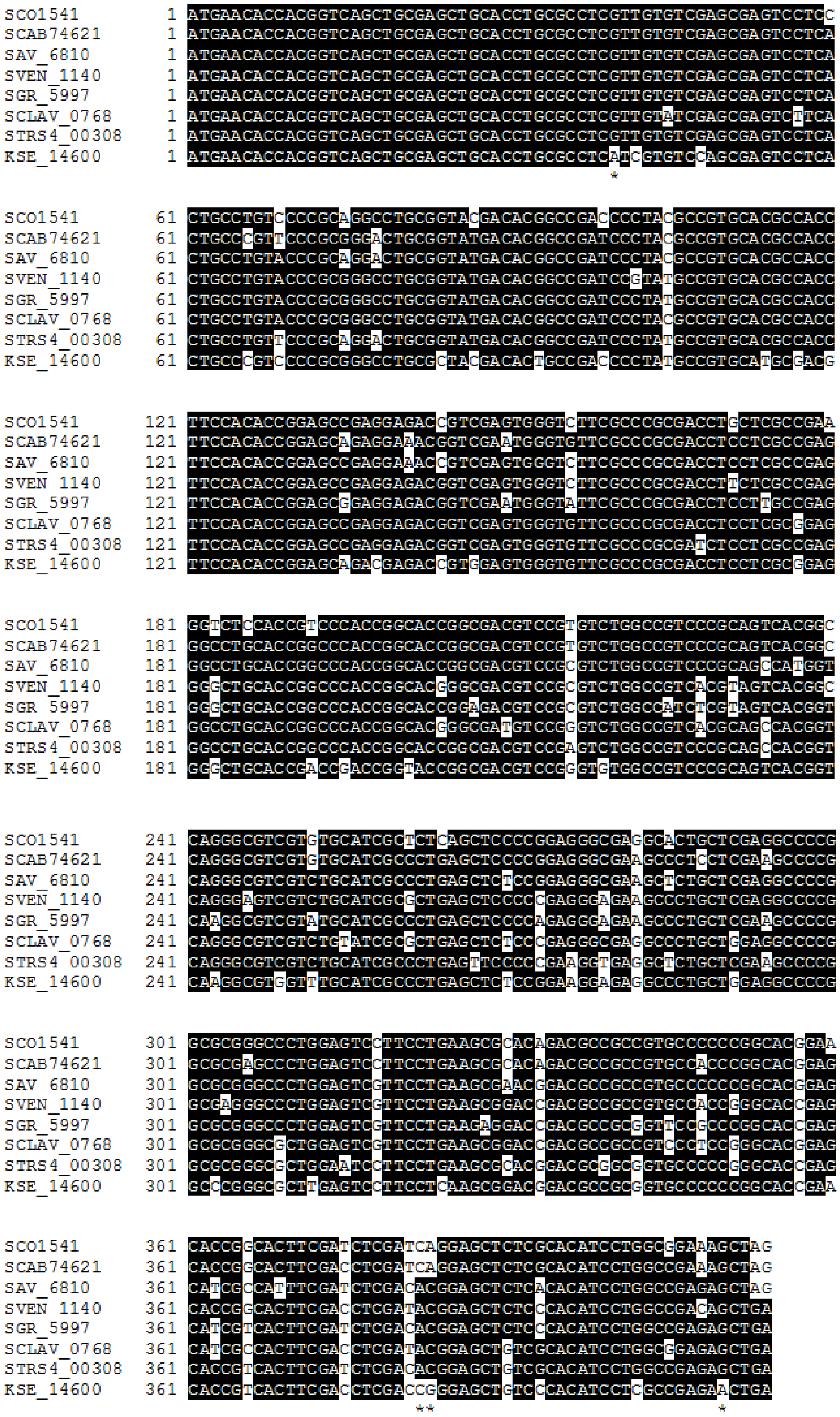

Figure S4. Alignment of *ssgB* genes from *Streptomyces* and *Kitasatospora*. The locus of each gene within corresponding genomes is indicated. The *ssgB* genes are from the genome sequences of *Streptomyces coelicolor* (SCO1541), *S. scabies* (Scab74621), *S. avermitilis* (SAV_6810), *S. venezuelae* (SVEN1140), *S. griseus* (SGR_5997) *S. clavuligerus*

(SCLAV_0768), *S. sp. S4* (STRS4_00308) and *Kitasatospora setae* (KSE_14600).

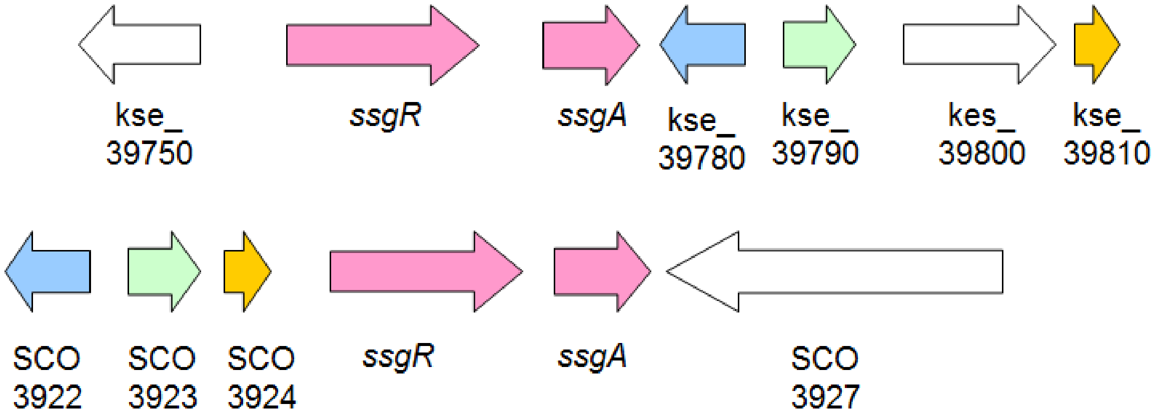

Figure S5. Gene synteny around *ssgA* from *Streptomyces coelicolor* and *Kitasatospora setae*. Direct orthologues are presented in the same colours (white genes are not related).

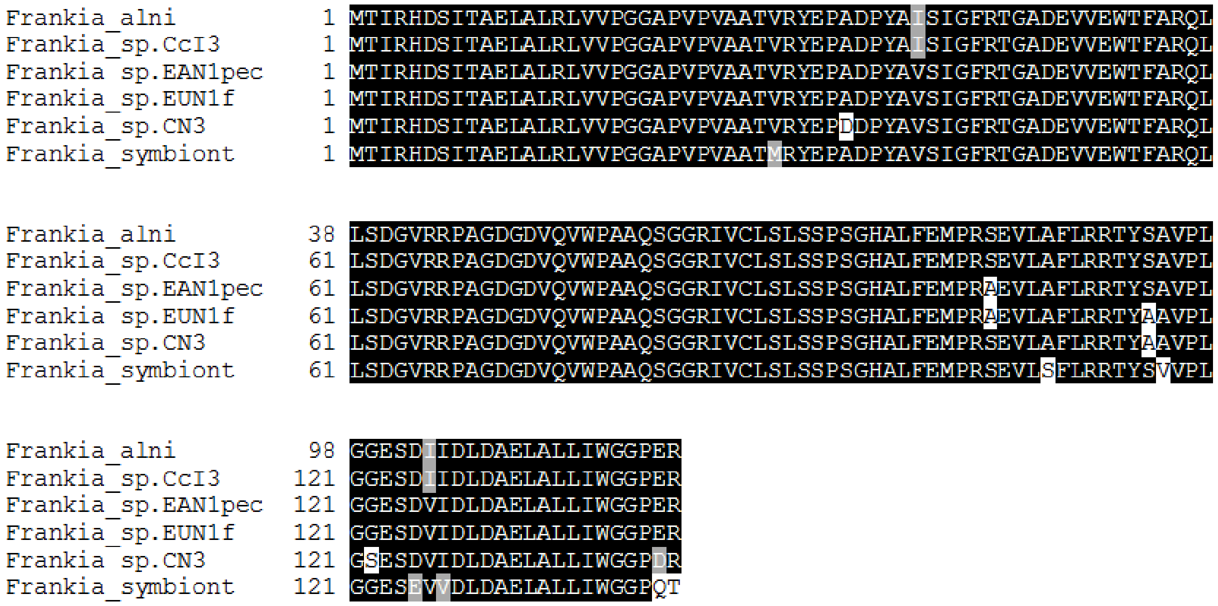

Figure S6. Alignment of SsgB orthologues from different *Frankia* species. Species from which SsgB orthologues originate are indicated on the left. For shading, at least 50% of the aligned proteins should share the same or similar aa residues. Identical residues shaded black, similar residues shaded grey. For input sequences and their accession numbers see Supplemental Data file 1.

Supplemental Data file 1. 16S rRNA sequences from actinomycetes used as input for Figure 1.

```
>gb|CP000481.1|:1397655-1399163 Acidothermus cellulolyticus 11B,
complete genome
GTTTGATCCTGGCTCAGGACGAACGCTGGCGGCGTGCTTAACACATGCAAGTCGAGCGGAAAGGCCCTTC
GGGGTACTCGAGCGGCGAACGGGTGAGTAACACGTGGGCAACCTACCCCCAGCACCGGGATAACCCCGGG
AAACCGGGGCTAATACCGGATATGCACCTCGGGTGGCATCACCTGAGGTGGAAAGCTTTTGCGGCTGGGG
ATGGGCCCGCGGCCTATCAGCTTGTTGGTGGGGTAACGGCCTACCAAGGCGACGACGGGTAGCCGGCCTG
AGAGGGCGACCGGCCACACTGGGACTGAGACACGGCCCAGACTCCTACGGGAGGCAGCAGTGGGGAATAT
TGCGCAATGGGCGAAAGCCTGACGCAGCGACGCCGCGTGGGGGAAGAAGGCCTTCGGGTTGTAAACCCCT
TTCAGCAGGAACGAAGCGAAAGTGACGGTACCTGCAGAAGAAGCACCGGCTAACTACGTGCCAGCAGCCG
CGGTAATACGTAGGGTGCAAGCGTTGTCCGGATTTACTGGGCGTAAAGAGCTCGTAGGCGGTCTGTCGCG
TCGGATGTGAAAACCCAGGGCTCAACCCTGGGCCTGCATTCGATACGGGCAGACTAGAGTGCGGCAGGGG
AGACTGGAATTCCTGGTGTAGCGGTGAAATGCGCAGATATCAGGAGGAACACCGGTGGCGAAGGCGGGTC
TCTGGGCCGCAACTGACGCTGAGGAGCGAAAGCGTGGGGAGCGAACAGGATTAGATACCCTGGTAGTCCA
CGCTGTAAACGTTGGGCGCTAGGTGTGGGGGACTTTCCACGTCCTCCGTGCCGCAGCTAACGCATTAAGC
GCCCCGCCTGGGGAGTACGGCCGCAAGGCTAAAACTCAAAGGAATTGACGGGGGCCCGCACAAGCGGCGG
AGCATGTGGCTTAATTCGATGCAACGCGAAGAACCTTACCTGGGCTTGACATGCAGGGAAATCCGGCAGA
GATGCCGGGTCCGCAAGGGCCCTGCACAGGTGGTGCATGGCTGTCGTCAGCTCGTGTCGTGAGATGTTGG
GTTAAGTCCCGCAACGAGCGCAACCCTCGTCCCATGTTGCCAGCGGGTAATGCCGGGGACTCATGGGAGA
CTGCCGGGGTCAACTCGGAGGAAGGTGGGGATGACGTCAAGTCATCATGCCCCTTACGTCCAGGGCTGCA
CACATGCTACAATGGCCGGTACAAAGGGCTGCGAAACCGTGAGGTGGAGCGAATCCCAAAAAGCCGGTCT
CAGTTCGGATCGGGGTCTGCAACTCGACCCCGTGAAGTCGGAGTCGCTAGTAATCGCGGATCAGCAATGC
CGCGGTGAATACGTTCCCGGGCCTTGTACACACCGCCCGTCACGTCACGAAAGTCGGTAACACCCGAAGC
CGGTGGCCCAACCCGCAAGGGAGGGAGCCGTCGAAGGTGGGACCGGCGATTGGGACGAAGTCGTAACAAG
GTAGCCGTACCGGAAGGTGTGGCTGGATCACCTCCTTTC
>gi|381368402:4544018-4545534 Actinoplanes missouriensis 431 DNA,
complete genome
TGTTGGAGAGTTTGATCCTGGCTCAGGACGAACGCTGGCGGCGTGCTTAACACATGCAAGTCGAGCGGAA
AGGCCCTTCGGGGTACTCGAGCGGCGAACGGGTGAGTAACACGTGAGTAACCTGCCCCGAACTTTGGGAT
AACCCTCGGAAACGGGGGCTAATACCGAATACGACACGCCTTCGCATGGGGTGCGTGTGGAAAGTTTTTC
GGTTCGGGATGGACTCGCGGCCTATCAGCTTGTTGGTGGGGTAATGGCCTACCAAGGCGACGACGGGTAG
CCGGCCTGAGAGGGCGACCGGCCACACTGGGACTGAGACACGGCCCAGACTCCTACGGGAGGCAGCAGTG
GGGAATATTGCACAATGGGCGGAAGCCTGATGCAGCGACGCCGCGTGAGGGATGACGGCCTTCGGGTTGT
AAACCTCTTTCAGCAGGGACGAAGCGCAAGTGACGGTACCTGCAGAAGAAGCGCCGGCCAACTACGTGCC
AGCAGCCGCGGTAAGACGTAGGGCGCGAGCGTTGTCCGGATTTATTGGGCGTAAAGAGCTCGTAGGCGGC
TTGTCGCGTCGAATGTGAAATCTCGGGGCTCAACTCCGAGCTTGCATTCGATACGGGCAGGCTAGAGTTC
GGTAGGGGAGACTGGAATTCCTGGTGTAGCGGTGAAATGCGCAGATATCAGGAGGAACACCGGTGGCGAA
GGCGGGTCTCTGGGCCGATACTGACGCTGAGGAGCGAAAGCGTGGGGAGCGAACAGGATTAGATACCCTG
GTAGTCCACGCTGTAAACGTTGGGCGCTAGGTGTGGGGACCCTCTCCGGGTTTCTGCGCCGCAGCTAACG
CATTAAGCGCCCCGCCTGGGGAGTACGGCCGCAAGGCTAAAACTCAAAGGAATTGACGGGGGCCCGCACA
AGCGGCGGAGCATGCGGATTAATTCGATGCAACGCGAAGAACCTTACCTGGGTTTGACATCGCCGGAAAA
CTCGCAGAGATGTGGGGTCCTTCGGGGCCGGTGACAGGTGGTGCATGGCTGTCGTCAGCTCGTGTCGTGA
GATGTTGGGTTAAGTCCCGCAACGAGCGCAACCCTCGTCCCATGTTGCCAGCATTCAGTTGGGGACTCAT
GGGAGACTGCCGGGGTCAACTCGGAGGAAGGTGGGGATGACGTCAAGTCATCATGCCCCTTATGTCCAGG
GCTTCACGCATGCTACAATGGCCGGTACAAAGGGCTGCGATACCGTGAGGTGGAGCGAATCCCAAAAAGC
CGGTCTCAGTTCGGATCGGGGTCTGCAACTCGACCCCGTGAAGTCGGAGTCGCTAGTAATCGCAGATCAG
CAACGCTGCGGTGAATACGTTCCCGGGCCTTGTACACACCGCCCGTCACGTCACGAAAGTCGGCAACACC
CGAAGCCGGTGGCCTAACCCCTTGTGGGAGGGAGCCGTCGAAGGTGGGGCTGGCGATTGGGACGAAGTCG
TAACAAGGTAGCCGTACCGGAAGGTGCGGCTGGATCACCTCCTTTCT
>gb|CP003170.1|:569506-571009 Actinoplanes sp. SE50/110, complete
genome
AGAGTTTGATCCTGGCTCAGGACGAACGCTGGCGGCGTGCTTAACACATGCAAGTCGAGCGGAAAGGCCC
TTCGGGGTACTCGAGCGGCGAACGGGTGAGTAACACGTGAGTAACCTGCCCCAGACTTTGGGATAACCCT
CGGAAACGGGGGCTAATACCGAATATGACCTAGCTTCGCATGGAGCGTGGTGGAAAGTTTTTCGGTTTGG
```

GATGGACTCGCGGCCTATCAGCTTGTTGGTGGGGTAATGGCCTACCAAGGCGACGACGGGTAGCCGGCCT
GAGAGGGCGACCGGCCACACTGGGACTGAGACACGGCCCAGACTCCTACGGGAGGCAGCAGTGGGGAATA
TTGCACAATGGGCGGAAGCCTGATGCAGCGACGCCGCGTGAGGGATGACGGCCTTCGGGTTGTAAACCTC
TTTCAGCAGGGACGAAGCGCAAGTGACGGTACCTGCAGAAGAAGCGCCGGCCAACTACGTGCCAGCAGCC
GCGGTAAGACGTAGGGCGCGAGCGTTGTCCGGATTTATTGGGCGTAAAGAGCTCGTAGGCGGCTTGTCGC
GTCGAATGTGAAATCCCGAGGCTCAACTTCGGGCTTGCATTCGATACGGGCAGGCTAGAGTTCGGTAGGG
GAGACTGGAATTCCTGGTGTAGCGGTGAAATGCGCAGATATCAGGAGGAACACCGGTGGCGAAGGCGGGT
CTCTGGGCCGATACTGACGCTGAGGAGCGAAAGCGTGGGGAGCGAACAGGATTAGATACCCTGGTAGTCC
ACGCTGTAAACGTTGGGCGCTAGGTGTGGGGGGCCTCTCCGGTTTCCTGCGCCGCAGCTAACGCATTAAG
CGCCCCGCCTGGGGAGTACGGCCGCAAGGCTAAAACTCAAAGGAATTGACGGGGGCCCGCACAAGCGGCG
GAGCATGCGGATTAATTCGATGCAACGCGAAGAACCTTACCTGGGTTTGACATCGCCGGAAATCTCGCAG
AGATGCGGGGTCCTTCGGGGCCGGTGACAGGTGGTGCATGGCTGTCGTCAGCTCGTGTCGTGAGATGTTG
GGTTAAGTCCCGCAACGAGCGCAACCCTCGTTCGATGTTGCCAGCGCGTAATGGCGGGGACTCATCGGAG
ACTGCCGGGGTCAACTCGGAGGAAGGTGGGGATGACGTCAAGTCATCATGCCCCTTATGTCCAGGGCTTC
ACGCATGCTACAATGGCCGGTACAAAGGGCTGCGATACCGTAAGGTGGAGCGAATCCCAAAAAGCCGGTC
TCAGTTCGGATCGGGGTCTGCAACTCGACCCCGTGAAGTCGGAGTCGCTAGTAATCGCAGATCAGCAACG
CTGCGGTGAATACGTTCCCGGGCCTTGTACACACCGCCCGTCACGTCACGAAAGTCGGCAACACCCGAAG
CCGGTGGCCTAACCCGTAAGGGAGGGAGCCGTCGAAGGTGGGGCTGGCGATTGGGACGAAGTCGTAACAA
GGTAGCCGTACCGGAAGGTGCGGCTGGATCACCT
>gi|860736|emb|X84447.1| Actinosynnema mirum 16S rRNA gene, strain DSM 43827T
TGGCGGCGTGCTTAACACATGCAAGTCGAGCGGTAAGGCCCTTCGGGGTACACGAGCGGCGAACGGGTGA
GTAACACGTGGGTAACCTGCCCTGTACTCTGGGATAAGCCTGGGAAACTAGGTCTAATACCGGATACGAG
CCTTCCCNCATGGGGTTGGTTGGAAAGTTCCGGCGGTACAGGATGGACCCGCGGCCTATCAGCTTGTTGG
TGGGGTAATGGCCTACCAAGGCGACGACGGGTAGCCGGCCTGAGAGGGTGACCGGCCACACTGGGACTGA
GACACGGCCCAGACTCCTACGGGAGGCAGCAGTGGGGAATATTGCACAATGGGCGAAAGCCTGATGCAGC
GACGCCGCGTGAGGGACGACGGCCTTCGGGTTGTAAACCTCTTTCAGCAGGGACGAAGCGCAAGTGACGG
TAGGTGCAGAAGAAGCACCGGCTAACTACGTGCCAGCAGCCGCGGTAATACGTAGGGTGCGAGCGTTGTC
CGGAATTATTGGGCGTAAAGAGCTCGTAGGCGGTTTGTCGCGTCGGCCGTGAAAACTTGGGGCTTAACCC
CGAGCCTGCGGTCGATACGGGCAGACTTGAGTTCGGCAGGGGAGACTGGAATTCCTGGTGTAGCGGTGAA
ATGCGCAGATATCAGGAGGAACACCGGTGGCGAAGGCGGGTCTCTGGGCCGATACTGACGCTGAGGAGCG
AAAGCGTGGGGAGCGAACAGGATTAGATACCCTGGTAGTCCACGCCGTAAACGGTGGGTGCTAGGTGTGG
GGGACTTCCACGTCCTCCGTGCCGCAGCTAACGCATTAAGCACCCCGCCTGGGGAGTACGGCCGCAAGGC
TAAAACTCAAAGGAATTGACGGGGGCCCGCACAAGCGGCGGAGCATGTGGATTAATTCGATGCAACGCGA
AGAACCTTACCTGGGCTTGACATGCACTGGAAACCAGTAGAGATATTGGCCCCCTTGTGGCCGGTGTACA
GGTGGTGCATGGCTGTCGTCAGCTCGTGTCGTGAGATGTTGGGTTAAGTCCCGCAACGAGCGCAACCCTC
GTTCCATGTTGCCAGCGCGTTATGGCGGGGACTCATGGGAGACTGCCGGGGTCAACTCGGAGGAAGGTGG
GGATGACGTCAAGTCATCATGCCCCTTATGTCCAGGGCTTCACACATGCTACAATGGCCGGTACAAAGGG
CTGCTAAGCCGTGAGGTGGAGCGAATCCCATAAAGCCGGTCTCAGTTCGGATCGGGGTCTGCAACTCGAC
CCCGTGAAGTCGGAGTCGCTAGTAATCGCAGATCAGCAACGCTGCGGTGAATACGTTCCCGGGCCTTGTA
CACACCGCCCGTCACGTCACGAAAGTCGGTAACACCCGAAGCCCGTGGCCCAACCCGCAAGGGGGGGAGC
GGTCGAAGGTGGGACTGGCGATTGGGACGAAGTCGTAACAAGGTAGCCGTACCGGAAGGT
>gi|399533820:1303703-1305161 Amycolatopsis mediterranei S699 chromosome, complete genome
CTGGCTCAGGACGAACGCTGGCGGCGTGCTTAACACATGCAAGTCGAACGATGAAGCCTTCGGGTGGATT
AGTGGCGAACGGGTGAGTAACACGTGGGTAATCTGCCCTGCACTCTGGGATAAGCCTTGGAAACGGGGTC
TAATACCGGATATCACAATCTCTCGCATGGGGGGTTGTTGAAAGTTCTGGCGGTGCAGGATGAACCCGCG
GCCTATCAGCTTGTTGGTGGGGTAGTGGCCTACCAAGGCGACGACGGGTAGCCGGCCTGAGAGGGTGACC
GGCCACACTGGGACTGAGACACGGCCCAGACTCCTACGGGAGGCAGCAGTGGGGAATATTGCACAATGGG
CGCAAGCCTGATGCAGCGACGCCGCGTGAGGGATGACGGCCTTCGGGTTGTAAACCTCTTTCGCCAGGGA
CGAAGCGCAAGTGACGGTACCTGGATAAGAAGCACCGGCTAACTACGTGCCAGCAGCCGCGGTAATACGT
AGGGTGCGAGCGTTGTCCGGATTTATTGGGCGTAAAGAGCTCGTAGGCGGTTTGTCGCGTCGGCCGTGAA
ATCTCCACGCTTAACGTGGAGCGTGCGGTCGATACGGGCAGACTTGAGTTCGGTAGGGGAGACTGGAATT
CCTGGTGTAGCGGTGAAATGCGCAGATATCAGGAGGAACACCGGTGGCGAAGGCGGGTCTCTGGGCCGAT
ACTGACGCTGAGGAGCGAAAGCGTGGGGAGCGAACAGGATTAGATACCCTGGTAGTCCACGCTGTAAACG
TTGGGCGCTAGGTGTGGGCGACATCCACGTTGTCCGTGCCGTAGCTAACGCATTAAGCGCCCCGCCTGGG
GAGTACGGCCGCAAGGCTAAAACTCAAAGGAATTGACGGGGGCCCGCACAAGCGGCGGAGCATGTGGATT
AATTCGATGCAACGCGAAGAACCTTACCTGGGCTTGACATGCGCCAGACATCCTCAGAGATGGGGCTTCC

CTTGTGGTTGGTGTACAGGTGGTGCATGGCTGTCGTCAGCTCGTGTCGTGAGATGTTGGGTTAAGTCCCG
CAACGAGCGCAACCCTTATCCTACGTTGCCAGCGCGTGATGGCGGGGACTCGTGGGAGACTGCCGGGGTC
AACTCGGAGGAAGGTGGGGATGACGTCAAGTCATCATGCCCCTTATGTCCAGGGCTTCACACATGCTACA
ATGGCTGGTACAGAGGGCTGCGATACCGCGAGGTGGAGCGAATCCCTTAAAGCCGGTCTCAGTTCGGATC
GCAGTCTGCAACTCGACTGCGTGAAGTCGGAGTCGCTAGTAATCGCAGATCAGCAACGCTGCGGTGAATA
CGTTCCCGGGCCTTGTACACACCGCCCGTCACGTCATGAAAGTCGGTAACACCCGAAGCCCATGGCCCAA
CCCCGTAAGGGGAGGGAGTGGTCGAAGGTGGGACTGGCGATTGGGACGAAGTCGTAACA
>gb|CP002000.1|:2307316-2308774 Amycolatopsis mediterranei U32, complete genome
CTGGCTCAGGACGAACGCTGGCGGCGTGCTTAACACATGCAAGTCGAACGATGAAGCCTTCGGGTGGATT
AGTGGCGAACGGGTGAGTAACACGTGGGTAATCTGCCCTGCACTCTGGGATAAGCCTTGGAAACGGGGTC
TAATACCGGATATCACAATCTCTCGCATGGGGGGTTGTTGAAAGTTCTGGCGGTGCAGGATGAACCCGCG
GCCTATCAGCTTGTTGGTGGGGTAGTGGCCTACCAAGGCGACGACGGGTAGCCGGCCTGAGAGGGTGACC
GGCCACACTGGGACTGAGACACGGCCCAGACTCCTACGGGAGGCAGCAGTGGGGAATATTGCACAATGGG
CGCAAGCCTGATGCAGCGACGCCGCGTGAGGGATGACGGCCTTCGGGTTGTAAACCTCTTTCGCCAGGGA
CGAAGCGCAAGTGACGGTACCTGGATAAGAAGCACCGGCTAACTACGTGCCAGCAGCCGCGGTAATACGT
AGGGTGCGAGCGTTGTCCGGATTTATTGGGCGTAAAGAGCTCGTAGGCGGTTTGTCGCGTCGGCCGTGAA
ATCTCCACGCTTAACGTGGAGCGTGCGGTCGATACGGGCAGACTTGAGTTCGGTAGGGGAGACTGGAATT
CCTGGTGTAGCGGTGAAATGCGCAGATATCAGGAGGAACACCGGTGGCGAAGGCGGGTCTCTGGGCCGAT
ACTGACGCTGAGGAGCGAAAGCGTGGGGAGCGAACAGGATTAGATACCCTGGTAGTCCACGCTGTAAACG
TTGGGCGCTAGGTGTGGGCGACATCCACGTTGTCCGTGCCGTAGCTAACGCATTAAGCGCCCCGCCTGGG
GAGTACGGCCGCAAGGCTAAAACTCAAAGGAATTGACGGGGGCCCGCACAAGCGGCGGAGCATGTGGATT
AATTCGATGCAACGCGAAGAACCTTACCTGGGCTTGACATGCGCCAGACATCCTCAGAGATGGGGCTTCC
CTTGTGGTTGGTGTACAGGTGGTGCATGGCTGTCGTCAGCTCGTGTCGTGAGATGTTGGGTTAAGTCCCG
CAACGAGCGCAACCCTTATCCTACGTTGCCAGCGCGTGATGGCGGGGACTCGTGGGAGACTGCCGGGGTC
AACTCGGAGGAAGGTGGGGATGACGTCAAGTCATCATGCCCCTTATGTCCAGGGCTTCACACATGCTACA
ATGGCTGGTACAGAGGGCTGCGATACCGCGAGGTGGAGCGAATCCCTTAAAGCCGGTCTCAGTTCGGATC
GCAGTCTGCAACTCGACTGCGTGAAGTCGGAGTCGCTAGTAATCGCAGATCAGCAACGCTGCGGTGAATA
CGTTCCCGGGCCTTGTACACACCGCCCGTCACGTCATGAAAGTCGGTAACACCCGAAGCCCATGGCCCAA
CCTCGTAAGGGGAGGGAGTGGTCGAAGGTGGGACTGGCGATTGGGACGAAGTCGTAACA
>gi|378781357:163561-165067 Blastococcus saxobsidens DD2 complete genome
AGAGTTTGATCCTGGCTCAGGACGAACGCTGGCGGCGTGCTTAACACATGCAAGTCGAACGGTGAACTCC
GCTTGCGGGGGGATCAGTGGCGAACGGGTGAGTAACACGTGGGCAACCTGCCCCTGGCTCTGGGATAACT
CCAAGAAATTGGGGCTAATACCGGATATGACCGCTGACCGCATGGTCTGGTGGTGGAAAGATTTATCGGC
TGGGGATGGGCCCGCGGCCTATCAGCTTGTTGGTGGGGTAGTGGCCTACCAAGGCGACGACGGGTAGCCG
GCCTGAGAGGGTGACCGGCCACACTGGGACTGAGACACGGCCCAGACTCCTACGGGAGGCAGCAGTGGGG
AATATTGCGCAATGGGCGGAAGCCTGACGCAGCGACGCCGCGTGGGGGATGACGGCCTTCGGGTTGTAAA
CCTCTTTCAGCAGGGACGAAGCGAGAGTGACGGTACCTGCAGAAGAAGCACCGGCCAACTACGTGCCAGC
AGCCGCGGTAATACGTAGGGTGCAAGCGTTGTCCGGAATTATTGGGCGTAAAGAGCTCGTAGGCGGTTCG
TCGCGTCGGCTGTGAAATCCCGAGGCTCAACCTCGGGTCTGCAGTCGATACGGGCGGACTTGAGTTCGGC
AGGGGAGACTGGAATTCCTGGTGTAGCGGTGAAATGCGCAGATATCAGGAGGAACACCGGTGGCGAAGGC
GGGTCTCTGGGCCGAAACTGACGCTGAGGAGCGAAAGCGTGGGGAGCGAACAGGATTAGATACCCTGGTA
GTCCACGCCGTAAACGTTGGGCGCTAGGTGTGGGGCTCATTCCACGAGTTCCGTGCCGCAGCTAACGCAT
TAAGCGCCCCGCCTGGGGAGTACGGCCGCAAGGCTAAAACTCAAAGGAATTGACGGGGGCCCGCACAAGC
GGCGGAGCATGTTGCTTAATTCGATGCAACGCGAAGAACCTTACCTAGGCTTGACATGCACGGAAATCCT
CCAGAGATGGTGGGTCCGTAAGGGCCGTGCACAGGTGGTGCATGGTTGTCGTCAGCTCGTGTCGTGAGAT
GTTGGGTTAAGTCCCGCAACGAGCGCAACCCTCGTTCTATGTTGCCAGCACGTCATGGTGGGGACTCATA
GGAGACTGCCGGGGTCAACTCGGAGGAAGGTGGGGATGACGTCAAATCATCATGCCCCTTATGTCTAGGG
CTGCAAACATGCTACAATGGCTGGTACAAAGGGCTGCGATACCGCGAGGTGGAGCGAATCCCAAAAAGCC
GGTCTCAGTTCGGATTGGGGTCTGCAACTCGACCCCATGAAGTTGGAGTCGCTAGTAATCGCAGATCAGC
AACGCTGCGGTGAATACGTTCCCGGGCCTTGTACACACCGCCCGTCACGTCACGAAAGTCGGTAACGCCC
GAAGCCGGTGGCCCAACCCTTGTGGAGGGAGCCGTCGAAGGCGGGATCGGCGATTGGGACGAAGTCGTAA
CAAGGTAGCCGTACCGGAAGGTGCGGCTGGATCACCT
>gi|343201636|ref|NR_042362.1| Catenulispora acidiphila DSM 44928 strain : ID139908 = DSM 44928 = NRRL B-24433 16S ribosomal RNA, partial sequence
GACGAACGCTGGCGGCGTGCTTAACACATGCAAGTCGAACGGTGAACCGCTTTCGGGCGGGGATCAGTGG

CGAACGGGTGAGTAACACGTGGGTAACCTGCCCTCCACTCTGGGATAACTCCGGGAAACCGGGGCTAATA
CCGGATACGACTCGAGGCGGCATCGCCTCGGGTGGAAAGTTTTTCGGTGGAGGATGGACCCGCGGCCTAT
CAGCTTGTTGGTGGGGTAACGGCCTACCAAGGCGATGACGGGTAGCCGGCCTGAGAGGGCGACCGGCCAC
ACTGGGACTGAGACACGGCCCAGACTCCTACGGGAGGCAGCAGTGGGGAATATTGCACAATGGGCGAAAG
CCTGATGCAGCGACGCCGCGTGAGGGATGACGGCCTTCGGGTTGTAAACCTCTTTCAGCAGGGACGAAGC
GCAAGTGACTGTACCTGCAGAAGAAGCACCGGCTAACTACGTGCCAGCAGCCGCGGTAATACGTAGGGTG
CGAGCGTTGTCCGGAATTATTGGGCGTAAAGAGCTTGTAGGTGGCTCGTTGCGTCGGGAGTGAAAACCCG
CGGCTTAACCGCGGGCCTGCTTCCGATACGGGCGAGCTAGAGTTCGGCAGGGGAGACTGGAATTCCTGGT
GTAGCGGTGGAATGCGCAGATATCAGGAGGAACACCGGTGGCGAAGGCGGGTCTCTGGGCCGATACTGAC
ACTGAGAAGCGAAAGCGTGGGGAGCGAACAGGATTAGATACCCTGGTAGTCCACGCCGTAAACGTTGGGA
ACTAGGTGTGGGGTCCATTCCACGGGCTCTGTGCCGCAGCTAACGCATTAAGTTCCCCGCCTGGGGAGTA
CGGCCGCAAGGCTAAAACTCAAAGGAATTGACGGGGGCCCGCACAAGCGGCGGAGCATGTGGCTTAATTC
GATGCAACGCGAAGAACCTTACCTGGGCTTGACATGTTCGGTCTACCTGCAGAGATGTGGGGTGCTTTTG
CGCCGTTCACAGGTGGTGCATGGTTGTCGTCAGCTCGTGTCGTGAGATGTTGGGTTAAGTCCCGCAACGA
GCGCAACCCTCGTTCCATGTTGCCAGCGCGTTATGGCGGGGACTCATGGGAGACTGCCGGGGTCAACTCG
GAGGAAGGTGGGGACGACGTCAAATCATCATGCCCCTTATGTCCAGGGCTGCACACATGCTACAATGGCC
GGTACAGAGGGCTGCGATACCGCAAGGTGGAGCGAATCCCAAAAAGCCGGTCTCAGTTCGGATCGGGGTC
TGCAACTCGACCCCGTGAAGTCGGAGTCGCTAGTAATCGCAGATCAGCAACGCTGCGGTGAATACGTTCC
CGGGCCTTGTACACACCGCCCGTCACGTCACGAAAGTCGGTAACACCCGAAGCCGGTGGCCTAACCCCTT
GTGGGAGGGAGCCGTCGAAGGTGGGACCGGCGATTGGGACG
>gb|CP002666.1|:1832187-1833696 Cellulomonas fimi ATCC 484, complete genome
AGAGTTTGATTCTGGCTCAGGACGAACGCTGGCGGCGTGCTTAACACATGCAAGTCGAACGGTGATGACA
GGGCTTGCTCTGTCTGATCAGTGGCGAACGGGTGAGTAACACGTGAGCAACCTGCCCCTGACTCTGGGAT
AACGACCGGAAACGGTCGCTAATACCGGATATGAGACGTACAGGCATCTGTTGCGTCTGGAAAGTTTTTC
GGTCAGGGATGGGCTCGCGGCCTATCAGCTTGTTGGTGGGGTAACGGCCTACCAAGGCGACGACGGGTAG
CCGGCCTGAGAGGGCGACCGGCCACACTGGGACTGAGACACGGCCCAGACTCCTACGGGAGGCAGCAGTG
GGGAATATTGCACAATGGGCGAAAGCCTGATGCAGCGACGCCGCGTGAGGGATGACGGCCTTCGGGTTGT
AAACCTCTTTCAGCAGGGAAGAAGCGCAAGTGACGGTACCTGCAGAAGAAGCGCCGGCTAACTACGTGCC
AGCAGCCGCGGTAATACGTAGGGCGCAAGCGTTGTCCGGAATTATTGGGCGTAAAGAGCTCGTAGGCGGT
TTGTCGCGTCTGCTGTGAAAACCTCAGGCTCAACCTGGGGCTTGCAGTGGGTACGGGCAGACTAGAGTGC
GGTAGGGGTGACTGGAATTCCTGGTGTAGCGGTGGAATGCGCAGATATCAGGAGGAACACCGATGGCGAA
GGCAGGTCACTGGGCCGCAACTGACGCTGAGGAGCGAAAGCATGGGGAGCGAACAGGATTAGATACCCTG
GTAGTCCATGCCGTAAACGTTGGGCACTAGGTGTGGGGCTCATTCCACGAGCTCTGTGCCGCAGCAAACG
CATTAAGTGCCCCGCCTGGGGAGTACGGCCGCAAGGCTAAAACTCAAAGAAATTGACGGGGGCCCGCACA
AGCGGCGGAGCATGCGGATTAATTCGATGCAACGCGAAGAACCTTACCAAGGCTTGACATACACCGGAAA
AGTGCAGAGATGTGCTCCCCGTAAGGTCGGTGTACAGGTGGTGCATGGTTGTCGTCAGCTCGTGTCGTGA
GATGTTGGGTTAAGTCCCGCAACGAGCGCAACCCTCGTCCCATGTTGCCAGCGGGTTATGCCGGGGACTC
ATGGGAGACTGCCGGGGTCAACTCGGAGGAAGGTGGGGATGACGTCAAATCATCATGCCCCTTATGTCTT
GGGCTTCACGCATGCTACAATGGCCGGTACAAAGGGCTGCGATACCGCGAGGTGGAGCGAATCCCAAAAA
GCCGGTCTCAGTTCGGATTGGGGTCTGCAACTCGACCCCATGAAGTCGGAGTCGCTAGTAATCGCAGATC
AGCAACGCTGCGGTGAATACGTTCCCGGGCCTTGTACACACCGCCCGTCAAGTCACGAAAGTCGGTAACA
CCCGAAGCCGGTGGCCCAACCCTTGTGGGGGGAGCCGTCGAAGGTGGGACTGGCGATTGGGACTAAGTCG
TAACAAGGTAGCCGTACCGGAAGGTGCGGCTGGATCACCT
>gb|CP001964.1|:1209818-1211325 Cellulomonas flavigena DSM 20109, complete genome
AGAGTTTGATTCTGGCTCAGGACGAACGCTGGCGGCGTGCTTAACACATGCAAGTCGAACGGTGAAGGTC
AGCTTGCTGACCGGATCAGTGGCGAACGGGTGAGTAACACGTGAGCAACCTACCCTTCACTCTGGGATAA
GCCTTGGAAACGAGGTCTAATACCGGATACGAGACGCACGGGCATCTGTAGCGTCTGGAAAGATTTATCG
GTGGGGGATGGGCTCGCGGCCTATCAGCTTGTTGGTGGGGTAATGGCCTACCAAGGCGACGACGGGTAGC
CGGCCTGAGAGGGCGACCGGCCACACTGGGACTGAGACACGGCCCAGACTCCTACGGGAGGCAGCAGTGG
GGAATATTGCACAATGGGCGCAAGCCTGATGCAGCGACGCCGCGTGCGGGATGACGGCCTTCGGGTTGTA
AACCGCTTTCAGCAGGGAAGAAGCGAGAGTGACGGTACCTGCAGAAGAAGCGCCGGCTAACTACGTGCCA
GCAGCCGCGGTAATACGTAGGGCGCAAGCGTTGTCCGGAATTATTGGGCGTAAAGAGCTCGTAGGCGGTT
TGTCGCGTCTGCTGTGAAAACCTCAGGCTCAACCTGGGGCTTGCAGTGGGTACGGGCAGACTAGAGTGCG
GTAGGGGTGACTGGAATTCCTGGTGTAGCGGTGGAATGCGCAGATATCAGGAGGAACACCGATGGCGAAG
GCAGGTCACTGGGCCGCAACTGACGCTGAGGAGCGAAAGCATGGGGAGCGAACAGGATTAGATACCCTGG
TAGTCCATGCCGTAAACGTTGGGCACTAGGTGTGGGGTCCATTCCACGGATTCCGTGCCGCAGCAAACGC

ATTAAGTGCCCCGCCTGGGGAGTACGGCCGCAAGGCTAAAACTCAAAGAAATTGACGGGGGCCCGCACAA
GCGGCGGAGCATGCGGATTAATTCGATGCAACGCGAAGAACCTTACCAAGGCTTGACATACACCGGAAAC
GTGCAGAGATGTGCGCCCCGCAAGGTCGGTGTACAGGTGGTGCATGGTTGTCGTCAGCTCGTGTCGTGAG
ATGTTGGGTTAAGTCCCGCAACGAGCGCAACCCTCGTCCCATGTTGCCAGCGGGTTATGCCGGGGACTCA
TGGGAGACTGCCGGGGTCAACTCGGAGGAAGGTGGGGATGACGTCAAATCATCATGCCCCTTATGTCTTG
GGCTTCACGCATGCTACAATGGCCGGTACAAAGGGCTGCGATACCGCGAGGTGGAGCGAATCCCAAAAAG
CCGGTCTCAGTTCGGATTGGGGTCTGCAACTCGACCCCATGAAGTCGGAGTCGCTAGTAATCGCAGATCA
GCAACGCTGCGGTGAATACGTTCCCGGGCCTTGTACACACCGCCCGTCAAGTCATGAAAGTCGGTAACAC
CCGAAGCCGATGGCCCAACCGCAAGGGGGGAGTCGTCGAAGGTGGGACTGGCGATTAGGACTAAGTCGTA
ACAAGGTAGCCGTACCGGAAGGTGCGGCTGGATCACCT
>gb|CP002665.1|:2110404-2111909 Cellulomonas gilvus ATCC 13127,
complete genome
AGAGTTTGATTCTGGCTCAGGACGAACGCTGGCGGCGTGCTTAACACATGCAAGTCGAACGGTGAAGACC
AGCTTGCTGGTTGGATCAGTGGCGAACGGGTGAGTAACACGTGAGCAACCTGCCCCTGACTCTGGGATAA
GCCTTGGAAACGAGGTCTAATACCGGATATGACACTGTCACGCATGTGGTGGTGTGGAAAGAATTTCGGT
TGGGGATGGGCTCGCGGCCTATCAGCTTGTTGGTGGGGTGATGGCCTACCAAGGCGACGACGGGTAGCCG
GCCTGAGAGGGCGACCGGCCACACTGGGACTGAGACACGGCCCAGACTCCTACGGGAGGCAGCAGTGGGG
AATATTGCACAATGGGCGCAAGCCTGATGCAGCGACGCCGCGTGAGGGATGACGGCCTTCGGGTTGTAAA
CCTCTTTCAGCAGGGAAGAAGCGAAAGTGACGGTACCTGCAGAAGAAGCGCCGGCTAACTACGTGCCAGC
AGCCGCGGTGATACGTAGGGCGCAAGCGTTGTCCGGAATTATTGGGCGTAAAGAGCTCGTAGGCGGTCAG
TCGCGTCTGCTGTGAAAACTCGAGGCTCAACCTCGGGCTTGCAGTGGGTACGGGCTGACTAGAGTGCGGT
AGGGGTGACCGGAATTCCTGGTGTAGCGGTGGAATGCGCAGATATCAGGAGGAACACCGATGGCGAAGGC
AGGTCACTGGGCCGCAACTGACGCTGAGGAGCGAAAGCATGGGGAGCGAACAGGATTAGATACCCTGGTA
GTCCATGCCGTAAACGTTGGGCACTAGGTGTGGGGTCCATTCCACGGATTCCGTGCCGCAGCTAACGCAT
TAAGTGCCCCGCCTGGGGAGTACGGCCGCAAGGCTAAAACTCAAAGAAATTGACGGGGGCCCGCACAAGC
GGCGGAGCATGCGGATTAATTCGATGCAACGCGAAGAACCTTACCAAGGCTTGACATACACCGGAAAAGT
GCAGAGATGTGCTCCCCGTAAGGTCGGTGTACAGGTGGTGCATGGTTGTCGTCAGCTCGTGTCGTGAGAT
GTTGGGTTAAGTCCCGCAACGAGCGCAACCCTCGTCCCATGTTGCCAGCGGGTTATGCCGGGGACTCATG
GGAGACTGCCGGGGTCAACTCGGAGGAAGGTGGGGATGACGTCAAATCATCATGCCCCTTATGTCTTGGG
CTTCACGCATGCTACAATGGCCGGTACAAAGGGCTGCGATACCGTAAGGTGGAGCGAATCCCAAAAAGCC
GGTCTCAGTTCGGATTGGGGTCTGCAACTCGACCCCATGAAGTCGGAGTCGCTAGTAATCGCAGATCAGC
AACGCTGCGGTGAATACGTTCCCGGGCCTTGTACACACCGCCCGTCAAGTCACGAAAGTCGGTAACACCC
GAAGCCGGTGGCCCAACCTTTTGGGGGGAGCCGTCGAAGGTGGGACTGGCGATTGGGACTAAGTCGTAAC
AAGGTAGCCGTACCGGAAGGTGCGGCTGGATCACCT
>gi|111147037:5633232-5634741 Frankia alni str. ACN14A chromosome,
complete sequence
TTGATGGAGAGTTTGATCCTGGCTCAGGACGAACGCTGGCGGCGTGCTTAACACATGCAAGTCGAGCGAG
GGGCTTCGGCTCCTAGCGGCGAACGGGTGAGTAACACGTGGGCAACCTGCCCCGAGCTCTGGAATAACCT
CGGGAAACCGGGGCTAATGCCGGATATGACGCTACCGGGCATCTGGTGGTGTGGAAAGATTTATCGGCTC
GGGATGGGCCCGCGGCCTATCAGCTTGTTGGTGGGGTGATGGCCTACCAAGGCGACGACGGGTAGCCGGC
CTGAGAGGGCGACCGGCCACACTGGGACTGAGACACGGCCCAGACTCCTACGGGAGGCAGCAGTGGGGAA
TATTGCGCAATGGGCGAAAGCCTGACGCAGCGACGCCGCGTGGGGGATGACGGCCTTCGGGTTGTAAACC
TCTTTCAGCAGGGACGAAGCGCAAGTGACGGTACCTGCAGAAGAAGCACCGGCCAACTACGTGCCAGCAG
CCGCGGTAATACGTAGGGTGCAAGCGTTGTCCGGAATTATTGGGCGTAAAGAGCTCGTAGGCGGCCTGTC
GCGTCGGCTGTGAAAACCCGGGGCTCAACCCCGGGCCTGCAGTCGATACGGGCAGGCTAGAGTCCGGCAG
GGGAGACTGGAATTCCTGGTGTAGCGGTGAAATGCGCAGATATCAGGAGGAACACCGGTGGCGAAGGCGG
GTCTCTGGGCCGGAACTGACGCTAAGGAGCGAAAGCGTGGGGAGCGAACAGGATTAGATACCCTGGTAGT
CCACGCCGTAAACGTTGGGCGCTAGGTGTGGGGGACCTTCCACGGCCTCCGTGCCGCAGCTAACGCATTA
AGCGCCCCGCCTGGGGAGTACGGCCGCAAGGCTAAAACTCAAAGGAATTGACGGGGGCCCGCACAAGCGG
CGGAGCATGTGGCTTAATTCGATGCAACGCGAAGAACCTTACCAGGGCTTGACATGCAGGGAAATCTCGT
AGAGATACGGGGTCCGTAAGGGTCCTGCACAGGTGGTGCATGGCTGTCGTCAGCTCGTGTCGTGAGATGT
TGGGTTAAGTCCCGCAACGAGCGCAACCCTCGTCCTATGTTGCCAGCGAGTTATGTCGGGGACTCATAGG
AGACTGCCGGGGTCAACTCGGAGGAAGGTGGGGATGACGTCAAGTCATCATGCCCCTTACGTCCTGGGCT
GCACACATGCTACAATGGCCGGTACAAAGGGCTGCGATACCGTGAGGTGGAGCGAATCCCAAAAAGCCGG
TCTCAGTTCGGATCGGGGTCTGCAACTCGACCCCGTGAAGTCGGAGTCGCTAGTAATCGCAGATCAGCAA
TGCTGCGGTGAATACGTTCCCGGGCCTTGTACACACCGCCCGTCACGTCACGAAAGTCGGTAACACCCGA
AGCCGGTGGCCTAACCCTTGTGGGGGGAGCCGTCGAAGGTGGGACCGGCGATTGGGACGAAGTCGTAACA
AGGTAGCCGTACCGGAAGGTGCGGCTGGATCACCTCCTTT

>gb|CP000249.1|:3751629-3753140 Frankia sp. CcI3, complete genome
TTGATGGAGAGTTTGATCCTGGCTCAGGACGAACGCTGGCGGCGTGCTTAACACATGCAAGTCGAGCGGG
GAGCTTCGGCTCTCAGCGGCGAACGGGTGAGTAACACGTGGGCAACCTGCCCCGAGCTCTGGAATAACCT
CGGGAAACCGGGGCTAATGCCGGATATGACATTGCCGGGCATCTGGTGGTGTGGAAAGATTTATCGGCTC
GGGATGGGCCCGCGGCCTATCAGCTTGTTGGTGGGGTGATGGCCTACCAAGGCGACGACGGGTAGCCGGC
CTGAGAGGGCGACCGGCCACACTGGGACTGAGACACGGCCCAGACTCCTACGGGAGGCAGCAGTGGGGAA
TATTGCGCAATGGGCGGAAGCCTGACGCAGCGACGCCGCGTGGGGGATGACGGCCTTCGGGTTGTAAACC
TCTTTCAGCAGGGACGAAGCGAGAGTGACGGTACCTGCAGAAGAAGCACCGGCCAACTACGTGCCAGCAG
CCGCGGTAATACGTAGGATGCAAGCGTTGTCCGGAATTATTGGGCGTAAAGAGCTCGTAGGCGGCCTGTT
GCGTCGGCTGTGAAAACCCGGGGCTCAACTCCGGGCCTGCAGTCGATACGGGCAGGCTAGAGTCCGGCAG
GGGAGACTGGAATTCCTGGTGTAGCGGTGAAATGCGCAGATATCAGGAGGAACACCGGTGGCGAAGGCGG
GTCTCTGGGCCGGAACTGACGCTAAGGAGCGAAAGCGTGGGGAGCGAACAGGATTAGATACCCTGGTAGT
CCACGCCGTAAACGTTGGGCGCTAGGTGTGGGGGACCTTCCACGGCCTCCGTGCCGCAGCTAACGCATTA
AGCGCCCCGCCTGGGGAGTACGGCCGCAAGGCTAAAACTCAAAGGAATTGACGGGGGCCCGCACAAGCGG
CGGAGCATGTGGCTTAATTCGATGCAACGCGAAGAACCTTACCAGGGCTTGACATGCAGGGAAATCTCGT
AGAGATACGGGGTCCGTAAGGGTCCTGCACAGGTGGTGCATGGCTGTCGTCAGCTCGTGTCGTGAGATGT
TGGGTTAAGTCCCGCAACGAGCGCAACCCTCGTCCTATGTTGCCAGCGAGTCGTGTCGGGGACTCATAGG
AGACTGCCGGGGTCAACTCGGAGGAAGGTGGGGATGACGTCAAGTCATCATGCCCCTTACGTCCTGGGCT
GCACACATGCTACAATGGCCGGTACAAAGGGCTGCGATGCCGTGAGGTGGAGCGAATCCCAAAAAGCCGG
TCTCAGTTCGGATCGGGGTCTGCAACTCGACCCCGTGAAGTCGGAGTCGCTAGTAATCGCAGATCAGCAA
TGCTGCGGTGAATACGTTCCCGGGCCTTGTACACACCGCCCGTCACGTCACGAAAGTCGGTAACACCCGA
AGCCGGTGGCCTAACCCTTGTGGGGGGAGCCGTCGAAGGTGGGACCGGCGATTGGGACGAAGTCGTAACA
AGGTAGCCGTACCGGAAGGTGCGGCTGGATCACCTCCTTTCT
>gi|261824163|gb|GQ891665.1| Frankia sp. CN3 16S ribosomal RNA gene, partial sequence; 16S-23S ribosomal RNA intergenic spacer, complete sequence; and 23S ribosomal RNA gene, partial sequence
CTTGTCCTATGTTGCCAGCGCGTTATGGGCGGGGACTCATACAAGACTGCCGGGGTCAACTCGGAGGAAG
GTGGGGATGACGTCAAGTCATCATGCCCCTTACGTCCTGGGCTGCACACATGCTACAATGGCCGGTACAA
TGGGCTGCGATACCGCGAGGTGGAGCGAATCCCAAAAAGCCGGTCTCAGTTCGGATCGGGGTCTGCAACT
CGACCCCGTGAAGTCGGAGTCGCTAGTAATCGCAGATCAGCATTGCTGCGGTGAATACGTTCCCGGGCCT
TGTACACACCGCCCGTCACGTCATGAAAGTCGGTAACACCCGAAGCCGGTGGCCTAACCCTTGTGGAGGG
AGCCGTCGAAGGTGGGACCGGCGATTAGGACGAAGTCGTAACAAGGTAACCGTACCGGAAGGTGCGGCTG
GATCACCTCCTTTCTAAGGAGCTTCTCACGGAGTTGTCCGTGGGGCCGGTTCCCGGCTGTGCATGCCGGG
CTGGTTTGCTCAGTGGCGTGGAATGCTGACTTCAGAGGCTGTTTTTCGGCTGGTTCGGTGGCTAGTACTC
TCTGCTCTTCGGGGTGGGGGTGGAACGGGCTGGCTGGTCGGGGATGGTTTCGTGTGGCGCGCTGTTGGGT
CCTGAGGGAGCGGAAATCGTTTCTTCGGTGGCCATCGACCCCCGGGTCTCCCTTTTGGGGAGGGTCGGGG
GGTTGGCTGCGGTACGGACCAGTCCTTCTAGACATCGGCCCCTGTGGGGGTGTCGGTACCCCTAGGGGTT
GGGGTCGTCCGTTCTTTGAGAACTGCACAGTGGACGCGAGCATCTGTGTGGCCAAGTTAGTAAGGGCAC
>gb|CP000820.1|:1274937-1276437 Frankia sp. EAN1pec, complete genome
AGAGTTTGATCCTGGCTCAGGACGAACGCTGGCGGCGTGCTTAACACATGCAAGTCGAGCGAGGAGCTTG
CTCCTAGCGGCGAACGGGTGAGTAACACGTGGGCAACCTGCCCCAAGCTCTGGAATAACTCCGGGAAACC
GGGGCTAATGCCGGATATGACATCATCGGGCATCTGGTGATGTGGAAAGATTTATCGGCTTGGGATGGGC
CCGCGGCCTATCAGCTTGTTGGTGGGGTGATGGCCTACCAAGGCGACGACGGGTAGCCGGCCTGAGAGGG
CGATCGGCCACACTGGGACTGAGACACGGCCCAGACTCCTACGGGAGGCAGCAGTGGGGAATATTGCGCA
ATGGGCGAAAGCCTGACGCAGCGACGCCGCGTGAGGGATGACGGCCTTCGGGTTGTAAACCTCTTTCAGC
AGGGACGAAGCGAGAGTGACGGTACCTGCAGAAGAAGCACCGGCCAACTACGTGCCAGCAGCCGCGGTAA
TACGTAGGGTGCAAGCGTTGTCCGGAATTATTGGGCGTAAAGAGCTCGTAGGCGGCCTGTCGCGTCGGCT
GTGAAAACCCGGGGCTCAACCCCGGGCCTGCAGTCGATACGGGCAGGCTAGAGTCCGGTAGGGGAGACTG
GAATTCCTGGTGTAGCGGTGAAATGCGCAGATATCAGGAGGAACACCGGTGGCGAAGGCGGGTCTCTGGG
CCGGAACTGACGCTAAGGAGCGAAAGCGTGGGGAGCGAACAGGATTAGATACCCTGGTAGTCCACGCCGT
AAACGTTGGGCGCTAGGTGTGGGGGACCTTCCACGGCCTCCGTGCCGCAGCTAACGCATTAAGCGCCCCG
CCTGGGGAGTACGGCCGCAAGGCTAAAACTCAAAGGAATTGACGGGGGCCCGCACAAGCGGCGGAGCATG
TGGCTTAATTCGATGCAACGCGAAGAACCTTACCAGGGCTTGACATGCAGAGAAATCCTGTAGAGATATG
GGGTCCTTAGGGGCTCTGCACAGGTGGTGCATGGCTGTCGTCAGCTCGTGTCGTGAGATGTTGGGTTAAG
TCCCGCAACGAGCGCAACCCTTGTCCTATGTTGCCAGCGAGTCATGTCGGGGACTCATAGGAGACTGCCG
GGGTCAACTCGGAGGAAGGTGGGGATGACGTCAAGTCATCATGCCCCTTACGTCCTGGGCTGCACACATG
CTACAATGGCCGGTACAATGGGCTGCGATACCGCGAGGTGGAGCGAATCCCAAAAAGCCGGTCTCAGTTC
GGATCGGGGTCTGCAACTCGACCCCGTGAAGTCGGAGTCGCTAGTAATCGCAGATCAGCAATGCTGCGGT

GAATACGTTCCCGGGCCTTGTACACACCGCCCGTCACGTCACGAAAGTCGGTAACACCCGAAGCCGGTGG
CCCAACCCTTGTGGGGGGAGCCGTCGAAGGTGGGACCGGCGATTGGGACGAAGTCGTAACAAGGTAGCCG
TACCGGAAGGTGCGGCTGGATCACCTCCTTT
>gi|261824138|gb|GQ891640.1| Frankia sp. EUN1f 16S ribosomal RNA gene, partial sequence; 16S-23S ribosomal RNA intergenic spacer, complete sequence; and 23S ribosomal RNA gene, partial sequence
CTTGTCCTATGTTGCCAGCGCGTTATGGCGGGGACTCATAGGAGACTGCCGGGGTCAACTCGGAGGAAGG
TGGGGATGACGTCAAGTCATCATGCCCCTTACGTCCTGGGCTGCACACATGCTACAATGGCCGGTACAAA
GGGCTGCGATGCCGTGAGGTGGAGCGAATCCCAAAAAGCCGGTCTCAGTTCGGATCGGGGTCTGCAACTC
GACCCCGTGAAGTCGGAGTCGCTAGTAATCGCAGATCAGCAATGCTGCGGTGAATACGTTCCCGGGCCTT
GTACACACCGCCCGTCACGTCACGAAAGTCGGTAACACCCGAAGCCGGTGGCCCAACCCTTGTGGGGGGA
GCCGTCGAAGGTGGGACCGGCGATTGGGACGAAGTCGTAACAAGGTAGCCGTACCGGAAGGTGCGGCTGG
ATCACCTCCTTTCTAAGGAGCTTTCTGGCTGGTGGCTTCCTGTAGTGGGGGGTTACTGGTCCAGGGCGAA
GGCCGGTCGAGTGGCTGGTCTGGCGTGCTCATGGGTGTGGAACGCTGACTTGTTCTGCCGGGTTGGTCTG
GTGCTCTGGGTTCCTGTGTGTTCCTGGGGTTGGGCTTTCCTGAATGATCGGTACGCTGTTGGGTTTTGAG
GGAGTGTGATGCCCTCGTGTACGTCCCTGCCCTGTCAGCCTGTTGGCTGCCGGGGTGGGTGTGCGGGCTG
GTTCGTGCGGGATACCGCCATCCGTGTGGGGTGGTTGGTCTCGTGGGGCGTGACTGGTTTCCGTTCGTAG
TTTGAGAACTGCACAGTGGACGCGAGCATCTTTATCTTTGTGGCCAAGTTATTAAGGGCGC
>gb|CP002801.1|:2958590-2960087 Frankia symbiont of Datisca glomerata, complete genome
AGAGTTTGATCCTGGCTCAGGACGAACGCTGGCGGCGTGCTTAACACATGCAAGTCGAGCGGGGGACTTC
GGTCTTCAGCGGCGAACGGGTGAGTAACACGTGGGCAACCTGCCCCGAGCTCTGGGATAACTTCGGGAAA
CCGGGGCTAATACCGGATATGACATTGCCGGGCATCTGGTGGTGTGGAAAGATTTATCGGCTCGGGATGG
GCCCGCGGCCTATCAGCTTGTTGGTGGGGTGATGGCCTACCAAGGCGACGACGGGTAGCCGGCCTGAGAG
GGCGATCGGCCACACTGGGACTGAGACACGGCCCAGACTCCTACGGGAGGCAGCAGTGGGGAATATTGCG
CAATGGGCGGAAGCCTGACGCAGCGACGCCGCGTGGGGGATGACGGCCTTCGGGTTGTAAACCTCTTTCA
GCAGGGACGAAGCGAAAGTGACGGTACCTGCAGAAGAAGCACCGGCCAACTACGTGCCAGCAGCCGCGGT
AATACGTAGGGTGCAAGCGTTGTCCGGAATTATTGGGCGTAAAGAGCTCGTAGGCGGCTTGTCACGTCGG
CTGTGAAATCCCGGGGCTCAACTCCGGGCGTGCAGTCGATACGGGCAGGCTAGAGTCCGGCAGGGGAGAC
TGGAATTCCTGGTGTAGCGGTGAAATGCGCAGATATCAGGAGGAACACCGGTGGCGAAGGCGGGTCTCTG
GGCCGGTACTGACGCTAAGGAGCGAAAGCGTGGGGAGCGAACAGGATTAGATACCCTGGTAGTCCACGCC
GTAAACGTTGGGCGCTAGGTGTGGGGGACCTTCCACGGCCTCCGTGCCGCAGCTAACGCATTAAGCGCCC
CGCCTGGGGAGTACGGCCGCAAGGCTAAAACTCAAAGGAATTGACGGGGGCCCGCACAAGCGGCGGAGCA
TGTGGCTTAATTCGATGCAACGCGAAGAACCTTACCAAGGCTTGACATGCAGGGAAATCTCGTAGAGATA
CGGGGTCCGTAAGGGCTCTGCACAGGTGGTGCATGGCTGTCGTCAGCTCGTGTCGTGAGATGTTGGGTTA
AGTCCCGCAACGAGCGCAACCCTCGTTCTATGTTGCCAGCGAGTAATGTCGGGGACTCATAGGAGACTGC
CGGGGTCAACTCGGAGGAAGGTGGGGATGACGTCAAGTCATCATGCCCCTTACGTCTTGGGCTGCACACA
TGCTACAATGGCCGGTACAAAGGGCTGCGATGCCGTGAGGTGGAGCGAATCCCAAAAAGCCGGTCTCAGT
TCGGATCGGGGTCTGCAACTCGACCCCGTGAAGTCGGAGTCGCTAGTAATCGCAGATCAGCAATGCTGCG
GTGAATACGTTCCCGGGCCTTGTACACACCGCCCGTCACGTCACGAAAGTCGGTAACACCCGAAGCCGGT
GGCCCAACCCTTGTGGGGGGAGCCGTCGAAGGTGGGACCGGCGATTGGGACGAAGTCGTAACAAGGTAGC
CGTACCGGAAGGTGCGGCTGGATCACCT
>gi|228721038|ref|NR_027601.1| Geodermatophilus obscurus DSM 43160 16S ribosomal RNA, partial sequence
TGATCCTGGCTCAGGACGAACGCTGGCGGCGTGCTTAACACATGCAAGTCGGACGGTGAACCTGCTTCGG
GTGGGGGATCAGTGCCGAACGGGTGAGTAACACGTGGCCAACCTGCCCCCGGCTCTGGGATAACTCCAAG
AAATTGGGGCTAATACCGGATGTGACCGCTGCCCGCATGGTCTGGTGGTGGAAAGGGTTTCCGGCTGGGG
ATGGACCCGCGGCCTATCAGCTTGTTGGTGGGGTAGTGGCCTACCAAGGCGACGACGGGTAGCCGGCCTG
AGAGGGTGACCGGCCAGACTGGGACTGAGACACGGCCCAGACTCCTACGGGAGGCAGCAGTGGGGAATAT
TGCACAATGGGCGGAAGCCTGACGCAGCGACGCCGCGTGGGGGATGACGGCCTTCGGGTTGTAAACCTCT
TTCAGCAGGGACGAAGCCGTGAGGTGACGGTACCTGCAGAAGAAGCACCGGCCAACTACGTGCCAGCAGC
CGCGGTAATACGTAGGGTGCAAGCGTTGTCCGGAATTATTGGGCGTAAAGAGCTCGTAGGCGGTTGGTCG
CGTCGGCTGTGAAAACCCGGAGATCAACTCCGGGCCTGCAGTCGATACGGGCCGACTTGAGTGTTGTAGG
GGAGACTGGAATTCCTGGTGTAGCGGTGAAATGCGCAGATATCAGGAGGAACACCGGTGGCGAAGGCGGG
TCTCTGGGCAACAACTGACGCTGAGGAGCGAAAGCGTGGGGAGCGAACAGGATTAGATACCCTGGTAGTC
CACGCCGCAAACGTTGGGCGCTAGGTGTGGGGGCCATTCCACGGTCTCCGTGCCGCAGATAACGCATTAA
GCGCCCCGCCTGGGGAGTACGGCCGCAAGGCTAAAACTCAAAGGAATTGACGGGGGCCCGCACAAGCGGC
GGAGCATGTTGCTTACTTCGATGCAACGCGAAGAACCTTACCTAGGCTTGACATGCACGGAAATCTCGCA

GAGATGCGGGGTCCGCAAGGGCCGTGCACAGGTGGTGCATAGTTGTCGCCAGCTCGTGTCGTGAGATGTT
GGGTTAAGTCCCGCAACGAGCGCAACCCTCGTTCCATGTTGCCAGCACGTGATGGTGGGGACTCATGGGA
GACTGCCGGGGTCAACTCGGAGGAAGGTGGGGATGACGTCAAATCATCATGCCCCTTATGTCTAGGGCTG
CAAACATGCTACAATGGCCGGAACAAAGGGCTGCGATACCGCAAGGTGGAGCGAATCCCAAAAAGCCGGT
CTCAGTTCGGATTGGGGTCTGCAACTCGACCCCATGAAGTTGGAGTCGCTAGTAATCGCAGATCAGCAAC
GCTGCGGTGAATACGTTCCCGGGGCTTGTACACACCGCCCGTCACGTCACGAAGGTCGTTAACGCCCGAA
GCCGGTGGCCCAACCCCTTGTGGGAGGGAGCCGTCGAGGCGGGGATCGGTGATTGGGACGAAGTCGTAAC
AAGGTTGCCGTA
>gb|CP000750.2|:2411132-2412643 Kineococcus radiotolerans SRS30216, complete genome
AGAGTTTGATCCTGGCTCAGGACGAACGCTGGCGGCGTGCTTAACACATGCAAGTCGAACGGTGAACCCC
TTCGGGGGGGATCAGTGGCGAACGGGTGAGTAACACGTGAGCAACCTGCCCCTGGCTCTGGGACAACCAC
TGGAAACGGTGGCTAATACCGGATACGACTCATCACCGCATGGTGTGTGGGTGGAAAGTTTTTCGGCTGG
GGATGGGCTCGCGGCCTATCAGCTTGTTGGTGGGGTAATGGCCTACCAAGGCGACGACGGGTAGCCGGCC
TGAGAGGGCGACCGGCCACACTGGGACTGAGACACGGCCCAGACTCCTACGGGAGGCAGCAGTGGGGAAT
ATTGCACAATGGGCGAAAGCCTGATGCAGCGACGCCGCGTGAGGGATGACGGCCTTCGGGTTGTAAACCT
CTTTCAGCTCCGAAGAAGCGAAAGTGACGGTAGGAGCAGAAGAAGCACCGGCTAACTACGTGCCAGCAGC
CGCGGTAATACGTAGGGTGCAAGCGTTGTCCGGAATTATTGGGCGTAAAGAGCTCGTAGGCGGTGTGTCG
CGTCTGCTGTGAAAACTCAGGGCTCAACTCTGAGCTTGCAGTGGGTACGGGCACACTTGAGTGCTGTAGG
GGAGACTGGAATTCCTGGTGTAGCGGTGAAATGCGCAGATATCAGGAGGAACACCGGTGGCGAAGGCGGG
TCTCTGGGCAGTTACTGACGCTGAGGAGCGAAAGCATGGGGAGCGAACAGGATTAGATACCCTGGTAGTC
CATGCCGTAAACGTTGGGCGCTAGGTGTGGGGTCCATTCCACGGATTCCGTGCCGCAGCTAACGCATTAA
GCGCCCCGCCTGGGGAGTACGGCCGCAAGGCTAAAACTCAAAGGAATTGACGGGGGCCCGCACAAGCGGC
GGAGCATGCGGATTAATTCGATGCAACGCGAAGAACCTTACCAAGGCTTGACATGCGCGGTGGAATCCCA
GAGATGGGGTGTCATTTAGTTGGTCGCGTACAGGTGGTGCATGGTTGTCGTCAGCTCGTGTCGTGAGATG
TTGGGTTAAGTCCCGCAACGAGCGCAACCCTCGTTCCATGTTGCCAGCACGTTATGGTGGGGACTCATGG
GAGACTGCCGGGGTCAACTCGGAGGAAGGTGGGGATGACGTCAAATCATCATGCCCCTTATGTCTTGGGC
TTCACGCATGCTACAATGGCCAGTACAGAGGGCTGCGATACCGTGAGGTGGAGCGAATCCCAAAAAGCTG
GTCTCAGTTCGGATCGGGGTCTGCAACTCGACCCCGTGAAGTCGGAGTCGCTAGTAATCGCAGATCAGCA
ACGCTGCGGTGAATACGTTCCCGGGCCTTGTACACACCGCCCGTCACGTCATGAAAGTCGGTAACACCCG
AAGCCGGTGGCCCAACCCGTAAGGGGGGGAGCTGTCGAAGGTGGGACTGGCGATTGGGACGAAGTCGTAA
CAAGGTAGCCGTACCGGAAGGTGCGGCTGGATCACCTCCTTT
>gi|311893446:4796513-4798037 Kitasatospora setae KM-6054 DNA, complete genome
TCACGGAGAGTTTGATCCTGGCTCAGGACGAACGCTGGCGGCGTGCTTAACACATGCAAGTCGAACGGTG
AAGCCCTTCGGGGTGGATCAGTGGCGAACGGGTGAGTAACACGTGGGGAATCTGCCCTGAACTCTGGGAC
AAGCCTTGGAAACGAGGTCTAATACCGGATACGACCGCTTCCCGCATGGGGGGTGGTGGAAAGCTCCGGC
GGTTCAGGATGATCCCGCGGCCTATCAGCTTGTTGGTGGGGTAACGGCCCACCAAGGCGACGACGGGTAG
CCGGCCTGAGAGGGCGACCGGCCACACTGGGACTGAGACACGGCCCAGACTCCTACGGGAGGCAGCAGTG
GGGAATATTGCACAATGGGCGAAAGCCTGATGCAGCGACGCCGCGTGAGGGATGACGGCCTTCGGGTTGT
AAACCTCTTTCAGCAGGGAAGAAGCGCAAGTGACGGTACCTGCAGAAGAAGCACCGGCTAACTACGTGCC
AGCAGCCGCGGTAATACGTAGGGTGCGAGCGTTGTCCGGAATTATTGGGCGTAAAGAGCTCGTAGGCGGC
CTGTCGCGTCGGATGTGAAAGCCCGGGGCTTAACCCCGGGTCTGCATTCGATACGGGCAGGCTAGAGTGT
GGTAGGGGAGATCGGAATTCCTGGTGTAGCGGTGAAATGCGCAGATATCAGGAGGAACACCGGTGGCGAA
GGCGGATCTCTGGGCCATTACTGACGCTGAGGAGCGAAAGCGTGGGGAGCGAACAGGATTAGATACCCTG
GTAGTCCACGCCGTAAACGTTGGGAACTAGGTGTTGGCGACATTCCACGTCGTCGGTGCCGCAGCTAACG
CATTAAGTTCCCCGCCTGGGGAGTACGGCCGCAAGGCTAAAACTCAAAGGAATTGACGGGGGCCCGCACA
AGCAGCGGAGCATGTGGCTTAATTCGACGCAACGCGAAGAACCTTACCAAGGCTTGACATACACCGGAAA
CTGGTAGAGATATCAGCCCCCTTGTGGTCGGTGTACAGGTGGTGCATGGTTGTCGTCAGCTCGTGTCGTG
AGATGTTGGGTTAAGTCCCGCAACGAGCGCAACCCTTGTTCTGTGTTGCCAGCATGCCTTTCGGGGTGAT
GGGGACTCACAGGAGACTGCCGGGGTCAACTCGGAGGAAGGTGGGGACGACGTCAAATCATCATGCCCCT
TATGTCTTGGGCTGCACACGTGCTACAATGGCCGGTACAAAGGGCTGCGATGCCGCGAGGCGGAGCGAAT
CCCAAAAAGCCGGTCTCAGTTCGGATTGGGGTCTGCAACTCGACCCCATGAAGTTGGAGTTGCTAGTAAT
CGCAGATCAGCATGCTGCGGTGAATACGTTCCCGGGCCTTGTACACACCGCCCGTCACGTCACGAAAGTC
GGTAACACCCGAAGCCGGTGGCCTAACCCGTGAGGGGAGGAGCCGTCGAAGGTGGGACCAGCGATTGGGA
CGAAGTCGTAACAAGGTAGCCGTACCGGAAGGTGCGGCTGGATCACCTCCTTTCT
>gi|75709237|gb|DQ181633.1| Kutzneria sp. 744 16S ribosomal RNA gene, partial sequence

GCTGGCGGCGTGCTTAACACATGCAAGTCGAGCGGTAAGGCCCTTCGGGGTACACGAGCGGCGAACGGGT
GAGTAACACGTGGGCAACCTGCCCTGCACTCTGGGATAAGCCCGGGAAACTGGGTCTAATACCGGATATG
ACCTTCTGCCGGCATGGTGGAGGGTGGAAAGTTCCGGCGGTGCAGGATGGGCCCGCGGCCTATCAGCTTG
TTGGTGGGGTAGTGGCCTACCAAGGCGACGACGGGTAGCCGGCCTGAGAGGGCGACCGGCCACACTGGGA
CTGAGACACGGCCCAGACTCCTACGGGAGGCAGCAGTGGGGAATATTGCGCAATGGGCGAAAGCCTGACG
CAGCGACGCCGCGTGAGGGATGACGGCCTTCGGGTTGTAAACCTCTTTCAGCAGGGACGAAGCGCAAGTG
ACGGTACCTGCAGAAGAAGCACCGGCTAACTACGTGCCAGCAGCCGCGGTAATACGTAGGGTGCGAGCGT
TGTCCGGAATTATTGGGCGTAAAGAGCTCGTAGGCGGTTTGTCGCGTCGACTGTGAAAACCTACAGCTTA
ACTGTGGGCTTGCAGTCGATACGGGCAGACTTGAGTTCGGTAGGGGAGACTGGAATTCCTGGTGTAGCGG
TGAAATGCGCAGATATCAGGAGGAACACCGGTGGCGAAGGCGGGTCTCTGGGCCGATACTGACGCTGAGG
AGCGAAAGCGTGGGGAGCGAACAGGATTAGATACCCTGGTAGTCCACGCCGTAAACGGTGGGAACTAGGT
GTGGGGGGCTTCCACGTCCTCCGTGCCGTAGCTAACGCATTAAGTTCCCCGCCTGGGGAGTACGGCCGCA
AGGCTAAAACTCAAAGGAATTGACGGGGGCCCGCACAAGCGGCGGAACATGTGGATTAATTCGATGCAAC
GCGAAGAACCTTACCTGGGCTTGACATGCACTGGAAACCAGTAGAGATATTGGCCCCCTTGTGGCCGGTG
TACAGGTGGTGCATGGCTGTCGTCAGCTCGTGTCGTGAGATGTTGGGTTAAGTCCCGCAACGAGCGCAAC
CCTCGTTCCATGTTGCCAGCGCGTAATGGCGGGGACTCATGGGAGACTGCCGGGGTCAACTCGGAGGAAG
GTGGGGATGACGTCAAGTCATCATGCCCCTTATGTCCAGGGCTTCACACATGCTACAATGGCCGGTACAA
AGGGCTGCTAAGCCGCGAGGTGGAGCGAATCCCATAAAGCCGGTCTCAGTTCGGATCGGGGTCTGCAACT
CGACCCCGTGAAGTCGGAGTCGCTAGTAATCGCAGATCAGCAACGCTGCGGTGAATACGTTCCCGGGCCT
TGTACACACCGCCCGTCACGTCACGAAAGTCGGTAACACCCGAAGCCCGTGGCCCAACCCGCAAGGGAGG
GAGCGGTCGAAGGTGGGACTGGCGA
>gb|CP002162.1|:2546563-2548065 Micromonospora aurantiaca ATCC 27029, complete genome
AGAGTTTGATCCTGGCTCAGGACGAACGCTGGCGGCGTGCTTAACACATGCAAGTCGAGCGGAAAGGCCC
TTCGGGGTACTCGAGCGGCGAACGGGTGAGTAACACGTGAGCAACCTGCCCCAGGCTTTGGGATAACCCC
GGGAAACCGGGGCTAATACCGAATATGACCTCTGACCGCATGGTTGGTGGTGGAAAGTTTTTCGGCTTGG
GATGGGCTCGCGGCCTATCAGCTTGTTGGTGGGGTGATGGCCTACCAAGGCGACGACGGGTAGCCGGCCT
GAGAGGGCGACCGGCCACACTGGGACTGAGACACGGCCCAGACTCCTACGGGAGGCAGCAGTGGGGAATA
TTGCACAATGGGCGGAAGCCTGATGCAGCGACGCCGCGTGAGGGATGACGGCCTTCGGGTTGTAAACCTC
TTTCAGCAGGGACGAAGCGTAAGTGACGGTACCTGCAGAAGAAGCGCCGGCCAACTACGTGCCAGCAGCC
GCGGTAAGACGTAGGGCGCGAGCGTTGTCCGGATTTATTGGGCGTAAAGAGCTCGTAGGCGGCTTGTCGC
GTCGACCGTGAAAACTTGGGGCTCAACCCCAAGCCTGCGGTCGATACGGGCAGGCTAGAGTTCGGTAGGG
GAGACTGGAATTCCTGGTGTAGCGGTGAAATGCGCAGATATCAGGAGGAACACCGGTGGCGAAGGCGGGT
CTCTGGGCCGATACTGACGCTGAGGAGCGAAAGCGTGGGGAGCGAACAGGATTAGATACCCTGGTAGTCC
ACGCTGTAAACGTTGGGCGCTAGGTGTGGGGGGCCTCTCCGGTTCCCTGTGCCGCAGCTAACGCATTAAG
CGCCCCGCCTGGGGAGTACGGCCGCAAGGCTAAAACTCAAAGGAATTGACGGGGGCCCGCACAAGCGGCG
GAGCATGCGGATTAATTCGATGCAACGCGAAGAACCTTACCTGGGTTTGACATGGCCGCAAAACTGTCAG
AGATGGCAGGTCCTTCGGGGGCGGTCACAGGTGGTGCATGGCTGTCGTCAGCTCGTGTCGTGAGATGTTG
GGTTAAGTCCCGCAACGAGCGCAACCCTCGTTCGATGTTGCCAGCGCGTTATGGCGGGGACTCATCGAAG
ACTGCCGGGGTCAACTCGGAGGAAGGTGGGGATGACGTCAAGTCATCATGCCCCTTATGTCCAGGGCTTC
ACGCATGCTACAATGGCCGGTACAATGGGCTGCGATACCGTGAGGTGGAGCGAATCCCAAAAAGCCGGTC
TCAGTTCGGATCGGGGTCTGCAACTCGACCCCGTGAAGTCGGAGTCGCTAGTAATCGCAGATCAGCAACG
CTGCGGTGAATACGTTCCCGGGCCTTGTACACACCGCCCGTCACGTCACGAAAGTCGGCAACACCCGAAG
CCGGTGGCCCAACCCTTGTGGAGGGAGCCGTCGAAGGTGGGGCTGGCGATTGGGACGAAGTCGTAACAAG
GTAGCCGTACCGGAAGGTGCGGCTGGATCACCT
>gi|343201587|ref|NR_042313.1| Micromonospora lupini strain : Lupac 14N 16S ribosomal RNA, partial sequence
TAGAGTTTGATGCATGGCTCAGGACGANCGCTGGCGGCGTGCTTAACACATGCAAGTCGAGCGGAAAGGC
CCTTCGGGGTACTCGAGCGGCGAACGGGTGAGTAACACGTGAGCAACCTGCCCCAAGCTTTGGGATAACC
CCGGGAAACCGGGGCTAATACCGAATATTACTTCTGGCCGCATGGCTGGGGGTGGAAAGTTTTTCGGCTT
GGGATGGGCTCGCGGCCTATCAGCTTGTTGGTGGGGTGATGGCCTACCAAGGCGACGACGGGTAGCCGGC
CTGAGAGGGCGACCGGCCACACTGGGACTGAGACACGGCCCAGACTCCTACGGGAGGCAGCAGTGGGGAA
TATTGCACAATGGGCGGAAGCCTGATGCAGCGACGCCGCGTGAGGGATGACGGCCTTCGGGTTGTAAACC
TCTTCAGCAGGGACGAAGCGAGAGTGACGGTACCTGCAGAAGAAGCACCGGCCAACTACGTGCCAGCAGC
CGCGGTAAGACGTAGGGTGCGAGCGTTGTCCGGATTTATTGGGCGTAAAGAGCTCGTAGGCGGCTTGTCG
CGTCGACCGTGAAAACTTGGGGCTCAACCCCAAGCCTGCGGTCGATACGGGCAGGCTAGAGTTCGGTAGG
GGAGACTGGAATTCCTGGTGTAGCGGTGAAATGCGCAGATATCAGGAGGAACACCGGTGGCGAAGGCGGG
TCTCTGGGCCGATACTGACGCTGAGGAGCGAAAGCGTGGGGAGCGAACAGGATTAGATACCCTGGTAGTC

CACGCTGTAAACGTTGGGCGCTAGGTGTGGGGGGCCTCTCCGGTTCCCTGTGCCGCAGCTAACGCATTAA
GCGCCCCGCCTGGGGAGTACGGCCGCAAGGCTAAAACTCAAAGGAATTGACGGGGGCCCGCACAAGCGGC
GGAGCATGCGGATTAATTCGATGCAGACGCGAAGAACCTTACCTGGGTTTGACATGGCCGCAAAACCTCC
AGAGATGGGGGGTTCCTTCGGGGGCGGTCACAGGGGGTGCATGGCAGTCGTCAGCTTGTGTCGTGAGATG
TTGGGTTAAGTCCCGCAAACGAGCGCAAACCCTCGTTCGATGTTGCCAGCGCGTTATGGCGGGGACTCAT
CGAAGACTGCCGGGGTCAAACTCGGAGGAAGGTGGGGATGACGTCAAGTCATCATGCCCCTTATGTCCAG
GGCTTCACGCATGCTACAATGGCCGGTACAATGGGCTGCGATACCGTGAGGTGGAGCGAATCCCAAAAAG
CCGGTCTCAGTTCGGATCGGGGTCTGCAACTCGACCCCGTGAAGTCGGAGTCGCTAGTAATCGCAGATCA
GCAACGTCTGCGGTGAATACGTTCCCGGGCCTTGTACACACCGCCCGTCACGTCACGAAAGTCGGCAACA
GCCCGAAGCCGGTGGCCCAACCCTTGTGGAAAGAGCCGTCGAAGGTGGGGCTGGCGA
>gi|444303992|ref|NR_074416.1| Micromonospora sp. L5 strain L5 16S
ribosomal RNA, complete sequence
AGAGTTTGATCCTGGCTCAGGACGAACGCTGGCGGCGTGCTTAACACATGCAAGTCGAGCGGAAAGGCCC
TTCGGGGTACTCGAGCGGCGAACGGGTGAGTAACACGTGAGCAACCTGCCCCAGGCTTTGGGATAACCCC
GGGAAACCGGGGCTAATACCGAATATGACCTCTGACCGCATGGTTGGTGGTGGAAAGTTTTTCGGCTTGG
GATGGGCTCGCGGCCTATCAGCTTGTTGGTGGGGTGATGGCCTACCAAGGCGACGACGGGTAGCCGGCCT
GAGAGGGCGACCGGCCACACTGGGACTGAGACACGGCCCAGACTCCTACGGGAGGCAGCAGTGGGGAATA
TTGCACAATGGGCGGAAGCCTGATGCAGCGACGCCGCGTGAGGGATGACGGCCTTCGGGTTGTAAACCTC
TTTCAGCAGGGACGAAGCGTAAGTGACGGTACCTGCAGAAGAAGCGCCGGCCAACTACGTGCCAGCAGCC
GCGGTAAGACGTAGGGCGCGAGCGTTGTCCGGATTTATTGGGCGTAAAGAGCTCGTAGGCGGCTTGTCGC
GTCGACCGTGAAAACTTGGGGCTCAACCCCAAGCCTGCGGTCGATACGGGCAGGCTAGAGTTCGGTAGGG
GAGACTGGAATTCCTGGTGTAGCGGTGAAATGCGCAGATATCAGGAGGAACACCGGTGGCGAAGGCGGGT
CTCTGGGCCGATACTGACGCTGAGGAGCGAAAGCGTGGGGAGCGAACAGGATTAGATACCCTGGTAGTCC
ACGCTGTAAACGTTGGGCGCTAGGTGTGGGGGGCCTCTCCGGTTCCCTGTGCCGCAGCTAACGCATTAAG
CGCCCCGCCTGGGGAGTACGGCCGCAAGGCTAAAACTCAAAGGAATTGACGGGGGCCCGCACAAGCGGCG
GAGCATGCGGATTAATTCGATGCAACGCGAAGAACCTTACCTGGGTTTGACATGGCCGCAAAACTGTCAG
AGATGGCAGGTCCTTCGGGGGCGGTCACAGGTGGTGCATGGCTGTCGTCAGCTCGTGTCGTGAGATGTTG
GGTTAAGTCCCGCAACGAGCGCAACCCTCGTTCGATGTTGCCAGCGCGTTATGGCGGGGACTCATCGAAG
ACTGCCGGGGTCAACTCGGAGGAAGGTGGGGATGACGTCAAGTCATCATGCCCCTTATGTCCAGGGCTTC
ACGCATGCTACAATGGCCGGTACAATGGGCTGCGATACCGTGAGGTGGAGCGAATCCCAAAAAGCCGGTC
TCAGTTCGGATCGGGGTCTGCAACTCGACCCCGTGAAGTCGGAGTCGCTAGTAATCGCAGATCAGCAACG
CTGCGGTGAATACGTTCCCGGGCCTTGTACACACCGCCCGTCACGTCACGAAAGTCGGCAACACCCGAAG
CCGGTGGCCCAACCCTTGTGGAGGGAGCCGTCGAAGGTGGGGCTGGCGATTGGGACGAAGTCGTAACAAG
GTAGCCGTACCGGAAGGTGCGGCTGGATCACCT
>gi|444304055|ref|NR_074479.1| Modestobacter marinus strain BC501 16S
ribosomal RNA, complete sequence
AGAGTTTGATCCTGGCTCAGGACGAACGCTGGCGGCGTGCTTAACACATGCAAGTCGAGCGAGGCCCTCC
TTCGGGGGGTGCCCTAGCGGCGAACGGGTGAGTAACACGTGGGCAACCTGCCCTCAGCTCTGGGATAACT
CCAAGAAATTGGTGCTAATACCGGATGTGACCGCTGACCGCATGGTCTGGTGGTGGAAAGATTCATCGGC
TGAGGATGGGCCCGCGGCCTATCAGCTTGTTGGTGGGGTAATGGCCTACCAAGGCGACGACGGGTAGCCG
GCCTGAGAGGGTGACCGGCCACACTGGGACTGAGACACGGCCCAGACTCCTACGGGAGGCAGCAGTGGGG
AATATTGCGCAATGGGCGAAAGCCTGACGCAGCGACGCCGCGTGAGGGATGACGGCCTTCGGGTTGTAAA
CCTCTTTCAGTAGGGACGAAGCGCAAGTGACGGTACCTACAGAAGAAGCACCGGCCAACTACGTGCCAGC
AGCCGCGGTAATACGTAGGGTGCAAGCGTTGTCCGGAATTATTGGGCGTAAAGAGCTCGTAGGCGGTCTG
TCACGTCGGCTGTGAAAACCCGAGGCTCAACCTCGGGCCTGCAGTCGATACGGGCAAACTAGAGTACTGC
AGGGGAGACTGGAATTCCTGGTGTAGCGGTGAAATGCGCAGATATCAGGAGGAACACCGGTGGCGAAGGC
GGGTCTCTGGGCAGTAACTGACGCTGAGGAGCGAAAGCGTGGGGAGCGAACAGGATTAGATACCCTGGTA
GTCCACGCCGTAAACGTTGGGCGCTAGGTGTGGGGGCCATTCCACGGTCTCCGTGCCGCAGCTAACGCAT
TAAGCGCCCCGCCTGGGGAGTACGGCCGCAAGGCTAAAACTCAAAGGAATTGACGGGGGCCCGCACAAGC
GGCGGAGCATGTTGCTTAATTCGATGCAACGCGAAGAACCTTACCTAGGCTTGACATGCACGGAAATCTC
GTAGAGATACGGGGTGCCTTTGGCGTCGTGCACAGGTGGTGCATGGTTGTCGTCAGCTCGTGTCGTGAGA
TGTTGGGTTAAGTCCCGCAACGAGCGCAACCCTCGTTCTATGTTGCCAGCACGTGATGGTGGGGACTCAT
AGGAGACTGCCGGGGTCAACTCGGAGGAAGGTGGGGATGACGTCAAATCATCATGCCCCTTATGTCTAGG
GCTGCAAACATGCTACAATGGCCGGTACAAAGGGCTGCGATACCGCGAGGTGGAGCGAATCCCAAAAAGC
CGGTCTCAGTTCGGATTGGGGTCTGCAACTCGACCCCATGAAGTTGGAGTCGCTAGTAATCGCAGATCAG
CAACGCTGCGGTGAATACGTTCCCGGGCCTTGTACACACCGCCCGTCACGTCACGAAAGTCGGTAACGCC
CGAAGCCGGTGGCCCAACCCTTGTGGAGGGAGCCGTCGAAGGCGGGATCGGCGATTGGGACGAAGTCGTA
ACAAGGTAGCCGTACCGGAAGGTGCGGCTGGATCACCT

>gi|228721225|ref|NR_027604.1| Nakamurella multipartita DSM 44233 16S ribosomal RNA, partial sequence
GACGAACGCTGGCGGCGTGCTTAACACATGCAAGTCGAACGGAAAGGCCCTTCGGGGTACTCGAGTGGCG
AACGGGTGAGTAACACGTGGGTAACCTGCCCTCAGCTTCGGGATAACTCCGGGAAACCGGGGCTAATACC
GGATATTCATTGTCTGCCGCATGGTGGGTGGTGGAAAGTTTTTTCGGCTGGGGATGGGCCCGCGGCCTAT
CAGCTTGTTGGTGGGGTGATGGCCTACCAAGGCGTCGACGGGTAGCCGGCCTGAGAGGGCGACCGGCCAC
ACTGGGACTGAGACACGGCCCAGACTCCTACGGGAGGCAGCAGTGGGGAATATTGCGCAATGGGCGGAAG
CCTGACGCAGCGACGCCGCGTGAGGGATGACGGCCTTCGGGTTGTAAACCTCTTTCAGCAGGGACGAATG
TAGACGGTACCTGTAGAAGAAGCACCGGCCAACTACGTGCCAGCAGCCGCGGTAATACGTAGGGTGCGAG
CGTTGTCCGGAATTATTGGGCGTAAAGAGCTCGTAGGCGGTCTGTCGCGTCGAATGTGAAAACCCGGGGC
TCAACTCCGGGCTTGCATTCGATACGGGCAGGCTAGAGTTCGGTAGGGGAGTCTGGAATTCCTGGTGTAG
CGGTGAAATGCGCAGATATCAGGAGGAACACCGGTGGCGAAGGCGGGACTCTGGGCCGATACTGACGCTG
AGGAGCGAAAGCGTGGGGAGCGAACAGGATTAGATACCCTGGTAGTCCACGCCGTAAACGTTGGGCGCTA
GGTGTGGGGGTCCTTCCACGATCTCCGTGCCGCAGCTAACGCATTAAGCGCCCCGCCTGGGGAGTACGGC
CGCAAGGCTAAAACTCAAAGGAATTGACGGGGGCCCGCACAAGCGGCGGAGCATGCGGATTAATTCGATG
CAACGCGAAGAACCTTACCAAGGCTTGACATGCACGGGAAAGTTCCAGAGATGGTTCCCCCGCAAGGTCC
GTGCACAGGTGGTGCATGGTTGTCGTCAGCTCGTGTCGTGAGATGTTGGGTTAAGTCCCGCAACGAGCGC
AACCCTTGTCTTATGTTGCCAGCACGTGATGGTGGGGACTCATAAGAGACTGCCGGGGTCAACTCGGAGG
AAGGTGGGGATGACGTCAAATCATCATGCCCCTTATGTCTTGGGCTTCACGCATGCTACAATGGCCGGTA
CAAAGGGCTGCGAAACCGTGAGGTGGAGCGAATCCCAAAAAGCCGGTCTCAGTTCGGATTGGGGTCTGCA
ACTCGACCCCATGAAGTCGGAGTCGCTAGTAATCGCAGATCAGCAACGCTGCGGTGAATACGTTCCCGGG
CCTTGTACACACCGCCCGTCACGTCACGAAAGTCGGTAACACCCGAAGCCAGTGGCTCAACCCCTTGTGG
GGAGGGAGCTGTCGAAGGTGGGATCGGCGATTGGGACGAAGTCGTAACAAGGTAGCCGTACCGGAAGG
>Nocardioidaceae_bacterium_Broad_1
GACGAACGCTGGCGGCGTGCTTAACACATGCAAGTCGAGCGGAAAGGCCCTTCGGGGTACTCGAGCGGCG
AACGGGTGAGTAACACGTGAGTAATCTGCCCCAGGCTCTGGGATAGCTACCGGAAACGGTGATTAATACC
GGATACGACAACTTCCTGCATGGGATGGTTGTGGAAAGTTTTTCGGCCTGGGATGTGCTCGCGGCCTATC
AGCTTGTTGGTGAGGTAATGGCTCACCAAGGCTTCGACGGGTAGCCGGCCTGAGAGGGTGACCGGCCACA
CTGGGACTGAGACACGGACCAGACTCCTACGGAGCCAGCAGTGGGGATATTGGACAATGGGCGGAAGCTG
ATCCAGCAACGCCGCGGGGAGGGATGACGGCCTTTCGGGTTGTAAACTTCTTTCAGCACAGACGAAGCCG
CAAGTTGACGGTATGTGCAGAAAGAAGGACCGGGCCAACTACGTGCCAGCAGCCGCCGGTAATACGTAGG
GTCCGAGCGTTGTCCGGAATTTATTTGGGCGTAAAGGGCTCGTAGGCGGTCTTGTCNNNNNNNNNNNNNN
NNNNNNNNNNNNNNNNNNNNNNNNNNNNNNNNNNNNNNNNNNNNNNNNNNNNNNNNNNNNNNNNNNNNNN
NNNNNNNNNNNNNNNNNNNNNNNNNNNNNNNNNNNNNNNNNNNNNNNNNNNNNNNNNNNNNNNNNNNNNN
NNNNNNNNNNNNNNNNNNNNNNNNNNNNNNNNNNNNNNNNNNNNNNNNNNNNNNNNNNNNNNNNNNNNNN
NNNNNNNNNNNNNNNNNNNNNNNNNNNNNNNNNNNNNNNNNNNNNNNNNNNNNNNNNNNNNNNNNNNNN
NNNNNNNNNNNNNNNNNNNNNNNNNNNNNNNNNNNNNNNNNNNNNNNNNNNNNNNNNNNNNNNNNNNNNN
NNNNNNNNNNNNNNNNNNNNNNNNNNNNNNNNNNNNNNNNNNNNNNNNNNNNNNNNNNNNNNNNNNNNNN
NNNNNNNNNNNNNNNNNNNNNNNNNNNNNNNNNNNNNNNNNNNNNNNNNNNNNNNNNNNNNNNNNNNNNN
NNNNNNNNNNNNNNNNNNNNNNNNNNNNNNNNNNNNNNNNNNNNNNNNNNNNNNNNNNNNNNNNNNNNNN
NNNNNNNNNNNNNNNNNNNNNNNNNNNNNNNNNNNNNNNNNNNNNNNNNNNNNNNNNNNNNNNNNNNNNN
NNNNNNNNNNNNNNNNNNNNNNNNNNNNNNNNNNNNNNNNNNNNNNNNNNNNNNNNNNNNNNNNNNNNNN
NNNNNNNNNNNNNNNNNNNNNNNNNNNNGGTGAATACGTTCCCGGTCTTGTACACACGCCCGTCACGTCA
CGAAAGTCGGCAACACCCGAAGCCAGTGGCCCAACCCTTGTGGGGGGAGCTGTCGAAGGTGGGGCTGGCG
ATTGGGACGAAGTCGTAACAAGGTAGCCGTACCGGAAGG
>gb|CP000509.1|:2113497-2115016 Nocardioides sp. JS614, complete genome
AGAGTTTGATCCTGGCTCAGGACGAACGCTGGCGGCGTGCTTAACACATGCAAGTCGAGCGGAAAGGCCC
TTTCGGGGGTACTCGAGCGGCGAACGGGTGAGTAACACGTGAGTAATCTGCCCTTCACTTTGGGATAACT
ACCGGAAACGGTGGCTAATACCGGATACGACACAGGTACGCATGTGCTCTGTGTGGAAAGTTTTTTCGGT
GGGGGATGTGCTCGCGGCCTATCAGCTTGTTGGTGGGGTAATGGCCTACCAAGGCTTCGACGGGTAGCCG
GCCTGAGAGGGTGACCGGCCACACTGGGACTGAGACACGGCCCAGACTCCTACGGGAGGCAGCAGTGGGG
AATATTGGACAATGGGCGGAAGCCTGATCCAGCAACGCCGCGTGAGGGATGACGGCCTTCGGGTTGTAAA
CCTCTTTCAGTACCGACGAAGCACCGGTGATGGTGGTGACGGTAGGTACAGAAGAAGCACCGGCCAACTA
CGTGCCAGCAGCCGCGGTAATACGTAGGGTGCGAGCGTTGTCCGGAATTATTGGGCGTAAAGGGCTCGTA
GGCGGTTTGTCGCGTCGGGAGTGAAAACACTGGGCTTAACTCAGTGCTTGCTTTCGATACGGGCAGACTA
GAGGCATGCAGGGGAGAACGGAATTCCTGGTGTAGCGGTGAAATGCGCAGATATCAGGAGGAACACCGGT
GGCGAAGGCGGTTCTCTGGGCATGTCCTGACGCTGAGGAGCGAAAGTGTGGGGAGCGAACAGGATTAGAT
ACCCTGGTAGTCCACACCGTAAACGTTGGGCGCTAGGTGTGGGATCCATTCCACGGGTTCCGTGCCGCAG

CTAACGCATTAAGCGCCCCGCCTGGGGAGTACGGCCGCAAGGCTAAAACTCAAAGGAATTGACGGGGGCC
CGCACAAGCGGCGGAGCATGCGGATTAATTCGATGCAACGCGAAGAACCTTACCTGGGTTTGACATACGC
CGGAAAGCGCCAGAGATGGTGCCCCTTTTAGTCGGTGTACAGGTGGTGCATGGCTGTCGTCAGCTCGTGT
CGTGAGATGTTGGGTTAAGTCCCGCAACGAGCGCAACCCTCGTCCTATGTTGCCAGCACGTGATGGTGGG
GACTCATAGGAGACTGCCGGGGTCAACTCGGAGGAAGGTGGGGATGACGTCAAGTCATCATGCCCCTTAT
GTCCAGGGCTTCACGCATGCTACAATGGCCGGTACAAAGGGCTGCGATGCTGTAAGGCGGAGCGAATCCC
AAAAAGCCGGTCTCAGTTCGGATTGGGGTCTGCAACTCGACCCCATGAAGTCGGAGTCGCTAGTAATCGC
AGATCAGCAACGCTGCGGTGAATACGTTCCCGGGCCTTGTACACACCGCCCGTCACGTCACGAAAGTCGG
CAACACCCGAAGCCGGTGGCCTAACCCTTGTGGGGGGAGCCGTCGAAGGTGGGGCTGGCGATTGGGACGA
AGTCGTAACAAGGTAGCCGTACCGGAAGGTGCGGCTGGATCACCTCCTTT
>gi|444304183|ref|NR_074607.1| Nocardiopsis alba ATCC BAA-2165 strain
ATCC BAA-2165 16S ribosomal RNA, complete sequence
GAGTTTGATCCTGGCTCAGGACGAACGCTGGCGGCGTGCTTAACACATGCAAGTCGAGCGGTAAGGCCCT
TCGGGGTACACGAGCGGCGAACGGGTGAGTAACACGTGAGTAACCTGCCCCTGACTCTGGGATAAGCGGT
GGAAACGCCGTCTAATACCGGATACGACCTTCCGCCTCATGGTGGAGGGTGGAAAGTTTTTTCGGTCAGG
GATGGGCTCGCGGCCTATCAGCTTGTTGGTGGGGTAACGGCCTACCAAGGCGATTACGGGTAGCCGGCCT
GAGAGGGCGACCGGCCACACTGGGACTGAGACACGGCCCAGACTCCTGCGGGAGGCAGCAGTGGGGAATA
TTGCGCAATGGGCGAAAGCCTGACGCAGCGACGCCGCGTGGGGGATGACGGCCTTCGGGTTGTAAACCTC
TTTTACCACCAACGCAGGCTCCGGGTTTTCTCGGGGTTGACGGTAGGTGGGGAATAAGGACCGGCTAACT
ACGTGCCAGCAGCCGCGGTAATACGTAGGGTCCGAGCGTTGTCCGGAATTATTGGGCGTAAAGAGCTCGT
AGGCGGCGTGTCGCGTCTGCTGTGAAAGACCGGGGCTTAACTCCGGTTCTGCAGTGGATACGGGCATGCT
AGAGGTAGGTAGGGGAGACTGGAATTCCTGGTGTAGCGGTGAAATGCGCAGATATCAGGAGGAACACCGG
TGGCGAAGGCGGGTCTCTGGGCCTTACCTGACGCTGAGGAGCGAAAGCATGGGGAGCGAACAGGATTAGA
TACCCTGGTAGTCCATGCCGTAAACGTTGGGCGCTAGGTGTGGGGACTTTCCACGGTTTCCGCGCCGTAG
CTAACGCATTAAGCGCCCCGCCTGGGGAGTACGGCCGCAAGGCTAAAACTCAAAGGAATTGACGGGGGCC
CGCACAAGCGGCGGAGCATGTTGCTTAATTCGACGCAACGCGAAGAACCTTACCAAGGTTTGACATCACC
CGTGGACCTGTAGAGATACAGGGTCATTTAGTTGGTGGGTGACAGGTGGTGCATGGCTGTCGTCAGCTCG
TGTCGTGAGATGTTGGGTTAAGTCCCGCAACGAGCGCAACCCTTGTTCCATGTTGCCAGCACGTAATGGT
GGGGACTCATGGGAGACTGCCGGGGTCAACTCGGAGGAAGGTGGGGACGACGTCAAGTCATCATGCCCCT
TATGTCTTGGGCTGCAAACATGCTACAATGGCCGGTACAATGGGCGTGCGATACCGTAAGGTGGAGCGAA
TCCCTTAAAGCCGGTCTCAGTTCGGATTGGGGTCTGCAACTCGACCCCATGAAGGTGGAGTCGCTAGTAA
TCGCGGATCAGCAACGCCGCGGTGAATACGTTCCCGGGCCTTGTACACACCGCCCGTCACGTCATGAAAG
TCGGCAACACCCGAAACTTGCGGCCTAACCCTTCGGGGAGGGAGTGAGTGAAGGTGGGGCTGGCGATTGG
GACGAAGTCGTAACAAGGTAGCCGTACCGGAAGGTGCGGCTGGATCACC
>gb|CP002040.1|:67127-68647 Nocardiopsis dassonvillei subsp.
dassonvillei DSM 43111 chromosome 1, complete sequence
AGAGTTTGATCCTGGCTCAGGACGAACGCTGGCGGCGTGCTTAACACATGCAAGTCGAGCGGTAAGGCCC
TTCGGGGTACACGAGCGGCGAACGGGTGAGTAACACGTGAGCAACCTGCCCCTGACTCTGGGATAAGCGG
TGGAAACGCCGTCTAATACCGGATACGACCCGCCACCTCATGGTGGAGGGTGGAAAGTTTTTCGGTCAGG
GATGGGCTCGCGGCCTATCAGCTTGTTGGTGGGGTAACGGCCTACCAAGGCGATTACGGGTAGCCGGCCT
GAGAGGGCGACCGGCCACACTGGGACTGAGACACGGCCCAGACTCCTGCGGGAGGCAGCAGTGGGGAATA
TTGCGCAATGGGCGAAAGCCTGACGCAGCGACGCCGCGTGGGGGATGACGGCCTTCGGGTTGTAAACCTC
TTTTACCACCAACGCAGGCTTCCAGTTCTCTGGAGGTTGACGGTAGGTGGGGAATAAGGACCGGCTAACT
ACGTGCCAGCAGCCGCGGTAATACGTAGGGTCCGAGCGTTGTCCGGAATTATTGGGCGTAAAGAGCTCGT
AGGCGGCGTGTCGCGTCTGCTGTGAAAGACCGGGGCTTAACTCCGGTTCTGCAGTGGATACGGGCATGCT
AGAGGTAGGTAGGGGAGACTGGAATTCCTGGTGTAGCGGTGAAATGCGCAGATATCAGGAGGAACACCGG
TGGCGAAGGCGGGTCTCTGGGCCTTACCTGACGCTGAGGAGCGAAAGCATGGGGAGCGAACAGGATTAGA
TACCCTGGTAGTCCATGCCGTAAACGTTGGGCGCTAGGTGTGGGGACTTTCCACGGTTTCCGCGCCGTAG
CTAACGCATTAAGCGCCCCGCCTGGGGAGTACGGCCGCAAGGCTAAAACTCAAAGGAATTGACGGGGGCC
CGCACAAGCGGCGGAGCATGTTGCTTAATTCGACGCAACGCGAAGAACCTTACCAAGGTTTGACATCACC
CGTGGACTCGCAGAGATGTGAGGTCATTTAGTTGGCGGGTGACAGGTGGTGCATGGCTGTCGTCAGCTCG
TGTCGTGAGATGTTGGGTTAAGTCCCGCAACGAGCGCAACCCTTGTTCCATGTTGCCAGCACGTAATGGT
GGGGACTCATGGGAGACTGCCGGGGTCAACTCGGAGGAAGGTGGGGATGACGTCAAGTCATCATGCCCCT
TATGTCTTGGGCTGCAAACATGCTACAATGGCCGGTACAATGGGCGTGCGATACCGTAAGGTGGAGCGAA
TCCCTAAAAGCCGGTCTCAGTTCGGATTGGGGTCTGCAACTCGACCCCATGAAGGTGGAGTCGCTAGTAA
TCGCGGATCAGCAACGCCGCGGTGAATACGTTCCCGGGCCTTGTACACACCGCCCGTCACGTCATGAAAG
TCGGCAACACCCGAAACTTGCGGCCTAACCCCTTGTGGGAGGGAGTGAGTGAAGGTGGGGCTGGCGATTG
GGACGAAGTCGTAACAAGGTAGCCGTACCGGAAGGTGCGGCTGGATCACCT

>gb|CP002593.1|:577609-579116 Pseudonocardia dioxanivorans CB1190, complete genome
AGAGTTTGATCCTGGCTCAGGACGAACGCTGGCGGCGTGCTTAACACATGCAAGTCGAGCGGTAAGGCCT
TTCGGGGTACACGAGCGGCGAACGGGTGAGTAACACGTGGGTGACCTGCCCTCCACTCTGGGATAAGCCT
GGGAAACTGGGTCTAATACCGGATATGACCACGGATCGCATGGTGTGTGGTGGAAAGTTTTTTCGGTGGG
GGATGGGCCCGCGGCCTATCAGCTTGTTGGTGGGGTGATGGCCTACCAAGGCGACGACGGGTAGCCGGCC
TGAGAGGGCGACCGGCCACACTGGGACTGAGACACGGCCCAGACTCCTACGGGAGGCAGCAGTGGGGAAT
ATTGCGCAATGGGCGGAAGCCTGACGCAGCGACGCCGCGTGGGGGATGACGGCCTTCGGGTTGTAAACCT
CTTTCGCCAGGGACGAAGCGTGAGTGACGGTACCTGGATAAGAAGCACCGGCCAACTACGTGCCAGCAGC
CGCGGTAACACGTAGGGTGCGAGCGTTGTCCGGAATTATTGGGCGTAAAGAGCTCGTAGGCGGTCTGTCG
CGTCGGTCGTGAAAACCTGCAGCTTAACTGTGGGCTTGCGGTCGATACGGGCATGACTGGAGTTCGGCAG
GGGAGACTGGAATTCCTGGTGTAGCGGTGAAATGCGCAGATATCAGGAGGAACACCGGTGGCGAAGGCGG
GTCTCTGGGCCGATACTGACGCTGAGGAGCGAAAGCGTGGGGAGCGAACAGGATTAGATACCCTGGTAGT
CCACGCCGTAAACGTTGGGCGCTAGGTGTGGGGGCCATTCCACGGTCTCTGTGCCGCAGCTAACGCATTA
AGCGCCCCGCCTGGGGAGTACGGCCGCAAGGCTAAAACTCAAAGGAATTGACGGGGGCCCGCACAAGCGG
CGGAGCATGTGGATTAATTCGATGCAACGCGAAGAACCTTACCTGGGTTTGACATGCACCAGACATCCCT
AGAGATAGGGCTTCCCTTGTGGTTGGTGTGCAGGTGGTGCATGGCTGTCGTCAGCTCGTGTCGTGAGATG
TTGGGTTAAGTCCCGCAACGAGCGCAACCCTCGTTCCATGTTGCCAGCGGGTTATGCCGGGGACTCATGG
GAGACTGCCGGGGTCAACTCGGAGGAAGGTGGGGATGACGTCAAGTCATCATGCCCCTTATGTCCAGGGC
TTCACACATGCTACAATGGCAAGTACAGAGGGCTGCGAGACCGTGAGGTGGAGCGAATCCCTTAAAGCTT
GTCTCAGTTCGGATCGGGGTCTGCAACTCGACCCCGTGAAGTTGGAGTCGCTAGTAATCGCAGATCAGCA
ACGCTGCGGTGAATACGTTCCCGGGCCTTGTACACACCGCCCGTCACGTCACGAAAGTTGGTAACACCCG
AAGCCGACGGCCTAACCCCTTGTGGGAGGGAGTTGTCGAAGGTGGGACTGGCGATTGGGACGAAGTCGTA
ACAAGGTAGCCGTACCGGAAGGTGCGGCTGGATCACCT
>gi|418460477|ref|NZ_AHBX01000067.1| Saccharomonospora azurea SZMC 14600 contig67, whole genome shotgun sequence
TTGGAGAGTTTGATCCTGGCTCAGGACGAACGCTG
GCGGCGTGCTTAACACATGCAAGTCGAACGCTGAAGCCCAGCTTGCTGGGTGGATGAGTG
GCGAACGGGTGAGTAACACGTGGGTAATCTGCCCTGTACTCTGGGATAAGCCTGGGAAAC
TGGGTCTAATACCGGATAGGACACACTGCCGCATGGTGGTGTGTGGAAAGCTCCGGCGGT
ACAGGTTGAGCCCGCGGCCTATCAGCTTGTTGGTGGGGTGATGGCCTACCAAGGCGACGA
CGGGTAGCCGGCCTGAGAGGGTGACCGGCCACACTGGGACTGAGACACGGCCCAGACTCC
TACGGGAGGCAGCAGTGGGGAATATTGCACAATGGGCGCAAGCCTGATGCAGCGACGCCG
CGTGGGGGATGACGGCCTTCGGGTTGTAAACCCCTTTCGCCAGGGACGAAGCGTAAGTGA
CGGTACCTGGAGAAGAAGCACCGGCCAACTACGTGCCAGCAGCCGCGGTAATACGTAGGG
TGCAAGCGTTGTCCGGAATTATTGGGCGTAAAGAGCTCGTAGGCGGTGTGTCACGTCTGC
CGTGAAAACCTGCGGCTTAACCGTGGGCGTGCGGTGGATACGGGCATCACTTGAGTTCGG
TAGGGGAGACTGGAATTCCTGGTGTAGCGGTGGAATGCGCAGATATCAGGAGGAACACCG
GTGGCGAAGGCGGGTCTCTGGGCCGATACTGACGCTGAGGAGCGAAAGCGTGGGGAGCGA
ACAGGATTAGATACCCTGGTAGTCCACGCCGTAAACGTTGGGCGCTAGGTGTGGGGCGCT
GTTCACGTGTCCCGTGCCGTAGCTAACGCATTAAGCGCCCCGCCTGGGGAGTACGGCCGC
AAGGCTAAAACTCAAAGGAATTGACGGGGGCCCGCACAAGCGGCGGAGCATGTGGATTAA
TTCGATGCAACGCGAAGAACCTTACCTGGGCTTGACATGCACCGGATCGCCTCAGAGATG
GGGTTTCCCTTGTGGTCGGTGCACAGGTGGTGCATGGCTGTCGTCAGCTCGTGTCGTGAG
ATGTTGGGTTAAGTCCCGCAACGAGCGCAACCCTTGTCCCATGTTGCCAGCGGGTAATGC
CGGGGACTCGTGGGAGACTGCCGGGGTCAACTCGGAGGAAGGTGGGGATGACGTCAAGTC
ATCATGCCCCTTATGTCCAGGGCTTCACACATGCTACAATGGCTGGTACAGAGGGTTGCG
ATACCGTGAGGTGGAGCGAATCCCTTAAAGCCAGTCTCAGTTCGGATCGCAGTCTGCAAC
TCGACTGCGTGAAGTCGGAGTCGCTAGTAATCGCAGATCAGCATTGCTGCGGTGAATACG
TTCCCGGGCCTTGTACACACCGCCCGTCACGTCATGAAAGTCGGTAACACCCGAAGCCCA
TGGCCCAACCCGCTTGCGGGGGGGAGTGGTCGAAGGTGGGACTGGCGATTGGGACGAAGT
CGTAACAAGGTAGCCGTACCGGAAGGTGCGGCTGGATCACCTCCTTTCTAAGGA
>gb|CP001683.1|:564225-565751 Saccharomonospora viridis DSM 43017, complete genome
CATTGTTGGAGAGTTTGATCCTGGCTCAGGACGAACGCTGGCGGCGTGCTTAACACATGCAAGTCGAACG
CTGAAGCCGTCTTCGGGCGGTGGATGAGTGGCGAACGGGTGAGTAACACGTGGGTAATCTGCCCTGTACT
CTGGGATAAGCCTGGGAAACTGGGTCTAATACCGGATAGGACACGCTACCGCATGGTGGTGTGTGGAAAG
CTTCGGCGGTACAGGATGAGCCCGCGGCCTATCAGCTAGTTGGTGGGGTGATGGCCTACCAAGGCGACGA

CGGGTAGCCGGCCTGAGAGGGTGACCGGCCACACTGGGACTGAGACACGGCCCAGACTCCTACGGGAGGC
AGCAGTGGGGAATATTGCACAATGGGCGGAAGCCTGATGCAGCGACGCCGCGTGGGGGATGACGGCCTTC
GGGTTGTAAACCCCTTTCGCCCGGGACGAAGCGAGAGTGACGGTACCGGGAGAAGAAGCACCGGCCAACT
ACGTGCCAGCAGCCGCGGTAATACGTAGGGTGCAAGCGTTGTCCGGAATTATTGGGCGTAAAGAGCTCGT
AGGCGGTGTGTCGCGTCTGCCGTGAAAACCTGCGGCTTAACCGTGGGCGTGCGGTGGATACGGGCACACT
TGAGTTCGGTAGGGGAGACTGGAATTCCTGGTGTAGCGGTGGAATGCGCAGATATCAGGAGGAACACCAG
TGGCGAAGGCGGGTCTCTGGGCCGAAACTGACGCTGAGGAGCGAAAGCGTGGGGAGCGAACAGGATTAGA
TACCCTGGTAGTCCACGCCGTAAACGGTGGGCGCTAGGTGTGGGATGCTGTTCACGTGTCCCGTGCCGTA
GCTAACGCATTAAGCGCCCCGCCTGGGGAGTACGGCCGCAAGGCTAAAACTCAAAGGAATTGACGGGGGC
CCGCACAAGCGGCGGAGCATGTGGATTAATTCGATGCAACGCGAAGAACCTTACCTGGGTTTGACATGCA
CTGGACCGGCGTAGAGATACGCCTTCCCTTGTGGCTGGTGCACAGGTGGTGCATGGCTGTCGTCAGCTCG
TGTCGTGAGATGTTGGGTTAAGTCCCGCAACGAGCGCAACCCTTGTCCCATGTTGCCAGCGGGTAATGCC
GGGGACTCGTGGGAGACTGCCGGGGTCAACTCGGAGGAAGGTGGGGACGACGTCAAGTCATCATGCCCCT
TATGCCCAGGGCTTCACACATGCTACAATGGCCAGTACAGAGGGTTGCGAGACCGTGAGGTGGAGCGAAT
CCCTTAAAGCTGGTCTCAGTTCGGATCGTAGTCTGCAACTCGACTACGTGAAGTCGGAGTCGCTAGTAAT
CGCAGATCAGCAACGCTGCGGTGAATACGTTCCCGGGCCTTGTACACACCGCCCGTCACGTCATGAAAGT
CGGTAACACCCGAAGCCCATGGCCTAACCCCGTCAGGGGAGGGAGTGGTCGAAGGTGGGACCGGCGATTG
GGACGAAGTCGTAACAAGGTAGCCGTACCGGAAGGTGCGGCTGGATCACCTCCTTTC
>gi|133909243:1402795-1404188 Saccharopolyspora erythraea NRRL2338
complete genome
TTAACACATGCAAGTCGAACGCTGAAGCATCTTCGGGTGTGGATGAGTGGCGAACGGGTGAGTAACACGT
GGGTAATCTGCCCTGCACTCTGGGATAAGCCCGGGAAACTGGGTCTAATACCGGATAGGACACATGGCCG
CATGGTCTGTGTGTGGAAAGTTCCGGCGGTGCAGGATGAGCCCGCGGCCTATCAGCTTGTTGGTGGGGTG
ATGGCCTACCAAGGCGACGACGGGTAGCCGGCCTGAGAGGGTGACCGGCCACACTGGGACTGAGACACGG
CCCAGACTCCTACGGGAGGCAGCAGTGGGGAATCTTGCGCAATGGGCGAAAGCCTGACGCAGCAACGCCG
CGTGGGGGATGACGGCCTTCGGGTTGTAAACCTCTTTCGACATCGACGAAGCCTTCGGGTGACGGTAGGT
GTAGAAGAAGCACCGGCTAACTACGTGCCAGCAGCCGCGGTAATACGTAGGGTGCGAGCGTTGTCCGGAT
TTATTGGGCGTAAAGAGCTCGTAGGCGGTTTGTCGCGTCGTTCGTGAAAACTGGAGGCTTAACCTTCAGC
TTGCGGTCGATACGGGCAGACTTGAGTTCGGTAGGGGAGACTGGAATTCCTGGTGTAGCGGTGAAATGCG
CAGATATCAGGAGGAACACCGGTGGCGAAGGCGGGTCTCTGGGCCGATACTGACGCTGAGGAGCGAAAGC
GTGGGGAGCGAACAGGATTAGATACCCTGGTAGTCCACGCCGTAAACGTTGGGCGCTAGGTGTGGGGACT
GTTTCCACGGTTCCTGTGCCGTAGCTAACGCATTAAGCGCCCCGCCTGGGGAGTACGGCCGCAAGGCTAA
AACTCAAAGGAATTGACGGGGGCCCGCACAAGCGGCGGAGCATGTGGATTAATTCGATGCAACGCGAAGA
ACCTTACCTGGGTTTGACATGCACTAGACTGCCTCAGAGATGGGGTTTCCCTTGTGGTTGGTGTACAGGT
GGTGCATGGCTGTCGTCAGCTCGTGTCGTGAGATGTTGGGTTAAGTCCCGCAACGAGCGCAACCCTTGTC
CTGTGTTGCCAGCACGTAATGGTGGGGACTCGCGGGAGACTGCCGGGGTCAACTCGGAGGAAGGTGGGGA
TGACGTCAAGTCATCATGCCCCTTATGTCCAGGGCTTCACACATGCTACAATGGCCGGTACAGAGGGTTG
CGATGCCGTGAGGTGGAGCGAATCCCTTAAAGCTGGTCTCAGTTCGGATCGGGGTCTGCAACTCGACCCC
GTGAAGTCGGAGTCGCTAGTAATCGCAGATCAGCATTGCTGCGGTGAATACGTTCCCGGGCCTTGTACAC
ACCGCCCGTCACGTCATGAAAGTCGGTAACACCCGAAGCCCATGGCCCAACCCTTTGGGGGGGA
>gi|2289247|gb|AF002818.1| Saccharopolyspora spinosa 16S ribosomal RNA
gene, partial sequence
GCAAGTCGAACGCTGAAGCATCTTCGAGGTGTGGATGAGTGGCGAACGGGTGAGTAACACGTGGGTAATC
TGCCCTGCACTCTGGGATAAGCCTTGGAAACGGGGTCTAATACCGGATATGACACATTATCGCATGGTGG
TGTGTGGAAAGTTCTGGCGGTGCAGGATGAGTCCGCGGCCTATCAGCTTGTTGGTGGGGTGATGGCCTAC
CAAGGCGACGACGGGTAGCCGGCCTGAGAGGGTGACCGGCCACACTGGGACTGAGACACGGCCCAGACTC
CTACGGGAGGCAGCAGTGGGGAATCTTGCGCAATGGGCGAAAGCCTGACGCAGCAACGCCGCGTGGGGGA
TGACGGCTTCGGGTTGTAAACCTCTTTCGACATCGACGAAGCCTTCGGGTGACGGTAGGTGTAGAAGAAG
CACCGGCTAACTACGTGCCAGCAGCCGCGGTAATACGTAGGGTGCGAGCGTTCTCCGGATTTATTGGGCG
TAAAGAGCTCGTAGGCGGTTTGTCGCGTCGGCCGTGAAAACCTGCAGCTTAACTGTGGGCTTGCGGTCGA
TACGGGCAGACTTGAGTTCGGTAGGGGAGACTGGAATTCCTGGTGTAGCGGTGAAATGCGCAGATATCAG
GAGGAACACCGGTGGCGAAGGCGGGTCTCTGGGCCGATACTGACGCTGAGGAGCGAAAGCGTGGGGAGCG
AACAGGATTAGATACCCTGGTAGTCCACGCCGTAAACGTTGGGCGCTAGGTGTGGGGATGGGTTCCACTG
TTTCCGTGCCGTAGCTAACGCATTAAGCGCCCCGCCTGGGGAGTACGGCCGCAAGGCTAAAACTCAAAGG
AATTGACGGGGGCCCGCACAAGCGGCGGAGCATGTGGATTAATTCGATGCAACGCGAAGAACCTTACCTG
GGTTTGACATGCACTAGACAGCTCCAGAGATGGGGTTTCCCTTGTGGTTGGTGTACAGGTGGTGCATGGC
TGTCGTCAGCTCGTGTCGTGAGATGTTGGGTTAAGTCCCGCAACGAGCGCAACCCTTGCCCTATGTTGCC
AGCGGGTTATGCCGGGGACTCGTGGGGGACTGCCGGGGTCAACTCGGAGGAAGGTGGGGATGACGTCAAG

TCATCATGCCCCTTATGCCCAGGGCTTCACACATGCTACAATGGCTGGTACAGAGGGTGGCGATATCGTG
AGGTGGAGCGAATCCCTTAAAGCCGGTCTCAGTTCGGATCGGGGTCTGCAACTCGACCTCGTGAAGTCGG
AGTCGCTAGTAATCGCAGATCAGCATTGCTGCGGTGAATACGTTCCCGGGCCTTGTACACACCGCCCGTC
ACGTCATGAAAGTCGGTAACACCCGAAGCCCATGGCCCAACCCTTGTGGGGGGAGTGGTCGAAGGTGGGA
CTGGCGATTGGGACGAAGTCGTAACAAGGTAGCCGTACCGGAAGGTGCGGCTGGATCACCTCCTT
>gi|433601838:1012089-1013592 Saccharothrix espanaensis DSM 44229
complete genome
AGAGTTTGATCCTGGCTCAGGACGAACGCTGGCGGCGTGCTTAACACATGCAAGTCGAGCGGTAAGGCCC
TTCGGGGTACACGAGCGGCGAACGGGTGAGTAACACGTGGGTAACCTGCCCTGTACTCCGGGATAAGCCT
GGGAAACTAGGTCTAATACCGGATACGACCCCATGGGGCATCTTGTGGGGTGGAAAGTTCCGGCGGTATG
GGATGGACCCGCGGCCTATCAGCTTGTTGGTGGGGTGATGGCCTACCAAGGCGACGACGGGTAGCCGGCC
TGAGAGGGTGACCGGCCACACTGGGACTGAGACACGGCCCAGACTCCTACGGGAGGCAGCAGTGGGGAAT
ATTGCACAATGGGCGAAAGCCTGATGCAGCGACGCCGCGTGAGGGATGACGGCCTTCGGGTTGTAAACCT
CTTTCAGCAGGGACGAAGCGTGAGTGACGGTACCTGCAGAAGAAGCACCGGCTAACTACGTGCCAGCAGC
CGCGGTAATACGTAGGGTGCGAGCGTTGTCCGGAATTATTGGGCGTAAAGAGCTCGTAGGCGGTTTGTTG
CGTCGGCCGTGAAAACTTCACGCTTAACGTGGAGCCTGCGGTCGATACGGGCAGACTTGAGTTCGGCAGG
GGAGACTGGAATTCCTGGTGTAGCGGTGAAATGCGCAGATATCAGGAGGAACACCGGTGGCGAAGGCGGG
TCTCTGGGCCGATACTGACGCTGAGGAGCGAAAGCGTGGGGAGCGAACAGGATTAGATACCCTGGTAGTC
CACGCCGTAAACGGTGGGTGCTAGGTGTGGGGGGCTTCCACGTCCTCCGTGCCGCAGCTAACGCATTAAG
CACCCCGCCTGGGGAGTACGGCCGCAAGGCTAAAACTCAAAGGAATTGACGGGGGCCCGCACAAGCGGCG
GAGCATGTGGATTAATTCGATGCAACGCGAAGAACCTTACCTGGGCTTGACATGCACCGGAAACCTGCAG
AGATGTAGGCCTCTTCGGACTGGTGTACAGGTGGTGCATGGCTGTCGTCAGCTCGTGTCGTGAGATGTTG
GGTTAAGTCCCGCAACGAGCGCAACCCTCGTTCCATGTTGCCAGCGCGTTATGGCGGGGACTCATGGGAG
ACTGCCGGGGTCAACTCGGAGGAAGGTGGGGATGACGTCAAGTCATCATGCCCCTTATGTCCAGGGCTTC
ACACATGCTACAATGGCCGGTACAGAGGGCTGCTAAGCCGTGAGGTGGAGCGAATCCCACAAAGCCGGTC
TCAGTTCGGATCGGGGTCTGCAACTCGACCCCGTGAAGTCGGAGTCGCTAGTAATCGCAGATCAGCAACG
CTGCGGTGAATACGTTCCCGGGCCTTGTACACACCGCCCGTCACGTCACGAAAGTCGGTAACACCCGAAG
CCCGTGGCCCAACCCGTAAGGGGGGGAGCGGTCGAAGGTGGGACTGGCGATTGGGACGAAGTCGTAACAA
GGTAGCCGTACCGGAAGGTGCGGCTGGATCACCT
>gb|CP000850.1|:222623-224141 Salinispora arenicola CNS-205, complete
genome
TTTGTTGGAGAGTTTGATCCTGGCTCAGGACGAACGCTGGCGGCGTGCTTAACACATGCAAGTCGAGCGG
AAAGGCCCTTCGGGGTACTCGAGCGGCGAACGGGTGAGTAACACGTGAGTAACCTGCCCCAGGCTTTGGG
ATAACCCCGGGAAACCGGGGCTAATACCGGATATGACCATCTGTCGCATGGTGGGTGGTGGAAAGATTTT
TTGGCTTGGGATGGGCTCGCGGCCTATCAGCTTGTTGGTGGGGTGATGGCCTACCAAGGCGGCGACGGGT
AGCCGGCCTGAGAGGGCGACCGGCCACACTGGGACTGAGACACGGCCCAGACTCCTACGGGAGGCAGCAG
TGGGGAATCTTGCACAATGGGCGGAAGCCTGATGCAGCGACGCCGCGTGAGGGATGACGGCCTTCGGGTT
GTAAACCTCTTTCAGCAGGGACGAAGCGTTTGTGACGGTACCTGCAGAAGAAGCGCCGGCCAACTACGTG
CCAGCAGCCGCGGTAAGACGTAGGGCGCAAGCGTTGTCCGGATTTATTGGGCGTAAAGAGCTCGTAGGCG
GCTTGTCGCGTCGACTGTGAAAACCCGTGGCTCAACTGCGGGCTTGCAGTCGATACGGGCAGGCTAGAGT
TCGGTAGGGGAGACTGGAATTCCTGGTGTAGCGGTGAAATGCGCAGATATCAGGAGGAACACCGGTGGCG
AAGGCGGGTCTCTGGGCCGATACTGACGCTGAGGAGCGAAAGCGTGGGGAGCGAACAGGATTAGATACCC
TGGTAGTCCACGCTGTAAACGTTGGGCGCTAGGTGTGGGGGGCCTCTCCGGTTCTCTGTGCCGCAGCTAA
CGCATTAAGCGCCCCGCCTGGGGAGTACGGCCGCAAGGCTAAAACTCAAAGGAATTGACGGGGGCCCGCA
CAAGCGGCGGAGCATGCGGATTAATTCGATGCAACGCGAAGAACCTTACCTGGGTTTGACATCGCCGGAA
ATCCTTCAGAGATGGGGGGTCCTTCGGGGCCGGTGACAGGTGGTGCATGGCTGTCGTCAGCTCGTGTCGT
GAGATGTTGGGTTAAGTCCCGCAACGAGCGCAACCCTTGTTCGATGTTGCCAGCGCGTTATGGCGGGGAC
TCATCGAAGACTGCCGGGGTCAACTCGGAGGAAGGTGGGGATGACGTCAAGTCATCATGCCCCTTATGTC
CAGGGCTTCACGCATGCTACAATGGCCGGTACAGTGGGCTGCGATACCGTGAGGTGGAGCGAATCCCAAA
AAGCCGGTCTCAGTTCGGATCGGGGTCTGCAACTCGACCCCGTGAAGTCGGAGTCGCTAGTAATCGCAGA
TCAGCAACGCTGCGGTGAATACGTTCCCGGGCCTTGTACACACCGCCCGTCACGTCACGAAAGTCGGCAA
CACCCGAAGCCGGTGGCCTAACCCTTGTGGGGGGAGCCGTCGAAGGTGGGGCTGGCGATTGGGACGAAGT
CGTAACAAGGTAGCCGTACCGGAAGGTGCGGCTGGATCACCTCCTTTCT
>gi|343198502|ref|NR_042724.1| Salinispora tropica CNB-440 strain
CNB440 16S ribosomal RNA, partial sequence
AGAGTTTGATCCTGGCTCAGGACGAACGCTGGCGGCGTGCTTAACACATGCAAGTCGAGCGGAAAGGCCC
TTCGGGGTACTCGAGCGGCGAACGGGTGAGTAACACGTGAGTAACCTGCCCCAGGCTTTGGGATAACCCC
GGGAAACCGGGGCTAATACCGGATATGACTGGCTGCCGCATGGTGGTTGGTGGAAAGATTTTTTGGCTTG

GGATGGGCTCGCGGCCTATCAGCTTGTTGGTGGGGTGATGGCCTACCAAGGCGGCGACGGGTAGCCGGCC
TGAGAGGGCGACCGGCCACACTGGGACTGAGACACGGCCCAGACTCCTACGGGAGGCAGCAGTGGGGAAT
CTTGCACAATGGGCGGAAGCCTGATGCAGCGACGCCGCGTGAGGGATGACGGCCTTCGGGTTGTAAACCT
CTTTCAGCAGGGACGAAGCGTTTGTGACGGTACCTGCAGAAGAAGCGCCGGCCAACTACGTGCCAGCAGC
CGCGGTAAGACGTAGGGCGCAAGCGTTGTCCGGATTTATTGGGCGTAAAGAGCTCGTAGGCGGCTTGTCG
CGTCGACTGTGAAAACCCGTGGCTCAACTGCGGGCTTGCAGTCGATACGGGCAGGCTAGAGTTCGGTAGG
GGAGACTGGAATTCCTGGTGTAGCGGTGAAATGCGCAGATATCAGGAGGAACACCGGTGGCGAAGGCGGG
TCTCTGGGCCGATACTGACGCTGAGGAGCGAAAGCGTGGGGAGCGAACAGGATTAGATACCCTGGTAGTC
CACGCTGTAAACGTTGGGCGCTAGGTGTGGGGAGCCTCTCCGGTTCTCTGTGCCGCAGCTAACGCATTAA
GCGCCCCGCCTGGGGAGTACGGCCGCAAGGCTAAAACTCAAAGGAATTGACGGGGGCCCGCACAAGCGGC
GGAGCATGCGGATTAATTCGATGCAACGCGAAGAACCTTACCTGGGTTTGACATCGCCGGAAATCCTTCA
GAGATGGGGGGTCCTTCGGGGCCGGTGACAGGTGGTGCATGGCTGTCGTCAGCTCGTGTCGTGAGATGTT
GGGTTAAGTCCCGCAACGAGCGCAACCCTTGTTCGATGTTGCCAGCGCGTTATGGCGGGGACTCATCGAA
GACTGCCGGGGTCAACTCGGAGGAAGGTGGGGATGACGTCAAGTCATCATGCCCCTTATGTCCAGGGCTT
CACGCATGCTACAATGGCCGGTACAATGGGCTGCGATACCGTGAGGTGGAGCGAATCCCAAAAAGCCGGT
CTCAGTTCGGATCGGGGTCTGCAACTCGACCCCGTGAAGTCGGAGTCGCTAGTAATCGCAGATCAGCAAC
GCTGCGGTGAATACGTTCCCGGGCCTTGTACACACCGCCCGTCACGTCACGAAAGTCGGCAACACCCGAA
GCCGGTGGCCTAACCCTTGTGGGGGGAGCCGTCGAAGGTGGGGCTGGCGATTGGGACGAAGTCGTAACAA
GGTAGCCGTA
>gi|343202671|ref|NR_043072.1| Stackebrandtia nassauensis DSM 44728
strain NRRL B-16338 16S ribosomal RNA, partial sequence
TGACCTGGCTCAGGACGAACGCTGGCGGCGTGCTTCACACATGCAAGTCGAACGGAAAGGCCCTTTCGGG
GGTACTCGAGTGGCGAACGGGTGAGTAACACGTGAGTAATCTGCCTTTGGCTTTGGGATAAGCCTCGGAA
ACGGGGTCTAATACCGGATACGACATCTTCTCGCATGAGACGGGTGTGGAAAGTTTTTCGGCTGAAGATG
GGCTCGCGGTCTATCAGCTTGTTGGTGGGGTAATGGCTCACCAAGGCTTTGACGGGTAGCCGGCCTGAGA
GGGCGACCGGCCACACTGGGACTGAGACACGGCCCAGACTCCTACGGGAGGCAGCAGTGGGGAATATTGC
GCAATGGGCGAAAGCCTGACGCAGCGACGCCGCGTGAGGGATGACGGCCTTCGGGTTGTAAACCTCTTTC
GGTAGGGACGAAGTGAAAATGACGGTACCTGCAGAAGAAGCTCCGGCTAACTACGTGCCAGCAGCCGCGG
TAAGACGTAGGGAGCAAGCGTTGTCCGGATTTATTGGGCGTAAAGGGCTCGTAGGCGGCTTGTCACGTCT
GTCGTGAAAGATCAGGGCTTAACTCTGGTTTTGCGGTGGATACGGGCAGGCTTGAGGCAAGTAGGGGAGA
TCGGAATTCCATGTGTAGCGGTGAAATGCGCAGATATGTGGAGGAACACCGGTGGCGAAGGCGGATCTCT
GGGCTTGTTCTGACGCTGAGGAGCGAAAGCGTGGGGAGCGAACAGGATTAGATACCCTGGTAGTCCACGC
TGTAAACGGTGGGCGCTAGGTGTGGGGTCACATTTGTTGGTTCCGTGCCGTAGCTAACGCATTAAGCGCC
CCGCCTGGGGAGTACGGCCGCAAGGCTAAAACTCAAAGGAATTGACGGGGGCCCGCACAAGCGGCGGAGC
ATGCGGATTAATTCGATGCAACGCGAAGAACCTTACCTGGGTTTGACATCACCCGGAAACACCTGGAGAC
AGGTGCCTCTTCGGATCGGGTGACAGGTGGTGCATGGCTGTCGTCAGCTCGTGTCGTGAGATGTTGGGTT
AAGTCCCGCAACGAGCGCAACCCTTATCCTATGTTGCCAGCGCGTTATGGCGGGGACTCATGGGAGACTG
CCGGGGTCAACTCGGAGGAAGGTGGGGATGAGGTCAAGTCATCATGCCCCTTATGTCCAGGGCTTCACGC
ATGCTACAATGGCCGGTACAGAGGGCTGCGATACCGTGAGGTGGAGCGAATCTCGTAAAGCCGGTCTCAG
TTCGGATCCCAGTCTGCAACTCGACTGGGTGAAGTCGGAGTCGCTAGTAATCGCAGATCAGCAACGCTGC
GGTGAATACGTTCCCGGGCCTTGTACACACCGCCCGTCACGTCATGAAAGTCGGCAACACCCGAAGCCCG
TGGCCTAACCCCTCGTGGGATGGAGCGGTCGAAGGTGGGGCTGGCGATTGGGACGAAGTCGTAACAAGGT
AGCCGTACCGGAAGGTGCGGC
>gi|48948|emb|X60514.1| S.coelicolor rRNA 16S gene
GACGACCGCACCGCGCTCGGTGATGTGCTGACCCGCCTCCTGCGCAGGGTGGAGCACGCACAGCCGGACC
GCGGTGGCCGGCTCAGCGACCTCGATTAAAGATCTTGACAGCGACGCTTGACAGCCCTCTGCGGGATCCG
TAAGGTTCTTCGGGTTGCCACGGGACCGTAACGGTTCTTCGGCAGCACCTCCCGCCGCAGCAGCGAATCA
AACCACCAGCACGACCCCCAAGTGGGAATGACTTCGGCGCGCCCGAATTCAATTCCGCTGGGTACTCGTC
GGCCCGATCGACCCCGATTGGGAATCGCCGAGAGGGTCCGCTAAGGTTTGAGACGTCGGAACGGCCCAAC
AGCCGGGACGACAACCCCTCCTGACGGGGAATCAGGGCCCGAAAGGATCTGATAGAGTCGGAACCGCCGG
AAAGGGAAACGCGGAAGCGGGAACCTGGAAAGCACCGAGGAAATCGGATCGGAAAGATCTGATAGAGTCG
GAAACGCAAGACCGAAGGGAAGCGCCCGGAGGAAAGCCCACGAGAGTCTCCTGGGTGAGTACAAAGGAAG
CGTCCGTTCCTTGAGAACTCAACAGCGTGCCAAAAGTCAACGCCAGATATGTTGATACCCCGACCTGATC
GGATCTCCGTTCGGGTTGAGGTTCCTTTGAAGTAACACAACAGCGAGGACGCTGTGAACGGTCGGATTAT
TCCTCCGACTGTTCCGCTCTCGTGGTGTCACCCGATTACGGGTATACATTCACGGAGAGTTTGATCCTGG
CTCAGGACGAACGCTGGCGGCGTGCTTAACACATGCAAGTCGAACGATGAACCACTTCGGTGGGGATTAG
TGGCGAACGGGTGAGTAACACGTGGGCAATCTGCCCTTCACTCTGGGACAAGCCCTGGAAACGGGGTCTA
ATACCGGATACTGACCCTCGCAGGCATCTGCGAGGTTCGAAAGCTCCGGCGGTGAAGGATGAGCCCGCGG

CCTATCAGCTTGTTGGTGAGGTAATGGCTCACCAAGGCGACGACGGGTAGCCGGCCTGAGAGGGCGACCG
GCCACACTGGGACTGAGACACGGCCCAGACTCCTACGGGAGGCAGCAGTGGGGAATATTGCACAATGGGC
GAAAGCCTGATGCAGCGACGCCGCGTGAGGGATGACGGCCTTCGGGTTGTAAACCTCTTTCAGCAGGGAA
GAAGCGAAAGTGACGGTACCTGCAGAAGAAGCGCCGGCTAACTACGTGCCAGCAGCCGCGGTAATACGTA
GGGCGCAAGCGTTGTCCGGAATTATTGGGCGTAAAGAGCTCGTAGGCGGCTTGTCACGTCGGTTGTGAAA
GCCCGGGGCTTAACCCCGGGTCTGCAGTCGATACGGGCAGGCTAGAGTTCGGTAGGGGAGATCGGAATTC
CTGGTGTAGCGGTGAAATGCGCAGATATCAGGAGGAACACCGGTGGCGAAGGCGGATCTCTGGGCCGATA
CTGACGCTGAGGAGCGAAAGGCTGGGGAGCGAACAGGATTAGATACCCTGGTAGTCCACGCCGTAAACGG
TGGGCACTAGGTGTGGGCAACATTCCACGTTGTCCGTGCCGCAGCTAACGCATTAAGTGCCCCGCCTGGG
GAGTACGGCCGCAAGGCTAAAACTCAAAGGAATTGACGGGGCCCCGCACAAGCGGCGGAGCATGTGGCTT
AATTCGACGCAACGCGAAGAACCTTACCAAGGCTTGACATACACCGGAAAGCATCAGAGATGGTGCCCCC
CTTGTGGTCGGTGTACAGGTGGTGCATGGCTGTCGTCAGCTCGTGTCGTGAGATGTTGGGTTAAGTCCCG
CAACGAGCGCAACCCTTGTCCCGTGTTGCCAGCAAGCCCTTCGGGGTGTTGGGGACTCACGGGAGACCGC
CGGGGTCAACTCGGAGGAAGGTGGGGACGAAGGCAAGTCATCATGCCCCTTATGTCTTGGGCTGCACACG
TGCTACAATGGCCGGTACAATGAGCTGCGATACCGCAAGGTGGAGCGAATCTCAAAAAGCCGGTCTCAGT
TCGGATTGGGGTCTGCAACTCGACCCCATGAAGTCGGAGTCGCTAGTAATCGCAGATCAGCATTGCTGCG
GTGAATACGTTCCCGGGCCTTGTACACACCGCCCGTCACGTCACGAAAGTCGGTAACACCCGAAGCCGGT
GGCCCAACCCCTTGTGGGAGGGAGCTGTCGAAGGTGGGACTGGCGATTGGGACGAAGTCGTAACAAGGTA
GCCGTACCGGAAGGTGCGGCTGGATCACCTCCTTTCTAAGGAGCACATAGCCGACTGCAG
>Streptomyces griseoflavus Tu4000
CTGGCGGCGTGCTTAACACATGCAAGTCGAACGATGAACCACTTCGGTGGGGATTAGTGGCGAACGGGTG
AGTAACACGTGGGCAATCTGCCCTGCACTCTGGGACAAGCCCTGGAAACGGGGTCTAATACCGGATACTG
ACCCGCCTGGGCATCCAGGCGGTTCGAAAGCTCCGGCGGTGCAGGATGAGCCCGCGGCCTATCAGCTTGT
TGGTGAGGTAATGGCTCACCAAGGCGACGACGGGTAGCCGGCCTGAGAGGGCGACCGGCCACACTGGGAC
TGAGACACGGCCCAGACTCCTACGGGAGGCAGCAGTGGGGAATATTGCACAATGGGCGAAAGCCTGATGC
AGCGACGCCGCGTGAGGGATGACGGCCTTCGGGTTGTAAACCTCTTTCAGCAGGGAAGAAGCGAAAGTGA
CGGTACCTGCAGAAGAAGCGCCGGCTAACTACGTGCCAGCAGCCGCGGTAATACGTAGGGCGCGAGCGTT
GTCCGGAATTATTGGGCGTAAAGAGCTCGTAGGCGGCTTGTCACGTCGGTTGTGAAAGCCCGGGGCTTAA
CCCCGGGTCTGCAGTCGATACGGGCAGGCTAGAGTTCGGTAGGGGAGATCGGAATTCCTGGTGTAGCGGT
GAAATGCGCAGATATCAGGAGGAACACCGGTGGCGAAGGCGGATCTCTGGGCCGATACTGACGCTGAGGA
GCGAAAGCGTGGGGAGCGAACAGGATTAGATACCCTGGTAGTCCACGCCGTAAACGGTGGGCACTAGGTG
TGGGCGACATTCCACGTCGTCCGTGCCGCAGCTAACGCATTAAGTGCCCCGCCTGGGGAGTACGGCCGCA
AGGCTAAAACTCAAAGGAATTGACGGGGGCCCGCACAAGCGGCGGAGCATGTGGCTTAATTCGACGCAAC
GCGAAGAACCTTACCAAGGCTTGACATACACCGGAAAGCATTAGAGATAGTGCCCCCCTTGTGGTCGGTG
TACAGGTGGTGCATGGCTGTCGTCAGCTCGTGTCGTGAGATGTTGGGTTAAGTCCCGCAACGAGCGCAAC
CCTTGTCCCGTGTTGCCAGCAGGCCCTTGTGGTGCTGGGGACTCACGGGAGACCGCCGGGGTCAACTCGG
AGGAAGGTGGGGACGACGTCAAGTCATCATGCCCCTTATGTCTTGGGCTGCACACGTGCTACAATGGCCG
GTACAATGAGCTGCGATACCGCGAGGTGGAGCGAATCTCAAAAAGCCGGTCTCAGTTCGGATTGGGGTCT
GCAACTCGACCCCATGAAGTCGGAGTCGCTAGTAATCGCAGATCAGCATTGCTGCGGTGAATACGTTCCC
GGGCCTTGTACACACCGCCCGTCACGTCACGAAAGTCGGTAACACCCGAAGCCGGTGGCCCAACCCCTTG
TGGGAGGGAGCTGTCGAAGGTGGGACTGGCGATTGGGACGAAGTCGTAACAAGGTAGCCGTAC
>Streptomyces sp. AA4
GACGAACGCTGGCGGCGTGCTTAACACATGCAAGTCGAACGCTGAACCGGTTTCGGCCGGGGATGAGTGG
CGAACGGGTGAGTAACACGTGGGTAATCTGCCCTGTACTCTGGGATAAGCCTGGGAAACTGGGTCTAATA
CCGGATACGACCACTACAGGCATCTGTGGTGGTGGAAAGTTCCGGCGGTATGGGATGAACCCGCGGCCTA
TCAGCTTGTTGGTGGGGTAATGGCCTACCAAGGCGACGACGGGTAGCCGGCCTGAGAGGGTGACCGGCCA
CACTGGGACTGAGACACGGCCCATTTTTCCTACGGGGTGCAGCAGTGGGGAATATTGTTTCTATTGGGCG
CAAGCCTGATGCAGCGACGCCGCGTGAGGGATGACGGCCTTCGGGTTGTAAACCTCTTTCGCCAGGGACG
AAGCGCAAG
>gi|282153487|gb|GU225938.1| Streptomyces zinciresistens K42 16S
ribosomal RNA gene, partial sequence
GCACTTGCGCTGCTTACCATGCAAGTCGAACGATGAACCATTTCGGTGGGGATTAGTGGCGAACGGGTGA
GTAACACGTGGGCAATCTGCCCTTCACTCTGGGACAAGCCCTGGAAACGGGGTCTAATACCGGATAATAC
TTCTGCCTGCATGGGCGGGGGTTGAAAGCTCCGGCGGTGAAGGATGAGCCCGCGGCCTATCAGCTTGTTG
GTGAGGTAACGGCTCACCAAGGCGACGACGGGTAGCCGGCCTGAGAGGGCGACCGGCCACACTGGGACTG
AGACACGGCCCAGACTCCTACGGGAGGCAGCAGTGGGGAATATTGCACAATGGGCGGAAGCCTGATGCAG
CGACGCCGCGTGAGGGATGACGGCCTTCGGGTTGTAAACCTCTTTCAGCAGGGAAGAAGCGAGAGTGACG
GTACCTGCAGAAGAAGCGCCGGCTAACTACGTGCCAGCAGCCGCGGTAATACGTAGGGCGCAAGCGTTGT

```
CCGGAATTATTGGGCGTAAAGAGCTCGTAGGCGGCTTGTCGCGTCGGTTGTGAAAGCCCGGGGCTTAACC
CCGGGTCTGCAGTCGATACGGGCAGGCTAGAGTTCGGTAGGGGAGATCGGAATTCCTGGTGTAGCGGTGA
AATGCGCAGATATCAGGAGGAACACCCGGTGGCGAAGGCGGATCTCTGGGCCGATACTGACGCTGAGGAG
CGAAAGCGTGGGGAGCGAACAGGATTAGATACCCTGGTAGTCCACGCCGTAAACGGTGGGCACTAGGTGT
GGGCAACATTCCACGTTGTCCGTGCCGCAGCTAACGCATTAAGTGCCCCGCCTGGGGAGTACGGCCGCAA
GGCTAAAACTCAAAGGAATTGACGGGGGCCCGCACAAGCGGCGGAGCATGTGGCTTAATTCGACGCAACG
CGAAGAACCTTACCAAGGCTTGACATACACCGGAAAACCCTGGAGACAGGGTCCCCCTTGTGGTCGGTGT
ACAGGTGGTGCATGGCTGTCGTCAGCTCGTGTCGTGAGATGTTGGGTTAAGTCCCGCAACGAGCGCAACC
CTTGTCCCGTGTTGCCAGCAGGCCCTTGTGGTGCTGGGGACTCACGGGAGACCGCCGGGGTCAACTCGGA
GGAAGGTGGGGACGACGTCAAGTCATCATGCCCCTTATGTCTTGGGCTGCACACGTGCTACAATGGCCGG
TACAATGAGCTGCGATACCGCGAGGTGGAGCGAATCTCAAAAAGCCGGTCTCAGTTCGGATTGGGGTCTG
CAACTCGACCCCATGAAGTCGGAGTCGCTAGTAATCGCAGATCAGCATTGCTGCGGTGAATACGTTCCCG
GGCCTTGTACACACCGCCCGTCACGTCACGAAAGTCGGTAACACCCGAAGCCGGTGGCCCAACCCCTTGT
GGGAGGGAGCTGTCGAAGGTGGGACTGGCGATGGACGAAGTCGAACAAAGTGGTGAGGTG
>gi|1297280|gb|U48996.1|SRU48996 Streptosporangium roseum 16S rRNA gene
GCAAGTCGAGCGGAAAGGCCCTTCGGGGTACTCGAGCGGCGAACGGGTGAGTAACACGTGAGTAACCTGC
CCCTGACTCTGGGATAAGCCCGGGAAACTGGGTCTAATACCGGATACGACACCGTTCCCGATGGGATGGG
TGGTGGAAAGTTTTTTCGTTTGGGGATGGACTTGCGGCCTATCAGCTTGTTGGTGGGGTAACGGCCTACC
AAGGCGACGACGGGTAGCCGGCCTGAGAGGGCGACCGGCCACACTGGGACTGAGACACGGCCCAGACTCC
TACGGGAGGCAGCAGTGGGGAATATTGCGCAATGGGCGAAAGCCTGACGCAGCGACGCCGCGTGGGGGAT
GACGGCCTTCGGGTTGTAAACCTCTTTCAGCAGGGACGAAGTTGACGTGTACCTGCAGAAGAAGCGCCGG
CTAACTACGTGCCAGCAGCCGCGGTAATACGTAGGGCGCAAGCGTTGTCCGGAATTATTGGGCGTAAAGA
GCTCGTAGGTGGCTTGTCACGTCGGGTGTGAAAGCTTGGGGCTTAACTCCAGGTCTGCATTCGATACGGG
CTGGCTAGAGGTAGGTAGGGGAGAACGGAATTCCTGGTGTAGCGGTGAAATGCGCAGATATCAGGAGGAA
CACCGGTGGCGAAGGCGGTTCTCTGGGCCTTACCTGACGCTGAGGAGCGAAAGCGTGGGGAGCGAACAGG
ATTAGATACCCTGGTAGTCCACGCCGTAAACGTTGGGCGCTAGGTGTGGGGGACCTTCCACGGTTTCCGC
GCCGTAGCTAACGCATTAAGCGCCCCGCCTGGGGAGTACGGCCGCAAGGCTAAAACTCAAAGGAATTGAC
GGGGGCCGCACAAGCGGCGGAGCATGTTGCTTAATTCGACGCAACGCGAAGAACCTTACCAAGGCTTGAC
ATCGCCCGGAAACACTAGAGATGGGTGCCTCTTCGGATCGGGTGACAGGTGGTGCATGGCTGTCGTCAGC
TCGTGTCGTGAGATGTTGGGTTAAGTCCCGCAACGAGCGCAACCCTTGTTCCATGTTGCCAGCACGCTCC
TTCGGGGTGGTGGGGACTCATGGGAGACTGCCGGGGTCAACTCGGAGGAAGGTGGGGATGACGTCAAGTC
ATCATGCCCCTTATGTCTTGGGCTGCAAACATGCTACAATGGCCGGTACAGAGGGTTGCGATACCGCAAG
GTGGAGCGAATCCCAAAAAGCCGGTCTCAGTTCGGATTGGGGTCTGCAACTCGACCCCATGAAGTCGGAG
TCGCTAGTAATCGCAGATCAGCAACGCTGCGGTGAATACGTTCCCGGGCCTTGTACACACCGCCCGTCAC
GTCACGAAAGTCGGCAACACCCGAAGCCCGTGGCCCAACCTTTGGGGGGGGAGCGGTCGAAGGTGGGGCT
GGCGATTGGGACG
>gb|CP000088.1|:2390559-2392029 Thermobifida fusca YX, complete genome
ACGAACGCTGGCGGCGTGCTTAACACATGCAAGTCGAGCGGTAAGGCCCCTTCGGGGGTACACGAGCGGC
GAACGGGTGAGTAACACGTGAGTAACCTGCCCCTGACTCTGGGATAAGCCTGGGAAACTGGGTCTAATAC
CGGATATGACCTCGTGCCGCATGGTGTGGGGTGGAAAGGTGTTTTTCCGGTCAGGGATGGGCTCGCGGCC
TATCAGCTTGTTGGTGGGGTAACGGCCTACCAAGGCGATGACGGGTAGCCGGCCTGAGAGGGCGACCGGC
CACACTGGGACTGAGACACGGCCCAGACTCCTGCGGGAGGCAGCAGTGGGGAATATTGCGCAATGGGCGG
AAGCCTGACGCAGCGACGCCGCGTGGGGGATGAAGGCCTTCGGGTTGTAAACCTCTTTTACCACTCACGA
AGGCCCCTGGTTTTCTGGGGGTTGACGGTAGGTGGTGAATAAGGACCGGCTAACTACGTGCCAGCAGCCG
CGGTAATACGTAGGGTCCGAGCGTTGTCCGGAATTATTGGGCGTAAAGAGCTCGTAGGCGGCCTGTCGCG
TCTGCTGTGAAAGTCCGGGGCTTAACTCCGGGTGTGCAGTGGATACGGGCAGGCTTGAGGTAGGTAGGGG
AGACTGGAATTCCTGGTGTAGCGGTGAAATGCGCAGATATCAGGAGGAACACCGGTGGCGAAGGCGGGTC
TCTGGGCCTTACCTGACGCTGAGGAGCGAAAGCGTGGGGAGCGAACAGGATTAGATACCCTGGTAGTCCA
CGCCGTAAACGTTGGGCGCTAGGTGTGGGGACTTTCCACGGTTTCCGTGCCGTAGCTAACGCATTAAGCG
CCCCGCCTGGGGAGTACGGCCGCAAGGCTAAAACTCAAAGGAATTGACGGGGGCCCGCACAAGCGGCGGA
GCATGTTGCTTAATTCGACGCAACGCGAAGAACCTTACCAAGGCTTGACATCACCGGTAATCCTCCAGAG
ATGGGGGGTCCTTCGGGGATCGGTGACAGGTGGTGCATGGCTGTCGTCAGCTCGTGTCGTGAGATGTTGG
GTTAAGTCCCGCAACGAGCGCAACCCCTGTCCCATGTTGCCAGCACGTGGTGGTGGGGACTCATGGGAGA
CTGCCGGGGTCAACTCGGAGGAAGGTGGGGATGACGTCAAGTCATCATGCCCCTTATGCCTTGGGCTGCA
AACATGCTACAATGGCCGGTACAGAGGGCGTGCGATGTCGTGAGGCGGAGCGAATCTCTTAAAGCCGGTC
TCAGTTCGGATTGGGGTCTGCAACTCGACCCCATGAAGGTGGAGTCGCTAGTAATCGCGGATCAGCAACG
CCGCGGTGAATACGTTCCCGGGCCTTGTACACACCGCCCGTCACGTCACGAAAGTCGGCAACACCCGAAA
CACGTGGCCTAACCCTTTGTGGGGGGGAGCGTGTGAAGGTGGGGCTGGCGATTGGGACGAAGTCGTAACA
```

A
>gb|CP001874.1|:443556-445080 Thermobispora bispora DSM 43833, complete genome
AGAGTTTGATCCTGGCTCAGGACGAACGCTGGCGGCGTGCTTAACACATGCAAGTCGAGCGAGGACCGCA
CCTTCGGGTGTGGAGCCGAGCGGCGAACGGGTGAGTAACACGTGAGCAACCTGCCCCTGGCACCGGGATA
GCCCCGGGAAACTGGGATTAATACCGGATACGACCACTTCCCGCATGGGATGGTGGTGGAAAGGTTCCCC
CTTGTGGGGTTCCGGCCAGGGATGGGCTCGCGGCCCATCAGCTAGTTGGCGGGGTAACGGCCCACCAAGG
CGATGACGGGTAGCCGGCCTGAGAGGGCGGTCGGCCACACTGGGACTGAGACACGGCCCAGACTCCTACG
GGAGGCAGCAGTGGGGAATATTGCGCAATGGGCGGAAGCCTGACGCAGCGACGCCGCGTGGGGGATGAAG
GCCTTCGGGTTGTAAACCTCTTTCAGCAGGGACGAAGTTGACGTGTACCTGCAGAAGAAGCGCCGGCCAA
CTACGTGCCAGCAGCCGCGGTAATACGTAGGGCGCGAGCGTTGTCCGGAGTTACTGGGCGTAAAGGGCTC
GTAGGCGGCTTGTCGCGTCGGCTGTGAAAGCCCACGGCTTAACCGTGGAATTGCAGTCGATACGGGCAGG
CTAGAGGGCGGCAGAGGGGAGTGGAATTCCCGGTGTAGCGGTGAAATGCGCAGATATCGGGAGGAACACC
GGTGGCGAAGGCGGCTCCCTGGGCCGTCCCTGACGCTGAGGAGCGAAAGCGTGGGGAGCGAACAGGATTA
GATACCCTGGTAGTCCACGCCGTAAACGTTGGGCGCTAGGTGTGGGGATCTTTTGTGATCTCCGTGCCGT
AGCTAACGCATTAAGCGCCCCGCCTGGGGAGTACGGCCGCAAGGCTAAAACTCAAAGGAACTGACGGGGG
CCCGCACAAGCGGCGGAGCATGTTGCTTAATTCGATGCAACGCGAAGAACCTTACCAGGGCTTGACATGC
TGGGAAATCCCGCAGAGATGCGGGGTGCCTTCGGGCGTCCAGCACAGGTGGTGCATGGCTGTCGTCAGCT
CGTGTCGTGAGATGTTGGGTTAAGTCCCGCAACGAGCGCAACCCCCACCCTATGTTGCCAGCGGGTTATG
CCGGGCACTCATGGGGAACTGCCGGGGTCAACCCGGAGGAAGGCGGGGACGACGTCAAGTCATCATGCCC
CTTATGCCCTGGGCTGCAAACATGCTACAATGGCCGGTACAATGGGCTGCGATCCCGTGAGGGTGAGCGA
ATCCCCAAAGCCGGTCCCAGTTCGGATCGGGGTCTGCAACTCGACCCCGTGAAGTCGGAGTCGCTAGTAA
TCGCGGATCAGCAATGCCGCGGTGAATACGTTCCCGGGCTTTGTACACACCGCCCGTCACGTCCTGAAAG
TCGGCAACACCCGAAGCCGGTGGCCTAACCCCCTTTTTGGGGGAGGGAGCCGTCGAAGGTGGGGCTGGTG
ATTGGGACGAAGTCGTAACAAGGTAGCCGTACCGGAAGGTGCGGCTGGATCACCT
>gb|CP001738.1|:898387-899905 Thermomonospora curvata DSM 43183, complete genome
AGAGTTTGATCCTGGCTCAGGACGAACGCTGGCGGCGTGCTTAACACATGCAAGTCGAGCGGAAAGGCCC
CTTCGGGGGTACTCGAGCGGCGAACGGGTGAGTAACACGTGAGTAACCTGCCCTCGACTCTGGGATAAGC
CTGGGAAACTGGGTCTAATACCGGATATTCACCTTCTGTCGCATGGTGGGGGGTGGAAAGTTCCCTCTTT
TTGGGGGTTCGGTCGGGGATGGGCTCGCGGCCTATCAGCTTGTTGGTGGGGTGACGGCCTACCAAGGCGA
CGACGGGTAGCCGGCCTGAGAGGGCGACCGGCCACACTGGGACTGAGACACGGCCCAGACTCCTACGGGA
GGCAGCAGTGGGGAATCTTGCGCAATGGGCGAAAGCCTGACGCAGCGACGCCGCGTGGGGGATGAAGGCC
TTCGGGTTGTAAACCCCTTTCAGCACCGACGAAGTTTGGACGGTAGGTGCAGAAGAAGCGCCGGCTAACT
ACGTGCCAGCAGCCGCGGTAATACGTAGGGCGCGAGCGTTGTCCGGAATTATTGGGCGTAAAGAGCTCGT
AGGCGGCTTGTCGCGTCTGTCGTGAAAGCCCACGGCTTAACCGTGGGTTTGCGGTGGATACGGGCAGGCT
AGAGGCAGGTAGGGGAGCATGGAATTCCCGGTGTAGCGGTGAAATGCGCAGATATCGGGAGGAACACCGG
TGGCGAAGGCGGTGCTCTGGGCCTGTTCTGACGCTGAGGAGCGAAAGCGTGGGGAGCGAACAGGATTAGA
TACCCTGGTAGTCCACGCCGTAAACGTTGGGCGCTAGGTGTGGGGTTCTTCCACGGATTCCGCGCCGTAG
CTAACGCATTAAGCGCCCCGCCTGGGGAGTACGGCCGCAAGGCTAAAACTCAAAGGAATTGACGGGGGCC
CGCACAAGCGGCGGAGCATGTTGCTTAATTCGACGCAACGCGAAGAACCTTACCAAGGCTTGACATCACC
CGAAAACCTGCAGAGATGCAGGGTCCCTTCGGGGGCGGGTGACAGGTGGTGCATGGCTGTCGTCAGCTCG
TGTCGTGAGATGTTGGGTTAAGTCCCGCAACGAGCGCAACCCTCGTTCCATGTTGCCAGCGGGTTATGCC
GGGGACTCATGGAAGACCGCCGGGGTCAACTCGGAGGAAGGTGGGGACGACGTCAAGTCATCATGCCCCT
TATGTCTTGGGCTGCAAACATGCTACAATGGCCGGTACAGAGGGCTGCGATACCGTGAGGTGGAGCGAAT
CCCTAAAAGCCGGTCTCAGTTCGGATCGAAGTCTGCAACTCGACTTCGTGAAGTCGGAGTCGCTAGTAAT
CGCAGATCAGCAACGCTGCGGTGAATACGTTCCCGGGCCTTGTACACACCGCCCGTCACGTCACGAAAGT
CGGCAACACCCGAAGCCCGTGGCCTAACCCGTAAGGGGGGGAGCGGTCGAAGGTGGGGCTGGCGATTGGG
ACGAAGTCGTAACAAGGTAGCCGTACCGGAAGGTGCGGCTGGATCACCT
>gb|CP002638.1|:2336703-2338222 Verrucosispora maris AB-18-032, complete genome
TTGGAGAGTTTGATCCTGGCTCAGGACGAACGCTGGCGGCGTGCTTAACACATGCAAGTCGAGCGGAAAG
GCCTTTCGGGGTACTCGAGCGGCGAACGGGTGAGTAACACGTGAGCAACCTGCCCTAGGCTTTGGGATAA
CCCTCGGAAACGGGGGCTAATACCGAATATTCACTCATGGGCGCATGTTTGTGGGTGGAAAGTTTTTCGG
CTTGGGATGGGCTCGCGGCCTATCAGCTTGTTGGTGGGGTAATGGCCTACCAAGGCGACGACGGGTAGCC
GGCCTGAGAGGGCGACCGGCCACACTGGGACTGAGACACGGCCCAGACTCCTACGGGAGGCAGCAGTGGG
GAATATTGCACAATGGGCGGAAGCCTGATGCAGCGACGCCGCGTGAGGGATGACGGCCTTCGGGTTGTAA
ACCTCTTTCAGCAGGGACGAAGCGCAAGTGACGGTACCTGCAGAAGAAGCGCCGGCCAACTACGTGCCAG

CAGCCGCGGTAAGACGTAGGGCGCGAGCGTTGTCCGGATTTATTGGGCGTAAAGAGCTCGTAGGCGGCTT
GTCGCGTCGACTGTGAAAACCCGTGGCTCAACTGCGGGCCTGCAGTCGATACGGGCAGGCTAGAGTTCGG
TAGGGGAGACTGGAATTCCTGGTGTAGCGGTGAAATGCGCAGATATCAGGAGGAACACCGGTGGCGAAGG
CGGGTCTCTGGGCCGATACTGACGCTGAGGAGCGAAAGCGTGGGGAGCGAACAGGATTAGATACCCTGGT
AGTCCACGCTGTAAACGTTGGGCGCTAGGTGTGGGGGGCCTCTCCGGTTCTCTGTGCCGCAGCTAACGCA
TTAAGCGCCCCGCCTGGGGAGTACGGCCGCAAGGCTAAAACTCAAAGGAATTGACGGGGGCCCGCACAAG
CGGCGGAGCATGCGGATTAATTCGATGCAACGCGAAGAACCTTACCTGGGTTTGACATCGCCGGAAATCC
TGCAGAGATGTGGGGTCCTTCGGGGCCGGTGGCAGGTGGTGCATGGCTGTCGTCAGCTCGTGTCGTGAGA
TGTTGGGTTAAGTCCCGCAACGAGCGCAACCCTCGTCCCATGTTGCCAGCAATTCGGTTGGGGACTCATG
GGAGACTGCCGGGGTCAACTCGGAGGAAGGTGGGGATGACGTCAAGTCATCATGCCCCTTATGTCCAGGG
CTTCACGCATGCTACAATGGCCGGTACAATGGGCTGCGATACCGTGAGGTGGAGCGAATCCCAAAAAGCC
GGTCTCAGTTCGGATCGGGGTCTGCAACTCGACCCCGTGAAGTCGGAGTCGCTAGTAATCGCAGATCAGC
AACGCTGCGGTGAATACGTTCCCGGGCCTTGTACACACCGCCCGTCACGTCACGAAAGTCGGCAACACCC
GAAGCCGGTGGCCCAACCCTTGTGGAGGGAGCCGTCGAAGGTGGGGCTGGCGATTGGGACGAAGTCGTAA
CAAGGTAGCCGTACCGGAAGGTGCGGCTGGATCACCTCCTTTCTAAGGAG

Supplemental Data file 2. RpoB protein sequences from actinomycetes used as input for Figure 2.

```
>gi|117927508|ref|YP_872059.1| DNA-directed RNA polymerase subunit beta
[Acidothermus cellulolyticus 11B]
MVDREPWKEPHLAASGSTSMINGPLAPHRVSFAKIHEPLDLPNLLALQIESFDWLVGNDAWKARVEAALA
AGRTDVNTSSGLEEIFEEISPIEDISGTMSLSFRDHRFEPPKNTPDECRERDFTYSAPLFVTAEFVNNTT
GEIKSQTVFMGDFPLMTEKGTFIINGTERVVVSQLVRSPGVYFDRTVDKTSDRDIYGCKIIPSRGAWLEL
EIDKRDNVGVRIDRKRKQSVTVLLKALGWDDARILERFGEFEAIRATLEKDHVHTQEEALLDIYRKLRPG
EPPTYEAAQTLLNNLYFNPKRYDLAKVGRYKVNKKLGLDLPYTQNTLTEEDIVATIDYLVRLHAGQTETT
RNGRTVIIETDDIDHFGNRRLRTVGELIQNQIRLGLARMERVVRERMTTQDIVAITPQTLINIRPVVASI
KEFFGTSQLSQFMDQTNPLAGLTHKRRLNALGPGGLSRERAGFEVRDVHPSHYGRMCPIETPEGPNIGLI
GSLATHGRVNAFGFVETPYRKVINGRVTDQVDYLTADEEDRHVIAQANAPLNEDGSFAEERVLVRRRGGE
VDYVHPSTVDYMDVSPRQMVSVATALIPFLEHDDANRALMGSNMQRQAVPLLRAEAPLVGTGMEFRAAVD
AGDVVIASKAGVVEEVSADAITIAADDGTRQTYRLAKFRRSNQGTCINQKPIVDEGQRVEAGQPIADGPC
TQGGELALGRNLLVAFMSWEGHNYEDAIIISERLVKEDILSSIHIEEHEVDARDTKLGPEEITRDIPNVS
EEILADLDERGIIRIGADVVPGDILVGKVTPKGETELTPEERLLRAIFGEKAREVRDTSLKVPHGESGKV
IDVKVFTRDDAELPPGVNELVRVYVAQKRKITDGDKLAGRHGNKGVIAKILPEEDMPFLEDGTPVDIILN
PLGVPGRMNVGQVLETHLGWAAKSGWKVELNGQPEGWRKRLADIGALDAPPNTLVATPVFDGAKEDEIRG
LLENALPNSDGLRMVGPDGKARLFDGRTGEPYPEPISVGYIYIMKLHHLVDDKLHARSTGPYSMITQQPL
GGKAQFGGQRFGEMEVWALEAYGAAYALQELLTIKSDDVLGRVKVYEAIVKGENIPEPGIPESFKVLVKE
MQALCLNVEVLSSDGMTVEMRETDDEVFRAAEELGIDLSRREPSSVEEI
>gi|383775764|ref|YP_005460330.1| rpoB gene product [Actinoplanes
missouriensis 431]
MAASRPAKTSRTSSAYAPRRVSFGRITEQLEVPNLLALQTDSFDWLVGNEAWQARTTDDPHAHSGLAEIL
EEISPIEDFSGTMSLSFSAPRFDEVKASIEECKEKDLTYCAPLFVTAEFTNNTTGEIKSQTVFMGDFPMM
TPKGTFVINGTERVVVSQLVRSPGVYFTKEPDKTSDRDLTSVKVIPSRGAWLEFDIDKRDTVGVRIDRKR
RQAVTVLLKAIGWSADQIRERFGWSELLMTTLEKDHIAGQDEALLDIYRKLRPGEPPTRENAQTLLDNLF
FNPKRYDVAKVGRYKFNKKLEIDVPIVRGTLSEEDIVKTVDYLCRLHAGEEGYEADDIDHFGNRRLRTVG
ELIQNQVRVGLSRMERVVRERMTTQDVEAITPQTLINIRPVVAAIKEFFGTSQLSQFMDQTNPLAGLTHR
RRLSALGPGGLSRERAGFEVRDVHPSHYGRMCPIETPEGPNIGLIGALSTFARVNPFGFIETPYRKVENG
VVTDEVHYLTADEEDRFIKAQANAVLSSDNTFAEDRVLVRRKGGEVDYVPGGEVDYMDVSPRQMTSVATA
MIPFLEHDDANRALMGANMQRQAVPLVKAESPLVGTGMEYRAAVDAGDVVVAEVGGVVEDLCADYVTVHQ
DDGHRRTYLLHKFRRSNAGSCVNQKPVVFEGDRVEAGQVIADGPCTDEGEMALGRNLLVAFMCWEGHNYE
DAIILSQRLVQQDVLTSIHIEEHEVDARDTKLGPEEITRDIPNVSEEMLADLDERGIIRIGAEVVPGDIL
VGKVTPKGETELTPEERLLRAIFGEKAREVRDTSLKVPHGETGTVIGVRTFSREDGDELPPGVNELVRVY
VAQKRKIQDGDKLAGRHGNKGVISKILPVEDMPFLEDGTPVDIVLNPLGVPSRMNIGQVLETHLGWIAKT
GWSVEGEDADWKRQLRAIEAHESPADSNVATPVFDGAQEEEIKGLLESTLVNRDGKRLVNGDGKAQLFDG
RSGEPLPDPISVGYVYILKLNHLVDDKIHARSTGPYSMITQQPLGGKAQFGGQRFGEMECWAMQAYGAAY
ALQELLTIKSDDVLGRVKVYEAIVKGENIPEPGIPESFKVLLKELQSLCLNVEVLSSDGVALEMRETDDE
VFRAAEELGIDLSRRPNEGVSSVEEI
>gi|386845725|ref|YP_006263738.1| DNA-directed RNA polymerase subunit
beta [Actinoplanes sp. SE50/110]
MAASRPAKTSRTSSAYAPRRVSFGRITEQLEVPNLLALQTDSFDWLVGNEAWQARTTDDPHAHSGLAEIL
EEISPIEDFSGTMSLSFSAPRFDEVKASIEECKEKDLTYCAPLFVTAEFTNNTTGEIKSQTVFMGDFPMM
TPKGTFVINGTERVVVSQLVRSPGVYFTKEPDKTSDRDLTSVKVIPSRGAWLEFDIDKRDTVGVRIDRKR
RQAVTVLLKAIGWSADQIRERFGWSELLMTTLEKDHIAGQDEALLDIYRKLRPGEPPTRENAQTLLDNLF
FNPKRYDVAKVGRYKFNKKLEIDVPIVRGTLSEEDIVKTVEYLCRLHAGEEGYEADDIDHFGNRRLRTVG
ELIQNQVRVGLSRMERVVRERMTTQDVEAITPQTLINIRPVVAAIKEFFGTSQLSQFMDQTNPLAGLTHR
RRLSALGPGGLSRERAGFEVRDVHPSHYGRMCPIETPEGPNIGLIGALSTFARVNPFGFIETPYRKVENG
VVTDEVHYLTADEEDRYIKAQANAVLSSDNTFAEDRVLVRRKGGEVDYVPGTEVDYMDVSPRQMTSVATA
MIPFLEHDDANRALMGANMQRQAVPLVKAESPLVGTGMEYRAAVDAGDVVVAEVAGVVEDLCADYVTVHQ
DDGHRRTYLLHKFRRSNAGSCVNQKPVVFEGDRVEAGQVIADGPCTDEGEMALGRNLLVAFMCWEGHNYE
DAIILSQRLVQQDVLTSIHIEEHEVDARDTKLGPEEITRDIPNVSEEMLADLDERGIIRIGAEVVPGDIL
VGKVTPKGETELTPEERLLRAIFGEKAREVRDTSLKVPHGETGTVIGVRTFSREDGDELPPGVNELVRVY
VAQKRKIQDGDKLAGRHGNKGVISKILPVEDMPFLEDGTPVDIVLNPLGVPSRMNIGQVLETHLGWIAKT
```

GWSIEGDDEEWKRQLRSIEAHESPADSNVATPVFDGAQEEEIKGLLESTLVNRDGKRLVNGDGKAQLFDG
RSGEPLPDPISVGYVYILKLNHLVDDKIHARSTGPYSMITQQPLGGKAQFGGQRFGEMECWAMQAYGAAY
ALQELLTIKSDDVLGRVKVYEAIVKGENIPEPGIPESFKVLLKELQSLCLNVEVLSSDGVALEMRETDDE
VFRAAEELGIDLSRRPNEGVSSVEEI
>gi|256380610|ref|YP_003104270.1| DNA-directed RNA polymerase subunit beta [Actinosynnema mirum DSM 43827]
MAISRATKATAATNSTSGIPGAPKRVSFAKIHEPLQTPNLLDLQIQSFEWLTGDEAWFQRRVDEGDEAPT
GGLEEVLNEISPIEDFSGSMSLSFSDPRFDEVKASTEECKDKDMTYAAPLFVTAEFTNHTTGEIKSQTVF
MGDFPMMTDKGTFIINGTERVVVSQLVRSPGVYYDTAIDKTTDKDVFSVKIIPSRGAWLEFDVDKRDTVG
VRIDRKRRQPVTVLLKALGWSTEQIRERFSFSETLLTTLEKDHTAGTDEALLDIYRKLRPGEPPTKESAQ
ALLETLFFKEKRYDLAKVGRYKVNKKLGLDTPISTGVLTEDDIVTTIEYLVRLHAGETSMQPGETEVPVE
VDDIDHFGNRRLRTVGELIQNQIRVGLSRMERVVRERMTTQDVEAITPQTLINIRPVVAAIKEFFGTSQL
SQFMDQTNPLAGLTHKRRLSALGPGGLSRERAGMEVRDVHPSHYGRMCPIETPEGPNIGLIGSLSSYGRV
NPFGFIETPYRKVVDGRVTDQIDYLTADEEDRYVKAQANARIDGEGNFLEGKVLVRKKGGEVEMIDPRDV
DYMDVSPRQMVSAATAMIPFLEHDDANRALMGANMQRQAVPLLRSESPLVGTGMELRAAVDAGDVVVAKK
TGVVEEISADYVTVMADDGSRQTYGMHKFRRSNQGTCINQKPIVNEGDQVKVGQVLADGPCTENGEMALG
KNLLVAIMPWEGHNYEDAIILSQRLVQDDVLTSIHIEEHEIDARDTKLGAEEITRDIPNVSEEVLADLDE
RGIIRIGAEVQPGDILVGKVTPKGETELTPEERLLRAIFGEKAREVRDTSLKVPHGEYGKVIGIRVFSRE
DDDELPPGVNELVRVYVAQKRKIQDGDKLAGRHGNKGVIGKILPVEDMPFLEDGTPVDIVLNTHGVPRRM
NIGQVLETHLGWIAKQGWSINGDPDWAKNLPADLYEVDPGTKTATPVFDGAREEEITGLLGSTVPNRDGE
RMVKENGKAQLFDGRSGEPYPFPVSVGYMYILKLLHLVDDKIHARSTGPYSMITQQPLGGKAQFGGQRFG
EMECWAMQAYGAAYTLQELLTIKSDDVLGRVKVYEAIVKGENIPEPGIPESFKVLLKELQSLCLNVEVLS
SDGAAIEMRDGDDEDLERAAANLGINLSRSESPSVDDVVN
>gi|399534473|ref|YP_006547135.1| DNA-directed RNA polymerase subunit beta [Amycolatopsis mediterranei S699]
MAVSPANQATAATTSAESRSEATGIPGAPKRVSFAKIREPLNTPNLLDVQIQSFQWFTGDEAWFQRRVEE
GEENPVGGLEEVLNEISPIEDFSGSMSLSFSAPRFDEVKASIEECKDKDMTYAAPLFVTAEFVNNNTGEI
KSQTVFLGDFPVMTDKGTFVINGTERVVVSQLVRSPGVYYSKDIDKTSDKDVFSVRVIPSRGAWLEFDVD
KRDTVGVRIDRKRRQPVTVLLKALGWTTEAIRERFSFSETLLATLEKDHTAGTDEALLDIYRKLRPGEPP
TKESAQTLLENLFFKAKRYDLAKVGRYKVNKKLGLSTPIENGTLTEEDIVTIIEYLVRLHAGEDKMTAAN
NTEIPVETDDIDHFGNRRIRTVGELIQNQIRVGLSRTERVVRERMTTQDVEAITPQTLINIRPIGAAIKE
FFGTSQLSQFMQQTNPIDGLTHKRRLNALGPGGLSRERAGMEVRDVHPSHYGRMCPIETPEGPNIGLIGS
LCSYARVNPFGFIETPYRKVVEGRVTDQIDYLTADEEDRFVKAQANAPISDDGTFIEDRVMARRKGGEVE
LIDPLDIDYMDVSPRQMVSIATAMIPFLEHDDANRALMGANMQRQAVPLLRSQAPYVGTGVELRAAIDSG
DMLVAEQSGVVEELSADLITVMHDDGTRKSYSLYKFRRSNHGTCFNHRPIVNEGDRIEAGQVIADGPSTE
NGEVALGKNLLVAVMPWEGHNYEDAIILSERLVQDDVLTSIHIEEHEIDARDTKLGAEEITRDIPNVSEE
VLADLDERGIIRIGAEVRDGDILVGKVTPKGETELTPEERLLRAIFGEKAREVRDTSLKVPHGETGKVIG
IRVFSREDDDELPPGVNELVRVYVAQKRKIQPGDKLAGRHGNKGVIGKILPVEDMPFMEDGTPVDIILNT
HGVPRRMNIGQILELHLGWLASQGWTIEGDPDWAKNLSAELRDVAPGTNTATPVFDGAKEEELTGLLSAT
KPNRDGERMVKENGKANLFDGRSGEPYPYPVAVGYMYILKLHHLVDDKIHARSTGPYSMITQQPLGGKAQ
FGGQRFGEMECWAMQAYGAAYTLQELLTIKSDDVVGRVKVYEAIVKGENIPEPGIPESFKVLLKELQSLC
LNVEVLSSDGSSIEMRDSDDEDLERAAANLGINLSRNESPSVDDVVH
>gi|300782587|ref|YP_003762878.1| DNA-directed RNA polymerase subunit beta [Amycolatopsis mediterranei U32]
MAVSPANQATAATTSAESRSEATGIPGAPKRVSFAKIREPLNTPNLLDVQIQSFQWFTGDEAWFQRRVEE
GEENPVGGLEEVLNEISPIEDFSGSMSLSFSAPRFDEVKASIEECKDKDMTYAAPLFVTAEFVNNNTGEI
KSQTVFLGDFPVMTDKGTFVINGTERVVVSQLVRSPGVYYSKDIDKTSDKDVFSVRVIPSRGAWLEFDVD
KRDTVGVRIDRKRRQPVTVLLKALGWTTEAIRERFSFSETLLATLEKDHTAGTDEALLDIYRKLRPGEPP
TKESAQTLLENLFFKAKRYDLAKVGRYKVNKKLGLSTPIENGTLTEEDIVTIIEYLVRLHAGEDKMTAAN
NTEIPVETDDIDHFGNRRIRTVGELIQNQIRVGLSRTERVVRERMTTQDVEAITPQTLINIRPIGAAIKE
FFGTSQLSQFMQQTNPIDGLTHKRRLNALGPGGLSRERAGMEVRDVHPSHYGRMCPIETPEGPNIGLIGS
LCSYARVNPFGFIETPYRKVVEGRVTDQIDYLTADEEDRFVKAQANAPISDDGTFIEDRVMARRKGGEVE
LIDPLDIDYMDVSPRQMVSIATAMIPFLEHDDANRALMGANMQRQAVPLLRSQAPYVGTGVELRAAIDSG
DMLVAEQSGVVEELSADLITVMHDDGTRKSYSLYKFRRSNHGTCFNHRPIVNEGDRIEAGQVIADGPSTE
NGEVALGKNLLVAVMPWEGHNYEDAIILSERLVQDDVLTSIHIEEHEIDARDTKLGAEEITWDIPNVSEE
VLADLDERGIIRIGAEVRDGDILVGKVTPKGETELTPEERLLRAIFGEKAREVRDTSLKVPHGETGKVIG
IRVFSREDDDELPPGVNELVRVYVAQKRKIQSGDKLAGRHGNKGVIGKILPVEDMPFMEDGTPVDIILNT
HGVPRRMNIGQILELHLGWLASQGWTIEGDPDWAKNLSAELRDVAPGTNTATPVFDGAKEEELTGLLSAT

KPNRDGERMVKENGKANLFDGRSGEPYPYPVAVGYMYILKLHHLVDDKIHARSTGPYSMITQQPLGGKAQ
FGGQRFGEMECWAMQAYGAAYTLQELLTIKSDDVVGRVKVYEAIVKGENIPEPGIPESFKVLLKELQSLC
LNVEVLSSDGSSIEMRDSDDEDLERAAANLGINLSRNESPSVDDVVH
>gi|379737650|ref|YP_005331156.1| rpoB gene product [Blastococcus saxobsidens DD2]
MAGSRPTSINPSTTESGPFGQQNQSPVSGPRTGEADQAKIPGAPIRVSFAKISEPLAVPDLLALQTASFD
WLVGSPDWRATLSPEEQADAVGGLAEILEEISPIEDFSGSMSLSFSNPRFEDVKASLEECKDKDMTYAAP
LFVTAEFMNNTTGEIKSQTVFMGDFPIMTNKGTFVINGTERVVVSQLVRSPGVYFDKTLDKTSDKDVFSA
KVIPSRGAWLEFDVDKRDTVGVRIDRKRRQPVTVLLKALGWGEDRIREHFQWSPTVLATLEKDHIAGQDE
ALLDIYRKLRPGEPPTRESAQALLENLFFNPKRYDLAKVGRYKVKKKLGVDVPQSTSTLTEDDIVATIEY
VVRLHAGEPDHGTDDIDHFGNRRLRTVGELIQNQIRVGLSRMERVVRERMTTQDVEAITPQTLINIRPVV
ASIKEFFGTSQLSQFMDQTNPLAGLTHKRRLSALGPGGLSRERAGMEVRDVHPSHYGRMCPIETPEGPNI
GLIGSLSSFGRVNPFGFIETPYRKVENGTVTTQIDYLTADEEDRFVVAQANSPLDAQGRLAEERVLVRTK
GGEVDYLAPENVDYMDVSPRQMTSVATAMIPFLEHDDANRALMGANMQRQAVPLLRSEAPLVGTGMELRA
AVDAGDVVVAEKAGVVEDSTADYVTVMADDGTRQTYRLLKFRRSNQGTSINQSPVVDEGQRVEVGQVIAD
GPCTDQGEMALGKNLLVAFMPWEGHNYEDAIILSQRLVQDDVLSSIHIEEFEVDARDTKLGAEEITRDIP
NVSEDVLADLDERGIIRIGAEVVPGDILVGKVTPKGETELTPEERLLRAIFGEKAREVRDTSLKVKHGES
GKVIGVRVFSREDGDELPAGVNELIRVYVAQMRKISDGDKLAGRHGNKGVISKILPQEDMPFLEDGTPVD
IVLNPLGVPGRMNVGQVLETHLGWVAKQGWQVEGNPDWAKNLPEAAKSAGPGTRTATPVFDGAREEEIVG
LLGSTTPNRDGDRMVKETGKARLFDGRSGEPFPEPISVGYVYILKLLHLVDDKIHARSTGPYSMITQQPL
GGKAQFGGQRFGEMECWAMQAYGAAYALQELLTIKSDDILGRVKVYEAIVKGENIPEPGIPESFKVLLKE
LQSLCLNVEVLSGDGQAIELRDTDDEVFRAAEELGIDLSRREPSSVEDV
>gi|256390126|ref|YP_003111690.1| DNA-directed RNA polymerase subunit beta [Catenulispora acidiphila DSM 44928]
MAASRTAQNITKNAARRISFAKIKEPLEVPNLLALQTESFDWLVGNATWKGRVSEQQAAGLDVPTTSGLE
EIFEEISPIEDFQGTMSLTFRDHRFEPAKYSIDECKDRDFTYAAPLFVTAEFTNNETGEIKSQTVFMGDF
PLMTNKGTFIINGTERVVVTQLVRSPGLYFSQDIDKTSDKDVFNAKIIPSRGAWLEFEIDKRDMVGVRVD
RKRKQHVTVLLKALGWDDARILEEFGEYESMRQTLEKDHTAGQDEALLDIYRKLRPGEPPTREAAQTLLD
NLYFNPKRYDLAKVGRYKIDKKLGQASGIDNTVLTEDDIVNAIKFLVKLHAGETEMDPQAEGMPMVVVEE
DDIDHFGNRRLRSVGELIQNQVRTGLARMERVVRERMTTQDVEAITPQTLINIRPVVASIKEFFGTSQLS
QFMDQTNPLSGLTHKRRLNALGPGGLSRERAGFEVRDVHPSHYGRMCPIETPEGPNIGLIGSLASYGRIN
AFGFIETPYRKVEDGRVTDHIDYLTADEEDRYVVAQANAPVRDDGSYTDERVLCRRKGGEIEMMAPADID
YMDVSPRQMVSVATAMIPFLEHDDANRALMGANMMRQAVPLLQSDSPYVGTGMEYRCAVDAADVVTADKG
GVVTEVSADLVTVANDDATTSVYRIAKFRRSNQGTSFNQKVVVAEGERVEVGSVLADGPCTQDGEMALGK
NLLVAFMPWEGHNYEDAIILSQRLVQDDVLSSIHIEEHEVDARDTKLGPEEITRDIPNVSEEVLADLDER
GIIRIGADVVPGDILVGKVTPKGETELTPEERLLRAIFGEKAREVRDTSLKVPHGEQGKIIGVRIFDRDS
GDELPPGVNQLVRVYVAQKRKITHGDKLAGRHGNKGVIAKILPVEDMPFLEDGTPVDIVLNPLGVPSRMN
PGQVLETHLGWIAATGWDVGEGNAEWQERLRSIGADSAAPKTKVATPVFDGAREDEITGLLGNTLKTRDG
VRLVDSTGKARMFDGRSGEPFPYPISVGYMYILKLHHLVDDKLHARSTGPYSMITQQPLGGKAQFGGQRF
GEMEVWALEAYGAAYALQELLTIKSDDVLGRVKVYEAIVKGDNIPEPGIPESFKVLIKEMQSLCLNVEVL
SKDGTMIELRDTDEDVFRAAEELGIDLSRREPSSVEEV
>gi|332669509|ref|YP_004452517.1| DNA-directed RNA polymerase subunit beta [Cellulomonas fimi ATCC 484]
MAASRTPSAPTADAIANRTASRRISFAKIHEPLEVPDLLGLQTESFDWLLGNERWQARVAAALEAGRQDV
PETAGLEEIFEEISPIEDFGGSMSLSFREHRFEPPKYTAEECKEKDFTYAAPLFVTAEFVNYTTGEIKSQ
TVFMGDFPLMTERGTFIINGTERVVVSQLVRSPGVYFERMADKTSDKDILTAKVIPSRGAWLEFEIDKRD
NVGVRVDRKRKQNATVLLKALGMTEAEIREEFAAYPAVIETLEKDHVQTQDEALLDLYRKIRPGEPPTVE
AGRALIENFYFNPKRYDLAKVGRYKLNKKLGQDAPLADSVLTLADVVATIKYLASLHIDLPTLPGTRAGE
AIEIRVETDDIDHFGNRRIRAVGELIQNQVRTGLSRMERVVRERMTTQDVEAITPQTLINIRPVVASIKE
FFGTSQLSQFMDQNNPLAGLTHKRRLSALGPGGLSRDRAGMEVRDVHPSHYGRMCPIETPEGPNIGLIGS
LASFGRINPFGFVETPYRKVVDGRVTDEVHYLTADDEDRHVIAQANAPLLEDGTFEEDRVLVRVKGGEIE
YVTGENIDYMDVSPRQMVSVATALIPFLEHDDANRALMGANMQRQAVPLVRSEAPLVGTGMERRAAVDAG
DVIVATKPGVVTEVSADLIVTANDDATTSTYRVAKFRRSNQGTSYNQRVLVDTGARVEVGSVLADGPATD
EGELALGRNLLVAFMSWEGHNYEDAIILSQRLVQDDVLSSIHIEEHEVDARDTKLGPEEITRDIPNVSEE
VLADLDERGIIRIGAEVAAGDILVGKVTPKGETELTPEERLLRAIFGEKAREVRDTSLKVPHGESGTVIE
VRTFSREDGDELPAGVNELVRVYIAQRRKITDGDKLAGRHGNKGVISKILPVEDMPFLADGTPVDVVLNP
LGVPGRMNVGQVLETHLGWVAKQGWDIELAEGDVSWREGVPAVAAKSPAGNPVATPVFDGVPEETLTGLL
GSTLPNRDGERTVQQDGKARLFDGRSGEPFPDPVAVGYMYILKLHHLVDDKIHARSTGPYSMITQQPLGG

KAQFGGQRFGEMEVWALEAYGAAYTLQELLTIKSDDVPGRVKVYEAIVKGENIPDSGIPESFKVLLKEMQ
SLCLNVEVLSSDGVSIDMKENDDEVYRAAEELGIDLSRRPNASSIEEI
>gi|296130503|ref|YP_003637753.1| unnamed protein product [Cellulomonas flavigena DSM 20109]
MAASRIPTAPSADAIANRTASRRISFAKIHEPLEVPDLLGLQTESFDWLLGNERWQARVAAALEVGRNDV
PETAGLEEIFEEISPIEDFGGTMSLSFREHRFEPPKYTAEECKEKDFTFAAPLFVTAEFVNYTTGEIKSQ
TVFMGDFPLMTERGTFIINGTERVVVSQLVRSPGVYFERTADKTSDKDVLTAKVIPSRGAWLEFEIDKRD
NVGVRVDRKRKQNATVLLKALGMTESEIREEFAEYPAVIDTLEKDHVQTQDEALLDLYRKIRPGEPPTVE
AGRALLENFYFNPKRYDLAKVGRYKLNKKLGQDAPLSDSVLALSDVVATIKYLAALHIDKPTLPGTRGGQ
AVEIRVEPDDIDHFGNRRIRAVGELIQNQVRTGLSRMERVVRERMTTQDVEAITPQTLINIRPVVASIKE
FFGTSQLSQFMDQNNPLAGLTHKRRLSALGPGGLSRDRAGMEVRDVHTSHYGRMCPIETPEGPNIGLIGS
LATYGRINPFGFVETPYRRVVDGKVTDEVDYLTADDEDRHVIAQANAPLNADGSFTESTVLVRTKGGEPD
LVPGANVDYMDVSPRQMVSVATALIPFLEHDDANRALMGANMQRQAVPLVRSEAPLVGTGMERRAAVDAG
DVVVATKAGVVTEVSADLVTVANDDATTSTYRIAKFRRSNQGTCYNQRVLVEHGARVEPGSVLADGPATD
EGELALGRNLLVAFMSWEGHNYEDAIILSQRLVQDDVLSSIHIEEHEVDARDTKLGPEEITRDIPNVSEE
VLGDLDERGIIRIGAEVAAGDILVGKVTPKGETELTPEERLLRAIFGEKAREVRDTSLKVPHGESGTVIE
VRTFSRDDGDELPAGVNELVRVYIAQRRKITDGDKLAGRHGNKGVISKILPVEDMPFLEDGTPVDVVLNP
LGVPGRMNVGQVLETHLGWVAKQGWDIELAEGEATWRDGVPAVAARSTPGNPVATPVFDGVPEETLTGLL
STTLPNRDGERTVKGDGKARLFDGRSGEPFPEPVSVGYMYILKLHHLVDDKIHARSTGPYSMITQQPLGG
KAQFGGQRFGEMEVWALEAYGAAYTLQELLTIKSDDVPGRVKVYEAIVKGENIPDSGIPESFKVLLKEMQ
SLCLNVEVLSSDGVSIDMKENDDEVYRAAEELGIDLSRRPNASSIEEI
>gi|336319916|ref|YP_004599884.1| DNA-directed RNA polymerase, beta subunit [Cellulomonas gilvus ATCC 13127]
MAASRTPSAPTADAIANRTASRRISFAKIYEPLEVPDLLGLQTESMDWLLGNERWQARVAAALEAGRNDV
PQTAGLEEIFEEISPIEDFGGTMSLSFREHRFEPPKYTAEECKEKDFTFAAPLFVTAEFVNYTTGEIKSQ
TVFMGDFPLMTERGTFIINGTERVVVSQLVRSPGVYFERTADKTSDKDVLTAKVIPSRGAWLEFEIDKRD
NVGVRVDRKRKQNATVLLKALGMTEGEIREEFAAYPAVIDTLEKDHVQTQDEALLDLYRKIRPGEPPTVE
AGRALIENFYFNSKRYDLAKVGRYKVNKKLGQDAPLTDSVLSLSDVVATIKYLAALHIDQAALPGTRNGE
AIEVRVETDDIDHFGNRRIRAVGELIQNQVRTGLSRMERVVRERMTTQDVEAITPQTLINIRPVVASIKE
FFGTSQLSQFMDQNNPLAGLTHKRRLSALGPGGLSRDRAGMEVRDVHPSHYGRMCPIETPEGPNIGLIGS
LASFGRINPFGFVETPYRKVVKGKVTDEVHYLTADDEDRHVIAQANAPLKANGEFEESRVLVRTKGGEID
YVTGETVDYMDVSPRQMVSVATALIPFLEHDDANRALMGANMQRQAVPLVRSEAPLVGTGMERRAAIDAG
DVIVATKPGVVTEVSADLITVANDDATTTTYRVAKFRRSNQGTSYNQRVLVDHGARVEVGSVLADGPATD
EGELALGRNLLVAFMSWEGHNYEDAIILSQRLVQDDVLSSIHIEEHEVDARDTKLGPEEITRDIPNVSEE
VLADLDERGIIRIGAEVGAGDVLVGKVTPKGETELTPEERLLRAIFGEKAREVRDTSLKVPHGESGTVIE
VRTFSRDDGDELPAGVNELVRVYIAQRRKITDGDKLAGRHGNKGVISKILPVEDMPFLADGTAVDVVLNP
LGVPGRMNVGQVLETHLGWVAKQGWDIQLAEGDVSWRDGVPDVAASSPAGNPVATPVFDGVPEETLAGLL
GTTLPNRDGVRTVGPDGKARLFDGRSGEPFPDPVSVGYMYILKLHHLVDDKIHARSTGPYSMITQQPLGG
KAQFGGQRFGEMEVWALEAYGAAYTLQELLTIKSDDVPGRVKVYEAIVKGENIPDSGIPESFKVLLKEMQ
SLCLNVEVLSSDGVSIDMKENDDEVYRAAEELGIDLSRRPNASSVEEI
>gi|111220537|ref|YP_711331.1| rpoB gene product [Frankia alni ACN14a]
MAASRSSSRISFAKIIEPLEVPDLLALQTQSFDWLIGSDAWAERVQEAIDSGRDDVPITSGLEEVFEEIS
PIEDFSGSMSLSFRDHRFEPPKYSVEECKDKDMTFSAPLFVTAEFTNNTTGEIKSQTVFMGDFPLMTPKG
TFVINGTERVVVSQLVRSPGVYFERSLDKASDKDLYSCKVIPSRGAWLEFEIDKRDTVGVRIDRKRRQSV
TVLLKALGWDEARILERFGDFPSMRITLEKDHTASQDDALLDIYRKLRPGEPPTRESAQTLLENLFFNPK
RYDLAKVGRYKVNKKLTLEIAHDVGVLTEDDIVRTIEYVVKLHAGADPAEYEVDDIDHFGNRRLRTVGEL
IQNQVRLGLARMERVVRERMTTQDVEAITPQTLINIRPVVASIKEFFGTSQLSQFMDQTNPLAGLTHKRR
LSALGPGGLSRERAGFEVRDVHPSHYGRMCPIETPEGPNIGLIGSLSTFARVNPFGFVETPYRKVVNGRV
TDQIDYLTADEEDRHVKAQANTPLSPDGTFAEDRVLVRRKGGEVEFIPPDEVDYMDVSPRQMVSVATAMI
PFLEHDDANRALMGSNMQRQSVPLLRSEAPLVGTGMEARAAKDAGDVVVCVQAGVVEDLSADYITVMHDD
GTRRTYRLAKFRRSNQGTCINQKPIVFEGDRVEAGQVIADGPCTDNGEMALGKNLLVAFMPWEGHNYEDA
IILSQRLVQDDVLSSIHIEEHEVDARDTKLGPEEITRDIPNVAEEVLADLDERGIIRIGAEVSPGDVLVG
KVTPKGETELTPEERLLRAIFGEKAREVRDTSLKVPHGESGKVIGVRVFSREDGDELPPGVNELVRVYVA
QKRKITDGDKLAGRHGNKGVIAKILPQEDMPFLEDGTPVDIVLNPHGVPRRMNIGQILETHLGWVAKTGW
QVDAGTEDWKERLRGIGADSSTPGTNVATPVFDGAREEEITGLLDSTLPNRDGVQLIGSSGKATLFDGRT
GEPYPYPVAVGYIYILKLLHLVDDKIHARSTGPYSMITQQPLGGKAQFGGQRFGEMEVWALEAYGAAYAL
QELLTIKSDDVVGRVKVYEAIVKGENIPEPGIPESFKVLIKEMQSLCLNVEVLSSDGVQIEMRDTDEDVF
RAAEELGIDLSRREPSSVEEV

>gi|86739286|ref|YP_479686.1| DNA-directed RNA polymerase subunit beta [Frankia sp. CcI3]
MAASRSSSRISFAKIIEPLEVPDLLALQTQSFDWLIGSDAWAERVQEAIDSGRDDVPITSGLEEVFKEISP
IEDFSGSMSLSFRDHRFEPPKYSVEECKDKDMTFSAPLFVTAEFTNNNTGEIKSQTVFMGDFPLMTPKGTF
VINGTERVVVSQLVRSPGVYFERSLDKASDKDLYSCKVIPSRGAWLEFEIDKRDTVGVRIDRKRRQSVTVL
LKALGWDEARILERFGDFPSMRITLEKDHTAGQDDALLDIYRKLRPGEPPTRESAQTLLENLFFNPKRYDL
AKVGRYKVNKKLSLGVAHDVGVLTENDIVRTIEYVVKLHAGADPAEYEVDDIDHFGNRRIRTVGELIQNQV
RLGLARMERVVRERMTTQDVEAITPQTLINIRPVVASIKEFFGTSQLSQFMDQTNPLAGLTHKRRLNALGP
GGLSRERAGFEVRDVHPSHYGRMCPIETPEGPNIGLIGSLSTFARVNPFGFIETPYRKVENGRVTGQIDWL
TADEEDRHVKAQANTPLRPDGSFAEDRVLVRRKGGEVEFIPPDEVDYMDVSPRQMVSVATAMIPFLEHDDA
NRALMGSNMQRQSVPLLRSEAPLVGTGMEARAAKDAGDVVVCAQAGVVEDLSADYITVMHDDGTRRTYRLA
KFRRSNQGTCINQKPIVNEGDRVGAGQVIADGPCTDNGEMALGKNLLVAFMPWEGHNYEDAIILSQRLVQD
DVLSSIHIEEHEVDARDTKLGPEEITRDIPNVAEEVLADLDERGIIRIGAEVSPGDVLVGKVTPKGETELT
PEERLLRAIFGEKAREVRDTSLKVPHGESGKVIGVRVFSREDGDELPPGVNELVRVYVAQKRKITDGDKLA
GRHGNKGVIAKILPAEDMPFLEDGTPVDVVLNPHGVPRRMNIGQILETHLGWVAKTGWQVDSGTEDWKERL
RGIGADAASAGTNVATPVFDGAREEEITGLLDSTLPNRDGIQLIGSSGKAKLFDGRTGEPYPYPVAVGYIY
ILKLLHLVDDKIHARSTGPYSMITQQPLGGKAQFGGQRFGEMEVWALEAYGAAYALQELLTIKSDDVVGRV
KVYEAIVKGENIPEPGIPESFKVLIKEMQSLCLNVEVLSSDGVQIEMRDTDEDVFRAAEELGIDLSRREPS
SVEEV
>gi|358459761|ref|ZP_09169955.1| DNA-directed RNA polymerase subunit beta [Frankia sp. CN3]
MAASRSASRISFAKIVEPLEVPDLLALQTQSFDWLIGSDTWQERVQESIDAGRDDVPLTSGLEEVFEEIS
PIEDFSGSMSLSFRDHRFEPSKYSVEECKDKDMTYSAPMFVTAEFTNNTTGEIKSQTVFMGDFPLMTPKG
TFVINGTERVVVSQLVRSPGVYFEKSLDKTSDKDLFSCKVIPSRGAWLEFEIDKRDTVGVRIDRKRRQSV
TVLLKALGWDEARILERFGDYPSMRVTLEKDHTAGQDDALLDIYRKLRPGEPPTRESAQTLLENLFFNPK
RYDLAKVGRYKVNKKLGLHVEGPVGVLTEEDVVRTIEYVVKLHEGIDPAYEVDDIDHFGNRRLRTVGELI
QNQVRLGLARMERVVRERMTTQDVEAITPQTLINIRPVVASIKEFFGTSQLSQFMDQTNPLAGLTHKRRL
SALGPGGLSRERAGMEVRDVHPSHYGRMCPIETPEGPNIGLIGSLSTFARVNPFGFVETPYRKVVAGRVT
DQIDYLTADEEDRHVVAQANAPLTGDGSFAEDRVLVRRKGGEVEFIPPDEVDYMDVSPRQMVSVATAMIP
FLEHDDANRALMGSNMQRQSVPLLRSESPLVGTGMEARAAKDAGDVVVCQQAGVVEDLSADYITVMHDDG
TRRTYRLAKFRRSNQGTCINQKPIVDEGDRVESGQVIADGPCTDNGEMALGKNLLVAFMPWEGHNYEDAI
ILSQRLVQDDVLSSIHIEEHEVDARDTKLGPEEITRDIPNVAEEVLADLDERGIIRIGAEVNPGDVLVGK
VTPKGETELTPEERLLRAIFGEKAREVRDTSLKVPHGESGKVIGVRVFSREDGDELPPGVNELVRVYVAQ
KRKITDGDKLAGRHGNKGVIAKIMPQEDMPFLEDGTPVDVVLNPHGVPRRMNIGQILETHLGWVAKTGWQ
VDAGKEEWKERLRTIGADAANPGQNVATPVFDGAREEEITGLLDSTLPNRDGVQLIGASGKAKLFDGRTG
EQYPYPVAVGYIYILKLLHLVDDKIHARSTGPYSMITQQPLGGKAQFGGQRFGEMEVWALEAYGAAYALQ
ELLTIKSDDVHGRVKVYEAIVKGENIPEPGIPESFKVLIKEMQSLCLNVEVLSSDGVQIEMRDTDEDVFR
AAEELGIDLSRREPSSVEEV
>gi|158317799|ref|YP_001510307.1| rpoB gene product [Frankia sp. EAN1pec]
MAASRSASRISFAKIIEPLEVPDLLALQTQSFDWLIGNDIWAERVQEAIDADRNDVPITSGLEEVFEEIS
PIEDFSGSMSLSFRDHRFEPPKYSVEECKDKDMTYSAPLFVTAEFTNNTTGEIKSQTVFMGDFPLMTPKG
TFVINGTERVVVSQLVRSPGVYFEKAIDKASDKDLFSCKVIPSRGAWLEFEIDKRDTVGVRIDRKRRQSV
TVLLKALGWDEARILERFGDFPSMRVTLEKDHTSSQDDALLDIYRKLRPGEPPTRESAQTLLENLFFNPK
RYDLAKVGRYKVNKKLTLEVAHDVGVLTEDDVVRTIEYIVKLHAGQDPDNYEVDDIDHFGNRRLRTVGEL
IQNQVRLGLARMERVVRERMTTQDVEAITPQTLINIRPVVASIKEFFGTSQLSQFMDQTNPLAGLTHKRR
LSALGPGGLSRERAGFEVRDVHPSHYGRMCPIETPEGPNIGLIGSLSTFARVNPFGFVETPYRKVENGRV
TGQIDYLTADEEDRHVKAQANTPLNADGTFAEDRVLVRRKGGEVEFIPPDEVDYMDVSPRQMVSVATAMI
PFLEHDDANRALMGSNMQRQSVPLLRSEAPLVGTGMEARAAKDAGDVVVCAQSGVVEDLSADYITVMHDD
GTRRTYRLAKFRRSNQGTCINQKPIVFEGDRVEAGQVIADGPCTDNGEMALGKNLLVAFMPWEGHNYEDA
IILSQRLVQDDVLSSIHIEEHEVDARDTKLGPEEITRDIPNVAEEVLADLDERGIIRIGAEVSPGDVLVG
KVTPKGETELTPEERLLRAIFGEKAREVRDTSLKVPHGESGKVIGVRVFSREDGDELPPGVNELVRVYVA
QKRKITDGDKLAGRHGNKGVIAKILPVEDMPFLPDGTAVDVVLNPHGVPRRMNIGQILETHLGWVAKTGW
QVDSGAEGWKERLRGIGADAAPPGTNVATPVFDGAREEEITGLLDATLPNRDGQQMIGSSGKAELYDGRT
GEPYPYPVAVGYIYILKLLHLVDDKIHARSTGPYSMITQQPLGGKAQFGGQRFGEMEVWALEAYGAAYAL
QELLTIKSDDVVGRVKVYEAIVKGENIPEPGIPESFKVLIKEMQSLCLNVEVLSSDGVSIEMRDTDEDVF
RAAEELGIDLSRREPSSVEEV

>gi|288917220|ref|ZP_06411589.1| DNA-directed RNA polymerase, beta subunit [Frankia sp. EUN1f]
MAASRSASRISFAKIIEPLEVPDLLALQTQSFDWLIGNDAWAERVQESIDAGRDDVPITSGLEEVFEEIS
PIEDFSGSMSLSFRDHRFEPPKYSVEECKDKDMTFSAPLFVTAEFTNNTTGEIKSQTVFMGDFPLMTTKG
TFVINGTERVVVSQLVRSPGVYFEKAIDKASDKDLFSCKIIPSRGAWLEFEIDKRDTVGVRIDRKRRQSV
TVLLKALGWDEARILERFGEFPSMRVTLEKDHTSSQDDALLDIYRKLRPGEPPTRESAQTLLENLFFNPK
RYDLAKVGRYKVNKKLELEVAYDVGVLTEDDVVRTIEYIVKLHAGQEPDTYEVDDIDHFGNRRLRTVGEL
IQNQVRLGLARMERVVRERMTTQDVEAITPQTLINIRPVVASIKEFFGTSQLSQFMDQTNPLAGLTHKRR
LSALGPGGLSRERAGFEVRDVHPSHYGRMCPIETPEGPNIGLIGSLSTFARVNPFGFVETPYRKVENGRV
TGQIDYLTADEEDRHVKAQANTPLNADGSFAEERVLVRRKGGEVEFIPPDEVDYMDVSPRQMVSVATAMI
PFLEHDDANRALMGSNMQRQSVPLLRSEAPLVGTGMEARAAKDAGDVVVCAQSGVVEDLSADYITVMHDD
GTRRTYRLAKFRRSNQGTCINQKPIVFEGDRVEAGQVIADGPCTDNGEMALGKNLLVAFMPWEGHNYEDA
IILSQRLVQDDVLSSIHIEEHEVDARDTKLGPEEITRDIPNVAEEVLADLDERGIIRIGAEVSPGDVLVG
KVTPKGETELTPEERLLRAIFGEKAREVRDTSLKVPHGESGKVIGVRVFSREDGDELPPGVNELVRVYVA
QKRKITDGDKLAGRHGNKGVIAKILPVEDMPFLSDGTAVDVVLNPHGVPRRMNIGQILETHLGWVAKTGW
QVDSGSEDWKERLRGIGADAAPPGTNVATPVFDGAREEEITGLLDATLPNRDGTQLIGSSGKAELYDGRT
GEPYPYPVAVGYIYILKLLHLVDDKIHARSTGPYSMITQQPLGGKAQFGGQRFGEMEVWALEAYGAAYAL
QELLTIKSDDVVGRVKVYEAIVKGENIPEPGIPESFKVLIKEMQSLCLNVEVLSSDGVSIEMRDTDEDVF
RAAEELGIDLSRREPSSVEEV
>gi|392944827|ref|ZP_10310469.1| DNA-directed RNA polymerase, beta subunit [Frankia sp. QA3]
MAASRSSSRISFAKIIEPLEVPDLLALQTQSFDWLIGSDAWAERVQEAIDSGRDDVPITSGLEEVFEEIS
PIEDFSGSMSLSFRDHRFEPPKYSVEECKDKDMTFSAPLFVTAEFTNNTTGEIKSQTVFMGDFPLMTPKG
TFVINGTERVVVSQLVRSPGVYFERSLDKASDKDLYSCKVIPSRGAWLEFEIDKRDTVGVRIDRKRRQSV
TVLLKALGWDEARILERFGDYPSMRITLEKDHTASQDDALLDIYRKLRPGEPPTRESAQTLLENLFFNPK
RYDLAKVGRYKVNKKLTLEIAHDVGVLTEDDIVRTIEYVVKLHAGADPAEYEVDDIDHFGNRRLRTVGEL
IQNQVRLGLARMERVVRERMTTQDVEAITPQTLINIRPVVASIKEFFGTSQLSQFMDQTNPLAGLTHKRR
LSALGPGGLSRERAGFEVRDVHPSHYGRMCPIETPEGPNIGLIGSLSTFARVNPFGFVETPYRKVVNGRV
TDQIDYLTADEEDRHVKAQANTPLQPDGAFAEDRVLVRRKGGEVEFIPPDEVDYMDVSPRQMVSVATAMI
PFLEHDDANRALMGSNMQRQSVPLLRSEAPLVGTGMEARAAKDAGDVVVCVQAGVVEDLSADYITVMHDD
GTRRTYRLAKFRRSNQGTCINQKPIVFEGDRVEAGQVIADGPCTDNGEMALGKNLLVAFMPWEGHNYEDA
IILSQRLVQDDVLSSIHIEEHEVDARDTKLGPEEITRDIPNVAEEVLADLDERGIIRIGAEVSPGDVLVG
KVTPKGETELTPEERLLRAIFGEKAREVRDTSLKVPHGESGKVIGVRVFSREDGDELPPGVNELVRVYVA
QKRKITDGDKLAGRHGNKGVIAKILPAEDMPFLEDGTPVDIVLNPHGVPRRMNIGQILETHLGWVAKTGW
QVDTGDEEWKERLRGIGADAAPAGTNVATPVFDGAREEEITGLLDSTLPNRDGVQLIGSSGKATLFDGRT
GEPYPYPVAVGYIYILKLLHLVDDKIHARSTGPYSMITQQPLGGKAQFGGQRFGEMEVWALEAYGAAYAL
QELLTIKSDDVVGRVKVYEAIVKGENIPEPGIPESFKVLIKEMQSLCLNVEVLSSDGVQIEMRDTDEDVF
RAAEELGIDLSRREPSSVEEV
>gi|336179792|ref|YP_004585167.1| unnamed protein product [Frankia symbiont of Datisca glomerata]
MAASPTSSRISFAKIIEPLEVPDLLALQTQSFDWLIGSDAWAERVQEAIDAGRDDVPVTSGLEEVFEEIS
PIEDFSGSMSLSFRDHRFEPPKYSVEECKDKDMTYSAPMFVTAEFTNNTTGEIKSQTVFMGDFPLMTPKG
TFVINGTERVVVSQLVRSPGVYFEKTIDKVSDKDLFSCKVIPSRGAWLEFEVDKRDTVGVRIDRKRRQSV
TVLLKALGWDEARILERFGDFPSMRVTLEKDHTSSQDDALLDIYRKLRPGEPPTRESAQTLLENLFFNPK
RYDLAKVGRYKVNKKLEVDVPHDVGVLTEDDIVRTIEYVVQLHAGRDSSYDVDDIDHFGNRRLRTVGELI
QNQVRLGLARMERVVRERMTTQDVEAITPQTLINIRPVVASIKEFFGTSQLSQFMDQTNPLAGLTHKRRL
SALGPGGLSRERAGFEVRDVHPSHYGRMCPIETPEGPNIGLIGSLSTFARVNPFGFVETPYRKVVAGRVT
DQIDYLTADEEDRHVIAQANAPLSSDGSFAEHRVLVRRKGGEVEFIPPDEVDYMDVSPRQMVSVATAMIP
FLEHDDANRALMGSNMQRQSVPLLRSEAPLVGTGMEARAAKDAGDVVVCAQAGVVEDLSADYITVMHDDG
TRRTYRMAKFRRSNQGTCINQKPIVDEGDRVEAGQVIADGPCTDNGEMALGKNLLVAFMPWEGHNYEDAI
ILSQRLVQDDVLSSIHIEEHEVDARDTKLGPEEITRDIPNVAEEVLADLDERGIIRIGAEVSPGDVLVGK
VTPKGETELTPEERLLRAIFGEKAREVRDTSLKVPHGESGKVIGVRVFSREDGDELPPGVNELVRVYVAQ
KRKITDGDKLAGRHGNKGVIAKILPAEDMPFLSDGTPVDVVLNPHGVPRRMNIGQILETHLGWVAKTGWQ
VDGGAEGWKERLRGIRADQAPPNTNVATPVFDGAREEEITGLLASTLPNRDGVQLIGASGKAELFDGRTG
EPYPYPVAVGYIYILKLLHLVDDKIHARSTGPYSMITQQPLGGKAQFGGQRFGEMEVWALEAYGAAYALQ
ELLTIKSDDVLGRVKVYEAIVKGENIPEPGIPESFKVLIKEMQSLCLNVEVLSSDGVSIEMRDTDEDVFR
AAEELGIDLSRREPSSVEEV

>gi|284992886|ref|YP_003411440.1| DNA-directed RNA polymerase subunit beta [Geodermatophilus obscurus DSM 43160]
MAGSRPTSINPRTTDSGPYGQQSQAPQAGPRTGEADQQKIPGAPLRVSFAKISEPLEVPDLLALQTASFD
WLIGSPTWRATLSPEEQADAVGGLAEILEEISPIEDFSGSMSLSFSNPRFEDVKASLEECKDKDMTYAAP
LFVTAEFMNNTTGEIKSQTVFMGDFPIMTNKGTFVINGTERVVVSQLVRSPGVYFDKTLDKTSDKDVFSA
KVIPSRGAWLEFDVDKRDTVGVRIDRKRRQPVTVLLKALGWTEDRIREHFQWSPTVLATLEKDHIAGQDE
ALLDIYRKLRPGEPPTRESAQALLENLFFNGKRYDLAKVGRYKVNKKLGVDVPQATLTLTEDDIVATIEY
VVRLHAGEPDHGVDDIDHFGNRRLRTVGELIQNQIRVGLSRMERVVRERMTTQDVEAITPQTLINIRPVV
ASIKEFFGTSQLSQFMDQTNPLAGLTHKRRLSALGPGGLSRERAGMEVRDVHPSHYGRMCPIETPEGPNI
GLIGSLSAYGRVNAFGFIETPYRRVQNGVVTDTIDYLTADEEDRFVVAQANSPLDAQGRFAEDRVLVRTK
GGEVDYLVPENVDYMDVSPRQMTSVATAMIPFLEHDDANRALMGANMQRQAVPLLRSEAPLVGTGMELRA
AVDAGDVVVAEKAGVVEDSTADYVTVMADDGTRQTYRLLKFRRSNQGTSINQTPVVDEGQRVEVGQVIAD
GPCTDEGEMALGKNLLVAFMPWEGHNYEDAIILSQRLVQDDVLSSIHIEEFEVDARDTKLGAEEITRDIP
NVSEEVLADLDERGIIRIGAEVVPGDILVGKVTPKGETELTPEERLLRAIFGEKAREVRDTSLKVKHGES
GKVIGVRVFSREDGDELPAGVNELIRVYVAQMRKISDGDKLAGRHGNKGVIAKILPQEDMPFLEDGTPVD
IVLNPLGVPGRMNVGQVLETHLGWVAKSGWQVEGTPEWAVNLPDPARAAGPGTRTATPVFDGAREEEIVG
LLGSTIPNRDGNRMVQPTGKARLFDGRSGEPFPEAISVGYVYILKLLHLVDDKIHARSTGPYSMITQQPL
GGKAQFGGQRFGEMECWAMQAYGAAYALQELLTIKSDDILGRVKVYEAIVKGENIPEPGIPESFKVLLKE
LQSLCLNVEVLSGDGQAIELRDTDDEVFRAAEELGIDLSRREPSSVEDV
>gi|152964649|ref|YP_001360433.1| DNA-directed RNA polymerase subunit beta [Kineococcus radiotolerans SRS30216]
MEGPLLAASLPASAPNSTYIGNQSPRTVSGRYSFGKIHEPLEVPDLLALQTDSFDWLLGNKRWQDRVEAS
TNGGLAVPTTSGLEEIFEEISPIEDFSGSMSLSFRDHRFEPPKYSLDDCKERDLTYSAPLFVTAEFINGN
TGEIKSQTVFMGDFPLMTDRGTFVINGTERVVVSQLVRSPGIYFERVPDKTSDRDTWTAKIIPSRGAWLE
FEIDKRDTVGVRVDRKRKQSVTVLMKALGWSESQIREEFADYESMISTLEKDHTSGVEDALLDIYRKLRP
GEPPTQEAARNLLDNLYFNPKRYDLAKVGRYKVNKKLGTEEPLSDSVLSVDDIVRTIKYLVKLHAGEVTM
PGVKNGQPVDVRVEVDDIDHFGNRRLRSVGELIQNQVRTGLSRMERVVRERMTTQDVEAITPQTLINIRP
VVASIKEFFGTSQLSQFMDQTNPLAGLTHKRRLSALGPGGLSRERAGMEVRDVHPSHYGRMCPIETPEGP
NIGLIGSLSSYGRINPFGFIETPYRKIVDGVVSDEVEYLTADEEDAFVIAQANAPLDADSRFAEARVLVR
AKGGETEFVPRDEVDYMDVAARQMVSVATAMIPFLEHDDANRALMGANMQRQAVPLVKSEAPLIGTGMEF
RAAVDAGDVVVATKAGVATDVSADMITTSNDDGTSTTYKVAKFRRSNHGTAYNQQVVINEGDRVEVGTVL
ADGPSTDGGEMALGRNLMVAFMPWEGHNYEDAIILSQRLVQDDVLSSIHIEEHEVDARDTKLGPEEITRD
IPNVAEEVLADLDERGIIRIGAEVRDGDLLVGKVTPKGETELTPEERLLRAIFGEKAREVRDTSLKVPHG
ETGTVIGVKVFDRDEGDELPPGVNQLVRVYVANKRKITDGDKLAGRHGNKGVISKILPVEDMPFLEDGTP
VDVILNPLGVPSRMNVGQVLELHLGWIASRGWKIEGQPDWAKLIPEEIREAPAGSRIATPVFDGAREEEI
TGLLSSTIPTRDGDRLVGGDGKARLFDGRSGEPFPDPVAVGYMYILKLHHLVDDKIHARSTGPYSMITQQ
PLGGKAQFGGQRFGEMEVWALEAYGAAYALQELLTIKSDDVLGRVKVYEAIVKGENIPEPGIPESFKVLI
KEMQSLCLNVEVLSSDGMAIEMRDSDEDVFRAAEELGIDLARREPSSVEEV
>gi|357390126|ref|YP_004904966.1| rpoB gene product [Kitasatospora setae KM-6054]
MAAPRNASNNSASTAPLRVSFAKIKEPLEVPNLLALQTESFDWLLGNAAWKSRVEAALESGQDVPTKSGL
EEIFEEISPIEDFSGSMSLTFRDHRFEPPKNSIDECKDRDFTFAAPLFVTAEFTNNETGEIKSQTVFMGD
FPLMTNKGTFIINGTERVVVSQLVRSPGVYFDSTLDKVSDKDIFSAKIIPSRGAWLEMEIDKRDMVGVRI
DRKRKQSVTVLLKALGWTTEMILEEFGEYESMRATLEKDHTQGQDDALLDIYRKLRPGEPPTREAAQTLL
ENLYFNPKRYDLAKVGRYKVNRKLGNAESLDSGVLTEPDIINTIKYLVKLHAGETEWRDDEGRDIVIEVD
DIDHFGNRRLRNVGELIQNQVRTGLARMERVVRERMTTQDVEAITPQTLINIRPVVASIKEFFGTSQLSQ
FMDQTNPLSGLTHKRRLSALGPGGLSRERAGFEVRDVHPSHYGRMCPIETPEGPNIGLIGSLASYGRVNA
FGFIETPYRKVVDGVVTESVDYLTADEEDRYVIAQANAPLTAELTFAEPRVLVRRRGGEIDYIPGTEIDY
MDVSPRQMVSVATAMIPFLEHDDANRALMGSNMMRQAVPLLRSEAPLVGTGMEYRCAVDAADVITADKAG
VVQEVSADYVTVANDDGTYNTYRAAKFTRSNQGTAFNQKVLVDEGQRVEVNQVLADGPCTDEGEMALGKN
LLVAFMSWEGHNYEDAIILSQRLVQDDVLSSIHIEEHEVDARDTKLGPEEITRDIPNVSEEVLADLDERG
IIRIGADVVTGDILVGKVTPKGETELTPEERLLRAIFGEKAREVRDTSLKVPHGESGKVIGVRVFDREEG
DELPPGVNQLVRVYVAQKRKITNGDKLAGRHGNKGVISKILPVEDMPFLEDGTPVDIILNPLGVPSRMNP
GQVLEIHLGWLAMQGWDVSGLADEWAQRLQAIGADTVEGGTNLATPVFDGAREDEITGLLDNTTLTRDGE
RLVNSTGKARLFDGRSGEPFPMPVSVGYMYILKLHHLVDDKLHARSTGPYSMITQQPLGGKAQFGGQRFG
EMEVWALEAYGAAYALQELLTIKSDDVLGRVKVYEAIVKGENIPEPGIPESFKVLIKEMQSLCLNVEVLS
SDGQSIEMRDSDEDVFRAAEELGIDLSRREPSSVEEV

>lcl|KUTG_05840T0 | KUTG_05840 | Kutzneria sp. 744 DNA-directed RNA polymerase subunit beta (1165 aa)
MEGRILAVSRATKATAATNSTGIPGAPRRVSFAKIREPLAAPNLLELQTHSFEWLTGNEAWFQRRVDAGDE
APTGGLEEVLNEISPIEDFSGSMSLSFSDPRFDEVKASVEDCKDKDMTYAAPLFVTAEFTNHTTGEIKSQT
VFMGDFPMMTDKGTFIINGTERVVVSQLVRSPGVYYDTAIDKTTDKDVFNVKIIPSRGAWLEFDVDKRDTV
GVRIDRKRRQPVTVLLKALGWTTEQIRERFHFSETLLATLEKDHTAGPDEALLDIYRKLRPGEPPTKESAQ
ALLENLFFKEKRYDLAKVGRYKVNKKLGLGFPVSTGVLTEDDIVTTIEYLVRLHAGDTEMTAGDTTVPVEV
DDIDHFGNRRLRTVGELIQNQIRVGLSRMERVVRERMTTQDVEAITPQTLINIRPVVAAIKEFFGTSQLSQ
FMDQTNPLAGLTHKRRLSALGPGGISRERASMDVRDVHPSHYGRMCPIETPEGPNIGLIGSLSSFARVNPF
GFIESPYRKVVDGRVTDQIDYLTADEEDRYVKAQANTLVDDEGNFLEDRVMVRRKGGETDLADTADVDYMD
VSPRQMVSVATAMVPFLEHDDANRALMGANMQRQAVPLLRSESPLVGTGMELRAAVDAGDVVIAKKSGVVE
ELSADFITVMADDGSRQTYAMHKFRRSNQGTCINQKPIVNEGDRVEHGQVIADGPCTQNGEMALGKNLLVA
IMPWEGHNYEDAIILSQRLVQDDVLTSIHIEEHEIDARDTKLGAEEITRDIPNVSEEVLADLDERGIIRIG
AEVRDGDILVGKVTPKGETELTPEERLLRAIFGEKAREVRDTSLKVPHGETGKVIGIRVFSREDDDELPPG
VNELVRVYVAQKRRIQDGDKLAGRHGNKGVIGKILPVEDMPFMPDGTPVDIVLNTHGVPRRMNIGQVLETH
LGWIAKQGWSIDGDPEWASKMPEELYEVPPNTNTATPVFDGAREEEIIGLLGSTTPNRDGERMVGQNGKAT
LLDGRSGEPYPFPVAVGYMYILKLLHLVDDKIHARSTGPYSMITQQPLGGKAQFGGQRFGEMECWAMQAYG
AAYTLQELLTIKSDDKLGRVKVYEAIVKGQNVPEPGVPESFKVLLKELQSLCLNVEVLSSDGAAIEMRDGD
DEDLERAAANLGINLSRNESPSVDDVVN
>gi|302869981|ref|YP_003838618.1| DNA-directed RNA polymerase subunit beta [Micromonospora aurantiaca ATCC 27029]
MAASRPAKTSRTSSAFAPRRVSFGRITEHLEVPNLLAIQNESFDWLVGNEAWQGRSADDPHARSGLAEILD
EISPIEDFSGTMSLSFSAPRFDEVKASIEECKEKDLTYCAPLFVTAEFTNNTTGEIKSQTVFMGDFPMMTP
KGTFIINGTERVVVSQLVRSPGVYFDKQPDKTSDRDLSSVKVIPSRGAWLEFDIDKRDTVGVRIDRKRRQA
VTVLLKAIGWSAERIREKFGWSELMMTTLEKDHIAGQDEALLDIYRKLRPGEPPTRENAQTLLDNLFFNPK
RYDVAKVGRYKFNKKLELDVPITTGTLTEDDIVATVEYLCRLHAGEEGYEADDIDHFGNRRLRTVGELIQN
QVRVGLSRMERVVRERMTTQDVEAITPQTLINIRPVVAAIKEFFGTSQLSQFMDQTNPLAGLTHRRRLSAL
GPGGLSRERAGFEVRDVHPSHYGRMCPIETPEGPNIGLIGALSTFARVNPFGFIETPYRKVIDGRVTDQID
YLTADEEDRFVKAQANAPLKSDGTFAEDRVLVRRKGGETEDVAPGAVDYMDVSPRQMTSVATAMIPFLEHD
DANRALMGANMQRQAVPLVKAEAPLVGTGMEYRAAVDAGDVVVAEVGGVVEDLCADYITVHQDDGHRRTYL
LHKFRRSNAGSCVNQKPVVFEGDRVEAGQVIADGPCTDEGEMALGRNLLVAFMCWEGHNYEDAIILSQRLV
QQDVLTSIHIEEHEVDARDTKLGPEEITRDIPNVSEEMLADLDERGIIRIGAEVVPGDILVGKVTPKGETE
LTPEERLLRAIFGEKAREVRDTSLKVPHGETGTVIGVRTFSREDGDELPPGVNELVRVYVAQKRKIQDGDK
LAGRHGNKGVISKILPIEDMPFLEDGTPVDIVLNPLGVPSRMNIGQVLETHLGWVAKTGWSVDGDDAEWKR
QLRSINAHESEPDTNVATPVFDGAREEEISGLLASTLPNRDGNQLIGGSGKAQLFDGRSGEPLPDPIAVGY
VYILKLNHLVDDKIHARSTGPYSMITQQPLGGKAQFGGQRFGEMECWAMQAYGAAYALQELLTIKSDDVLG
RVKVYEAIVKGENIPEPGIPESFKVLLKELQSLCLNVEVLSSDGVALEMRETDDEVFRAAEELGIDLSRRE
PSSVEEV
>gi|443294185|ref|ZP_21033279.1| DNA-directed RNA polymerase subunit beta [Micromonospora lupini str. Lupac 08]
MAASRPAKTSRTSSAFAPRRVSFGRITEHLEVPNLLAIQNESFDWLVGNEAWQGRSADDPHARSGLAEIL
EEISPIEDFSGTMSLSFSAPRFDEVKASIEECKEKDLTYCAPLFVTAEFTNNTTGEIKSQTVFMGDFPMM
TPKGTFVINGTERVVVSQLVRSPGVYFDKQPDKTSDRDLSSVKVIPSRGAWLEFDIDKRDTVGVRIDRKR
RQAVTVLLKAIGWSAERIREKFGWSELMMTTLEKDHIAGADEALLDIYRKLRPGEPPTRENAQTLLDNLF
FNPKRYDVAKVGRYKFNKKLEVDVPITTGTLTEDDIVATVEYLCRLHAGEDGYEADDIDHFGNRRLRTVG
ELIQNQVRVGLSRMERVVRERMTTQDVEAITPQTLINIRPVVAAIKEFFGTSQLSQFMDQTNPLAGLTHR
RRLSALGPGGLSRERAGFEVRDVHPSHYGRMCPIETPEGPNIGLIGALSTFARVNPFGFIETPYRKVVEG
RVTDQIDYLTADEEDRFVKAQANARLKADGTFAEERVLCRRKGGETEDVVPGAVDYMDVSPRQMTSVATA
MIPFLEHDDANRALMGANMQRQAVPLVKAEAPLVGTGMEYRAAVDAGDVVVAEVGGVIEDLCADYVTIHQ
DDGHRRTYLLHKFRRSNAGSCVNQKPVVFEGDRVEAGQVIADGPCTDEGEMALGRNLLVAFMCWEGHNYE
DAIILSQRLVQQDVLTSIHIEEHEVDARDTKLGPEEITRDIPNVSEEMLADLDERGIIRIGAEVVPGDIL
VGKVTPKGETELTPEERLLRAIFGEKAREVRDTSLKVPHGETGTVIGVRTFSREDGDELPPGVNELVRVY
VAQKRKIQDGDKLAGRHGNKGVISKILPIEDMPFLEDGTPVDIVLNPLGVPSRMNIGQVLETHLGWVAKT
GWKVEGDDTDWKRQLRSIDAHESEGDTNVATPVFDGAREEEISGLLESTLPNRDGKQLIGRTGKAQLFDG
RSGEPLPDPIAVGYVYILKLNHLVDDKIHARSTGPYSMITQQPLGGKAQFGGQRFGEMECWAMQAYGAAY
ALQELLTIKSDDVLGRVKVYEAIVKGENIPEPGIPESFKVLLKELQSLCLNVEVLSSDGVALEMRETDDE
VFRAAEELGIDLSRREPSSVEEV

>gi|315501442|ref|YP_004080329.1| DNA-directed RNA polymerase subunit beta [Micromonospora sp. L5]
MAASRPAKTSRTSSAFAPRRVSFGRITEHLEVPNLLAIQNESFDWLVGNEAWQGRSADDPHARSGLAEIL
DEISPIEDFSGTMSLSFSAPRFDEVKASIEECKEKDLTYCAPLFVTAEFTNNTTGEIKSQTVFMGDFPMM
TPKGTFIINGTERVVVSQLVRSPGVYFDKQPDKTSDRDLSSVKVIPSRGAWLEFDIDKRDTVGVRIDRKR
RQAVTVLLKAIGWSAERIREKFGWSELMMTTLEKDHIAGQDEALLDIYRKLRPGEPPTRENAQTLLDNLF
FNPKRYDVAKVGRYKFNKKLELDVPITTGTLTEDDIVATVEYLCRLHAGEEGYEADDIDHFGNRRLRTVG
ELIQNQVRVGLSRMERVVRERMTTQDVEAITPQTLINIRPVVAAIKEFFGTSQLSQFMDQTNPLAGLTHR
RRLSALGPGGLSRERAGFEVRDVHPSHYGRMCPIETPEGPNIGLIGALSTFARVNPFGFIETPYRKVIDG
RVTDQIDYLTADEEDRFVKAQANAPLKSDGTFAEDRVLVRRKGGETEDVAPGAVDYMDVSPRQMTSVATA
MIPFLEHDDANRALMGANMQRQAVPLVKAEAPLVGTGMEYRAAVDAGDVVVAEVGGVVEDLCADYITVHQ
DDGHRRTYLLHKFRRSNAGSCVNQKPVVFEGDRVEAGQVIADGPCTDEGEMALGRNLLVAFMCWEGHNYE
DAIILSQRLVQQDVLTSIHIEEHEVDARDTKLGPEEITRDIPNVSEEMLADLDERGIIRIGAEVVPGDIL
VGKVTPKGETELTPEERLLRAIFGEKAREVRDTSLKVPHGETGTVIGVRTFSREDGDELPPGVNELVRVY
VAQKRKIQDGDKLAGRHGNKGVISKILPIEDMPFLEDGTPVDIVLNPLGVPSRMNIGQVLETHLGWVAKT
GWSVDGDDAEWKRQLRSINAHESEPDTNVATPVFDGAREEEISGLLASTLPNRDGNQLIGGSGKAQLFDG
RSGEPLPDPIAVGYVYILKLNHLVDDKIHARSTGPYSMITQQPLGGKAQFGGQRFGEMECWAMQAYGAAY
ALQELLTIKSDDVLGRVKVYEAIVKGENIPEPGIPESFKVLLKELQSLCLNVEVLSSDGVALEMRETDDE
VFRAAEELGIDLSRREPSSVEEV
>gi|238061130|ref|ZP_04605839.1| DNA-directed RNA polymerase, beta subunit [Micromonospora sp. ATCC 39149]
MAASRPAKTSRTSSAFAPRRVSFGRITEHLEVPNLLAIQNESFDWLVGNEAWQGRSADDPHARSGLAEIL
DEISPIEDFSGTMSLSFSAPRFDEVKASIEECKEKDLTYCAPLFVTAEFTNNTTGEIKSQTVFMGDFPMM
TPKGTFIINGTERVVVSQLVRSPGVYFDKQPDKTSDRDLSSVKVIPSRGAWLEFDIDKRDTVGVRIDRKR
RQAVTVLLKAIGWSAERIREKFGWSELLMTTLEKDHIAGQDEALLDIYRKLRPGEPPTRENAQTLLDNLF
FNPKRYDVAKVGRYKFNKKLELSVPITTGTLTEDDIVATVEYLCRLHAGEDGYEADDIDHFGNRRLRTVG
ELIQNQVRVGLSRMERVVRERMTTQDVEAITPQTLINIRPVVAAIKEFFGTSQLSQFMDQTNPLAGLTHR
RRLSALGPGGLSRERAGFEVRDVHPSHYGRMCPIETPEGPNIGLIGALSTFARVNPFGFIETPYRKVVDG
RVTDQIDYLTADEEDRFVKAQANAPLKADGTFVEDRVLVRRKGGETEDVAPGAVDYMDVSPRQMTSVATA
MIPFLEHDDANRALMGANMQRQAVPLVKAESPLVGTGMEYRAAVDAGDVVVAEVGGVIEDLCADYITIHQ
DDGHRRTYLLHKFRRSNAGSCVNQKPVVFEGDRVEAGQVIADGPCTDEGEMALGRNLLVAFMCWEGHNYE
DAIILSQRLVQQDVLTSIHIEEHEVDARDTKLGPEEITRDIPNVSEEMLADLDERGIIRIGAEVVPGDIL
VGKVTPKGETELTPEERLLRAIFGEKAREVRDTSLKVPHGETGTVIGVRTFSREDGDELPPGVNELVRVY
VAQKRKIQDGDKLAGRHGNKGVISKILPIEDMPFLEDGTPVDIVLNPLGVPSRMNIGQVLETHLGWVAKT
GWSVDGDDAEWKRQLRSIEAHESEPDTNVATPVFDGAREEEISGLLASTLPNRDGKQLIGSSGKAQLFDG
RSGEPLPDPIAVGYIYILKLNHLVDDKIHARSTGPYSMITQQPLGGKAQFGGQRFGEMECWAMQAYGAAY
ALQELLTIKSDDVLGRVKVYEAIVKGENIPEPGIPESFKVLLKELQSLCLNVEVLSSDGVALEMRETDDE
VFRAAEELGIDLSRREPSSVEEV
>gi|389866528|ref|YP_006368769.1| DNA-directed RNA polymerase subunit beta [Modestobacter marinus]
MAGSRPTSTTTTDSSTTPVDVQPGPRTGGTDQQKIPGAPLRVSFAKIREPLEVPDLLALQTASFDWLVGS
PQWKATLAPEEQADAVGGLAEILEEISPIEDFSGSMSLSFSNPRFEDVKASLEECKDKDMTYAAPLFVTA
EFMNNTTGEIKSQTVFMGDFPIMTSKGTFVINGTERVVVSQLVRSPGVYFDKTLDKTSDKDVYSAKVIPS
RGAWLEFDVDKRDTVGVRIDRKRRQPVSVLLKALGWTEDRIREHFQWSPTMLATLEKDHIAGQDEALLDI
YRKLRPGEPPTRESAQALLENLFFNPKRYDLAKVGRYKVNKKLGVEVPQGTSTLTEDDIVATIEYVVRLH
AGEPEHGVDDIDHFGNRRLRTVGELIQNQIRVGLSRMERVVRERMTTQDVEAITPQTLINIRPVVASIKE
FFGTSQLSQFMDQTNPLAGLTHKRRLSALGPGGLSRERAGMEVRDVHPSHYGRMCPIETPEGPNIGLIGS
LSSFGRVNPFGFIETPYRKVENGQVTDQIDYLTADEEDRFVVAQANSPLDAQGRLAEERVLVRTKGGEVD
YLAPENVDYMDVSPRQMTSVATAMIPFLEHDDANRALMGANMQRQAVPLLRSEAPLVGTGMELRAAVDAG
DVVVAEKAGVVEDSTADYVTVMADDGTRQTYRLLKFRRSNQGTSINQSPVVQEGQRVEVGQVIADGPCTD
EGEMALGKNLLVAFMPWEGHNYEDAIILSQRLVQDDVLSSIHIEEFEVDARDTKLGAEEITRDIPNVSEE
VLADLDERGIIRIGAEVVPGDILVGKVTPKGETELTPEERLLRAIFGEKAREVRDTSLKVKHGESGKVIG
VRVFSREDGDELPAGVNELIRVYVAQMRKISDGDKLAGRHGNKGVISKILPQEDMPFLEDGTPVDIVLNP
LGVPGRMNVGQVLETHLGWIAKQGWEVEGTPEWAAKLPGAARQAAPGTRTATPVFDGAKEDEIIGLLGST
IPNRDGDRMVKETGKARLFDGRSGEPFPEPISVGYVYILKLLHLVDDKIHARSTGPYSMITQQPLGGKAQ
FGGQRFGEMECWAMQAYGAAYALQELLTIKSDDILGRVKVYEAIVKGENIPEPGIPESFKVLLKELQSLC
LNVEVLSGDGQAIELRDTDDEVFRAAEELGIDLSRREPSSVEDV

>gi|258651304|ref|YP_003200460.1| DNA-directed RNA polymerase subunit beta [Nakamurella multipartita DSM 44233]
MAVTSTSRSTASSPNRVSFAKLREPLEVPNLLALQTDSFAWLVGGADWRARQDEPTPSGLDEILEEISPI
EDFSGNLSLSFSDPRFDEVKANEEECRDKDMTYSAPLFVTAEFMNGETGEIKSQTVFMGDFPMMTSKGTF
IINGTERVVVSQLVRSPGVYFDKSLDKTAGKDVFSCKVIPSRGAWLEFDVDKRDTIGVRIDRKRRQPVSV
LLKALGWDTERIREHFSWSPVLLATLEKDHIAGQDEALLDIYRKLRPGEPPTKESAQALLDNLFFKDKRY
DLAKVGRYKMNKKLGVNQPTSVGTLTEDDLLSIVEYLLRLHQGDAEWTKNGRVYPVETDDIDHFGNRRLR
TVGELIQNQIRVGLSRMERVVRERMTTQDVEAITPQTLINIRPVVASIKEFFGTSQLSQFMDQTNPLAGL
THKRRLSALGPGGLSRDRASLEVRDVHPSHYGRMCPIETPEGPNIGLIGSLSTFARVNPFGFIETPYRKV
IDGVATDQIEYLTADEEDRFIIAQASAKLDNDGRFTENRVLVRRKGSGEVDQVPPAEVEYMDVSPRQTVS
VATAMIPFLEHDEANRALMGANMQRQSVPLLRSEAPLVGTGMEARAAKDAGDVLIAEKSGVVEEATADYI
SVMNDDGTHRTYRLNKFRRSNQGTSFNQKPIVDEGARVEAGDVIADGPCTDGGEMALGKNLLVAFMPWEG
HNYEDAIILSQRIVQDDVLTSIHIEEHEIDARDTKLGPEEITRDIPNVSEEVLADLDERGIVRVGAEVQD
GDILVGKVTPKGETELTPEERLLRAIFGEKAREVRDTSLKVPHGETGKVIGIRVFNRDDDGAELPPGVNE
LVRVYVAQKRKIQDGDKLAGRHGNKGVIGKILPVEDMPFLPDGTPVDIVLNPHGVPRRMNIGQILETHLG
WVASQGWQVEGEPGWAKNLSEQTREAGPWTKTATPVFSGASEDEIMGLLGSTTPTRDGERLVHTDGKATL
FDGRTGEPFPYPVAVGYMYIIKLLHLVDDKIHARSTGPYSMITQQPLGGKAQFGGQRFGEMECWAMQAYG
AAYTLQELLTIKSDDIVGRVKVYEAIVKGENIPEPGIPESFKVLLKELQALCLNVEVLSSDGVAIEMRDS
DDDDLERAAANLGINLSRNESASVDDLVN
>gi|326333393|ref|ZP_08199639.1| DNA-directed RNA polymerase, beta subunit [Nocardioidaceae bacterium Broad-1]
MAARTSSGDSTPRRISFAKISEPLELPQLLALQTESFDWLIGNERWQEQVAAREAAGEDVSHKSGLQEIF
EEISPIEDFSETMSLSFESPVFYDPKYTVEECKEKDFTYSAPLYVSAEFTNNDTGEIKGQTVFMGDFPLM
TDQGTFVINGTERVVVSQLVRSPGVYFERTADKTSDKDIYTAKLIPSRGAWLEFEIDKRDLVGVRLDRKR
KQNVTVLLKALGWTNEQIREEFGEYESMMLTLEKDSTTAQDDALLDIYRKLRPGEPPTREAAQTLLNNYY
FNPKRYDTAKVGRYKVNKKLGLTEAFDQQTLTVDDIVATIKYVVALHDGRTQLETPQGTIDIAADDIDHF
GNRRMRTVGELIQNQLRTGLARMERVVRERMTTQDVEAITPQSLINIRPVVAALKEFFGTSQLSQFMDQN
NPLAGLTHKRRLSALGPGGLSRDRAGMEVRDVHPSHYGRMCPIETPEGPNIGLIGSLASFGRINPFGFVE
TPYRKVVNGVVTDQIDYLTADDEDKYVIAQANAAVDENMKFATERVLVRQRDGEVSELLADEVDYIDVSP
RQMVSVATALIPFLEHDDANRALMGANMQRQAVPLVRSDSPLVGTGIEYRAAVDAGDVLVAKKAGVVKEV
SADVIETLNDDGTYTTYKLAKFERSNQGNCVNQQPLVDEGARVEVGSPLADGPATQGAEMALGANLLVAF
QSWQGHNYEDAIILSQRLVQQDYLTSIHIEEHEVDARDTKLGPEEITRDIPNVSEEMLADLDERGIIRIG
AEVTTGDILVGKVTPKGETELTPEERLLRAIFGEKAREVRDTSMKVPHGEEGTVIGVRVFDREEGDELPP
GVNQLVRVYVAQKRKISVGDKLAGRHGNKGVIAKILPVEDMPFMEDGTPVDVLLNPLGVPRRMNIGQVLE
LHLGWLAKQGWDLNLSDDPDDSAWKQRLIKIHADKADPGTKVATPVFDGAREDEITGLLASTLPNRDGQR
VVGTDGKARLFDGRSGEPFPEPVAVGYMYILKLHHLVDDKIHARSTGPYSMITQQPLGGKAQFGGQRFGE
MEVWAMEAYGAAYALQELLTIKSDDVPGRVKVYEAIVKGENIPDSGIPESFKVLVKEMQSLCLNVEVLSQ
DGSAIELRDAEEDVFRAAEELGIDLSRREPSSVEEV
>gi|402802503|gb|AFR09913.1| DNA-directed RNA polymerase, beta subunit [Nocardiopsis alba ATCC BAA-2165]
MQTESFDWLLGNDTWKTRVETARNAGRKDVPEQSGLEEIFEEISPIEDFSGTMSLSFRDHRFEPPKYSEE
ECKDKDMTFSAPMFVTAEFINNDTGEIKSQTVFMGDFPLMTAKGTFIINGTERVVVSQLVRSPGVYFDKS
VDKTSDKDLFGCKVIPSRGAWLEFEVDKRDFVGVRIDRKRKQGVTVLLKALGWTTDQILERFGQYESIRN
TLEKDPTAGTDDALLDIYRKLRPGEPPTKESAQALLENLYFNPKRYDLAKVGRYKINKKLGLDTDYTQGT
LTEEDIVATIDYLVRLHAGEVEKETVFGLRPIETDDIDHFGNRRLRTVGELIQNQVRLGLARMERVVRER
MTTQDVEAITPQTLINIRPVVASIKEFFGTSQLSQFMDQTNPLAGLTHKRRLSALGPGGLSRERAGFEVR
DVHPSHYGRMCPIETPEGPNIGLIGSLAAYGRVNSFGFVETPYRKVIDGRISEQIDYLTADEEDLYVIAQ
ANTPLNADGSFAESGVLVRRKGGEFEQVDTSEVDYMDVSPRQMVSVATAMIPFLEHDDANRALMGSNMQR
QAVPLLRAEAPFVGTGMEYRAATDAGEVILAEKAGVVEDVTADYVTVMADDGTRKTYRMGKFQRSNQGTC
FNQRPIVHEGQRVEVKQVLADGPSTDQGEMSLGKNLLVAYMSWEGHNYEDAIILSQRLVQDDVLSSIHIE
EHEVDARDTKLGPEEITREIPNVSEEVLADLDDRGIIRIGAEVVDGDILVGKVTPKGETELTPEERLLRA
IFGEKAREVRDTSLKVPHGETGKVIGVRVFSREEGDELAPGVNEMVRVYVAQKRKITDGDKLAGRHGNKG
VIAKILPQEDMPFLEDGTPVDIILNPLGVPGRMNVGQVLEVHLGWLAKNGWVVEGAEEEWQKSLHAIGAS
DVEPNSRVATPVFDGLHGDELSGLIKSVRPNKDGNRLINDDGKARLFDGRTGEPFAEPISVGYKYILKLH
HLVDDKIHARSTGPYSMITQQPLGGKAQFGGQRFGEMEVWALEAYGAAYALQELLTIKSDDVVGRVKVYE
AIVKGENIPEPGIPESFKVLIKEMQSLCLNVEVLSSDGMSIEMRDSDEDVFRAAEELGIDLGRREPSSVE
EV

>gi|297564030|ref|YP_003683003.1| DNA-directed RNA polymerase subunit beta [Nocardiopsis dassonvillei subsp. dassonvillei DSM 43111]
MAASRNASANALGPHRVSFARIQEPLEVPNLLALQTESFDWLLGNDRWKTRVETARNAGRKDVPEQSGLE
EIFEEISPIEDFSGTMSLSFRDHRFEPPKYSEDECKDRDMTYSAPMFVTAEFINNDTGEIKSQTVFMGDF
PLMTETGTFIINGTERVVVSQLVRSPGVYFDKSVDKTSDKDLYGCKVIPSRGAWLEFEVDKRDFVGVRID
RKRKQGVTVLLKALGWTTDQILERFGQYESIRNTLEKDPTAGTDDALLDIYRKLRPGEPPTKESAQALLE
NLYFNPKRYDLAKVGRYKINKKLGLDTDYRQGTLTEEDIVATIDYLVRLHAGETEKETVNGPRPIETDDI
DHFGNRRLRTVGELIQNQVRLGLARMERVVRERMTTQDVEAITPQTLINIRPVVASIKEFFGTSQLSQFM
GQTNPLEGLTHKRRLSALGPGGLSRERAGFEVRDVHPSHYGRMCPIETPEGPNIGLIGSLAAYGRVNSFG
FVETPYRRIIDGKVSDQVDYLTADEEDLHVIAQANTPMNPDGSFAEAGVLVRRKGGEFEQVSTDEVDYMD
VSPRQMVSVATAMIPFLEHDDANRALMGSNMQRQAVPLLMAESPFVGTGMEYRAATDAGEVVLAQKAGVV
EDVTADYVTVMADDGTRKTYRMGKFQRSNQGTCFNQRPIVAEGQRVEERQVLADGPSTDQGEMSLGKNLL
VAYMSWEGHNYEDAIILSQRLVQDDVLSSIHIEEHEVDARDTKLGPEEITREIPNVSEEVLADLDDRGII
RIGAEVVDGDILVGKVTPKGETELTPEERLLRAIFGEKAREVRDTSLKVPHGETGKVIGVRVFSREDGDE
LAPGVNEMVRVYVAQKRKITDGDKLAGRHGNKGVISKILPQEDMPFLEDGTPVDIILNPLGVPGRMNVGQ
VLEVHLGWLAKNGWLVEGVEEEWQKSLEAIGATDVPPDSRVATPVFDGLRGDELSGLIKSVRPNADGNRL
INEDGKARLFDGRTGEPFAEPISVGYKYILKLHHLVDDKIHARSTGPYSMITQQPLGGKAQFGGQRFGEM
EVWALEAYGAAYALQELLTIKSDDVVGRVKVYEAIVKGENIPEPGIPESFKVLIKEMQSLCLNVEVLSSD
GMSIEMRDSDEDVFRAAEELGIDLGRREPSSVEEV
>gi|119714958|ref|YP_921923.1| rpoB gene product [Nocardioides sp. JS614]
MQIEERIVEGHLLAARTTPGNTRRISFAKITEPLEVPQLLSLQTDSFDWLVGNDKWNAAVERRRSEGEDV
SSKSGLQEIFEEISPIEDFSETMSLSFENPVFYDPKYTVDECKEKDFTYSAPLYVSAEFTNNDTGEIKGQ
TVFMGDFPLMTDKGTFIINGTERVVVSQLVRSPGVYFERTADKTSDKDIYTAKLIPSRGAWLEFEIDKRD
MVGVRLDRKRKQNVTVLLKALGWTNEQIREEFGEYESMMLTLEKDHTQGQDDALLDIYRKLRPGEPPTRE
AAQTLLNNYYFNPKRYDLAKVGRYKINKKLGLLEAFDQQTLTIDDVVAAIKYIVALHDGREQIEAPQGTL
DISADDIDHFGNRRMRTVGELIQNQLRTGLARMERVVRERMTTQDVEAITPQSLINIRPVVAALKEFFGT
SQLSQFMDQTNPIAGLTHKRRLSALGPGGLSRDRAGMEVRDVHPSHYGRMCPIETPEGPNIGLIGSLASY
GRINPFGFVETPYRKVTKGKVTDQIDYLTADDEDRYVIAQANAALDDKSRFVEERVLVRQRDGEVSEVLA
DEVDYMDVSPRQMVSVATALIPFLEHDDANRALMGANMQRQAVPLIKSDSPLVGTGIEYRAAVDAGDVVV
ATAAGVVKEVSADVIETMNDDGTYSTYRLAKFKRSNQGTCINQRPLVSEGDRLEIGTPIADGPCTDEAEM
ALGTNLLVAFMPWQGHNYEDAIILSQRLVQEDILTSIHIEEHEVDARDTKLGPEEITRDIPNVSEEMLAD
LDERGIIRIGAEVTTGDVLVGKVTPKGETELTPEERLLRAIFGEKAREVRDTSMKVPHGESGTVIGVRVF
DREDGDELPPGVNQLVRVYVAQKRKISVGDKLAGRHGNKGVIAKILPIEDMPFMEDGTPVDVVLNPLGVP
RRMNIGQILELHLGWLAKQGWDLNLNDDKSGADWKQRLIKIHADKAEPGTKVATPVFDGAREDEITGLLG
STIPNRDGVRMIDNTGKASLFDGRSGEPFPEPVAVGYMYILKLHHLVDDKIHARSTGPYSMITQQPLGGK
AQFGGQRFGEMEVWAMEAYGAAYALQELLTIKSDDVPGRVKVYEAIVKGENIPDSGIPESFKVLVKEMQS
LCLNVEVLSQDGTAIEMRDAEEDVFRAAEELGIDLSRREPSSVEEV
>gi|331699183|ref|YP_004335422.1| DNA-directed RNA polymerase subunit beta [Pseudonocardia dioxanivorans CB1190]
MAVSRATVTTATPGSAVSANPSFPGAPTRVSFAKIREPLEVPDLLDLQIQSFEWLVGSEAWFQRRIDAGD
DNPVGGLEEILAEISPIEDFSGSMSLSFSDPRFDEVKASVEECRDKDMTYGAPLFVTAEFTNNTTGEIKS
QTVFMGDFPVMTDKGTFIINGTERVVVSQLVRSPGVYFDHAIDKTTEKDVYSVKIIPSRGAWLEFDVDKR
DTVGVRIDRKRRQPVTVLLKALGWTNEQISERFGFSETIMATLEKDHTAGQDEALLDIYRKLRPGEPPTR
ESAQTLLENLFFKDKRYDLAKVGRYKANKKLGLAIDNSVGTLTEEDIATTIEYLVRLHAGETSMAGGGNG
TAGIPGEVVPVETDDIDHFGNRRLRTVGELIQNQVRVGLSRMERVVRERMTTQDVEAITPQTLINIRPVV
AAIKEFFGTSQLSQFMDQHNPLAGLTHKRRLSALGPGGLSRERAGFEVRDVHPSHYGRMCPIETPEGPNI
GLIGSLSTFAQVNPFGFIQTPYRKVVDGVVTEQIDYLTADEEDRFVIAQANAPLNDDGSFTEDRVLVRRK
GGEVDLIPPTGVEYIDVSPRQMVSVATALIPFLEHDDANRALMGANMQRQSVPLLRSEAPLVGTGMELRA
AVDAGDVLVAEKAGVVEELCADYITVMADEGTRQTYRLNKFRRSNQGTCNNQKPIVDEGQRVEVGQVLAD
GPCTENGEMALGKNLLVAIMPWEGHNYEDAIILSQRLVQDDVLTSIHIEEHEIDARDTKLGAEEITRDIP
NVSEEVLADLDERGIIRIGAEVQPGDILVGKVTPKGETELTPEERLLRAIFGEKAREVRDTSLKVPHGEN
GKVIGIRVFSRDDEDELAPGVNELVRVYVAQKRKIQDGDKLAGRHGNKGVIGKILPAEDMPFLPDGTPVD
IILNTHGVPRRMNIGQVLETHLGWIAKSGWEIEGTPEWASKMPEELYSVAPNTNTATPVFDGAKEHEITG
LLASTRPNRDGDRMVGPDGKAQLFDGRSGEPYPFPVAVGYMYILKLAHLVDDKIHARSTGPYSMITQQPL
GGKAQFGGQRFGEMECWAMQAYGAAYTLQELLTIKSDDVVGRVKVYEAIVKGENIPEPGIPESFKVLLKE
LQSLCLNVEVLSSDGAAIEMRDSDDEDLERAAANLGINLSSRPGSDSITVDDVVN

>gi|418460788|ref|ZP_13031875.1| DNA-directed RNA polymerase subunit beta [Saccharomonospora azurea SZMC 14600]
MQIRSFEWFTGSEAWYERAVNEGEENPVGGLEEVLNEISPIEDFSGSMSLSFSDPRFDEVKASVEECKDK
DMTYAAPLFVTAEFVNNNTGEIKSQTVFMGDFPVMTDKGTFIINGTERVVVSQLVRSPGVYFDQSVDKST
DKDVFSVKIIPSRGAWLEFDVDKRDTVGVRIDRKRRQPVTVLLKALGWTAEQIRERFSFSETLLATLEKD
STATQDEALLDIHRKLRPGEPPTKESAQALVENLFFKDKRYDLAKVGRYKVNKKLGLSLPYDTGTLTEDD
IVATIEYLVRLHAGEETMRVGDVEVPVETDDIDHFGNRRIRTVGELIQNQIRVGLSRMERVVRERMTTQD
VEAITPQTLINIRPVTAAIREFFGTSQLSQFMDQNNPLSGLTHKRRLNALGPGGLSRERAGMDVRDVHPS
HYGRMCPIETPEGPNIGLIGSLSSYARVNPFGFIETPYRKVVDGKVTDEVHYLTADEEDRFVKAQANAPL
TEDGHFMEEQVLGRRKGGEVELIDPMDVDYMDVSPRQMVSVATAMIPFLEHDDANRALMGANMQRQAVPL
LQAESPLVGTGVELAAAVDAGDVIVAEEAGVVEELSADMITVMQDDGTRRTYGLYKFRRTNAGTCFNHRP
VVDEGDRVEKGQVLADGPSTDKGEMALGKNLLVAIMPWEGHNYEDAIVISQRLVQDDVLTSIHIEEHEID
ARDTKLGAEEITRDIPNVSEDVLADLDERGIVRIGAEVRDGDILVGKVTPKGETELTPEERLLRAIFGEK
AREVRDTSLKVPHGETGKVIGVRVFSREDDDELPPGVNELVRVYVAKKSKIQDGDKLAGRHGNKGVIGKI
LPVEDMPFLEDGTPVDIVLNTHGVPRRMNIGQILELHLGWLASQGWKVEGNPEWAKNLTEELMDVEPGTN
TATPVFDGAREHELTGLLGCAKPNRDGDRLVADDGKATLLDGRTGEPFPYPVAVGYMYILKLHHMVDDKI
HARSTGPYSMITQQPLGGKAQFGGQRFGEMECWAMQAYGAAYTLQELLTIKSDDVLGRVKVYEAIVKGEN
MPSPGIPESFKVLLKELQSLCLNVEVLSTDGAAIEMRDSDDEDLERAAANLGINLSRNESPSVDDVVQ
>gi|257054452|ref|YP_003132284.1| DNA-directed RNA polymerase subunit beta [Saccharomonospora viridis DSM 43017]
MSPEGRILAVSPANQASAATNSNRPSTGIPGAPKRVSFGEIREPLGIPNLLDVQIQSFEWFTGSEAWYERA
VNEGEENPVGGLEEVLNEISPIEDFSGSMSLSFSDPRFDEVKASVEECKDKDMTYAAPLFVTAEFVNHNTG
EIKSQTVFMGDFPVMTDKGTFIINGTERVVVSQLVRSPGVYFDQSIDKSTDKDVFSVKIIPSRGAWLEFDV
DKRDTVGVRIDRKRRQPVTVLLKALGWTNEQIRERFSFSETLLATLEKDPTTTQDEALLDIHRKLRPGEPP
TKESAQALVENLFFKEKRYDLAKVGRYKVNKKLGLNIPYDVGTLTEEDIVATIEYLVRLHAGEETMKVGDV
EVPVEVDDIDHFGNRRVRTVGELIQNQIRVGLSRMERVVRERMTTQDVEAITPQTLINIRPVVAAIREFFG
TSQLSQFMDQNNPLSGLTHKRRLNALGPGGLSRERAGMDVRDVHPSHYGRMCPIETPEGPNIGLIGSLASH
ARVNPFGFIETPYRKVVNGRVTDEVHYLTADEEDRFVKAQANAPLTEDGYFAEEKVLGRRKGGEVELLDPM
EIDYMDVSPRQMVSVATAMIPFLEHDDANRALMGANMQRQAVPLLRSEAPLVGTGVELPAAVDAGDVVVAE
ESGVVEELSADMITIMQDDGTRRSYGLYKFRRTNAGTCFNHRPIVNEGDRVERGQVIADGPSTDKGEMALG
KNLLVAIMPWEGHNYEDAIVISQRLVQDDVLTSIHIEEYEIDARDTKLGAEEITRDIPNVSEDVLADLDER
GIVRIGAEVRDGDILVGKVTPKGETELTPEERLLRAIFGEKAREVRDTSLKVPHGETGKVIGVRVFSREED
DELPPGVNELVRVYVAKKSKIQDGDKLAGRHGNKGVIGKILPVEDMPFLPDGTPVDVVLNTHGVPRRMNIG
QIMELHLGWLASQGWKVEGNPEWAKNLTEELRDVEPGTNTATPVFDGAREEEITGLLGCTKPNRDGQRLVG
PDGKATLFDGRTGEPFPYPVAVGYMYILKLHHMVDEKIHARSTGPYSMITQQPLGGKAQFGGQRFGEMECW
AMQAYGAAYTLQELLTIKSDDVVGRVKVYEAIVKGENMPSPGIPESFKVLLKELQSLCLNVEVLSSDGASI
EMRDSDDEDLERAAANLGINLSRNESPSVDDVVQ
>gi|134103282|ref|YP_001108943.1| DNA-directed RNA polymerase subunit beta [Saccharopolyspora erythraea NRRL 2338]
MAVSRATKVSAASNMMSGIPGAPKRVSFANIREPLEVPDLLDLQVQSFEWLVGDETWFQRRVDAGDENPV
GGLEEVLSEISPIEDFSGSMSLSFSDPRFDEVKASVEECKDKDMTYAAPLFVTAEFTNHTTGEIKSQTVF
MGDFPVMTDKGTFIINGTERVVVSQLVRSPGVYFDQSVDKTTDKDVYSVKIIPSRGAWLEFDVDKRDTVG
VRIDRKRRQPVTVLLKALGWTAEAIRERFGFSETLMATLEKDHTAGQDEALLDIYRKLRPGEPPTKESAQ
TLLENLFFKEKRYDLARVGRYKVNKKLGLDLPFESGVLTEEDIVTTIEYLVRLHAGEAEMPDRGQGGDAT
IPVEVDDIDHFGNRRLRTVGELIQNQVRVGLSRMERVVRERMTTQDVEAITPQTLINIRPVVAAIKEFFG
TSQLSQFMDQTNPIAGLTHKRRLSALGPGGLSRERAGMEVRDVHPSHYGRMCPIETPEGPNIGLIGSLAT
FARVNPFGFIETPYRKVVDGRVTDQIDYLTADEEDRFVKAQANTPIDDEGNFLADRVLGRRKGGEVELLA
PTEIDYMDVSPRQMVSAATAMIPFLEHDDANRALMGANMQRQAVPLLRSEAPLVGTGMELRAAVDAGDVV
TAEKAGVIEELCADYITVMADDGTRRTYRLQKFSRSNHGTCTNQKPIVNEGDRVEAGQVIADGPCTQNGE
MALGKNLRVAIMPWEGHNYEDAIILSQRLVQDDVLTSIHIEEHEVDARDTKLGAEEITRDIPNVSEDVLA
DLDERGIIRIGAEVQGGDILVGKVTPKGETELTPEERLLRAIFGEKAREVRDTSLKVPHGETGKVIGVRV
FNREDDDELPPGVNELVRVYVAQKRKIQDGDKLAGRHGNKGVIGKILPAEDMPFTEDGTPVDIILNTHGV
PRRMNIGQILETHLGWIASQGWSIDGDPEWAKRLPEELYDVDPGTNTASPVFDGAREEEITGLLASTKPN
RDGERMVKANGKAQLFDGRSGEPYPYPVAVGYMYILKLSHLVDDKIHARSTGPYSMITQQPLGGKAQFGG
QRFGEMECWAMQAYGAAYTLQELLTIKSDDVVGRVKVYEAIVKGENIPEPGIPESFKVLLKELQSLCLNV
EVLSSDGAAIEMRDGDDEDLERAAANLGINLSRNESPSVDDVVQ
>gi|348176452|ref|ZP_08883346.1| DNA-directed RNA polymerase subunit beta [Saccharopolyspora spinosa NRRL 18395]

MAVSRATKVSAASNYTSGIPGAPKRVSFANIREPLNVPNLLDLQIQSFEWLVGNEAWFQRRVDAGEENPV
GGLEEVLNEISPIEDFSGSMSLSFSDPRFDEVKASVEECKDKDMTYAAPLFVTAEFTNHTTGEIKSQTVF
MGDFPMMSNKGTFIINGTERVVVSQLVRSPGVYFDQSVDKTTDKDVFSVKIIPSRGAWLEFDVDKRDTVG
VRIDRKRRQPVTVLLKALGWSAEAIRERFGFSETLMATLEKDHTAGTDEALLDIYRKLRPGEPPTKESAQ
TLLENLFFKEKRYDLARVGRYKVNKKLGMGEPYEVGVLTEEDIVTTIEYLVRLHAAETEMPARGQGEGAT
VPVEVDDIDHFGNRRIRTVGELIQNQVRVGLSRMERVVRERMTTQDVEAITPQTLINIRPITAAIREFFG
TSQLSQFMDQTNPIAGLTHKRRLNALGPGGLSRERAGMEVRDVHPSHYGRMCPIETPEGPNIGLIGSLAT
FARINPFGFIETPYRKVVDGRVTDQIDYLTADEEDRYVKAQANAPIDEDGNFIDERVLGRRKGGEVELLA
PTEIDYMDVSPRQMVSAATAMIPFLEHDDANRALMGANMQRQAVPLLRSESPLVGTGMELRAAVDAGDVI
TAEKAGVVEELCADYITVMADDGTRRSYRMQKFSRSNHGTCINQRPIVNEGDRVEVGQVIADGPCTEDGE
MALGKNLLVAIMPWEGHNYEDAIILSQRLVQDDVLTSIHIEEHEVDARDTKLGAEEITRDIPNVSDDVLA
DLDERGIIRIGAEVQGGDILVGKVTPKGETELTPEERLLRAIFGEKAREVRDTSLKVPHGETGKVIGVRV
FNREDDDELPPGVNQLVRVYVAQKRKIQDGDKLAGRHGNKGVIGKILPAEDMPFLPDGTPVDIILNTHGV
PRRMNIGQVLETHLGWIASQGWSINGDAEWAKRLPDDLYEVEPGTNTASPVFDGAREEEITGLLASTRPN
RDGERMVGADGKAQLFDGRSGEPYPYPIAVGYMYILKLSHLVDDKIHARSTGPYSMITQQPLGGKAQFGG
QRFGEMECWAMQAYGAAYTLQELLTIKSDDVVGRVKVYEAIVKGENIPEPGIPESFKVLLKELQSLCLNV
EVLSSDGAAIEMRDSEDEDLERAAANLGINLSRNESASVDDLAH
>gi|433609674|ref|YP_007042043.1| DNA-directed RNA polymerase subunit beta [Saccharothrix espanaensis DSM 44229]
MAISRATKATAATNSTSGIPGAPKRVSFAKIHEPLQTPNLLDLQIQSFEWLTGDEAWFQRRVDEGDETPT
GGLEEVLNEISPIEDFSGSMSLSFSDPRFDEVKASTEECKDKDMTYAAPLFVTAEFTNHTTGEIKSQTVF
MGDFPMMTDKGTFIINGTERVVVSQLVRSPGVYYDTAIDKTTDKDVFSVKIIPSRGAWLEFDVDKRDTVG
VRIDRKRRQPVTVLLKALGWSTEQIRERFSFSETLLTTLEKDHTAGTDEALLDIYRKLRPGEPPTKESAQ
ALLENLFFKDKRYDLAKVGRYKINKKLGLASPIATGVLTEDDIVTTIEYLVRLHAGETSMKPGETEVPVE
VDDIDHFGNRRLRTVGELIQNQIRVGLSRMERVVRERMTTQDVEAITPQTLINIRPVVAAIKEFFGTSQL
SQFMDQTNPLAGLTHKRRLSALGPGGLSRERAGMEVRDVHPSHYGRMCPIETPEGPNIGLIGSLSSYGRV
NPFGFIETPYRKVIEGRVTDQIDYLTADEEDRFVKAQANAKIDNEGNFLEAKVLVRKKGGEVEQIDPLDV
DYMDVSPRQMVSAATAMIPFLEHDDANRALMGANMQRQAVPLLRSESPLVGTGMELRAAVDAGDVVVAKK
TGAVEEISADYVTVMADDGSRQTYGLHKFRRSNQGTCINQKPIVNEGDRVREGQVLADGPCTENGEMALG
KNLLVAIMPWEGHNYEDAIILSQRLVQDDVLTSIHIEEHEIDARDTKLGAEEITRDIPNVSEEVLADLDE
RGIIRIGAEVQPGDILVGKVTPKGETELTPEERLLRAIFGEKAREVRDTSLKVPHGEYGKVIGIRVFSRE
DDDELPPGVNELVRVYVAQKRKIQDGDKLAGRHGNKGVIGKILPVEDMPFLEDGTPVDIVLNTHGVPRRM
NIGQVLETHLGWIAKQGWSINGDPDWAKNLPGELYDVEPGTKTATPVFDGAREDEITGLLGSTVPNRDGE
RMVKENGKAQLFDGRSGEPYPFPVSVGYMYILKLLHLVDDKIHARSTGPYSMITQQPLGGKAQFGGQRFG
EMECWAMQAYGAAYTLQELLTIKSDDVLGRVKVYEAIVKGENIPEPGIPESFKVLLKELQSLCLNVEVLS
SDGAAIEMRDGDDEDLERAAANLGINLSRSESPSVDDVVN
>gi|159039844|ref|YP_001539097.1| rpoB gene product [Salinispora arenicola CNS-205]
MAASRPAKTSRTSSAFAPRRVSFGRITEHLEVPNLLAIQTESFDWLVGNEAWQGRSADDPHARSGLAEIL
DEISPIEDFSGTMSLSFSAPRFDEVKASIEECKEKDLTYCAPLFVTAEFTNNTTGEIKSQTVFMGDFPMM
TPKGTFIINGTERVVTSQLVRSSGVYFDKQPDKTSDRDLSSVKVIPSRGAWLEFDIDKRDTVGVRIDRKR
RQAVTVLLKAIGWSAEQIREKFGWSELMMTTLEKDHIAGQDEALLDIYRKLRPGEPPTRENAQTLLDNLF
FNPKRYDVAKVGRYKFNKKLDLDLPIDTGVLTQDDIVATVEYLCRLHAGEEGYEADDIDHFGNRRLRTVG
ELIQNQVRVGLSRMERVVRERMTTQDVEAITPQTLINIRPVVAAIKEFFGTSQLSQFMDQTNPLAGLTHR
RRLNALGPGGLSRERAGFEVRDVHPSHYGRMCPIETPEGPNIGLIGNLSTFARVNPFGFIETPYRKVVDG
RVTDQVDYLTADDEDRFVKAQANAPLNADGSFAEDRVLVRRKGGETEDVAPSAVDYMDISPRQMVSVGTA
MIPFLEHDDANRALMGANMQRQAVPLVKAEAPLVGTGMEYRAAVDAGDVVVAEVGGVIEDLCADYVTIHQ
DDGHRRTYLLHKFRRSNSGSCVNQKPVVFEGDRVEAGQVIADGPCTDEGEMALGRNLLVAFMTWEGYNYE
DAIILSQRLVQQDVLTSIHIEEHEVDARDTKLGPEEITRDIPNVSEEMLADLDERGIIRIGAEVVPGDVL
VGKVTPKGETELTPEERLLRAIFGEKAREVRDTSLKVPHGETGTVIGVRTFSREDGDELPPGVNELVRVY
VAQKRKIQDGDKLAGRHGNKGVISKILPVEDMPFLEDGTPVDIVLNPLGVPSRMNIGQVLETHLGWVAKT
GWKVEGDDAEWKRQLQAIGADESEPDSNVATPVFDGAREEEISGLLASTRSNRDGKQLIGSSGKAQLFDG
RSGEPLPDPIAVGYIYILKLNHLVDDKIHARSTGPYSMITQQPLGGKAQFGGQRFGEMECWAMQAYGAAY
ALQELLTIKSDDVLGRVKVYEAIVKGENIPEPGIPESFKVLLKELQSLCLNVEVLSSDGVALEMRETDDE
VFRAAEELGIDLSRREPSSVEEV
>gi|145596444|ref|YP_001160741.1| rpoB gene product [Salinispora tropica CNB-440]
MAASRPAKTSRTSSAFAPRRVSFGRITEHLEVPNLLAIQTESFDWLVGNEAWQGRSADDPHARSGLAEIL

DEISPIEDFSGTMSLSFSAPRFDEVKASIEECKEKDLTYCAPLFVTAEFTNNTTGEIKSQTVFMGDFPMM
TPKGTFIINGTERVVVSQLVRSPGVYFDKQPDKTSDRDLSSVKVIPSRGAWLEFDIDKRETVGVRIDRKR
RQAVTVLLKAIGWTAEQIRERFGWSELMMTTLEKDHIASQDEALLDIYRKLRPGEPPTRENAQTLLDNLF
FNPKRYDVAKVGRYKFNKKLELDLSIDTGVLTQDDIVATVEYLCRLHAGEEGYEADDIDHFGNRRLRTVG
ELIQNQVRVGLSRMERVVRERMTTQDVEAITPQTLINIRPVVAAIKEFFGTSQLSQFMDQTNPLAGLTHR
RRLSALGPGGLSRERAGFEVRDVHPSHYGRMCPIETPEGPNIGLIGALSTFARVNPFGFIETPYRKVVDG
RVTDQIDYLTADEEDRFVKAQANAPLRADGSFAEERVLVRRKGGETEDVPPAAVDYMDVSPRQMTSVATA
MIPFLEHDDANRALMGANMQRQAVPLVKAEAPLVGTGMEYRAAVDAGDVVVAEFGGVVEDLCADYITVHQ
DDGHRRTYLLHKFRRSNAGSCVNQKPVVFEGDRVEAGQVIADGPCTDDGEMALGRNLLVAFMTWEGHNYE
DAIILSQRLVQQDVLTSIHIEEHEVDARDTKLGPEEITRDIPNVSEEMLADLDERGIIRIGAEVVPGDVL
VGKVTPKGETELTPEERLLRAIFGEKAREVRDTSLKVPHGETGTVIGVRTFSREDGDELPPGVNELVRVY
VAQKRKIQDGDKLAGRHGNKGVISKILPVEDMPFLEDGTPVDIVLNPLGVPSRMNIGQVLETHLGWVAKT
GWKVEGDDAEWKRQLQAISADESEPDSNVATPVFDGAREEEISGLLASTRANRDGKQLIGSSGKAQLFDG
RSGEPLPDPIAVGYIYILKLNHLVDDKIHARSTGPYSMITQQPLGGKAQFGGQRFGEMECWAMQAYGAAY
ALQELLTIKSDDVLGRVKVYEAIVKGENIPEPGIPESFKVLLKELQSLCLNVEVLSSDGVALEMRETDDE
VFRAAEELGIDLSRREPSSVEEV
>gi|291298701|ref|YP_003509979.1| DNA-directed RNA polymerase subunit
beta [Stackebrandtia nassauensis DSM 44728]
MAASRPAKTSNQSALAPNRISFGRITEHLEVPNLLALQTDSFDWLVGNDAYRARTEAGATCGLDDILEEI
SPIEDFSGTMSLSFSNPRFDEVKAPIEECKEKDLTYCAPLFVTAEFINHNTGEIKSQTVFMGDFPMMTPK
GTFIINGTERVVVSQLVRSPGVYFDKQPDKTSDRDLSSVKVIPGRGAWLEFDIDKRETAGVRIDRKRRQA
VTVLLKAIGWTNDQIRERFGWSEIMMATLEKDHVGSQDEALLDIYRKLRPGEPPTKENAETLLDNLFFNP
KRYDLAKVGRYQVNKKFGLSTPISTGVLTEADLVSIIEYLCRLQNGEDGYDRDDIDHFGNRRIRTVGELI
QNQVRVGLSRMERVVRERMTTQDVEAITPQTLINIRPVVAAIREFFGTSQLSQFMDQVNPIAGLTHRRRL
NALGPGGLSRDRAAMEVRDVHPSHYGRMCPIETPEGPNIGLIGALAAFARVNPFGFIETPYRRIDNGRVT
DDVKYLTADEEDRCVIAQANTKLNDDGTFAEERILVRRKGGEMELVGHDAVDYMDVSPRQMTSVATAMVP
FLEHDDANRALMGANMQRQAVPLIKTQSPFVGTGMEYRAAVDAGDVVVNEVAGVAEDVCADYITVHQDDG
FRRTYLLHKFHRSNAGTCVNQIPVINEGDRVEAGQVMADGPCTDKGEMALGKNLLVAFMPWEGQNYEDAI
ILSSRLVQEDVLTSIHIDEHEVDARDTKLGPEEITREIPNVSEEMLSDLDERGIIRIGAEVRDGDILVGK
VTPKGETELTPEERLLRAIFGEKAREVRDTSLKVPHGESGTVIGVRTFSRDDGDELSPGVNELVRVYIAQ
KRKISDGDKLAGRHGNKGVISKIVPAEDMPFLPDGTPVDVVLNPLGVPGRMNIGQVLELHLGWIAKSGWQ
LAGDAEGWQKSLKAIGAEEAPPDSTVATPVFDGVHLEEITGLLESARPNRDGDRMVGTDGKAKLFDGRTG
EPFPGPISVGYMYILKLNHLVDDKIHARSTGPYSMITQQPLGGKAQFGGQRFGEMECWAMQAYGASYALQ
ELLTIKSDDILGRVKVYEAVVKGENIPEPGIPESFKVLLKELQSLCLNVEVLSSDGTAVELRDSDDEVFR
AAEELGIDLSRHEPSSVDLEGA
>gi|21223036|ref|NP_628815.1| DNA-directed RNA polymerase subunit beta
[Streptomyces coelicolor A3(2)]
MAASRNASTANTNNAASTAPLRISFAKIKEPLEVPNLLALQTESFDWLLGNDAWKARVESALESGQDVPT
KSGLEEIFEEISPIEDFSGSMSLTFRDHRFEPPKNSIDECKDRDFTYAAPLFVTAEFTNNETGEIKSQTV
FMGDFPLMTNKGTFVINGTERVVVSQLVRSPGVYFDSSIDKTSDKDIFSAKIIPSRGAWLEMEIDKRDMV
GVRIDRKRKQSVTVLLKALGWTTEQILEEFGEYESMRATLEKDHTQGQDDALLDIYRKLRPGEPPTREAA
QTLLENLYFNPKRYDLAKVGRYKVNKKLGADEPLDAGVLTTDDVIATIKYLVKLHAGETETVGESGREIV
VETDDIDHFGNRRIRNVGELIQNQVRTGLARMERVVRERMTTQDVEAITPQTLINIRPVVASIKEFFGTS
QLSQFMDQNNPLSGLTHKRRLNALGPGGLSRERAGFEVRDVHPSHYGRMCPIETPEGPNIGLIGSLASYG
RINPFGFIETPYRKVVEGQVTDDVDYLTADEEDRFVIAQANAALGDDMRFAEARVLVRRRGGEVDYVPGD
DVDYMDVSPRQMVSVATAMIPFLEHDDANRALMGANMMRQAVPLIKSESPLVGTGMEYRSAADAGDVVKA
EKAGVVQEVSADYITTTNDDGTYITYRLAKFSRSNQGTSVNQKVIVAEGDRIIEGQVLADGPATENGEMA
LGKNLLVAFMPWEGHNYEDAIILSQRLVQDDVLSSIHIEEHEVDARDTKLGPEEITRDIPNVSEEVLADL
DERGIIRIGAEVVAGDILVGKVTPKGETELTPEERLLRAIFGEKAREVRDTSLKVPHGEIGKVIGVRVFD
REEGDELPPGVNQLVRVYVAQKRKITDGDKLAGRHGNKGVISKINPIEDMPFLEDGTPVDIILNPLAVPS
RMNPGQVLEIHLGWLASRGWDVSGLAEEWAQRLQVIGADKVEPGTNVATPVFDGAREDELAGLLQHTIPN
RDGERMVLPSGKARLFDGRSGEPFPEPISVGYMYILKLHHLVDDKLHARSTGPYSMITQQPLGGKAQFGG
QRFGEMEVWALEAYGAAYALQELLTIKSDDVTGRVKVYEAIVKGENIPEPGIPESFKVLIKEMQSLCLNV
EVLSSDGMSIEMRDTDEDVFRAAEELGIDLSRREPSSVEEV
>gi|256814391|ref|ZP_05539406.1| DNA-directed RNA polymerase subunit
beta [Streptomyces griseoflavus Tu4000]
MAASRNASTANTNNGASTAPLRISFAKIKEPLEVPNLLALQTESFDWLLGNTAWQSRVEEALESGHDVPTK
SGLEEIFEEISPIEDFSGSMSLTFRDHRFEPPKNSIDECKERDFTYAAPLFVTAEFTNNETGEIKSQTVFM

GDFPLMTNKGTFVINGTERVVVSQLVRSPGVYFDSSIDKTSDKDIFSAKIIPSRGAWLEMEIDKRDMVGVR
IDRKRKQSVTVLLKALGWTTEQILEEFGEYESMRATLEKDHTQGQDDALLDIYRKLRPGEPPTREAAQTLL
ENLYFNPKRYDLAKVGRYKVNKKLGGDEPLDAGVLTTDDIIATIKYLVQLHAGETETVGGSGRSIIVETDD
IDHFGNRRIRNVGELIQNQVRTGLARMERVVRERMTTQDVEAITPQTLINIRPVVASIKEFFGTSQLSQFM
DQNNPLSGLTHKRRLNALGPGGLSRERAGFEVRDVHPSHYGRMCPIETPEGPNIGLIGSLASYGRINPFGF
IETPYRKVVDGQVTDEVDYLTADEEDRFVIAQANAPLNDEMRFVENRILVRRRGGEVDYVPGDEVDYMDVS
PRQMVSVATAMIPFLEHDDANRALMGANMMRQAVPLIKSESPLVGTGMEYRSAVDAGDVVKAEKDGVVQEV
SADYITTANDDGTYITYRLAKFSRSNQGTSVNQKVIVNEGDRIITGQVLADGPATQNGEMALGKNLLVAFM
PWEGHNYEDAIILSQRLVQDDVLSSIHIEEHEVDARDTKLGPEEITRDIPNVSEEVLADLDERGIIRIGAE
VIAGDILVGKVTPKGETELTPEERLLRAIFGEKAREVRDTSLKVPHGETGKVIGVRVFDREEGDELPPGVN
QLVRVYVAQKRKITDGDKLAGRHGNKGVISKINPIEDMPFLEDGTPVDIILNPLGVPSRMNPGQVLEIHLG
WLASRGWDVSGLAEEWAERLQVIGADKVEPGTNVATPVFDGAREDELAGLLQHTIPNRDGERMVLPSGKAR
LYDGRSGEPFPEPISVGYMYILKLHHLVDDKLHARSTGPYSMITQQPLGGKAQFGGQRFGEMEVWALEAYG
AAYALQELLTIKSDDVTGRVKVYEAIVKGENIPEPGIPESFKVLIKEMQSLCLNVEVLSSDGMSIEMRDTD
EDVFRAAEELGIDLSRREPSSVEEV
>lcl|SSMG_00351T0 | SSMG_00351 | Streptomyces sp_AA4 DNA-directed RNA
polymerase, beta subunit (1168 aa)
MAVSPANQATAATTSAESRAQATGIPGAPKRVSFAKIREPLSTPNLLDVQIQSFQWFTGNEAWFERRVNEG
EENPVGGLEEVLNEISPIEDFSGSMSLSFSAPRFDEVKASIEECKDKDMTYAAPLFVTAEFVNNNTGEIKS
QTVFLGDFPVMTDKGTFIINGTERVVVSQLVRSPGVYFDSSVDKTTDKDVFSVRVIPSRGAWLEFDVDKRD
TVGVRIDRKRRQPVTVLLKALGWTTEAIRERFSFSETLLATLEKDHTAGTDEALLDIYRKLRPGEPPTKES
AQTLLENLFFKPKRYDLAKVGRYKVNKKLGLDTPYETGTLTEEDIVSAIEYLVRLHAGEDKMTSSAGVEVP
VETDDIDHFGNRRLRTVGELIQNQIRVGLSRTERVVRERMTTQDVEAITPQTLINIRPIGAAIKEFFGTSQ
LSQFMDQNNPLSGLTHKRRLSALGPGGLSRERAGMEVRDVHPSHYGRMCPIETPEGPNIGLIGSLCSYARV
NPFGFIETPYRKVVEGQVTDQVDYLTADEEDRYVKAQANAPLTDDGHFVEEKVLGRRKGGEVELIDPLEID
YMDVSPRQMVSVATAMIPFLEHDDANRALMGANMQRQAVPLLRNQAPYVGTGVELRAAVDAGDVLVAEQAG
VVEELSADIITIMHDDGTRKSYGLYKFRRSNHGTCFNHRPIVNEGDRVEQGQVIADGPSTENGEMALGKNL
LVAVMPWEGHNYEDAIILSERLVQDDVLTSIHIEEHEIDARDTKLGAEEITRDIPNVSEEVLADLDERGII
RIGAEVRDGDILVGKVTPKGETELTPEERLLRAIFGEKAREVRDTSLKVPHGETGKVIGIRVFSREDDDEL
PPGVNELVRVYVAQKRKIQPGDKLAGRHGNKGVIGKILPVEDMPFMEDGTPVDIILNTHGVPRRMNIGQIL
ELHLGWLASQGWTIEGNPDWAKNLSPELYDVAPGTNTATPVFDGAKEEELTGLLGATKPNRDGERMVKENG
KATLFDGRSGEPYPYPVSVGYMYILKLHHLVDDKIHARSTGPYSMITQQPLGGKAQFGGQRFGEMECWAMQ
AYGAAYTLQELLTIKSDDVVGRVKVYEAIVKGENIPEPGIPESFKVLLKELQSLCLNVEVLSSDGAAIEMR
DSDDEDLERAAANLGINLSRNESPSVDDVVH
>gi|345850558|ref|ZP_08803553.1| DNA-directed RNA polymerase subunit
beta [Streptomyces zinciresistens K42]
MAASRTASNANTNNGASTAPLRISFAKIKEPLEVPNLLALQTESFDWLLGNTAWQSRVEAALESGQDVPT
KSGLEEIFEEISPIEDFSGSMSLTFRDHRFEPPKNSIDECKDRDFTYAAPLFVTAEFTNNETGEIKSQTV
FMGDFPLMTHKGTFVINGTERVVVSQLVRSPGVYFDSSIDKTSDKDIFSAKVIPSRGAWLELEIDKRDMV
GVRIDRKRKQSVTVLLKALGWTTEQILEEFGEYESMRATLEKDHTQGQDDALLDIYRKLRPGEPPTREAA
QTLLENLYFNPKRYDLAKVGRYKVNKKLGAEAPLDAGVLTVEDVISTIKYLVKLHAGETETVGDSGNTIV
VETDDIDHFGNRRLRSVGELIQNQVRTGLARMERVVRERMTTQDVEAITPQTLINIRPVVASIKEFFGTS
QLSQFMDQNNPLSGLTHKRRLSALGPGGLSRERAGFEVRDVHPSHYGRMCPIETPEGPNIGLIGSLASYG
RVNAFGFVETPYRKVVDGRVTDEVDYLTADEEDRFVIAQANATLNDDMRFAENRVLVRRRGGEVDYVAGD
DVDYMDVSPRQMVSVATAMIPFLEHDDANRALMGANMMRQAVPLIKSESPLVGTGMEYRSAVDAGDVVKA
EKAGVVQEVSADYITTANDDGTYITYRLAKFARSNQGTSVNQKVIVNEGDRIIEGQVLADGPATENGEMA
LGKNLLVAFMPWEGHNYEDAIILSQRLVQDDVLSSIHIEEHEVDARDTKLGPEEITRDIPNVSEEVLADL
DERGIIRIGAEVVAGDILVGKVTPKGETELTPEERLLRAIFGEKAREVRDTSLKVPHGEIGKVIGVRVFD
REEGDELPPGVNQLVRVYVAQKRKITDGDKLAGRHGNKGVISKILPIEDMPFLEDGTPVDIILNPLGVPS
RMNPGQVLEIHLGWLASRGWDVSGLADEWAQRLQVIGADQVDPGTNVATPVFDGAREDELAGLLQHTIPN
RDGERMVLPSGKARLFDGRSGEPFPDPISVGYMYILKLHHLVDDKLHARSTGPYSMITQQPLGGKAQFGG
QRFGEMEVWALEAYGAAYALQELLTIKSDDVTGRVKVYEAIVKGENIPEPGIPESFKVLIKEMQSLCLNV
EVLSSDGMSIEMRDTDEDVFRAAEELGIDLSRREPSSVEEV
>gi|271962625|ref|YP_003336821.1| unnamed protein product
[Streptosporangium roseum DSM 43021]
MAASRNASAVPAGPRRVSFARIQEPLEVPDLLALQTESFDWLLGNEKWKGRVEAARQAGRKDVPAQSGLE
EIFEEISPIEDFSGTMSLSFRDHRFEPPKYSVDECKDKDMTYSAPMFVTAEFINNTTGEIKSQTVFMGDF
PLMTGKGTFIINGTERVVVSQLVRSPGVYFDRSVDKTSDKDLYGCKVIPSRGAWLEFEIDKRDSVGVRID

RKRKQAVTVLLKALGWTNDQILERFGQYESMRATLEKDHTAGQDDALLDIYRKLRPGEPPTKESAQTLLE
NLYFNHKRYDLAKVGRYKINKKLGVDSEITQGTLTEEDIVATIEYIVKLHAGETSMAGANGEIVVETDDI
DHFGNRRLRTVGELIQNQVRLGLARMERVVRERMTTQDVEAITPQTLINIRPVVASIKEFFGTSQLSQFM
DQTNPLAGLTHKRRLSALGPGGLSRERAGFEVRDVHPSHYGRMCPIETPEGPNIGLIGSLASFGRVNSFG
FVETPYRKVLDGRVTDTVEYLTADEEDRYVIAQANTPIGSDGTFLEDRVLVRRKGGEFESLRANEVDYMD
VSARQMVSVATAMIPFLEHDDANRALMGSNMQRQSVPLLKSEAPLVGTGMEYRAATDAGDVITADKAGVV
EEVSADYVTVMNDDGTRTTYRVAKFKRSNQGTCFNQKPIVKEGDRIEVNQVVADGPCTDDGEMALGKNLL
VAFMPWEGHNYEDAIILSQRLVQDDVLSSIHIEEHEVDARDTKLGPEEITRDIPNVSEEVLADLDERGII
RIGAEVVPGDILVGKVTPKGETELTPEERLLRAIFGEKAREVRDTSLKVPHGEQGKVIGVRVFSREEGDE
LPPGVNELVRVYVAQKRKITDGDKLAGRHGNKGVISKILPVEDMPFLEDGTPVDIILNPLGVPGRMNVGQ
VLETHLGWIAARGWDISGIEEAWAERLRDKGFAEVDPRTNMATPVFDGANEEEIVGLLDNTLVNRDGGRM
VGANGKAQLFDGRSGEPFPHPISVGYIYILKLLHLVDDKIHARSTGPYSMITQQPLGGKAQFGGQRFGEM
EVWALEAYGAAYALQELLTIKSDDVLGRVKVYEAIVKGENIPEPGIPESFKVLIKEMQSLCLNVEVLSSD
GMSIEMRDTDEDVFRAAEELGIDLSRREPSSVEEV
>gi|72163053|ref|YP_290710.1| rpoB gene product [Thermobifida fusca YX]
MAASRIASANALGPNRVSFARIKEPLEVPNLLALQTESFDWLLGNERWRARVEAARKAGRKDIPEQSGLE
EIFEEISPIEDFSGTMSLSFRDHRFEPPKYSEEECKDKDMTYSAPMFVTAEFINNDTGEIKSQTVFMGDF
PLMTAKGTFIINGTERVVVSQLVRSPGVYFDSQMDKSSDKELYGCKIIPSRGAWLEFEIDKRDFVGVRID
RKRKQAVTILLKALGWTTDQILERFGEYESIRATLEKDPTAGTDDALLDIYRKLRPGEPPTKEAAQALLE
NLYFNPKRYDLAKVGRYKINKKLGLEIDITQGTLTEEDIVATVDYLVRLHAGEKELVRPHGTFPIEVDDI
DHFGNRRLRTVGELIQNQVRLGLARMERVVRERMTTQDVEAITPQTLINIRPVVASIREFFGTSQLSQFM
DQTNPLAGLTHKRRLSALGPGGLSRERAGFEVRDVHPSHYGRMCPIETPEGPNIGLIGSLAAYARVNSFG
FIETPYRKVVDGRITDEVVYLTADEEDRYVIAQANTPVNPDGTFAESQVLARRKGGEFESVAAEEVHYMD
ISPRQMVSVATAMIPFLEHDDANRALMGSNMQRQAVPLLRAEAPLVGTGMEYRAATDAGDVILAEKSGVV
EDVTADYITVLADDGTRKTYRVHKFRRTNQGTCFNQRPIVEEGQRVEEGQVLADGPSTEAGEMALGKNLL
VAYMSWEGHNYEDAIVLSQRLVEEDILSSIHIEEHEVDARETKLGPEEITREIPNVSEEVLADLDERGII
RIGAEVVDGDILVGKVTPKGETELTPEERLLRAIFGEKAREVRDTSLKVPHGESGKVIGVRVFSREEGDE
LPPGVNELVRVYVAQKRKITDGDKLAGRHGNKGVIAKILPKEDMPFLEDGTPVDIVLNPLGVPGRMNIGQ
IMEMHLGWLAKHGWKVEGDDAEWKRRLRDIGAHEAPPNSKVATPVFDGAREDEISGLLSCVLPDQDGDIL
VNKFGKAKLYDGRTGEPFKEPVAVGYAYFLKLHHLVDDKIHARSTGPYSMITQQPLGGKAQFGGQRFGEM
EVWALEAYGAAYALQELLTIKSDDINGRVKVYEAIVKGENIPEPGIPESFKVLIKEMQSLCLNVEVLSRD
GMSIEMRDSEEDVFRAAEELGIDLGRREPSSVEEV
>gi|296268531|ref|YP_003651163.1| DNA-directed RNA polymerase subunit
beta [Thermobispora bispora DSM 43833]
MAASRNASAAFASPRLSFARIQEPLEVPDLLAVQIESFDWLIGNERWKARVEAARKAGRKDIPMQSGLEE
VFEEISPIEDFSGTMSLSFRDHRFEPPKHTIEECRDKDMTYSASLFVTAEFINNTTGEIKSQTVFMGDFP
LMTPKGTFIINGTERVVVSQLVRSPGVYFDRTVDKTSDKDVYSCKIIPSRGAWLEFEIDKRDSVGVRIDR
KRKQPVTVLLKALGWTNDRILERFGQYESMRATLEKDHTTGQEDALIDIYRKLRPGEPPTKESAQALLEN
LYFNPKRYDLAKVGRYKLNKKLGVDADITQGTLTEDDIVATIEYLVRLHAGEETMIGANGREVIVETDDI
DHFGNRRLRTVGELIQNQVRLGLARMERVVRERMTTQDVEAITPQTLINVRPVTASIREFFGTSQLSQFM
DQTNPLAGLTHKRRLNALGPGGLSRERAGFEVRDVHPSHYGRMCPIETPEGPNIGLIGSLATYARVNAFG
FVETPYRKVVDGRVTDQIEYLTADEEDRYVVAQANTVVNPDGTFAEKRVLARRKGGEFEAVDPSEVDYMD
VSPRQMVSVATAMIPFLEHDDANRALMGSNMQRQAVPLLRSEAPLVGTGMEYRAATDAGDVIIAEKSGVV
EEVSADYITVLNDDGTRTTYRVVKFKRSNQGTCYNQKPIVEEGDRVEQGQVIADGPCTENGEMALGKNLL
VAFMPWEGHNYEDAIILSQRLVQDDVLTSIHIEEHEVDARDTKLGPEEITRDIPNVSEEVLADLDERGII
RIGAEVTTGDILVGKVTPKGETELTPEERLLRAIFGEKAREVRDTSLKVPHGESGKVIGVRVFRREDGDE
LPPGVNELVRVYVAQKRKITDGDKLAGRHGNKGVIAKILPVEDMPFLEDGTPVDIILNPLGVPGRMNLGQ
VLETHLGWAAKQGWDISGVEEEWAKRLREKGMEKVGPDTKVATPVFDGAREEEIVGLLQHTLPNRDGQRL
VGPNGKAILYDGRSGEPFPYPVSVGYIYILKLAHLVDDKIHARSTGPYSMITQQPLGGKAQFGGQRFGEM
EVWALEAYGAAYALQELLTIKSDDVTGRVKVYEAIVKGENIPEAGIPESFKVLIKEMQSLCLNVEVLSSD
GMSIEMREAEEDVFRAAEELGIDLSRREPSSVEEV
>gi|269128533|ref|YP_003301903.1| DNA-directed RNA polymerase subunit
beta [Thermomonospora curvata DSM 43183]
MAASRNASNTVTGPNRVSFARIKEPLEVPDLLALQTDSFDWLLGNERWKARVEAALKAGVRDIPTQSGLE
EIFEEISPIEDFSGTMSLSFRDHRFEPPKYSIEECRDKDMTYSAPMFVTAEFINNSTGEIKSQTVFMGDF
PLMTPKGTFIINGTERVVVSQLVRSPGVYFDRSLDKASDKDIYGCRIIPSRGAWLEFEIDKRDNVGVRID
RKRKQPVTVLLKALGWTEARIREHFGAYESMNITLEKDHTSGQDDALLDIYRKLRPGEPPTKESAQALLE
NLYFNPKRYDLAKVGRYKVNKKLGLRLDMNQGTLTEDDIVATIEYLVRLHAGEEEAVLTAAGPEGVSVPI

EIDDIDHFGNRRLRTVGELIQNQVRLGLARMERVVRERMTTQDVEAITPQTLINIRPVVASIKEFFGTSQ
LSQFMDQTNPLAGLTHKRRLSALGPGGLSRERAGFEVRDVHPSHYGRMCPIETPEGPNIGLIGSLSAYGR
VNPFGFIETPYRKVVDGRVTDEIHYLTADEEDRYIKAQANSPLNPDGTFAEEKVLVRTKGGEIEYLPPEE
VHYMDVSPRQMTSVATAMIPFLEHDDANRALMGSNMQRQAVPLLRSEAPLVGTGMEYRAATDAGDVILAE
KPGVVEEVSADYITVLNDDGTRTTYRVAKFKRSNQGTCFNQKPIVDEGQRVEAGQVIADGPCTDQGEMAL
GKNLLVAFMPWEGHNYEDAIILSQRLVQDDVLSSIHIEEHEVDARDTKLGPEEITRDIPNVSEEVLADLD
ERGIIRIGADVVPGDILVGKVTPKGETELTPEERLLRAIFGEKAREVRDTSLKVPHGEQGKVIGVRVFSR
EEGDELPPGVNELVRVYVAQKRKITDGDKLAGRHGNKGVIAKILPVEDMPFLEDGTPVDIILNPLGVPGR
MNVGQVLETHLGWVASQGWKIEGDDADWKRSLQAIGATEAGPWTKTATPVFDGAREEEITGLLSCTLPNR
DGMRLIGPNGKAKLYDGRTGEPFKDPIAVGYIYILKLLHLVDDKIHARSTGPYSMITQQPLGGKAQFGGQ
RFGEMEVWALEAYGAAYALQELLTIKSDDVLGRVKVYEAIVKGENIPEPGIPESFKVLIKEMQSLCLNVE
VLSSDGMSIEMRDSDEDVFRAAEELGIDLSRREPSSVEEV
>gi|330470182|ref|YP_004407925.1| rpoB gene product [Verrucosispora
maris AB-18-032]
MAASRPAKTSRTSSAFAPRRVSFGRITEHLEVPNLLAIQNESFDWLVGNEAWQGRSADDPHARSGLAEIL
DEISPIEDFSGTMSLSFSAPRFDEVKASIEECKEKDLTYCAPLFVTAEFTNNTTGEIKSQTVFMGDFPMM
TPKGTFIINGTERVVVSQLVRSPGVYFDKQPDKTSDRDLSSVKVIPSRGAWLEFDIDKRDTVGVRIDRKR
RQAVTVLLKAIGWSAERIREKFGWSELMMTTLEKDHIAGQDEALLDIYRKLRPGEPPTRENAQTLLDNLF
FNPKRYDVAKVGRYKFNKKLELDVPITTGTLTEDDIVATVEYLCRLHAGEEGYEADDIDHFGNRRLRTVG
ELIQNQVRVGLSRMERVVRERMTTQDVEAITPQTLINIRPVVAAIREFFGTSQLSQFMDQTNPLAGLTHR
RRLSALGPGGLSRERAGFEVRDVHPSHYGRMCPIETPEGPNIGLIGALSTFARVNPFGFIETPYRKVVDG
RVTDQIDYLTADEEDRFVKAQANAPLRADGTFAEDRVLVRRKGGETEDVPPAAVDYMDVSPRQMTSVATA
MIPFLEHDDANRALMGANMQRQAVPLVKAESPLVGTGMEYRAAVDAGDVVVAEVGGVVEDLCADYITVHQ
DDGHRRTYLLHKFRRSNAGSCVNQKPVVFEGDRVEAGQVIADGPCTDEGEMALGRNLLVAFMCWEGHNYE
DAIILSQRLVQQDVLTSIHIEEHEVDARDTKLGPEEITRDIPNVSEEMLADLDERGIIRIGAEVVPGDIL
VGKVTPKGETELTPEERLLRAIFGEKAREVRDTSLKVPHGETGTVIGVRTFSREDGDELPPGVNELVRVY
VAQKRKIQDGDKLAGRHGNKGVISKILPVEDMPFLEDGTPVDIVLNPLGVPSRMNIGQVLETHLGWVAKT
GWKVEGDDAEWKRQLQSIGADSSEPDTNVATPVFDGAREEEISGLLASTLPNRDGKQLIGATGKAQLFDG
RSGEPLPDPIAVGYIYILKLNHLVDDKIHARSTGPYSMITQQPLGGKAQFGGQRFGEMECWAMQAYGAAY
ALQELLTIKSDDVLGRVKVYEAIVKGENIPEPGIPESFKVLLKELQSLCLNVEVLSSDGVALEMRETDDE
VFRAAEELGIDLSRREPSSVEEV

Supplemental Data file 3. SsgB protein sequences from actinomycetes used as input for Figure 3.

>gi|117928576|ref|YP_873127.1| sporulation and cell division protein SsgA [Acidothermus cellulolyticus 11B] (Acel_1369)
MNASVHIIEVTEFHLLLDGDSRVITARLHYDPADPYVITVHFDTGVTWVLGRDLLADGLH
MEIGDGDVHLRPEGDQLFLTLRSPSGEALLAASRSAIAGFMADTERVVPRGTEHLRVDLD
KELARLLP
>gi|383781699|ref|YP_005466266.1| unnamed protein product [Actinoplanes missouriensis 431] SsgB 40% AMIS_65300
MSTIRPTTVEVETSLRLVAPDATALPVRASLRYDPADPYAVHVLFHAESAGGEAVSWSFARELLVTGLDE
PAGIGDVRVWPWATPRGDFVALALSSPDGNALFEVPRSVLVRFLRRTYVVVPRGRESDHLDVDAAVNRLL
AGR
>gi|359839220|gb|AEV87661.1| sporulation and cell division protein SsgA [Actinoplanes sp. SE50/110] ACPL_6779 SCO1541 50%
MSTIRPTTVEVETSLRLVAPDATALPVRASLRYDPSDPYAVHVLFHAESAGGEAVSWSFARELLVTGLDE
PAGIGDVRVWPWATPRGDFVALALSSPDGNALFEVPRSVLVRFLRRTYVVVPRGRESEHLDVDAAVNRLL
AGR
>gi|256376149|ref|YP_003099809.1| sporulation and cell division protein SsgA [Actinosynnema mirum DSM 43827] (Amir_2015)
mrndhvtlrstavfdllaprtpavpvqvelrydtkdpyaviaafrtgragwvewvfardl
ladgliadagdgdvrirpaaddpevvvielsspsghamfeasaqeladfldrtydvvvpg
nehlwvdvdealthlisndls
>gi|399539086|ref|YP_006551748.1| sporulation and cell division protein SsgA [Amycolatopsis mediterranei S699]
MRNDHVTLRSTAVFDLLAPRTPAVPVKVELRYDTRDPYAVVAAFRTGRAGWVEWVYARDLLADGLLADAG
DGDVRIRPSVEDPEAVLIELNSPSGHAMFEASAQELADFLDRTYDVVLPGNEHLWVDVDDALTHLIPHDL
A
>gi|300787203|ref|YP_003767494.1| sporulation and cell division protein SsgA [Amycolatopsis mediterranei U32] (Amed_5330)
mrndhvtlrstavfdllaprtpavpvkvelrydtrdpyavvaafrtgragwvewvyardl
ladglladagdgdvrirpsvedpeavlielnspsghamfeasaqeladfldrtydvvlpg
nehlwvdvddalthliphdla
>gi|379735734|ref|YP_005329240.1| unnamed protein product [Blastococcus saxobsidens DD2] BLASA_2304 SsgB 40%
MPGHISPITLQLIGPQSWTEVPALLCYDPADPFAVRIAFGDPGEQDPADGDEGIAWLVSRELLQAGLEHP
AGDGDVRVWPAHATADVLFLHLRAPSGEALFELSRGTVAAFLRHTEALVPTGSETGLLHLDQELDALLSN
GGPDAPGR
>gi|256391413|ref|YP_003112977.1| sporulation and cell division protein SsgA [Catenulispora acidiphila DSM 44928] (Caci_2217)
msssvtcelqlrlvisgdsslpvpaglrydtadpyavhatfhtggqdtvdwvfarellae
glrrptgtgdvrvwpsrsrgqaivclalsspegealleaparalesflkrtntavppgle
hrfydldaelvhlfadn
>gi|332670552|ref|YP_004453560.1| sporulation and cell division protein SsgA [Cellulomonas fimi ATCC 484] Celf_2043
MTQSSYDVVEVVAMQLIGSDASVIPVSAELSFRTSDPYTVRAVFTGPHTMSTWLLGRELLSLGMLASADA
PAGTGDVQVWRDEDPDYTLVSLNGVEGSALLAAPTEPITRFLASTEALVPLGAESARMESEITALIAALL
TA
>gi|296129627|ref|YP_003636877.1| unnamed protein product [Cellulomonas flavigena DSM 20109] (Cfla_1781)
madtsydvievvamqligsdatvipvsaemcfrtsdpytvravftgahtmstwllgrell
aqgvhavadapagtgdvqvwrdddpdytlvslsgvegsallaaptepllrflaateslvp
igaesdkmegeitaliaallta
>gi|336320679|ref|YP_004600647.1| sporulation and cell division protein SsgA [Cellulomonas gilvus ATCC 13127] Celgi_1568
MTHSSYDVVEVVAMQLIGSDASVIPVSAELSFRTTDPYTVRAVFTGPHTMSTWLIGRELLSMGMHAAADA
PAGSGDVQVWRDEDPDYALVSLSGVEGSALLAAPSEPILRFLATTESLVPLGSESDRMESEISAFIAALL

TA
>gi|111221561|ref|YP_712355.1| unnamed protein product [Frankia alni ACN14a] (FRAAL2127)
MPVAATVRYEPADPYAISIGFRTGADEVVEWTFARQLLSDGVRRPAGDGDVQVWPAAQSG
GRIVCLSLSSPSGHALFEMPRSEVLAFLRRTYSAVPLGGESDIIDLDAELALLIWGGPER
>gi|158316914|ref|YP_001509422.1| sporulation and cell division protein SsgA [Frankia sp. EAN1pec] Franean1_5158
MTIRHDSITAELALRLVVPGGAPVPVAATVRYEPADPYAVSIGFRTGADEVVEWTFARQLLSDGVRRPAG
DGDVQVWPAAQSGGRIVCLSLSSPSGHALFEMPRAEVLAFLRRTYSAVPLGGESDVIDLDAELALLIWGG
PER
>gi|86740065|ref|YP_480465.1| sporulation and cell division protein SsgA [Frankia sp. CcI3] Francci3_1359
MTIRHDSITAELALRLVVPGGAPVPVAATVRYEPADPYAISIGFRTGADEVVEWTFARQLLSDGVRRPAG
DGDVQVWPAAQSGGRIVCLSLSSPSGHALFEMPRSEVLAFLRRTYSAVPLGGESDIIDLDAELALLIWGG
PER
>gi|358463323|ref|ZP_09173392.1| sporulation and cell division protein SsgA [Frankia sp. CN3] FrCN3DRAFT_8065
MTIRHDSITAELALRLVVPGGAPVPVAATVRYEPDDPYAVSIGFRTGADEVVEWTFARQLLSDGVRRPAG
DGDVQVWPAAQSGGRIVCLSLSSPSGHALFEMPRSEVLAFLRRTYAAVPLGSESDVIDLDAELALLIWGG
PDR
>gi|288922741|ref|ZP_06416912.1| sporulation and cell division protein SsgA [Frankia sp. EUN1f] FrEUN1fDRAFT_6610
MTIRHDSITAELALRLVVPGGAPVPVAATVRYEPADPYAVSIGFRTGADEVVEWTFARQLLSDGVRRPAG
DGDVQVWPAAQSGGRIVCLSLSSPSGHALFEMPRAEVLAFLRRTYAAVPLGGESDVIDLDAELALLIWGG
PER
>gi|392943918|ref|ZP_10309560.1| sporulation and cell division protein, SsgA [Frankia sp. QA3]
MTIRHDSITAELALRLVVPGGAPVPVAATVRYEPADPYAISIGFRTGADEVVEWTFARQLLSDGVRRPAG
DGDVQVWPAAQSGGRIVCLSLSSPSGHALFEMPRSEVLAFLRRTYSAVPLGGESDIIDLDAELALLIWGG
PER
>gi|336178472|ref|YP_004583847.1| sporulation and cell division protein SsgA [Frankia symbiont of Datisca glomerata] FsymDg_2563
MTIRHDSITAELALRLVVPGGAPVPVAATMRYEPADPYAVSIGFRTGADEVVEWTFARQLLSDGVRRPAG
DGDVQVWPAAQSGGRIVCLSLSSPSGHALFEMPRSEVLSFLRRTYSVVPLGGESEVVDLDAELALLIWGG
PQT
>gi|284991618|ref|YP_003410172.1| sporulation and cell division protein SsgA [Geodermatophilus obscurus DSM 43160] (Gobs_3195)
minratpgytcpttlhlvgpqswtevpallsyeirdpfavritfgdhaeedgiswlvgrd
llhagldqpagegdvrlwpsrtsgdvlflhlrapsgealfevsrgtvagflqltasvvpf
geessvlqvdgelaallsnggadptgr
>gi|152967013|ref|YP_001362797.1| sporulation and cell division protein SsgA [Kineococcus radiotolerans SRS30216]
mpalphrktevdvdvslrlrttsgpglpvpaslhygaddpyaihavfrggdtdvewvfar
dllreglsapagagdvqvqpcsdspdgrprvllrlsspdgnaeleadesdvrrflrrada
lvppgretrhldldeliarlvs
>gi|357388405|ref|YP_004903244.1| ssgB gene product [Kitasatospora setae KM-6054]
mnttvscelhlrlivssesslpvpaglrydtadpyavh
atfhtgadetvewvfardllaeglhrptgtgdvrvwpsrshgqgvvcialsspegealle
aparalesflkrtdaavppgtehrhfdldrelshilaen
>SsgB_Kutzneria_sp744_KUTG_02305 not on GenBank
MRNDHVTLRSTAVFDLLAPRTPAVPVQVELRYDTRDPYAVVAAFRTGRAGWVEWVYARDL
LADGLIAEAGDGDVRIKPATDDPEVVVIELNSPSGHATFEAASEELADFLDRTYDVVVPG
NEHLWVNVDQALTHLISNDMS
>gi|302866229|ref|YP_003834866.1| sporulation and cell division protein SsgA [Micromonospora aurantiaca ATCC 27029]
MSVIRPTTVEVETSLRLVAPDATALPVRASLRYDPADPYAVHVLFHAESAGGEAVSWSFARELLVTGLDE
PAGIGDVRVWPWATPRGDFVALALSSPDGNALFEVPRSVLVRFLRRTYVVVPRGREAEHLDVDTAVNRLL

AGR
>gi|443289776|ref|ZP_21028870.1| Sporulation and cell division protein SsgA [Micromonospora lupini str. Lupac 08]
MSVIRPTTVEVETSLRLVAPDATALPVRASLRYDPADPYAVHVLFHAESAGGEAVSWSFARELLVTGLDE
PAGIGDVRVWPWATPRGDFVALALSSPDGNALFEVPRSVLVRFLRRTYVVVPRGREAEHLDVDTAVNRLL
AGR
>gi|238063620|ref|ZP_04608329.1| sporulation and cell division protein ssgA [Micromonospora sp. ATCC 39149] (Mcag_04586)
MSVIRPTTVEVETSLRLVAPDATALPVRASLRYDPADPYAVHVLFHAESAGGEAVSWSFARELLVTGLDE
PAGIGDVRVWPWATPRGDFVALALSSPDGNALFEVPRSVLVRFLRRTYVVVPRGREADHLDVDTAVTRLL
AGR
>gi|315502787|ref|YP_004081674.1| sporulation and cell division protein ssga [Micromonospora sp. L5]
MSVIRPTTVEVETSLRLVAPDATALPVRASLRYDPADPYAVHVLFHAESAGGEAVSWSFARELLVTGLDE
PAGIGDVRVWPWATPRGDFVALALSSPDGNALFEVPRSVLVRFLRRTYVVVPRGREAEHLDVDTAVNRLL
AGR
>gi|389865025|ref|YP_006367266.1| sporulation and cell division protein, SsgA [Modestobacter marinus]
MARSSRSGCTARTTFHLVGPQSWTAVPAALVYDSSDPFAVRVRFGDGATDDVEPALYDDPDDDGGVEWLL
SRDLLRAGLTGPVGEGDVRLWPARGGLDVLFLQLRAPSGEAMFELSGAVVGDFLRETELLVPAGGESDLL
RVDEELSALLRGGTDNPTGR
>gi|258653633|ref|YP_003202789.1| sporulation and cell division protein SsgA [Nakamurella multipartita DSM 44233] (Namu_3475)
mydfntveyrmtatvtemvlisdptmppvhaelryrsddpfavqlllsidqspaitwvfg
rdllvegvqapagigdvqvfpvldgtvielrsgeqvasllahtqdlaafldhsieivpsg
sesayfeieadlrdlpihdafea
>gi|326331443|ref|ZP_08197733.1| sporulation and cell division protein SsgA [Nocardioidaceae bacterium Broad-1]
MSDIKHPAVQQDIKISCLDPWGRTVEVPTTLGYRENDPYAVSLTFHSGAGDVEWVVSRTLMLQGLSAPAG
EGDVKIYPSIDEDAKAVAVFDFSSPDGRLIATADSGEVQTFLGKSFSAVPVGSESQHMDLDALIADLLSS
SFDAE
>gi|119716595|ref|YP_923560.1| unnamed protein product [Nocardioides sp. JS614] (Noca_2368)
mdlpatfgydpadpwavwitfrgprdevrwavgrdlllqgltdpagegdiqlwpsidehg
raavvmelcspdgrlvtqlhthelyrfltrtlaivpvgseaidlddlvaeltgisrae
>gi|403507738|ref|YP_006639376.1| putative regulator [Nocardiopsis alba ATCC BAA-2165]
MNSSDITATAELGLRLVVPERTAVPLVARLDYSADDPYAIRVAFHTGEDSPVEWIFARELLTVGIVRPVG
EGDVRVWPSKSEGGERSVSISLSSPFGQAEFDTQVSPLAEFLHRTYEIVPAGQEGSFVDLDEEIQQHLHS
>gi|297562209|ref|YP_003681183.1| sporulation and cell division protein SsgA [Nocardiopsis dassonvillei subsp. dassonvillei DSM 43111] (Ndas_3271)
mnssdttataelglrlvvpertavplvarldysaddpyairvafhtgednpvewifarel
ltvgivrpvgegdvrvwpskddngdrsvsialsspfgqaefdtqvsplaeflhrtyeivp
agqegqfvnldeeiqqhlhs
>gi|331695878|ref|YP_004332117.1| sporulation and cell division protein SsgA [Pseudonocardia dioxanivorans CB1190]
MRDVTNIICKQTVFELIAPDSTVTVNVELTYSFRDPFAVEASFRTGNSQPVVWVFARDLLADGLTEATGD
GDVTVAPIGHGQVELVLTSPSGHARFHADAAELSSFLEHTYAAVPEGAEYSWLDFDAALTELLDEKARD
>gi|418459703|ref|ZP_13030816.1| sporulation and cell division protein, SsgA [Saccharomonospora azurea SZMC 14600]
MRNEHVTLRSTAVFDLLAPRTPPVPVKVELRYDTRDPYAVVAAFRTGRAGWVEWVFARDLLADGLLGEAG
DGDVRLKPSIEDPDSVFVELNSPSGHAMFEASAQELADFLDQTYDVVLPGNEHLWVDVDDALTRLIPHDL
G
>gi|257055521|ref|YP_003133353.1| sporulation and cell division protein, SsgA [Saccharomonospora viridis DSM 43017] (Svir_14890)
MRNDHVTLRSTAVFDLLAPRTPPVPVKVELRYDTRDPYAVVAAFRTGRAGWVEWVFARDL
LSDGLLGEAGDGDVRIRPSMEDPDSVFVELNSPSGHAMFEASAQELADFLDQTYDVVLPG

NEHLWVDVDDALTRLIPHDLG
>gi|134098535|ref|YP_001104196.1| regulator [Saccharopolyspora erythraea NRRL 2338] (SACE_1961)
MRNDHVTLRSTAVFDLLAPQTPAVPVQVELRYDTRDPYAVVAAFRTGRAGWVEWVFARDL
LADGLIAHAGVGDVTIRPAVDDPEVVVIELSSPSGHAVFEASAQELADFLDRTYDVVVPG
NENLWVNVDDALTRLLPHDLS
>gi|348170571|ref|ZP_08877465.1| regulator [Saccharopolyspora spinosa NRRL 18395]
MTLRSTAVFDLLAPQTPAVPVQVELRYDTRDPYAVVAAFRTGRAGWVEWVFARDLLADGLIAHAGEGDVT
IRPAVDAPEVVAIELSSPSGHAVFEASAQELADFLDRTYDVVVPGNENLWVDVDEALTRLLPHDLG
>gi|433604679|ref|YP_007037048.1| Sporulation and cell division protein [Saccharothrix espanaensis DSM 44229]
MRNDHVTLRSTAVFDLLAPRTPAVPVQVELRYDTKDPYAVVAAFRTGRAGWVEWVYARDLLADGLIADAG
DGDVRIRPAADDPEVVVIELSSPSGHAMFEASAQELADFLDRTYDVVVPGNEHLWVDVDEALTHLISNDL
T
>gi|159037194|ref|YP_001536447.1| sporulation and cell division protein SsgA [Salinispora arenicola CNS-205] Sare_1560
MSVIRPTTVEVETSLRLVAPDATALPVRASLRYDPADPYAVHVLFHAESAGGEAVSWSFARELLVTGLDE
PAGIGDVRVWPWATPRGDFVALALSSPDGNALFEVPRSVLVRFLRRTYVVVARGREAEHLDVDTAVSRLL
AGR
>gi|145594147|ref|YP_001158444.1| unnamed protein product [Salinispora tropica CNB-440] (Strop1600)
MSVIRPTTVEVETSLRLVAPDATALPVRASLRYDPADPYAVHVLFHAESAGGEAVSWSFA
RELLVTGLDEPAGIGDVRVWPWATPRGDFVALALSSPDGNALFEVPRSVLVRFLRRTYVV
VARGREAEHLDVDTAVNRLLAGR
>gi|291300584|ref|YP_003511862.1| sporulation and cell division protein SsgA [Stackebrandtia nassauensis DSM 44728] (Snas_3098)
mattrpatvevdttmrfvatdfttvnvrasmrydaddpyaihvmfhpgnpdaeqvswsfa
rellaaglveptgmgdvrvwpwstprgdaialalsspdgnalfevsrslvsrflrrsyal
vpkgkegdsldmdatvaklladr
>gi|21220041|ref|NP_625820.1| regulator [Streptomyces coelicolor A3(2)] (SCO1541)
MNTTVSCELHLRLVVSSESSLPVPAGLRYDTADPYAVH
ATFHTGAEETVEWVFARDLLAEGLHRPTGTGDVRVWPSRSHGQGVVCIALSSPEGEALLE
APARALESFLKRTDAAVPPGTEHRHFDLDQELSHILAES
>gi|345854610|ref|ZP_08807425.1| regulator [Streptomyces zinciresistens K42]
MNTTVSCELHLRLVVSSESSLPVPAGLRYDTADPYAVHATFHTGAEETVEWVFARDLLAEGLHRPTGTGD
VRVWPSRSHGQGVVCIALSSPEGEALLEAPARALESFLKRTDAAVPPGTEHRHFDLDQELSHILAES
>gi|302527908|ref|ZP_07280250.1| regulator [Streptomyces sp. AA4]
MRNDHVTLRSTAVFDLLAPRTPAVPVKVELRYDTRDPYAVVAAFRTGRAGWVEWVYARDLLADGLLADAG
DGDVRIRPSVEDPESVLIELNSPSGHAMFEASAQELADFLDRTYDVVLPGNEHLWVDVDDALTHLIPNDL
A
>gi|271967422|ref|YP_003341618.1| unnamed protein product [Streptosporangium roseum DSM 43021] (Sros_6145)
mnsatvsaelglrlvvpdrttvpllaglsytaddpyairmafhvgndepvewifarellt
vgivrrvgdgdvqvwparadgertlhisltspfgqalfevplaplteflhrtyelvpagr
etdfmdldaelsnmlwss
>gi|72162510|ref|YP_290167.1| unnamed protein product [Thermobifida fusca YX] (Tfus_2111)
MSSSGTSITCEVGLQLIVPDRAPVPLVARLDYSVDDPYAIRAAFHVGDDEPV
EWIFARELLTVGIIRETGEGDVRIWPSQDGKERMVNIALSSPFGQARFHAQV
APLSEFLHRTYELVPAGQESDYIDIDAEIAEHLS
>gi|296270068|ref|YP_003652700.1| sporulation and cell division protein SsgA [Thermobispora bispora DSM 43833] (Tbis_2095)
mnsitvsaelglrlvvpdrttipltasltytatdpyairmafhvgndepvewifarellt
igmvrrvgegdvqvwpartegeqslnisltspfgqalfevplapltaflnrtyelvpagr
esdfmdldqelsnmlwss

>gi|269126283|ref|YP_003299653.1| sporulation and cell division protein SsgA [Thermomonospora curvata DSM 43183] (Tcur_2048)
mdssrtisaelglrlvvpdrttvpllaeveyatddpyairmsfyvgndepvewifarell
tvgivrrvgdgdvqvwpaepggdrivhialsspfgdalfevplpplaeflhrtyravpag
reaefidmeaelenllwps
>gi|330466623|ref|YP_004404366.1| sporulation and cell division protein ssga [Verrucosispora maris AB-18-032] VAB18032_13260
MSVIRPTTVEVETSLRLVAPDATALPVRASLRYDPADPYAVHVLFHAESAGGEAVSWSFARELLVTGLDE
PAGIGDVRVWPWATPRGDFVALALSSPDGNALFEVPRSVLVRFLRRTYVVVPRGREAEHLDVDTAVNRLL
AGR

Supplemental Data file 4. SsgA protein sequences.

>gi|395772008|ref|ZP_10452523.1| regulator [Streptomyces acidiscabies 84-104]
MNFLVSEELSFRIPVGLEYEPDDPYAVRLTFHLPGDAPVTWTFGRELLIDGVGRPCGEGDVRVAPTDPES
LGEVLIRLQVGADQAVFRSSAAPLIAFLDRTDRVVPLGQEGSFADFDAHLDEALGRILAEEQSAG
>gi|11066161|gb|AAG28482.1|AF195771_1 SsgA [Streptomyces albus]
MSFLVSEELAFRIPVELRYETVDPYAVRLTFHLPGDAPVTWVFGRELLVEGVLDAAGDGDVRVCPVGQTA
TREVHITLQVGSEQALFRVGKAPLLAFLDRTDQGLSLGSERAHADFDSHLDDALNRSLAEEQSAG
>gi|291452710|ref|ZP_06592100.1| SsgA [Streptomyces albus J1074]
MSFLVSEELAFRIPVELRYETVDPYAVRLTFHLPGDAPVTWVFGRELLVEGVLDAAGDGDVRVCPVGQTA
TREVHITLQVGSEQALFRVGKAPLLAFLDRTDRLVPLGSERAHADFDSHLDDALNRILAEEQSAG
>gi|398784277|ref|ZP_10547541.1| SsgA protein [Streptomyces auratus AGR0001]
MSFVVSDELAFRIPVELDFASADPFAVRLTFDLPGDAPVTWAFGRELLLDGLSRPSGEGDVRIEPASP
EHLSDVFISLQVGSERALFRVSAAPLVAFLDRTDRLVPLGKEEVCDTLEAVLDRILTEAPAG
>gi|29830810|ref|NP_825444.1| cell division protein, regulatory protein [Streptomyces avermitilis MA-4680]
MSFLVSEELSFRIPVELRYETCDPFAVQLTFHLPGDAPVTWTFGRELLIDGVGRPCGDGDVHIAPADREA
FGEVLIRLQVGGDHALFRSGAVPLVTFLDRTDKLVPLGQECSLADFDAHLDEALDRILAEEQSAG
>gi|374988085|ref|YP_004963580.1| SsgA protein [Streptomyces bingchenggensis BCW-1]
MTFLVSQDLSFRIPVELSYDSSDPYAVEITFHLPGDAPVSWAFARELLLDGLSKPSGEGDVRIAPASPEG
LSDVFIRLQVGAERALFRAGAAPLVAFLDRTDRVVPFGQEQAACDPVGDLDAELDRILAEDRYAG
>gi|418467927|ref|ZP_13038780.1| regulator [Streptomyces coelicoflavus ZG0656]
MSFLVSEELSFRIPVELRYETRDPYAVRLTFHLPGDAPVTWAFGRELLVDGVGRPCGDGD
VRIAPVDPEPLAEVLIRLQVGSDQALFRSSAAPLVAFLDRTDKLVPLGQEGALADFDSHLDEALDRILAE
EQSAG
>gi|21222332|ref|NP_628111.1| regulator [Streptomyces coelicolor A3(2)]
MSFLVSEELSFRIPVELRYETRDPYAVRLTFHLPGDAPVTWAFGRELLVDGVGRPCGDGDVRIAPVEPE
PLAEVLIRLQVGSDQALFRSSAAPLVAFLDRTDKLVPLGQEGALADFDSHLDEALDRILAEEQSAG
>gi|185534933|gb|ACC77835.1| SsgA [Streptomyces clavuligerus]
MNFLVSEELSFRIPVELRYETDDPYAVRMTFHLPGDAPVTWAFSRDLLVGGVTGPTGDG
DVHIAPTGPGRRADLGIRLQVGQERAYFVVGAPPVVAFLDRTDRLVPLGQERAYGNCAGDLDSALCGILA
EEQNAG
>gi|185534923|gb|ACC77830.1| SsgA [Streptomyces collinus]
MSFLVSEELSFRIPVELRYESSDPYAVRLTFHLPGDAPVTWAFGRELLIDGVGRPCGEGD
VRVTPVEPDALGEVLIRLQVGSDQALFRSSTAPLVAFLDRTDKLVPLGQEGSLADFDAHLDEALDRILAE
QGAG
>gi|408530778|emb|CCK28952.1| hypothetical protein BN159_4573 [Streptomyces davawensis JCM 4913]
MSFLVSEELSFRIPVELRYATSDPYAVRLTFHLPGDAPVTWAFGRELLIDGVGRACGEGD
VRVSPADPETLGDVLIRLQVGGDHALFRSSSAPLIAFLDRTDKLVPLGQEGALSDFDASLDEALDRILAE
EQSAG
>gi|185534927|gb|ACC77832.1| SsgA [Streptomyces diastatochromogenes]
MSFLVSEELSFRIPVELRYETCDPYAVRLTFHLPGDAPVTWAFGRELLIDGVGRPCGEGD
VHIAPVDAEVLGEVLIRLQVGCDHALFRSSTPPLVAFLDRTDKLVPLGQEGALADFDAHLDEALDRILAE
EQNAG
>gi|185534919|gb|ACC77828.1| SsgA [Streptomyces filamentosus]
MSFLVSEELSFRIPVELRYEVGDPYAIRMTFHLPGDAPVTWAFGRELLLDGLNSPSGDGD
VHIGPTEPEGLGDVHIRLQVGADRALFRAGTAPLVAFLDRTDKLVPLGQEHTLGDFDGNLEDALGRILAE
EQNAG
>gi|185534929|gb|ACC77833.1| SsgA [Streptomyces filipinensis]
MSFLVSEELSFRIPVELRYETCDPYAVRLTFHLPGDAPVTWAFGRELLIDGVGRPCGEGD
VHIAPADSEVLGEVLIRLQVGCDQALFRASTPPLVAFLDRTDKLVPLGQEGALADFDAHLEEALDRILAE
EQSAG

>gi|357412337|ref|YP_004924073.1| sporulation and cell division protein SsgA [Streptomyces flavogriseus ATCC 33331]
MSFLVSEELSFRIPVELRYEVSDPYAIRMTFHLPGDAPVTWAFGRELLLDGLNSPSGDGDVHIGPIEPEG
LSDVHIRLQVGADRALFRAGTAPLVAFLDRTDKLVPLGQEHTLGDFDGNLEEALGRILAKEQNAG
>gi|185534925|gb|ACC77831.1| SsgA [Streptomyces fradiae]
MSFLVSEELSFRIPVELGYETCDPYAVRLTFHLPGDAPVTWAFGRELLVDGVGRPCGDGD
VRIAPVDPEPLAEVLIRLQVGTDQALFRSSAAPLVAFLDRTDKLVPLGQEGALADFDAHLDEALDRILAE
EQNAG
>gi|291438568|ref|ZP_06577958.1| regulatory protein [Streptomyces ghanaensis ATCC 14672]
MSFLVSEELSFRIPVELRYDTCDPYAVRLTFHLPGDAPVTWAFGRELLVDGVGRPCGEGDVRVSPVDPD
TLGEVLIRLQVGSDQALFRSSTAPLVAFLDRTDKLVPLGQEGALADFDAHLDDALDRILAEQGAG
>gi|411003756|ref|ZP_11380085.1| SsgA protein [Streptomyces globisporus C-1027]
MSFLVSEELSFRIPVELRYEVGDPYAVRMTFHLPGDAPVTWAFGRELLLDGLNSPSGDGDVHIGPTEPEG
LGDVHIRLQVGADRALFRAGTAPLVAFLDRTDKLVPLGQEHTLGDFDGNLEDALGRILAEEQNAG
>gi|11066159|gb|AAG28481.1|AF195770_1 SsgA [Streptomyces goldeniensis]
MSFLVSEELSFRIPVELRYETCDPYAVRLTFHLPGDAPVTWAFGRELLIDGGPRPCGDGDVHIAPADPET
FGEVLIRLQVGSDQAMFRVGTAPLVAFLDRTDKIVPLGQERSLADFDALLDEALDRILAEEQNAG
>gi|185534935|gb|ACC77836.1| SsgA [Streptomyces granaticolor]
MSFLVSEELSFKIPVELRYETRDPYAVRMTFHLPGDAPVTWAFGRELLLDGINRPSGDGD
VHIAPTDPERLSDVSIRLQVGGDRALFRASAPPLVAFLDRTDKLVPLGQERTLGDFEDHLEAALGRILAE
ENAGPA
>gi|302559712|ref|ZP_07312054.1| conserved hypothetical protein [Streptomyces griseoflavus Tu4000]
MSFLVSEELSFRIPVELRYDTCDPYAVRLTFHLPGDAPVTWAFGRELLIDGVGRPCGEGD
VRVSPLDPEALGEVLIRLQVGGDQALFRSSTAPLVAFLDRTDKLVPLGQEGALADFDAHLDDALDRILAE
QSAG
>gi|182437448|ref|YP_001825167.1| SsgA protein [Streptomyces griseus subsp. griseus NBRC 13350]
MSFLVSEELSFRIPVELRYEVGDPYAIRMTFHLPGDAPVTWAFGRELLLDGLNSPSGDGDVHIGPTEPE
GLGDVHIRLQVGADRALFRAGTAPLVAFLDRTDKLVPLGQEHTLGDFDGNLEDALGRILAEEQNAG
>gi|326778104|ref|ZP_08237369.1| sporulation and cell division protein SsgA [Streptomyces griseus XylebKG-1]
MSFLVSEELSFRIPVELRYEVGDPYAIRMTFHLPGDAPVTWAFGRELLLDGLNSPSGDGDVHIGPTEPEG
LGDVHIRLQVGADRALFRAGTAPLVAFLDRTDKLVPLGQEHTLGDFDGNLEDALGRILAEEQNAG
>gi|329938685|ref|ZP_08288081.1| SsgA-like sporulation/cell division regulator [Streptomyces griseoaurantiacus M045]
MSFLVSEELSFRIPVELRYETCDPFAVRLTFHLPGDAPVTWAFGRELLVDGVGRPCGDGDVHIA
PADPHAIGEVLIRLQVGADRAVFKSGAAPLVAFLDRTDKLVPLGQERSLADFDTLLDEALDRILAEEQSA
G
>gi|386841227|ref|YP_006246285.1| regulator [Streptomyces hygroscopicus subsp. jinggangensis 5008]
MSFLVSEELSFRIPVELRYETCDPYAVRLTFHLPGDAPVTWAFGRELLIDGVGRPCGEGD
VHIAPADSEVLGEVLIRLQVGGDQALFRSSAPPLVAFLDRTDKLVPLGQEGALADFDAHLDEALDRILAE
EQSAG
>gi|429201636|ref|ZP_19193088.1| hypothetical protein STRIP9103_04759 [Streptomyces ipomoeae 91-03]
MSFLVSEELSFRIPVELRYETCDPYAVRLTFHLPGDAPVTWAFGRELLIDGVGRPCGDGD
VHITPADPETFGEVLIRLQVGTDQALFRVGTAPLVAFLDRTDKLVPLGQERSLADFDTLLDEALDRILAE
EQSAG
>lividans_TK24
MSFLVSEELSFRIPVELRYETRDPYAVRLTFHLPGDAPVTWAFGRELLVDGVGRPCGDGD
VRIAPVEPEPLAEVLIRLQVGSDQALFRSSAAPLVAFLDRTDKLVPLGQEGALADFDSHLDEALDRILAE
EQSAG
>gi|11066163|gb|AAG28483.1|AF195772_1 SsgA [Streptomyces netropsis]
MSFLVSEELSFKIPVELRYETRDPYAVRMTFHLPGDAPVTWAFGRELLLDGINRPSGDGD
VHIAPTDPEGLSDVSIRLQVGADRALFRAGAPPLVAFLDRTDKSVPLGQEQTLGDFEDSLEAALGKILAE

EQNAG
>gi|297193350|ref|ZP_06910748.1| SsgA protein [Streptomyces pristinaespiralis ATCC 25486]
MRFLVSEELSFRIPVGLDYETGDPYAVRMTFHLPGDAPVTWAFGRELLLDGINGPSGDG
DVHIAPVGPESLSDVIIRLRVGGDSAMFRAGAAPLVAFLDRTDKLLPMGQELTLGDFELSLEEALGRILA
EENAG
>gi|185534931|gb|ACC77834.1| SsgA [Streptomyces ramocissimus]
MSFLVSKELSFRIPVELSYEAADPYAVRLTFHLPGDAPVTWAFGRELLIDGVGRPCG
AGDVRVEPTDPDTLGEVLISLQVGTDQALFRVSTAPLVAFLDRTDKLVPLGQEGAFSDFDTHLDQALGRI
LAEEQSAG
>gi|441142623|ref|ZP_20962491.1| SsgA protein [Streptomyces rimosus subsp. rimosus ATCC 10970]
MSFLVSEELAFRIPVELFFDSADPYAVSFTFDLPGDAPVTWAFSRELLLDGLSKPSGEGDVRIGPASSEH
LSDVFIGLQVGDERALFRAGAPPLVAFLDRTDRLVPIGEEEVCDTLDSTLDRILSEAQKAG
>gi|239989179|ref|ZP_04709843.1| SsgA protein [Streptomyces roseosporus NRRL 11379]
MSFLVSEELSFRIPVELRYEVGDPYAIRMTFHLPGDAPVTWAFGRELLLDGLNSPSGDGDVHIGPTEPE
GLGDVHIRLQVGADRALFRAGTAPLVAFLDRTDKLVPLGQEHTLGDFDGNLEDALGRILAEEQNAG
>gi|291446185|ref|ZP_06585575.1| ssgA [Streptomyces roseosporus NRRL 15998]
MSFLVSEELSFRIPVELRYEVGDPYAIRMTFHLPGDAPVTWAFGRELLLDGLNSPSGDGDVHIGPTEPE
GLGDVHIRLQVGADRALFRAGTAPLVAFLDRTDKLVPLGQEHTLGDFDGNLEDALGRILAEEQNAG
>gi|290959050|ref|YP_003490232.1| unnamed protein product [Streptomyces scabiei 87.22]
MSFLVSEELSFRIPVELRYETCDPYAVRLTFHLPGDAPVTWAFGRELLIDGVGRPCGDGD
VHIAPADPETFGEVLIRLQVGTDQAMFRVGTAPLVAFLDRTDKLVPLGQERSLADFDTLLDEALDRILAE
EQSAG
>gi|408825596|ref|ZP_11210486.1| sporulation and cell division protein SsgA [Streptomyces somaliensis DSM 40738]
MSFIVSEELSFRIPVELGYETRDPFAVRMTFHLPGDAPVTWTFGRELLVDGINDATGDGDVRIEPSETEE
LSDVHIRLRVGADQALFRASAPPLVAFLDRTDKLVPLGQECSLGDFDEGLDEVLGRILADENAG
>gi|302535619|ref|ZP_07287961.1| SsgA protein [Streptomyces sp. C]
MSFLVSEELAFRIPVELRYEARDPYAVRLTFHLPGDAPVTWAFGRELLLDGINKPSGDGD
VHIAPTDPDELSDVHIRLQVGGDRALFRAGAGPLVAFLDRTDRLVPLGQERNLGDFEESLDQALGRILSE
SQHDERKAG
>gi|185534943|gb|ACC77840.1| SsgA [Streptomyces sp. Che26]
MSFLVSEELSFRIPVELRYEVGDPYAIRMTFHLPGDAPVTWAFGRELLLDGLNSPSGDGD
VHIGPTEPEGLGDVHIRLQVGADRALFRAGTAPLVAFLDRTDKLVPLGQEHTLGDFDGNLEDALGRILAE
EQNAG
>gi|294630283|ref|ZP_06708843.1| regulatory protein [Streptomyces sp. e14]
MSFLVSEELSFRIPVELRYETCDPYAVRLTFHLPGDAPVTWAFGRELLIDGVGAPCGDGD
VRISPADSDSLSEVLIRLQVGPDRALFRSSTAPLVAFLDRTDKLVPLGQEGALADFDAHLDEALDRILAE
EQSAG
>gi|185534941|gb|ACC77839.1| SsgA [Streptomyces sp. Gre54]
MSFLVSEELSFRIPVELRYEVSDPYAIRMTFHLPGDAPVTWAFGRELLLDGLNSPSGDGD
VHIGPTEPEGLSDVHIRLQVGADRALFRAGTAPLVAFLDRTDKLVPLGQEHTLGDFDGNLEEALGRILAK
EQNAG
>gi|254384822|ref|ZP_05000159.1| SsgA [Streptomyces sp. Mg1]
MSFLVSEELAFRIPVELGYEARDPYAVRLTFHLPGDAPVTWAFGRELLLDGINKPCGDGDVHIAPTSPEG
LSDVHIRLQVGCDRALFRASAAPLVAFLDRTDRLVPLGQERNLGDFEESLDEALCKILAQSRQNEQNAG
>gi|302520469|ref|ZP_07272811.1| regulator [Streptomyces sp. SPB78]
MSFLVSEELSFRIPVELRYEAADPYAVRLTFHLPGDAPVSWTFGRELLADGIDEACGDGD
VRVSPVEPEPLLDGEGPGELHIRLQVGTDQALFRVGMAPIVAFLDRTDRLVPLGEERACADFESHLDDAL
HRILAEEPSAG
>gi|359150781|ref|ZP_09183584.1| regulator [Streptomyces sp. S4]
MSFLVSEELAFRIPVELRYETVDPYAVRLTFHLPGDAPVTWVFGRELLVEGVLDAAGDGDVRVCPVGQT
ATREVHITLQVGSEQALFRAGKAPLLAFLDRTDRLVPLGSERAHADFDSHLDDALNRILAEEQSAG

>gi|318075542|ref|ZP_07982874.1| SsgA-like sporulation/cell division regulator [Streptomyces sp. SA3_actF]
MSFLVSEELSFRIPVELRYEAADPYAVRLTFHLPGDAPVSWTFGRELLADGIDEACGDGD
VRVSPVEPEPLLDGEGPGELHIRLQVGTDQALFRVGMAPIVAFLDRTDRLVPLGEERACADFESHLDDAL
HRILAEEPSAG
>gi|318059016|ref|ZP_07977739.1| SsgA-like sporulation/cell division regulator [Streptomyces sp. SA3_actG]
MSFLVSEELSFRIPVELRYEAADPYAVRLTFHLPGDAPVSWTFGRELLADGIDEACGDGD
VRVSPVEPEPLLDGEGPGELHIRLQVGTDQALFRVGMAPIVAFLDRTDRLVPLGEERACADFESHLDDAL
HRILAEEPSAG
>gi|345000910|ref|YP_004803764.1| sporulation and cell division protein SsgA [Streptomyces sp. SirexAA-E]
MSFLVSEELSFRIPVELRYEVSDPYAIRMTFHLPGDAPVTWAFGRELLLDGLNSPSGDGDVHIAPTEPEG
LSDVHIRLQVGADRALFRAGTAPLVAFLDRTDKLVPLGQEHTLGDFDGSLEEALGRILAKEQNAG
>gi|421740505|ref|ZP_16178755.1| sporulation and cell division protein, SsgA [Streptomyces sp. SM8]
MSFLVSEELAFRIPVELRYETVDPYAVRLTFHLPGDAPVTWVFGRELLVEGVLDAAGDGD
VRVCPVGQTATREVHITLQVGSEQALFRAGKAPLLAFLDRTDRLVPLGSERAHADFDSHLDDALNRILAE
EQSAG
>gi|295837801|ref|ZP_06824734.1| regulator [Streptomyces sp. SPB74]
MSFLVSEELSFRIPVELRYEAADPYAVRLTFHLPGDAPVSWTFGRELLADGIDEACGDGD
VRVSPVEPEPLLDGEGPGELHIRLQVGTDQALFRVGMAPIVAFLDRTDRLVPLGEERACADFESHLDDAL
HRILAEEPSAG
>gi|333025765|ref|ZP_08453829.1| putative regulator [Streptomyces sp. Tu6071]
MSFLVSEELSFRIPVELRYEAADPYAVRLTFHLPGDAPVSWTFGRELLADGIDEACGDGD
VRVSPVEPEPLLDGEGPGELHIRLQVGTDQALFRVGMAPIVAFLDRTDRLVPLGEERACADFESHLDDAL
HRILAEEPSAG
>gi|365864743|ref|ZP_09404422.1| SsgA protein [Streptomyces sp. W007]
MSFLVSEELSFRIPVELRYEVGDPYAIRMTFHLPGDAPVTWAFGRELLLDGLNSPSGDGDVHIGPTEPE
GLGDVHIRLQVGADRALFRAGTAPLVAFLDRTDKLVPLGQEHTLGDFDGNLEDALGRILAEEQNAG
>gi|185534939|gb|ACC77838.1| SsgA [Streptomyces sp. Wlb 19]
MSFLVSEELSFRIPVELRYEVGDPYAIRMTFHLPGDAPVTWAFGRELLLDGLNSPSGDGD
VHIGPTEPEGLGDVHIRLQVGADRALFRAGTAPLVAFLDRTDKLVPLGQEHTLGDFDGNLEDALGRILAE
EQNAG
>gi|297200889|ref|ZP_06918286.1| regulator [Streptomyces sviceus ATCC 29083]
MSFLVSEELSFRIPVELRYEACDPYAVRLTFHLPGDAPVTWAFGRELLIDGVGRPCGEGD
VRVSPAGSDLLGDVLIRLQVGGDQALFRSSTAPIIAFLDRTDKLVPLGQEAALADFDTHLDEALDRILAE
EQSAG
>gi|440694368|ref|ZP_20876991.1| hypothetical protein STRTUCAR8_05666 [Streptomyces turgidiscabies Car8]
MSFLVSEELSFRIPVELRYETCDPYAVRLTFHLPGDAPVTWAFGRELLIDGVGRPCGDGD
VRIAPADTDVLGEVLIRLQVGGDQALFRSGAAPLVAFLDRTDKLVPLGQERSLADFDAHLDEALDRILAE
EQSAG
>gi|185534937|gb|ACC77837.1| SsgA [Streptomyces venezuelae]
MSFLVSEELCFKIPVELRYETRDPYAVRMTFHLPGDAPVTWAFGRELLLDGINRPSGDG
DVHIAPTDPEGLSDVSIRLQVGGDRALFRASAPPLVAFLDRTDKLVPLGQERTLGDFEDNLEAALGRILA
EEQSAG
>gi|328883752|emb|CCA56991.1| putative regulator [Streptomyces venezuelae ATCC 10712]
MSFLVSEELSFKIPVELRYETRDPYAVRMTFHLPGDAPVTWAFGRELLLDGINRPSGDGDVHIAPTDPEG
LSDVSIRLQVGGDRALFRASAPPLVAFLDRTDKLVPLGQERTLGDFEDNLEAALGRILAEESAGPTA
>gi|345016399|ref|YP_004818753.1| sporulation and cell division protein SsgA [Streptomyces violaceusniger Tu 4113]
MTFLVSQDLSFRIPVELSYDSSDPYAVEITFHLPGDAPVSWAFARELLLDGLSRPTGEGDVRIAPASPEG
LSDVFIRLQVGCERALFRAGAAPLVAFLDRTDRVVPFGQEPAACDPVGDLDAELHRILAEDRFAG

>gi|302552643|ref|ZP_07304985.1| conserved hypothetical protein [Streptomyces viridochromogenes DSM 40736]
MSFLVSDELSFRIPVELRYETSDPYAVRLTFHLPGDAPVTWAFGRELLIDGVGRPCGEGD
VRVTPVEADSLGEVLIRLQVGSDQAVFRSSTAPLVAFLDRTDKLVPLGQEGSLADFDAHLDEALDRILAE
QGAG
>gi|443626097|ref|ZP_21110527.1| putative SsgA-like sporulation/cell division regulator [Streptomyces viridochromogenes Tue57]
MSFLVSEELSFRIPVELRYETCDPYAVRLTFHLPGDAPVTWAFGRELLVDGVGRPCGEGD
VRVSPAGADTLGDVLIRLQVGADQALFRSSVAPLVAFLDRTDKLVPLGQEGALADFDAHLDEALDRILAE
EQSAG
>gi|345849305|ref|ZP_08802318.1| SsgA-like sporulation/cell division regulator [Streptomyces zinciresistens K42]
MSFLVSEELSFRIPVELRYETCDPYAVRLTFHLPGDAPVTWAFGRELLVDGVGGPCGEGD
VRVSPAGAETLGDVLIRLQVGADQALFRSSVAPLVAFLDRTDKLVPLGQEGALADFDTHLDEALDRILAE
EQSAG

Supplemental Data file 5. SALP protein sequences from actinomycetes.

```
>Streptomyces_coelicolor_SsgA (SCO3926)
MSFLVSEELSFRIPVELRYETRDPYAVRLTFHLPGDAPVTWAFGRELLV
DGVGRPCGDGDVRIAPVEPEPLAEVLIRLQVGSDQALFRSSAAPLVAFLDRTDKLVPLGQ
EGALADFDSHLDEALDRILAEEQSAG*
>Streptomyces_coelicolor_SsgB (SCO1541)
MNTTVSCELHLRLVVSSESSLPVPAGLRYDTADPYAVHATFHTGAEETVEWVFARDLLAE
GLHRPTGTGDVRVWPSRSHGQGVVCIALSSPEGEALLEAPARALESFLKRTDAAVPPGTE
HRHFDLDQELSHILAES*
>Streptomyces_coelicolor_SsgC (SCO7289)
VNTVVHKTLVVQLQAGGTADRFPVLAHLAYDAADPFALTVVFSHDGRVLARWTLDREMVA
EGLTRPVGVGDVRLRPESRGMWDELRIELLGDGRADGERHRAVVFVWAAAVEAFLRETHA
VVRPGREEVRVDDFLAELTAEG*
>Streptomyces_coelicolor_SsgD (SCO6722)
LSTVIEQSVEARLVAAAPRMPSIPATLHYDRADPFAVRMTFPAPATLEGVEVCWTFSREL
LIAGMQEPNGHGDVRVRPYAYDRTVLEFHAPEGTAVIHVRSGELRRFLQAAGELVPVGLE
HLQLDLDHDLAELMRGSC*
>Streptomyces_coelicolor_SsgE (SCO3158)
MSVVEQYARAHILTDGDLPDQDDGGAIPVVLRYDPQLDPSKVCVALPGRGGRASGSREWT
FSRELLEQGLRAPAGSGEVRVWPCGRVQAVVEFHSPQGCSVVQFENKALIRFLRRTYAAT
AQPVAH*
>Streptomyces_coelicolor_SsgF (SCO7175)
MSGDHHGVQAQHSAAQALLPLSLCLSQMTGALEWEDVPAEFRYDPDHPLLVTIRFAPEGA
PPVTWHVGRDLLHEGLRTTSGLGDVQVWADTPTDRETAWLQVNAHGDIAIFSLPVPELEE
WIDRTYLHVPAGTESSRLGTDAFLSKLFDEPEASSR*
>Streptomyces_coelicolor_SsgG (SCO2924)
MHTNPTGPTVVERELELRLVLSPESGIPVPARLGYHTDDPYAVHITFHIDSGHPVHWTFA
RDLLVEGVFRPSGHGDVRVWPSKTEGRSVVLVALSSPDGDALLEAPTPQVSAWLERTLRA
VPPGTEGAQLGIDDGLAELLAR*
>Acidothermus_cellulolyticus_11B_Acel_1369
MNASVHIIEVTEFHLLLDGDSRVITARLHYDPADPYVITVHFDTGVTWVLGRDLLADGLH
MEIGDGDVHLRPEGDQLFLTLRSPSGEALLAASRSAIAGFMADTERVVPRGTEHLRVDLD
KELARLLP
>Actinoplanes_missouriensis_431_AMIS_65300
MSTIRPTTVEVETSLRLVAPDATALPVRASLRYDPADPYAVHVLFHAESAGGEAVSWSFARELLVTGLDE
PAGIGDVRVWPWATPRGDFVALALSSPDGNALFEVPRSVLVRFLRRTYVVVPRGRESDHLDVDAAVNRLL
AGR
>Actinosynnema_mirum_DSM_43827_Amir_2015
mrndhvtlrstavfdllaprtpavpvqvelrydtkdpyaviaafrtgragwvewvfardl
ladgliadagdgdvrirpaaddpevvvielsspsghamfeasaqeladfldrtydvvvpg
nehlwvdvdealthlisndls
>Actinosynnema_mirum_DSM_43827_Amir_6524
MSAESITTTTTFHLLVPGAAPAPVEAELHYEPEDPYAVAVLFHTGQGKVEWIFARDLLADGLLTPSGEGD
ILVRPASDDPERVLLELNAPTGFAILSAIAEHIAEFLDLTYDVVQPGEEDLWIDFDRELAKLVSTN
>Amycolatopsis_mediterranei_U32_AMED_5330
mrndhvtlrstavfdllaprtpavpvkvelrydtrdpyavvaafrtgragwvewvyardl
ladglladagdgdvrirpsvedpeavlielnspsghamfeasaqeladfldrtydvvlpg
nehlwvdvddalthliphdla
>Amycolatopsis_mediterranei_U32_AMED_0264
MHTDAVHQSQFVLLNESTTPVLSRLSYHAHEPFAVTVAFRTERGRWIEWTFARDLLVAGLDEPAGLGDVR
IRPDLSDDEDFLTLEIESPDGYASFELEVEDVRTFLDASTELVPLGEESAHFDVDGLIEEISNV
>Amycolatopsis_mediterranei_U32_AMED_0370
MELIYPADAVVNGRGPVPVALLYDPADPWAVTLLIAGHEWLFARELLATGLSEPAGDGDVVVRPDIGGTF
GHVLLTLTAPAGCADVALPRGTVESLLDAAEALVPRGCEGIDWDREWARLANGPAA
>Amycolatopsis_mediterranei_U32_AMED_6168
```

MELIYPADAVVNGRGPVPVALLYDPADPWAITLLIAGHEWLFARELLATGLSEPAGDGDVVVRPDTGGTF
GHLLLTLTTPAGCADVALPRGTVESLLAAAEALVPRGCEGIDWDSEWARLANGPAA
>Amycolatopsis_mediterranei_U32_AMED_7500
MADPNTVITARIDFALRTFGAGPRPVPAELEYDTLDPYAVAVVLHAGPSAVRWLFGRDLLADGLLARCGD
GDVRIGPAGDPALVLVELSSPDGAAVLEAPAKEIAAFLDRTYDVIPAGSESDWFDFDEELAKLSYQD
>Blastococcus_saxobsidens_DD2_BLASA_2304
MPGHISPITLQLIGPQSWTEVPALLCYDPADPFAVRIAFGDPGEQDPADGDEGIAWLVSRELLQAGLEHP
AGDGDVRVWPAHATADVLFLHLRAPSGEALFELSRGTVAAFLRHTEALVPTGSETGLLHLDQELDALLSN
GGPDAPGR
>Catenulispora_acidiphila_DSM_44928_Caci_2217
msssvtcelqlrlvisgdsslpvpaglrydtadpyavhatfhtggqdtvdwvfarellae
glrrptgtgdvrvwpsrsrgqaivclalsspegealleaparalesflkrtntavppgle
hrfydldaelvhlfadn
>Cellulomonas_fimi_ATCC_484_Celf_2043
MTQSSYDVVEVVAMQLIGSDASVIPVSAELSFRTSDPYTVRAVFTGPHTMSTWLLGRELLSLGMLASADA
PAGTGDVQVWRDEDPDYTLVSLNGVEGSALLAAPTEPITRFLASTEALVPLGAESARMESEITALIAALL
TA
>Frankia_alni_ACN14a_FRAAL2127
MPVAATVRYEPADPYAISIGFRTGADEVVEWTFARQLLSDGVRRPAGDGDVQVWPAAQSG
GRIVCLSLSSPSGHALFEMPRSEVLAFLRRTYSAVPLGGESDIIDLDAELALLIWGGPER
>Frankia_alni_ACN14a_FRAAL5533
MRDLSADFVTEDGARASVAGTLRYDPADPFAVELILRPGRESITWIFARTLLAEGTQSPTGTGDVRIRPT
RSEGRAVVTVTLSSPSGHADLELPRSIVTRFLAEIEEKVPAGRESEGIDWNAELRTLLRTRNP
>Frankia_alni_ACN14a_FRAAL5373
MSQRRPPVANPFESQMSAFLVQPDGTRAPLPAALRFDRADPFAVSLRLRTSTVQTVTWTFARQLLIDGAR
RPTGVGAVEVRPAFHDGTRLLVLSLKSASRHTEVQLRHEQVAAFINDTLAAVPSGAESDQVDWNAELNRF
HDDDS
>Frankia_alni_ACN14a_FRAAL6494
MLGRSVRRRSLGRGMTARLMREGRPPRAVRAVFGYDPADPIAVTLTLGGVIGTGPVVWRFARRLLADGAR
RPVGLGDVRVRPIVLRGQPALALCLCGTSGTAELELPAREVAAFLWRTYAMVSADAEAATIDWAGEFRFM
PGRPAGPEGGVAWPSGWGVGRRPSRTDGAGDPRVPAEGPW
>Frankia_alni_ACN14a_FRAAL4594
MTVFQICWRACDPLAVSMTLVSRPEHPALPQGSWVAPRDALRAGLEATVGDGDVRIGPDPGEGGVRLDLV
DGDRHSVVVLATASLRSFLDRTERLVPTGEEHTEQELDTVLANLLQT
>Frankia_sp_EAN1pec_Franean1_5158
MTIRHDSITAELALRLVVPGGAPVPVAATVRYEPADPYAVSIGFRTGADEVVEWTFARQLLSDGVRRPAG
DGDVQVWPAAQSGGRIVCLSLSSPSGHALFEMPRAEVLAFLRRTYSAVPLGGESDVIDLDAELALLIWGG
PER
>Frankia_sp_EAN1pec_Franean1_4060
MRGRPIVSNAVTMDVNATYLTGDEAGKRVPVLVTVRFDPLDPVAITLRTRVPDQRVLAWTFARELLAGGD
ERPVGEGTIRIAPVLRERRRLLTVDLPSPGGPVTVELPASRVADFVRRTYERVPASAESAVIDLDLDLDL
GLAALDRG
>Frankia_sp_EAN1pec_Franean1_2058
MQVEDVTTEFVVDDAPSRGRITVLQITWRSCDPLAVALVLVSRPDHPALPQGHWVAPRDALRAGLDGPAP
VGDGDVRITPEPTRDGIRLELADGERRSVVVLDAAPVRRFLERTERVVPAGEEHPEVALDAVLDDLLQT
>Frankia_sp_CcI3_Francci3_1359
MTIRHDSITAELALRLVVPGGAPVPVAATVRYEPADPYAISIGFRTGADEVVEWTFARQLLSDGVRRPAG
DGDVQVWPAAQSGGRIVCLSLSSPSGHALFEMPRSEVLAFLRRTYSAVPLGGESDIIDLDAELALLIWGG
PER
>Frankia_sp_CcI3_Francci3_3418
MLRHSSTPAHVVARDLSVDYVTEEGEREAIPGTLRYDPADPFAVELILRPGEEAITWIFARALLAEGIEV
PAGTGDVRIRPTRSKGQTVITVSLSSPSGHADLELAKSKVTAFLADCHGQVPAGRESNGIDWNNELHMLL
RTRNT
>Frankia_sp_CcI3_Francci3_1632
MLGKDVTAEFVVDDASSRGRITVFQICWGARDPLAVSMTLVSRPEHPALPQGSWVAPRDTLRAGLETAVG
DGDVRIGPDADRRGVRLDLVHGDRQSVVVVAASSLRSFLDRTERLVPTGQEHTEQELDAVLADLLRT
>Frankia_QA3_FraQA3DRAFT_0319
MLGKDVTAEFVVDDAASRGRVTVFQICWRACDPLAVSMTLVSRPEHPALPQGSWVAPRDALRAGLETAVG

DGDVRIGPDPGQGGVRLDLVDGDRRSVVILSTSSLRSFLDRTERLVPTGQEHTEQELDTVLANLLQT
>Frankia_QA3_FraQA3DRAFT_2919
MTIRHDSITAELALRLVVPGGAPVPVAATVRYEPADPYAISIGFRTGADEVVEWTFARQLLSDGVRRPAG
DGDVQVWPAAQSGGRIVCLSLSSPSGHALFEMPRSEVLAFLRRTYSAVPLGGESDIIDLDAELALLIWGG
PER
>Frankia_QA3_FraQA3DRAFT_5229
MLRRSVRRSTLARGMTARLMPEAEPPRPVRVVFGYDPTDPIAVALTVTGAAGADPVVWRFARRLLADGAR
RPVGLGDVRVRPIVLGGQPALALCLCGPSGTAELELPAREVAAFLWRTYAMVSADAEAAVIDWTAEFRFM
PGVGARRSRGLPGVRTPHART
>Frankia_QA3_FraQA3DRAFT_6034
MLRHSSTPAHVVVRDLSADFVTEDGARASVAGTLRYDPADPFAVELILRPGRESITWIFARTLLAEGTQG
PTGTGDVRIRPTRSEGRAVVTVSLSSPSGHADLELPRSIVTRFLAEIEEKVPAGRESEGIDWNAELRTLL
RTRNP
>Frankia_symbiont_of_Datisca_glomerata_FsymDg_2166
MQVEDVTTELVVDDAPSRGRLTVLQVNWRRADPLAVTLELISRPDHPALPRGTWVVLRDALRAGLDVPTG
TGDVRITPEPERGCIRLDLTAGELHSAVRLSMDAARAFLDATERVVPRGQERAAEELDAVLANLLEN
>Frankia_symbiont_of_Datisca_glomerata_FsymDg_2563
MTIRHDSITAELALRLVVPGGAPVPVAATMRYEPADPYAVSIGFRTGADEVVEWTFARQLLSDGVRRPAG
DGDVQVWPAAQSGGRIVCLSLSSPSGHALFEMPRSEVLSFLRRTYSVVPLGGESEVVDLDAELALLIWGG
PQT
>Geodermatophilus_obscurus_DSM_43160_Gobs_3195
minratpgytcpttlhlvgpqswtevpallsyeirdpfavritfgdhaeedgiswlvgrd
llhagldqpagegdvrlwpsrtsgdvlflhlrapsgealfevsrgtvagflqltasvvpf
geessvlqvdgelaallsnggadptgr
>Kineococcus_radiotolerans_SRS30216_Krad_3069
mpalphrktevdvdvslrlrttsgpglpvpaslhygaddpyaihavfrggdtdvewvfar
dllreglsapagagdvqvqpcsdspdgrprvllrlsspdgnaeleadesdvrrflrrada
lvppgretrhldldeliarlvs
>Kitasatospora_setae_KSE_3977
mhstvqaqmamhlvvsselsfrivvdldydgddpfavrftfhlpgdepvtwvfarellvd
glsrptgegdvhihpvgddlaevclvlrspegdalmraaappllaflartdrlvpmgqel
sggeldeelasilsttghenag
>Kitasatospora_setae_KSE_12250
mrntvrtrtamhllvpggpalrtpadldydtadplavrltfhppgdgpvewvvartllla
glsgpagegdlrisplvdaglaevaltlrspegeatlrspappllaflartgrlvplgre
qvtadldrklagilaag
>Kitasatospora_setae_KSE_14600
mgwsvrrraiprahterkalalmnttvscelhlrlivssesslpvpaglrydtadpyavh
atfhtgadetvewvfardllaeglhrptgtgdvrvwpsrshgqgvvcialsspegealle
aparalesflkrtdaavppgtehrhfdldrelshilaen
>Kitasatospora_setae_KSE_28490
magqstgvveqelelnlvlspersvpvaarlsygshdpfavyvtfhldsgtpvtwvfare
llvegtfrpcgqgdvriwptrsgrrsvlcmalsspagdalleaplptvaawlerahrlvp
pgaeleamdldgslaella
>Kitasatospora_setae_KSE_01860
mthdpyhdpyrdpaprrvppqrgsttriavhlvvevvvapalhvpvaarlrygadepytv
hldnhvdlpepvtwslsrevlltglthpaglgdmtvtpghgpdhghllltlhgeessall
rlpapalrdflrrtecvvpvgceqehvdldglverllgedgeedghrdee
>Kitasatospora_setae_KSE_06570
mavatcwstavtlphsphphlaveaglhfdsalpyavrlvfppvgrldavewvfgrdlln
egrlapaghgdvtvrpgaegevlvtlrggggeavvnvpaeivtgflvecyalvpagrehe
hldvdglvarltv
>Kitasatospora_setae_KSE_11930
mmespaelrldlvmdlllspeevfavparlvycpddpyavhvsfhvdsaqpigwffardl
lvqgllrptglgdvrvwpasvdgrrmvtlmlcspegdaflqapaalvaawvrrtlrlvpp
ggeaerarvdealdalfaatgptgaagsagvepgc
>Kitasatospora_setae_KSE_28620
mfaapapveqttharlivsthrsallrvtlrynaedplavrmvfpaeyaleavdpdtdpl

```
dgpldeafdapaepvepvadgvewvfarqlfaagldlpsgegdvhirpalgqrtmvelrs
pegvallqfdrtelrrflwhsylvvpegqelrhldcdralaella
>Kitasatospora_setae_KSE_55820
mpcdplsapsdpptaatptggsrsgavappatveevlelrialdqdlvgevrtrfryaaa
apyevrltfhlgrpdeadwvfsrdllrdglqalsgqgdvklwpaqcpchgptlhlalqsp
hgsalleasqpqvrawlertyrvvpdgsieellpsdeelaellagg
>Kitasatospora_setae_KSE_60010
mnhrntprawmrrngepdcvllldvtvapcpglrvavparlryhradpyavhldchvdle
epitwlfarellvdgldgwagigdvtirrstaaeelaialstpgqtavlhtpsapvrdfl
althrlvpagtehehldaeglmrrmlaeagdvgrfe
>Kitasatospora_setae_KSE_05120
mpaavfrsfsvtlpesaypdtrvaarlrfdpalpygvelafpphrpggeeitwrfgrdll
aegrltpagegdvrvapgpdgrilitlgalgpqaeralistpdtavaaflaeaytevppg
tetahldlapglarlla
>Kitasatospora_setae_KSE_68960
mgpvteritlhllapdgttvrldtdwryrpddpyavslefgpraagarwvlsremllasl
hgpvgdgdihfaplddgmlclalggaggtvvltaecaaiaaflaataelvplgseserid
wdggladllsa
>Micromonospora_aurantiaca_ATCC_27029_Micau_1735
MSVIRPTTVEVETSLRLVAPDATALPVRASLRYDPADPYAVHVLFHAESAGGEAVSWSFARELLVTGLDE
PAGIGDVRVWPWATPRGDFVALALSSPDGNALFEVPRSVLVRFLRRTYVVVPRGREAEHLDVDTAVNRLL
AGR
>Modestobacter_marinus_MODMU_3374
MARSSRSGCTARTTFHLVGPQSWTAVPAALVYDSSDPFAVRVRFGDGATDDVEPALYDDPDDDGGVEWLL
SRDLLRAGLTGPVGEGDVRLWPARGGLDVLFLQLRAPSGEAMFELSGAVVGDFLRETELLVPAGGESDLL
RVDEELSALLRGGTDNPTGR
>Nakamurella_multipartita_DSM_44233_Namu_3475
mydfntveyrmtatvtemvlisdptmppvhaelryrsddpfavqlllsidqspaitwvfg
rdllvegvqapagigdvqvfpvldgtvielrsgeqvasllahtqdlaafldhsieivpsg
sesayfeieadlrdlpihdafea
>Nocardioides_sp_JS614_Noca_2368
mdlpatfgydpadpwavwitfrgprdevrwavgrdlllqgltdpagegdiqlwpsidehg
raavvmelcspdgrlvtqlhthelyrfltrtlaivpvgseaidlddlvaeltgisrae
>Nocardiopsis_alba_ATCC_BAA2165_B005_0230
MNSSDITATAELGLRLVVPERTAVPLVARLDYSADDPYAIRVAFHTGEDSPVEWIFARELLTVGIVRPVG
EGDVRVWPSKSEGGERSVSISLSSPFGQAEFDTQVSPLAEFLHRTYEIVPAGQEGSFVDLDEEIQQHLHS
>Pseudonocardia_dioxanivorans_CB1190_Psed_2038
MRDVTNIICKQTVFELIAPDSTVTVNVELTYSFRDPFAVEASFRTGNSQPVVWVFARDLLADGLTEATGD
GDVTVAPIGHGQVELVLTSPSGHARFHADAAELSSFLEHTYAAVPEGAEYSWLDFDAALTELLDEKARD
>Pseudonocardia_dioxanivorans_CB1190_Psed_2715
MTTDSVQQDMFTVLHGQPAPVVTRWSYDARDPFAVTFAVRTRGDRWVEWLVARDLVVESLEGPAGHGHIR
MSPQIVQGYDIVEIEISSFDGRAILEVDRDLLRSFVDATTELVALGDEPTRMDLDSEIAKLTQGCL
>Pseudonocardia_dioxanivorans_CB1190_Psed_7011
MTLGRISQTITAHYHHLGKDGVSSSAIEIVVAYDTDDPYSVQFHFRSGSIQWFVSRDLVISGLEAASGEG
DVRVSTHGTVGGDTTILTLNNHEDPSADFTLDAYELMSFLQRTEQMVPLGAETRRVQRQIELIDWTRLM
>Saccharomonospora_viridis_DSM_43017_Svir_14890
MRNDHVTLRSTAVFDLLAPRTPPVPVKVELRYDTRDPYAVVAAFRTGRAGWVEWVFARDL
LSDGLLGEAGDGDVRIRPSMEDPDSVFVELNSPSGHAMFEASAQELADFLDQTYDVVLPG
NEHLWVDVDDALTRLIPHDLG
>Saccharomonospora_viridis_DSM_43017_Svir_01730
MDNNAVQQSQFVYLSGCETPVLSRLTYVPGAPFTVTMAFKVAPGEWVEWEFARDLLITGLHAPAGIGDIR
IRPDLPGQSDDMLVIELESPDGYAAVEISRNDVQRFVDATLARVPLGYESERLDIDTVIACLTTTRP
>Saccharopolyspora_erythraea_NRRL_2338_SeryN2_010100001839
MFDLLAPQTPAVPVQVELRYDTRDPYAVVAAFRTGRAGWVEWVFARDLLADGLIAHAGVGDVTIRPAVDD
PEVVVIELSSPSGHAVFEASAQELADFLDRTYDVVVPGNENLWVNVDDALTRLLPHDLS
>Saccharopolyspora_erythraea_NRRL_2338_SACE_1961
MRNDHVTLRSTAVFDLLAPQTPAVPVQVELRYDTRDPYAVVAAFRTGRAGWVEWVFARDLLADGLIAHAG
VGDVTIRPAVDDPEVVVIELSSPSGHAVFEASAQELADFLDRTYDVVVPGNENLWVNVDDALTRLLPHDL
```

S
>Saccharopolyspora_erythraea_NRRL_2338_SACE_5535
MKNHRTVLTSMVFKLLAPAGATAPVGVELRFESRNPYEVSLAFSAGSGGHVEWVLARDLLADGLMIDSGE
GDVRVGPDASDPAVIVVSLSSPSGQATFEADAEQLVDFLNRTYDVVPPGKEHQWMSIDEALARMLPHDLY
>Saccharothrix_espanaensis_DSM_44229_BN6_28660
MRNDHVTLRSTAVFDLLAPRTPAVPVQVELRYDTKDPYAVVAAFRTGRAGWVEWVYARDLLADGLIADAG
DGDVRIRPAADDPEVVVIELSSPSGHAMFEASAQELADFLDRTYDVVVPGNEHLWVDVDEALTHLISNDL
T
>Saccharopolyspora_spinosa_NRRL_18395_SspiN1_010100019986
MTSQNHKTIRSNVVFDLTAPAGVRSPVWVELRYESREPYAVNMSFHAGMTGQIDWVIARDLLADGLIVAS
GEGDVQIIPQPDNHELVIIALSSPSGQAAFEAHAAELADFLDETYDVVAPGDEHKWMSIDEELSRLLQND
LH
>Saccharopolyspora_spinosa_NRRL_18395_SspiN1_010100008660
MTLRSTAVFDLLAPQTPAVPVQVELRYDTRDPYAVVAAFRTGRAGWVEWVFARDLLADGLIAHAGEGDVT
IRPAVDAPEVVAIELSSPSGHAVFEASAQELADFLDRTYDVVVPGNENLWVDVDEALTRLLPHDLG
>Saccharopolyspora_spinosa_NRRL_18395_SspiN1_010100036409
MMANDHGTVLATMIFNLVAPAGVIAPVGVQLRYDSRNPYEISMKLNVGTDGQVDWVIARDLLADGLIAEA
GEGDVRIRPRLDDPTLVMIEMSSPSGQATFEVNADQLADFVNDTYDVVAPGDEHRWMNVDEALSRLLSHN
L
>Saccharopolyspora_spinosa_NRRL_18395_SspiN1_010100037964
MMANDHGTVLATMIFNLVAPAGVIAPVGVELRYDSRNPYEISMKLNVGTDGQVDWVIARDLLADGLIAEA
GEGDVRIRPRLDASGVVVIELSSPSGQASFEVDADQLVDFVNDTYDVVAPGDEHRWMNVDEVLSRLLSHN
L
>Saccharopolyspora_spinosa_NRRL_18395_SspiN1_010100037024
MMANDHGTVLATMIFNLVAPAGVIAPVGVQLRYDSRNPYEISMKLNVGTDGQVDWVIARDLLADGLIAEA
GEGDVRIRPRLDSSGVVVIELSSPSGQASFEVNADQLADFVNDTYDVVAPGDEHRWMNVDEVLSRLLSHN
L
>Saccharopolyspora_spinosa_NRRL_18395_SspiN1_010100013985
MMANDHGTVLATMTFNLVAPAGVIAPVGVELRYDSRNPYEIAMKLNVGTDGQVDWVIARGRLADGLIAEA
GEGDVRIRPRLDDPTLVVIELSSPSGQASFEVNADQLADFVNDTYDVVEPDDEHRWMNIDETLSQLLSHN
L
>Saccharopolyspora_spinosa_NRRL_18395_SspiN1_010100035064
MVRNHRLIIRFATVFDLATPPVVSPPIQLELRYETRDPYAVGMIFNPGRNSRAHCMIARDLLAAGLVSRS
GEGNVRISPRPDNPELVVIALTAPSGQGVFETRTDVLARFLNDTYAIIASGDEHRWLSVDDALSRVLLND
RRNDRSPAEHD
>Saccharopolyspora_spinosa_NRRL_18395_SspiN1_010100022770
MMANDHGTVLATMTFNLVAPAGAIAPVGVELRYDSRNPYEIAMKLNVGTDGQVDWVIARDLLADGLIAEA
GEGDVRIRPRLDSSGVVVIELSSPSGQASFEVRPRLDASGVVVIELSSPSGQASFEVDADQLVDFVNDTY
DVVAPGDEHRWMNVDEVLSRLLSHNL
>Saccharopolyspora_spinosa_NRRL_18395_SspiN1_010100038174
MKGDVMADLAFVANDHGTVLATMIFNLVAPAGVIAPVGVELRYDSRNPYEIAMKLNVGADGQVDWVIARD
LLADGLIATPHRRRHTTLSTTHRRPGSTPQIRRTLQTRLPHPHRQLFSTRPRGLTSRLLGQTISRCPMQR
DRNLIHHQIKRHPLRRLHTHKHRTSHAHHRQVSVAFRSNCVKTPGRGFLASLTGVVTACGRRQGAWRCVI
SGIVARRVGPWQGPRRRRRWVTSS
>Saccharothrix_espanaensis_DSM_44229_BN6_78400
MSAESITASTTFHLLVPGVAPAPVEAELHYEPEDPYAVAVLFHTGQGKVEWIFARDLLADGLLTPSGEGD
ILVRPAADDPERVLLELNAPTGFAILSAQAEDIAEFLDLTYDVVQPGEEDLWIDFDRELAKLVSTN
>Salinispora_arenicola_CNS205_Sare_1560
MSVIRPTTVEVETSLRLVAPDATALPVRASLRYDPADPYAVHVLFHAESAGGEAVSWSFARELLVTGLDE
PAGIGDVRVWPWATPRGDFVALALSSPDGNALFEVPRSVLVRFLRRTYVVVARGREAEHLDVDTAVSRLL
AGR
>Salinispora_tropica_CNB440_Strop1600
MSVIRPTTVEVETSLRLVAPDATALPVRASLRYDPADPYAVHVLFHAESAGGEAVSWSFA
RELLVTGLDEPAGIGDVRVWPWATPRGDFVALALSSPDGNALFEVPRSVLVRFLRRTYVV
VARGREAEHLDVDTAVNRLLAGR
>Stackebrandtia_nassauensis_DSM_44728_Snas_3098
mattrpatvevdttmrfvatdfttvnvrasmrydaddpyaihvmfhpgnpdaeqvswsfa
rellaaglveptgmgdvrvwpwstprgdaialalsspdgnalfevsrslvsrflrrsyal

```
vpkgkegdsldmdatvaklladr
>Thermobifida_fusca_YX_Tfus_2111
MSSSGTSITCEVGLQLIVPDRAPVPLVARLDYSVDDPYAIRAAFHVGDDEPV
EWIFARELLTVGIIRETGEGDVRIWPSQDGKERMVNIALSSPFGQARFHAQV
APLSEFLHRTYELVPAGQESDYIDIDAEIAEHLS
>Thermobispora_bispora_DSM_43833_Tbis_2095
mnsitvsaelglrlvvpdrttipltasltytatdpyairmafhvgndepvewifarellt
igmvrrvgegdvqvwpartegeqslnisltspfgqalfevplapltaflnrtyelvpagr
esdfmdldqelsnmlwss
>Thermomonospora_curvata_DSM_43183_Tcur_2048
mdssrtisaelglrlvvpdrttvpllaeveyatddpyairmsfyvgndepvewifarell
tvgivrrvgdgdvqvwpaepggdrivhialsspfgdalfevplpplaeflhrtyravpag
reaefidmeaelenllwps
>Verrucosispora_maris_AB18032_VAB18032_13260
MSVIRPTTVEVETSLRLVAPDATALPVRASLRYDPADPYAVHVLFHAESAGGEAVSWSFARELLVTGLDE
PAGIGDVRVWPWATPRGDFVALALSSPDGNALFEVPRSVLVRFLRRTYVVVPRGREAEHLDVDTAVNRLL
AGR
>Streptomyces_venezuelae_ATCC_10712_SsgB
MNTTVSCELHLRLVVSSESSLPVPAGLRYDTADPYAVHATFHTGAEETVEWVFARDLLAEGLHRPTGTGD
VRVWPSRSHGQGVVCIALSSPEGEALLEAPARALESFLKRTDAAVPPGTEHRHFDLDTELSHILADS
>Streptomyces_venezuelae_ATCC_10712_SsgG
MQNTDNTTVERELELKLVLSPERAVPVPARLAYRTDDPYAVHITFHVGTDHPVNWTFARELLVEGVFRAC
GHGDVRIWPTKVDGRNVVLMALSSPDGDALLEAPSAQVSAWLERTLRAVPPGTESDRLGIDDGLAELLAT
TVIADELWLRDPWPSDESQDGEQ
>Streptomyces_venezuelae_ATCC_10712_SsgE
MAAAEDPEVEDHARGRIISDAPLSRPVPVDLRYDPGFSPATVRFVFPGDIEWSFPRVLLETGLRAPTRRG
DIGVWPCGRVQTVVELHKNDGDVTVVQFDTTALTRFLKHTYAAGASMTTS
>Streptomyces_venezuelae_ATCC_10712_SsgA
MSFLVSEELSFKIPVELRYETRDPYAVRMTFHLPGDAPVTWAFGRELLLDGINRPSGDGDVHIAPTDPEG
LSDVSIRLQVGGDRALFRASAPPLVAFLDRTDKLVPLGQERTLGDFEDNLEAALGRILAEESAGPTA
>Streptomyces_venezuelae_ATCC_10712_SsgD
MPAVIEQAVQARLVASAPRMETVPATLRYDRGDPYAVSMAFPPPATLEGVEVSWAFARELLVQGVERPAG
VGDVRLRPYGYDRTVVEFHAPEGVAMVHVRTSELRRFLERSQHLVPAGREHQYLDWEQDLAELLRDRP
SVEN_0129
>Streptomyces_venezuelae_ATCC_10712_SVEN_0129
MSDSSRHETSRPTAAAPVRSWHTTARQVHGGMGLPVPATFHYDPDDPWAVHIVFRLPPGRVVDWVFSRDL
LRSGTRVLSGEGDVRLWPLRDGGREGRVHLRLGQAGAFALVDVDRAGLRSWLAETYEAVPEGTEAARIDW
AAETAQLFARP
>Streptomyces_griseus_subsp_griseus_NBRC_13350_SGR_41t
MSLHHDNAMPTADDDEFAALMAASSLRAPHVRALSDPIPNEARSRLRETAEGPDTEQEPTACETQEATAD
AGDGDHSEEEPLATYRAGTWQPPALSRFLTEVYRRLPWTIDRGPHGHHTSALHIRPLLAADLLETTRATD
ILVWLSREPDNDRHYLVTPTGAALAPTLLPCATDRSPTYSSARALLRLALPQWQKEYLLDARPGTGSPDF
RLACTSLGPSASLAWVDSAIRFQTTRDECSAFRWVGDSEKHAPDADSRHRLHTLLSQAYESALVYYAARE
PGGNLRPFDQLVRTPGTADPTDTGAPAPALASLTAGRLTDLLQPVQPPHLEQLHPHPADRWTGCRISTNP
DRFPAGAWRPLLLGALADSPWAPRKTGQRLITGEAAPGSPTPQDATPMIDVPLTPAHLLIAADSAVSLDQ
AHRHTIPEQVPTPPDLDAYTAQPGSLLLLPATTSTAAGHGDNREAVHWTPRTERDFPSEGGSSGPSKFPD
NDSRTPKLWSTKTEDGVDDLEQRMQLWMRQHDTEDDRGDRILIELVYRTKDPHAVTAVFYTGTEEELEWT
FARELLADGLHTSVGLGDVIVWPGPEGPGDTQRIFIRLRPPGSTALLSLDRDDALEFLEATHPLLHNPRA
TARTAVLPAWEQELRDLICPGTGE
>Streptomyces_griseus_subsp_griseus_NBRC_13350_SGR_128
MSLHQDNAMPTAADDDEFTALMAASSLRAPHVRALSDPIPDEAQTRLRESAEGPDAEQEPTARTTQEATT
TDAGDASEDDPLDPCRLGNWPLRTFSWTMNSVLGTPEHHAPALHLRALLAAGPPATTRTTGIHEWLLREP
DNNRHYLLMHTEDPLTPTLLPCTTDPGPTYSSASAFLRMALFRRRLEHVLGARPGTGFPDYRLTSTSAGP
SAESLARTSFDIELNTTCNMYTALRLAGEPESRGSFTSRIRIHALMKQAYESALIDYAAHELAGNSQRSD
QQVRAPGTADPTRFGAPAPAHSIDRLAAGRLTDLLQPARPPHIEQLHQHPADGRTRCRIATIPDRVPAGA
WRPLLLSALSGYPWAHGTTPGGHLITGETALPGLTPQDTTAVTNRPLTPAHLLIATDSAVSLDQAHRHTI
PEQAPTPPDLDAYTAQPGSLLLLPAATATAAGHGDSRKTVNRTHDTEDPDKELTSQNSSSDPSELLTDDR
RTTKLWTTRTDDAGDGLEQRMQLWMRQHHTEDDRGDRILTQLAYRTKDPYAVTAVFYAGTEEELEWTFAR
```

ELLTDGLHTSVGIGDVIIWPGPQRPGDTQRIFIRLRPPGSTALLSLDHDDALEFLETTQPLPQNPSATAR
TAVLATWEQELRDLICPSPGE
>Streptomyces_griseus_subsp_griseus_NBRC_13350_SsgD
MSSVIEQSVQARMVASAPRMETLPATLQYDRQYPFAVRMAFPAPATLEGTEVSWEFSRELLTTGVDSPAG
QGDVRVRPFGYERTVLEFHAPEGIAMVHVRTDELRRFLQRAQELVPMGDEHRYLDLDRGLTDLLGGPR
>Streptomyces_griseus_subsp_griseus_NBRC_13350_SsgA
MSFLVSEELSFRIPVELRYEVGDPYAIRMTFHLPGDAPVTWAFGRELLLDGLNSPSGDGDVHIGPTEPE
GLGDVHIRLQVGADRALFRAGTAPLVAFLDRTDKLVPLGQEHTLGDFDGNLEDALGRILAEEQNAG
>Streptomyces_griseus_subsp_griseus_NBRC_13350_SsgE
MSPVIEVHARARLITDGPLTRPVPVDLRYDPADERRTVHIGLPDGTDWSFGRDLLERGLRTPIERGDVRV
WPCGRTQLIVELHSTDGVEVFQFEIRTLIRFLARTRAQTPATAPDTGREAPPRPPSRTTRPERGTPERGA
RTEQRDARAGRGAQTARG
>Streptomyces_griseus_subsp_griseus_NBRC_13350_SsgG
MMSIVERELELKLVLSPERSIPVPARLTYRTDDPYAVHIAFHIGSESPVHWTFARELLVEGVFRPCGHGD
VRIWPTKVAGRSVVCVALTSPDGNALLEMNSAAVAAWVERTLRVVPPGSESDRLGIDEALAELLAPLPAD
DLWLSDPWQSDDAPSQDGEA
>Streptomyces_griseus_subsp_griseus_NBRC_13350_SsgB
MNTTVSCELHLRLVVSSESSLPVPAGLRYDTADPYAVHATFHTGAEET
VEWVFARDLLAEGLHRPTGTGDVRVWPSRSHGQGVVCIALSSPEGEALLEAPARALESFLKRTDAAVPPG
TEHRHFDLDTELSHILAES
>Streptomyces_bottropensis_ATCC_25435_SBD_2172
MTVRKGEPMDHLAATDEEFDALLDASSLGAPHVVATTGDIPATARRRMAHAASRPPKSLGMPDNTSYPQP_
DDEADSRPAAPTDAEEAAQEVQLLRCGILIGATSTGKSAVLAALLAYRHLSLGSRRQLLAPRFWMDPSAA
DLLRSTLRPTGGFDEAVRSGRAQRGIPRGDGTVIFSRRQDTQPWHDMLVSVLRSPSGEPQAIPSFTDACR
QAMQLLAPDIQMHASPTLHTLGWAWAGRKQQRLQNLRHMIEPWAHPAQPLFSATPTGLLVHPAPAARLHI
ARFTTCAYTAVGGGLLLPKTGAAPQHPRCARDSSRTGRDRRQPAASGGMTLRQTLVGGVHELSAELPMLL
HIEERESLALTSSLHTRLTYRVSDPYAVEARFRADDQGETVWIFARDLLRDGLERRNGLGDVSVWPDVGK
RGRPRVFLRLSSPEGSALLSAEYADVRAFLEAGSNMVAYGAEHAHLLPALNSLETTIGELARPGRCD
